# Chemical synthesis of glycans up to a 128-mer relevant to the *O*-antigen of *Bacteroides vulgatus*

**DOI:** 10.1038/s41467-020-17992-x

**Published:** 2020-08-18

**Authors:** Qian Zhu, Zhengnan Shen, Fabrizio Chiodo, Simone Nicolardi, Antonio Molinaro, Alba Silipo, Biao Yu

**Affiliations:** 1 State Key Laboratory of Bioorganic and Natural Products Chemistry, Center for Excellence in Molecular Synthesis, Shanghai Institute of Organic Chemistry, University of Chinese Academy of Sciences, Chinese Academy of Sciences, 345 Lingling Road, Shanghai, 200032 China; 2 School of Physical Science and Technology, ShanghaiTech University, 393 Huaxia Middle Road, Shanghai, 201210 China; 3 Department of Molecular Cell Biology and Immunology, Amsterdam Infection and Immunity Institute, De Boelelaan 1108, 1081HZ Amsterdam, The Netherlands; 4 Center for Proteomics and Metabolomics, Leiden University Medical Center, Leiden, 2333 ZA The Netherlands; 5 Department of Chemical Sciences, University of Naples Federico II, Via Cintia 4, 80126 Napoli, Italy; 6 School of Chemistry and Materials Science, Hangzhou Institute for Advanced Study, University of Chinese Academy of Sciences, 1 Sub-Lane Xiangshan, Hangzhou, 310024 China

**Keywords:** Carbohydrates, Glycobiology, Natural product synthesis

## Abstract

Glycans are involved in various life processes and represent critical targets of biomedical developments. Nevertheless, the accessibility to long glycans with precise structures remains challenging. Here we report on the synthesis of glycans consisting of [→4)-α-Rha-(1 → 3)-β-Man-(1 → ] repeating unit, which are relevant to the *O*-antigen of *Bacteroides vulgatus*, a common component of gut microbiota. The optimal combination of assembly strategy, protecting group arrangement, and glycosylation reaction has enabled us to synthesize up to a 128-mer glycan. The synthetic glycans are accurately characterized by advanced NMR and MS approaches, the 3D structures are defined, and their potent binding activity with human DC-SIGN, a receptor associated with the gut lymphoid tissue, is disclosed.

## Abstract (web summary)

Glycans are abundant biomolecules that mediate essential biological processes, but their chemical synthesis is challenging. Here, the authors report the synthesis of glycans up to a 128-mer, which represents the *O*-antigen of *Bacteroides vulgatus* lipopolysaccharide and one of the longest synthetic glycans to date.

## Introduction

Glycans represent the most abundant biopolymers in nature, which constitute prominent parts of all living organisms and mediate fundamental biological processes. Short glycans (oligosaccharides) with less than 20 monosaccharide units, such as those being conjugated with lipids and proteins on the cell surfaces, are usually sufficient to fulfill various biological functions, including cell signaling, adhesion, and migration^1^. Longer glycans, such as glycosaminoglycans, display both structural properties and various physiological and pathological functions^2^. Polysaccharides, such as cellulose and starch/glycogen, serve for structural requirements or energy storage. The microbial glycans are structurally and functionally extremely diverse. For example, those on the microbe cell envelope mediate host–microbial cross-talk and play a critical role in the activation of the immune system. The development of robust strategies for the synthesis of well-defined carbohydrate structures is nowadays a top priority and has an enormous impact in various biomedical sectors; in this context, synthetic carbohydrate–vaccines, as well as glycoconjugates-based adjuvants, have been produced^3^. Paradoxically, functional studies on long glycans with precise structures have been rarely performed^4^, due to the extremely difficult acquisition of pure long glycans via either isolation from natural sources or via chemical synthesis from mono- and oligosaccharide units. Indeed, natural glycans occur as heterogeneous mixtures, or despite showing homogeneous repeating units exhibit a range of size distributions. The chemical synthesis of long glycans has been limited by the notorious low efficiency of the glycosidic coupling and protecting group manipulations. Consequently, glycans containing over 20 monosaccharide units have been synthesized only occasionally^5–18^, contributing to the general idea that chemical synthesis of long glycans are not yet of practical usefulness^19^. In this regard, a paradigm shift in the functional studies of long glycans with precise structures requires the advancement of feasible approaches to their preparation, bringing innovation to the conventional synthetic carbohydrate chemistry^20,21^.

**Fig. 1:**
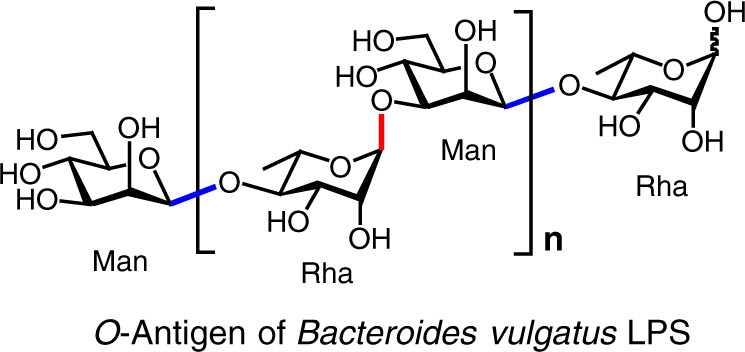
The O-antigen glycans of *Bacteroides vulgatus* LPS. The α-(1 → 3) glycosidic bond between l-rhamnopyranose (Rha) and d-mannopyranose (Man) is highlighted in red, and the β-(1 → 4) glycosidic bond between Man and Rha in blue. Source data are provided as a Source Data file.

*Bacteroides vulgatus* mpk is a commensal strain occurring commonly in the gastrointestinal tract of American and Western European population^22,23^. Recent studies demonstrated that this bacterium exerted strong immune-modulating properties leading to the prevention of colitis-induction in mouse models, and the *Bacteroides* lipopolysaccharide (LPS) induced hyporesponsiveness towards subsequent LPS-stimuli. Also, the administration of *Bacteroides* LPS re-established intestinal immune homeostasis in a mouse model for experimental colitis, thus correlating the health-promoting effects to the weak agoniztic properties of this LPS^24,25^. Further, in the human innate immune system, we demonstrated a relevant capability of the LPS to induce anti-inflammatory cytokines^26^. The *O*-antigen of *B. vulgatus* mpk LPS consists of a disaccharide repeating unit of [→4)-α-Rha-(1 → 3)-β-Man-(1 → ]_*n*_^27^ (Fig. 1). The uniqueness of this structure and the relevance of glycan motifs in modulating the immune functions prompted us to synthesize homogeneous glycoforms containing *Bacteroides O*-antigen repeating units for structural and functional studies.

Herein, we report the syntheses of glycans up to a 128-mer of the *O*-antigen of the LPS of *B. vulgatus* mpk, and the structural and functional studies of the synthetic, pure, and active glycoforms.

## Results

### Syntheses of the glycans

**Fig. 2:**
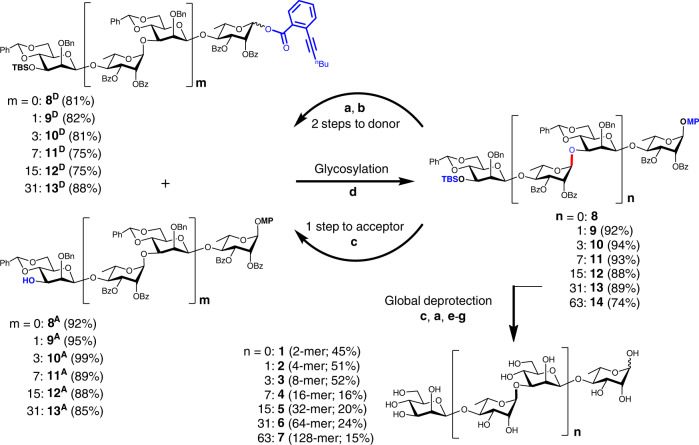
Iterative assembly to the preparation of glycans up to 128-mer (7). **a** CAN, CH_2_Cl_2_, MeCN, H_2_O, 0 °C; **b** *o*-hexynylbenzoic acid, EDCI, DMAP, CH_2_Cl_2_, rt; **c** TBAF, HOAc, THF, rt; **d** Ph_3_PAuNTf_2_ (0.1 or 0.2 equiv.), 5 Å MS, CH_2_Cl_2_, 0 °C; **e** BzCl, Et_3_N, CH_2_Cl_2_, rt; **f** H_2_, 10% Pd/C (and 20% Pd(OH)_2_), solvent, rt; **g** NaOCH_3_, CH_3_OH, rt. The experimental procedures varied slightly for substrates of different sizes (Supplementary Tables 2–5), and the hydrogenolysis (step **f**) was repeated a couple of times after step **g** until the benzyl groups were fully removed. CAN cerium (IV) diammonium nitrate, DMAP 4,4-dimethylaminopyridine, EDCI 1-(3-dimethylaminopropyl)-3-ethylcarbodiimide hydrochloride, MS molecular sieves, rt room temperature, TBAF tetrabutylammonium fluoride, THF tetrahydrofuran. The newly synthesized glycosidic bond is highlighted in red, and reacting functional groups in blue. Source data are provided as a Source Data file.

The target glycans consist of two types of glycosidic linkages, i.e., Rha-(1α→3)-Man and Man-(1β→4)-Rha. The β-mannopyranoside linkages are known to be difficult to construct in a highly stereoselective manner, while the α-rhamnopyranoside linkages are among the easiest to synthesize^28^. This knowledge forced us to fix the requisite β-mannopyranoside linkage at a disaccharide level and to extend the glycan resorting to α-rhamnopyranosylation. Such a disaccharide building block required orthogonal protection at the Rha 1-OH and Man 3-OH, which could be transformed into the corresponding donor and acceptor for a convergent glycan elongation. Disaccharide **8**, bearing MP (*p*-methoxyphenyl) and TBS (*tert*-butyldimethylsilyl) group at the reducing and non-reducing end, respectively, was found to be effective in the present work, in which the 4′,6′-*O*-benzylidene and 2′-*O*-benzyl group were used to facilitate the construction of the β-mannopyranoside linkage and the 2-*O*-benzoyl group to secure the α-rhamnopyranosylation in the subsequent glycan assembly (Fig. 2).

Although the bulky 3′-*O*-TBS group installed at the mannopyranoside donors was known to be detrimental to the β-selectivity in mannosylation^28,29^, we managed to prepare the desired disaccharide building block **8** in decagram scales with a convenient and inexpensive procedure (Supplementary Fig. 1 & Supplementary Table 1). Removal of the TBS group with TBAF (THF, rt) led to disaccharide acceptor **8**^**A**^ smoothly; at this stage, the *β*/*α* isomers resulted from the mannosylation step could be easily separated. The desired *o*-hexynylbenzoate donor **8**^**D**^ was prepared from **8** via two steps, i.e., oxidative removal of the anomeric MP group with CAN (CH_2_Cl_2_, MeCN, H_2_O, 0 °C) and condensation with *o*-hexynylbenzoic acid (EDCI, DMAP, CH_2_Cl_2_, rt). As expected, the glycosidic coupling between disaccharide donor **8**^**D**^ and acceptor **8**^**A**^ proceeded smoothly under the standard gold(I)-catalyzed conditions (0.1 equiv. Ph_3_PAuNTf_2_, 5 Å MS, CH_2_Cl_2_, 0 °C)^30^, leading to tetrasaccharide **9** in 92% yield at a decagram scale, with the α-rhamnopyranoside linkage being exclusively formed.

The convenient synthesis of 4-mer **9** from 2-mer **8** established an effective approach toward assembly of the 2^*n*+1^-mer glycans via repetition of a cycle of three transformations (Fig. 2), including (1) selective cleavage of the anomeric MP group (with CAN) and condensation of the resulting hemiacetal with *o*-hexynylbenzoic acid to provide the donor (steps **a** and **b**); (2) selective removal of the non-reducing end TBS ether (with TBAF) to provide the acceptor (step **c**); and (3) coupling of the donor and acceptor (under the catalysis of Ph_3_PAuNTf_2_) to furnish the 2^*n*+1^-mer glycan (step **d**). These transformations were found robust, so that the syntheses of 8-mer **10**, 16-mer **11**, and 32-mer **12** met with no incident in gram-scales.

Further transformations starting from 32-mer **12** (molecular weight (Mw) = 11,354) were challenged by the decreased solubility of the macromolecular substrates. Thus, removal of the TBS ether on **12** was carried out at a low concentration of 14.0 mM in THF; hydrolysis of the anomeric MP group at 2.2 mM in CH_2_Cl_2_/MeCN/H_2_O and condensation with *o*-hexynylbenzoic acid at 2.1 mM in CH_2_Cl_2_. These transformations were clean so that the desired 32-mer acceptor **12**^**A**^ (88%) and donor **12**^**D**^ (75%) were obtained with high purity after flash column chromatography on silica gel. The coupling of **12**^**A**^ and **12**^**D**^ was carried out at 7.4 mM in CH_2_Cl_2_ (0.2 equiv. Ph_3_PAuNTf_2_, 5 Å MS, 0 °C), leading to the coupled 64-mer **13** in 89% yield after purification on gel permeation chromatography (GPC).

The ready availability of 64-mer **13** (at a hundred mg scale) encouraged us to carry on the next synthetic cycle to prepare 128-mer **14**. The removal of the TBS group on **13** could only be carried out at a concentration of 1.7 mM in THF (10 equiv. TBAF, 4 equiv. HOAc, rt); hydrolysis of the anomeric MP group at 0.9 mM in CH_2_Cl_2_/MeCN/H_2_O (36 equiv. CAN, 0 °C) and subsequent condensation with *o*-hexynylbenzoic acid (7 equiv.) at 1.4 mM in CH_2_Cl_2_ (13 equiv. EDCI, 13 equiv. DMAP, rt). Upon flash chromatography on short silica gel columns to remove the excess reagents, 64-mer acceptor **13**^**A**^ and donor **13**^**D**^ were obtained with satisfactory purity (~88% yields), as indicated by nuclear magnetic resonance (NMR) analysis. The coupling of 64-mers **13**^**A**^ (80 mg, 1 equiv.) and **13**^**D**^ (1.4 equiv.) was then conducted at a concentration of 3.6 mM in CH_2_Cl_2_ under otherwise similar conditions as used in the previous glycosylation reactions, and furnished 128-mer **14** is a decent 74% yield after GPC purification.

The remaining synthetic challenge was the global deprotection of the fully protected long glycans. For 128-mer **14**, cleavage of 64 benzylidene, 64 benzyl, and 128 benzoyl groups, besides the terminal TBS and MP group, are required, involving scission of 322 C–O bonds. Fortunately, a procedure optimized on di- and tetrasaccharides **8** and **9** was successfully adapted to the deprotection of the longer congeners, that involved five sequential steps: (1) removal of the TBS ether with TBAF; (2) cleavage of the anomeric MP with CAN; (3) protection of the resulting hemiacetal with benzoyl group; (4) removal of the benzyl and benzylidene groups via hydrogenolysis; and (5) removal of the benzoyl groups with NaOCH_3_ in MeOH. The seemingly redundant step (3) for capping the resultant hemiacetal was found crucial to the subsequent hydrogenolysis (especially for the long glycan substrates), since it enabled complete removal of the detrimental impurities from the proceeding steps and avoided degradation of the aldehyde. Nevertheless, hydrogenolysis of the longer glycans starting from 16-mer **11** was found difficult to complete, because the gradually releasing hydroxyl groups could cause an unwanted decrease of the solubility in the reaction solvents. Thus, repetitive hydrogenolysis was performed after the removal of the benzoates to cleave the remaining benzyl groups in a mixture solvent of MeOH/H_2_O/HOAc. Moreover, a combination of 10% Pd/C and 10% Pd(OH)_2_ was used as the catalyst^31^. The complete and clean reactions enabled acquiring the free glycans with satisfactory purity via simple filtration through Sephadex G-25 (for 1–5) or LH-60 (for **6** and **7**).

### Structural characterization

**Fig. 3:**
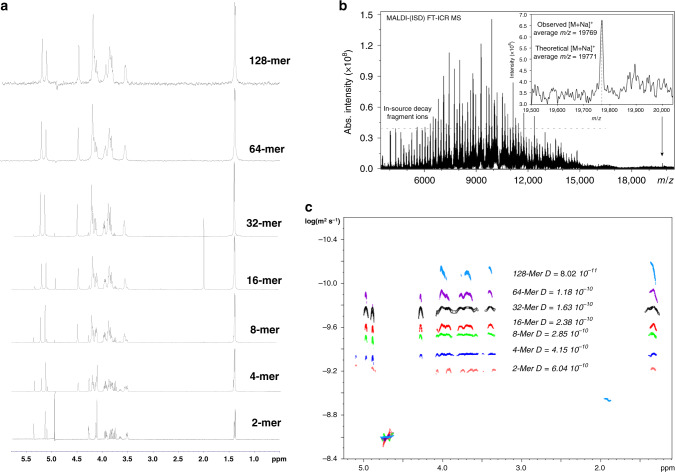
Characterization of the glycans. ∣ **a** Overlaid ^1^H-NMR spectra (acquired on Bruker 600 MHz with cryogenic probe and analyzed with Bruker Topspin) of the free glycans 1–7. **b** MALDI-FT-ICR-MS spectrum of 128-mer **7**. The observed average *m/z* of 128-mer **7**, detected as [M + Na]^+^, is in agreement with the calculated theoretical value (*m/z* 19771.1). **c** 2D diffusion ordered spectroscopy (DOSY) NMR spectra of glycans 1–7. The *x*-axis represents the ^1^H-NMR dimension; the *y*-axis represents the diffusion dimension; the double-logarithmic plot of *D* against Mw (Supplementary Fig. 13) provided a calibration curve described by the least-squares fitted linear equation Log *D* = −7873 − (0.486*Log Mw). Source data are provided as a Source Data file.

The fully protected and free glycans were well characterized by a combination of NMR and MS analyses. As for the free glycans 1–7, the ^1^H-NMR spectra (Fig. 3a) showed a characteristic set of signals of the disaccharide repeating unit and were fully assigned (Supplementary Figs. 3–7; Supplementary Table 6). The NMR resonances of the internal repeating units were coincident and clearly distinguishable from the terminal reducing and nonreducing units. The molecular size of the glycans was estimated by the integration of the ^1^H-NMR signals (ratio of internal and terminal units) and by the retention time on GPC (Supplementary Fig. 2); as for the longer size glycans, a combination of DOSY NMR and advanced MALDI-MS analyses was applied. In particular, DOSY-NMR measurements, capable of determining the molecular self-diffusion coefficients^32,33^, were successfully applied to estimate the molecular weights of the longest glycans (Fig. 3c; Supplementary Fig. 13). In particular, DOSY-derived Mw for 64-mer **6** was well consistent with the theoretical value; the average molecular mass of 128-mer **7**, complicated by its size and lability during ionization, was instead determined by MALDI-FT-ICR-MS (Fig. 3b; Supplementary Fig. 14)^34^.

### Conformation and immunological activities

**Fig. 4:**
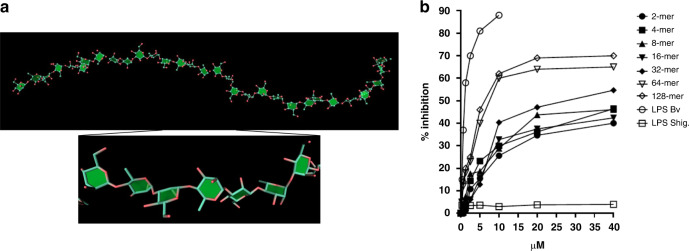
Conformation and immunological activities. **a** A representative solution conformation of the 32-mer as determined by NMR and molecular simulation (Maestro Schrödinger), prepared with SweetUnitMol^39^; **b** ELISA analysis of the competition binding of the glycans and LPS from *B. vulgatus* to the human C-type lectin DC-SIGN, with LPS from *Shigella flexneri* as a negative control. The competition experiments have been performed three times showing similar results; the graph shows data of one of these experiments^40^. Source data are provided as a Source Data file.

Together with the complete NMR assignments (Supplementary Table 6; Supplementary Figs. 3–7), molecular mechanics and dynamic simulation were carried out to assess the conformational behavior of the free glycans (Supplementary Figs. 8–12; Supplementary Table 7). The glycosidic linkages adopted Φ values in accordance with the *exo*-syn anomeric conformation and were characterized by a certain degree of flexibility. The oligomers tend to form extended, flexible structures, as confirmed by the *inter*-residue NOE contacts, diagnostic of the absence of a compact disposition of the sugar backbone (Fig. 4a). On longer glycans (i.e., 64- and 128-mer), this may lead to a tendency to pack into supramolecular assemblies, this latter also accounting for the low solubility of the longer glycans and the low sensitivity of MALDI technique for the high Mw 128-mer.

The synthetic approach allowed us to deploy a full set of pure synthetic different sized *O*-antigen fragments, which were tested for immunological activity. Given the presence of rhamnose and mannose in *B. vulgatus O*-antigen and considering the niche and the physiological roles of these commensal bacteria, we used three immune receptors of the gut-associated lymphoid tissues in the study. Thus, *B. vulgatus* LPS and their fragments were examined on a direct ELISA with three human C-type lectins (i.e., DC-SIGN, Langerin, and Dectin-1)^35,36^, showing a qualitative binding to DC-SIGN and Langerin (Supplementary Fig. 15a). Subsequently, the binding of *B. vulgatus* LPS to DC-SIGN and Langerin was tested in a quantitative ELISA competition experiment; a better binding to DC-SIGN was observed (Supplementary Fig. 15b). Following up, the synthetic glycans were tested on a competition ELISA experiment against DC-SIGN^37^. Expectedly, glycans 1–7 were shown to be able to inhibit the LPS binding to DC-SIGN in a typical dose-response curve (Fig. 4b). A better affinity for the longer glycans (**6** and **7**) was observed compared with the shorter ones. Hence, we additionally proved a selective binding of the synthetic *O*-antigens from *B. vulgatus* to DC-SIGN, adding unreported information that could explain the tolerogenic signaling induced by the commensal *B. vulgatus* in gut-associated lymphoid tissues.

## Discussion

The synthesis of glycans up to a 128-mer (**7**) consisting of disaccharide repeating unit of [→4)-α-Rha-(1 → 3)-β-Man-(1 → ]_*n*_ have been achieved. The present success is attributable to the following aspects: (1) The strategic [2^*n*^ + 2^*n*^] glycosylation enables exponential growth of the glycan sizes^17^, leading to 128-mer via only six repetitive cycles of transformations, and ready separation of the double-sized products from the remaining and decomposed substrates. The heroic syntheses of a linear 50-mer^13^ and a 100-mer^18^ via step-wide glycosylation demanded exhaustive reactions (glycosylation and capping on a solid support) at each step; while the synthesis of the 151-mer^18^ and 92-mer^14^ took advantage of multifold glycosylations to install the identical branches. (2) The overall protecting group arrangement enables robust transformations into donors and acceptors, provides appropriate solubility, and allows global deprotection at the macromolecular level. It is noteworthy that the 15% yield achieved for the deprotection of 128-mer (**14**→**7**) corresponds to an average ~99.4% yield for each cleavage of the 322 C–O bonds. Thus far, hydrogenolysis of benzyl ethers has been employed as the final step in all the synthesis of long glycans over 50-mer, however, cautiousness remains for this transformation which could require tedious exploration of the reaction conditions^38^. (3) The gold(I)-catalyzed glycosylation reaction, involving α-rhamnopyranosylation, is both stereospecific (in the presence of a neighboring-participating benzoyl group) and high-yielding. The 64-mer donor (**13**^**D**^) represents the longest glycosyl donor that has ever been employed for successful glycosylation; this might be attributed to the merits of glycosyl *ortho*-alkynylbenzoates, including easy preparation, stability, and selective activation under mild conditions^30^.

Besides the synthetic achievement, the availability of pure homogeneous glycans has allowed us to test the limits of analytical tools for the characterization of polysaccharides. Thus, the accurate confirmation of the molecular size of 64-mer (**6**) represents one of the longest glycan determined so far by DOSY-NMR, and the average molecular mass of 128-mer (**7**) represents the highest mass of a synthetic linear polysaccharide being measured by MS. Moreover, the synthetic *O*-antigen showed a selective affinity with human lectin DC-SIGN and this might be relevant information for therapeutic use against different gut-associated inflammatory diseases. We expect that the present report can inspire researches on the syntheses and thus functional studies on pure and homogenous polysaccharides for different therapeutic uses.

## Methods

### General methods for the syntheses

Reactions were carried out in glassware. Crushed 4 or 5 Å molecular sieves were activated through flame-drying under high vacuum immediately prior to use. All chemicals were purchased as reagent grade and used without further purification, unless otherwise noted. Analytical thin-layer chromatography was performed using Merck precoated silica gel 60 F-254 plates. Compound spots were visualized by UV light (254 nm) and immersion into a solution of 5% H_2_SO_4_ in ethanol, followed by hot air gun heating. Column chromatography was performed on silica gel (200–300 mesh). Gel filtration was performed on Sephadex LH-60, LH-20, or G25 (J&K). Optical rotations were obtained on Anton Paar MCP 5500 polarimeter at 589 nm (Na).

### Synthesis of 64-mer donor 13^D^

A solution of 64-mer **13** (320 mg, 14.2 μmol) in CH_2_Cl_2_/MeCN (10 mL/4 mL) was stirred at 0 °C. Ammonium ceric nitrate (281 mg, 0.51 mmol) was dissolved in H_2_O (1 mL) at 0 °C. The latter solution was added into the former via syringe, and the mixture was stirred at 0 °C for 2 h. The mixture was diluted with CH_2_Cl_2_ and washed with sat. aq. NaHCO_3_ and brine, respectively. The organic layer was dried over anhydrous Na_2_SO_4_ and concentrated in vacuo. The residue was purified by silica gel column chromatography (petroleum ether/EtOAc/CH_2_Cl_2_, 4:1.5:3) to give the corresponding crude hemiacetal (301 mg) as a yellowish solid.

To a mixture of the crude hemiacetal (301 mg, ~13.5 μmol), EDCI (32.1 mg, 0.17 mmol), DMAP (20.6 mg, 0.170 mmol), and *ortho-*hexynylbenzoic acid (20.4 mg, 0.10 mmol), was added CH_2_Cl_2_ (10 mL). The mixture was stirred at rt for 10 h, and was then diluted with CH_2_Cl_2_ and washed with sat. aq. NaHCO_3_. The organic layer was dried over anhydrous Na_2_SO_4_ and concentrated in vacuo. The residue was purified by silica gel column chromatography (petroleum ether/EtOAc/CH_2_Cl_2_, 12:1.5:3 then 4:1.5:3) to give compound **13**^**D**^ (283 mg, 88% for two steps, *α*/*β* = 2.3:1) as a white solid.

### Synthesis of 64-mer acceptor 13^A^

To a solution of 64-mer **13** (190 mg, 8.5 μmol) in anhydrous THF (5 mL), were added HOAc (2 μL, 34.9 μmol) and TBAF (1.0 M in THF, 86.0 μL, 86.0 μmol). The mixture was stirred at rt for 48 h, and was then diluted with CH_2_Cl_2_ and washed with sat. aq. NH_4_Cl and brine, respectively. The organic layer was dried over anhydrous Na_2_SO_4_ and concentrated in vacuo. The residue was purified by silica gel column chromatography (petroleum ether/EtOAc/CH_2_Cl_2_, 4:1.5:3) to give **13**^**A**^ (179 mg, ~85%; >90% purity based on ^1^H-NMR analysis) as a white solid.

### Synthesis of protected 128-mer 14

A mixture of 64-mer donor **13**^**D**^ (113 mg, 5.00 μmol) and acceptor **13**^**A**^ (80 mg, 3.58 μmol) in a Schlenk flask equipped with a Teflon-coated magnetic stir bar was dried in high vacuum at 40 °C overnight. And then 5 Å MS (100 mg) and anhydrous CH_2_Cl_2_ (1 mL) were added. The mixture was stirred at rt for 15 min, and was then cooled to 0 °C, to which Ph_3_PAuNTf_2_ (0.53 mg, 0.72 μmol) was added. The resulting mixture was stirred at 0 °C for 15 h. After addition of Ph_3_P (0.5 mg) and Et_3_N (4 μL), the mixture was filtrated through a pad of Celite. After removal of solvent, the resulting residue was dissolved in CHCl_3_ and filtrated through Millipore filter, and then GPC (CHCl_3_) was performed to give 128-mer **14** (114 mg, 74%) as a white solid.

### Synthesis of 128-mer 7

To a solution of 128-mer **14** (45 mg, 1.0 μmol) in anhydrous THF (5 mL), were added HOAc (2 μL, 34.9 μmol) and TBAF (1.0 M in THF, 86.2 μL, 86.2 μmol). The mixture was stirred at rt for 46 h, and was then diluted with CH_2_Cl_2_ and washed with sat. aq. NH_4_Cl and brine, respectively. The organic layer was dried over anhydrous Na_2_SO_4_ and concentrated in vacuo to give a residue.

A solution of the residue above (45 mg, 1.0 μmol) in MeCN/CH_2_Cl_2_ (8 mL/5 mL) was stirred at 0 °C. Ammonium ceric nitrate (242 mg, 0.44 mmol) was dissolved in H_2_O (0.5 mL) at 0 °C. The latter solution was added into the former via syringe. The mixture was stirred at 0 °C for 1.5 h, and was then diluted with CH_2_Cl_2_ and washed with sat. aq. NaHCO_3_ and brine, respectively. The organic layer was dried over anhydrous Na_2_SO_4_ and concentrated in vacuo. The residue was used in the next step without further purification.

To a mixture of the residue above (45 mg), DMAP (20 mg, 0.16 mmol), and Et_3_N (30 μL, 0.21 mmol) in CH_2_Cl_2_ (2 mL), was added BzCl (12 μL, 104 μmol). The mixture was stirred at rt overnight, and was then diluted with CH_2_Cl_2_ and washed with sat. aq. NaHCO_3_ and brine, respectively. The organic layer was dried over anhydrous Na_2_SO_4_ and concentrated in vacuo. The residue was purified by silica gel chromatography (EtOAc/CH_2_Cl_2_, 1:2) to give a white solid (40 mg).

The solid above (40 mg) was dissolved in a mixture of MeOH/CHCl_3_/H_2_O/HOAc (1.5 mL/3 mL/0.1 mL/0.15 mL) containing 10% Pd/C (80 mg, wetted with 55% H_2_O) and 20% Pd(OH)_2_/C (80 mg, wetted with 50% H_2_O). The resulting mixture was stirred under H_2_ atmosphere (1 atm) at rt for 48 h, and was then filtrated through a pad of Celite, and the Celite pad was washed with CH_2_Cl_2_/MeOH (2:1, v/v) three times. The filtrates were concentrated to give a white solid (30 mg).

The residue above (30 mg) was added into a solution of NaOCH_3_/CH_3_OH (5 mL, pH = ~11) and CH_2_Cl_2_ (4 mL). The mixture was stirred at rt for 24 h, and was then neutralized with Amberlyst 15 H^+^ resin and filtered. The filtrate was concentrated to give a residue (12 mg). ^1^H-NMR analysis indicated remaining of a few benzyl groups.

Thus, the solid above (6 mg) was dissolved in a mixture of MeOH/H_2_O/HOAc (1.5 mL/1.5 mL/0.1 mL) containing 10% Pd/C (12 mg, wetted with 55% H_2_O) and 20% Pd(OH)_2_/C (6 mg, wetted with 50% H_2_O). The resulting mixture was stirred under H_2_ atmosphere (1 atm) at rt for 24 h, and was then filtrated through a pad of Celite, and the Celite pad was washed with H_2_O and MeOH three times. The filtrate was concentrated. The residue was purified by gel filtration (Sephadex LH-60, H_2_O) to afford 128-mer **7** (3 mg, 15% over six steps) as a glassy solid or white powder after lyophilization.

### NMR analysis

For structural assignments, 1D and 2D ^1^H-NMR spectra were recorded in D_2_O at 308 K on Bruker 600 MHz equipped with a cryo probe. ROESY and NOESY experiments were recorded using data sets (*t*_1_ × *t*_2_) of 4096 × 800 points with mixing times between 100 and 700 ms. Double quantum-filtered phase-sensitive COSY experiments were performed using data sets of 4096 × 800 points. TOCSY experiments were performed with spinlock times of 100 ms, using data sets (*t*_1_ × *t*_2_) of 4096 × 800 points. In all homonuclear experiments the data matrix was zero-filled in both dimensions to give a matrix of 4 K × 2 K points and was resolution enhanced in both dimensions by a cosine-bell function before Fourier transformation. HSQC, HSQC-ROESY and HMBC experiments were measured in the ^1^H-detected mode via single quantum coherence with proton decoupling in the ^13^C domain, using data sets of 2048 × 600 points. Experiments were carried out in the phase-sensitive mode. A 60 ms delay was used for the evolution of long-range connectivities in the HMBC experiment. In all heteronuclear experiments the data matrix was extended to 4096 × 4096 points using forward linear prediction extrapolation.

### PFG-NMR analysis

All the experiments were performed on 100–200 μg of glycans solved in 500 μL of D_2_O. 1D ^1^H-NMR experiments for diffusion measurements were performed using a stimulated echo sequence with bipolar gradient pulses and one spoil gradient (stebpgp1s1d) with a diffusion time, Δ from 50 to 150 ms and a gradient duration varying from 1 to 4 ms. These parameters were optimized, kept as short as possible to minimize *T*_2_ and *T*_1_ losses, the sequence was run as 2D NMR experiment with a linear gradient incremented, in 32 steps, from 2% to 95% (between 1.8 and 32.9 G cm^−1^). The values of diffusion coefficients were calculated from the decay of the signal intensity of different protons. The normalized integral intensities of each proton were fitted to an exponential decay, according to: *I* = *I*_0_ exp − *D*_*k*_ with *k* = (G*γ*δ)^2^*(Δ − *δ*/3 − *τ*/2), where *I* is the integral intensity, normalized to the integral obtained at the lowest gradient amplitude; *I*_0_ is the signal intensity in the absence of an applied magnetic field gradient; *γ* is the magnetogyric ratio of the proton; *G* is the strength of the magnetic field gradient pulses; *δ* is their duration; *Δ* is the distance between the leading edges of the gradient pulses; and *τ* is the gradient recovery time. Diffusion rates were extracted from the slope of the straight lines obtained by plotting ln(*I*/*I0*) against the complex abscissa *k*.

### Conformational studies

Molecular mechanics calculations were performed using the MM3* force field, a dielectric constant of 80 was used. For the disaccharide structure, both Φ and Ψ were varied incrementally using a grid step of 18°, each (Φ, Ψ) point of the map was optimized using 2000 P.R. conjugate gradients. Molecular dynamic simulations were run by using the MM3* force field, bulk water solvation was simulated by using MacroModel generalized Born GB/SA continuum solvent model. All simulations were performed at 298 K, structures were initially subjected to an equilibration time of 300 ps, then a 10,000 ps molecular dynamic simulation was performed with a dynamic time-step of 1.5 fs, a bath constant of 0.2 ps and the SHAKE protocol to the hydrogen bonds. Trajectory coordinates were sampled every 2 ps, and a total of 5000 structures were collected for every simulation. Ensemble average-interproton distances were calculated using the NOEPROM program by applying the isolated spin pair approximation as described. Solvent-accessible surfaces were calculated with the Surface utility of Maestro. Conformers were visualized with Maestro, Discovery Studio Visualizer and SweetUnitMol.

### Reporting summary

Further information on research design is available in the Nature Research Reporting Summary linked to this article.

## Supplementary information

Supplementary Information

Reporting Summary

## Data Availability

The authors declare that all data supporting the findings of this study are available within the paper and its Supplementary information files, including experimental details, characterization data, and ^1^H and ^13^C NMR spectra (Supplementary Figs. 16–78) of all new compounds and MS spectra of the glycans (Supplementary Figs. 79–91). All the data are available from the authors upon reasonable request. Source data are provided with this paper.

## Source data

Source Data
