# Supplementary material for: Chemical synthesis of glycans up to a 128-mer relevant to the O-antigen of Bacteroides vulgatus
Source: Nat Commun. 2020 Aug 18;11:4142. doi: 10.1038/s41467-020-17992-x (PMC7434892; doi:10.1038/s41467-020-17992-x)
Supplement: Supplementary file 1 — Supplementary Information [file 41467_2020_17992_MOESM1_ESM.pdf]

SUPPLEMENTARY INFORMATION

Chemical synthesis of glycans up to a 128-mer relevant to the *O*-antigen of *Bacteroides vulgatus*

Zhu et al.

Supplementary Figures

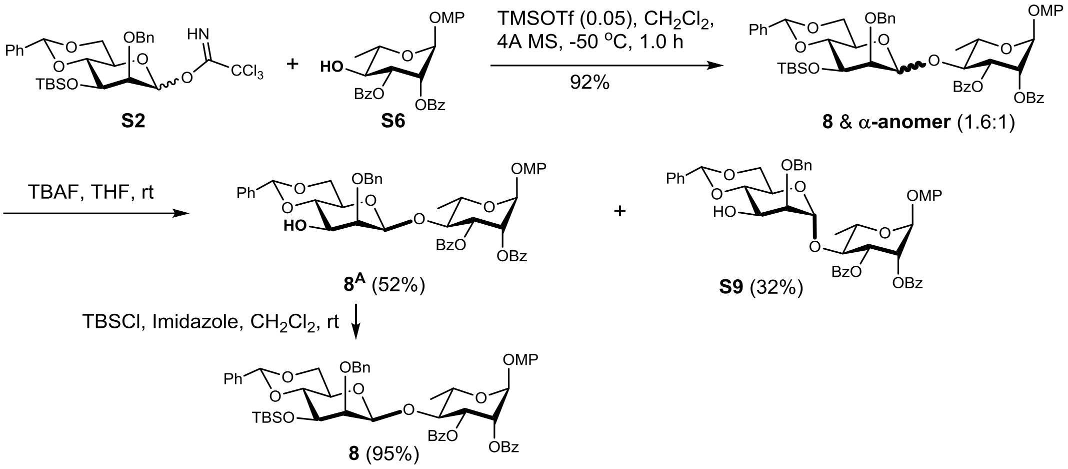

Supplementary Figure 1. A decagram-scale synthetic approach to disaccharide 8 along with acceptor 8^{A}.

a

b

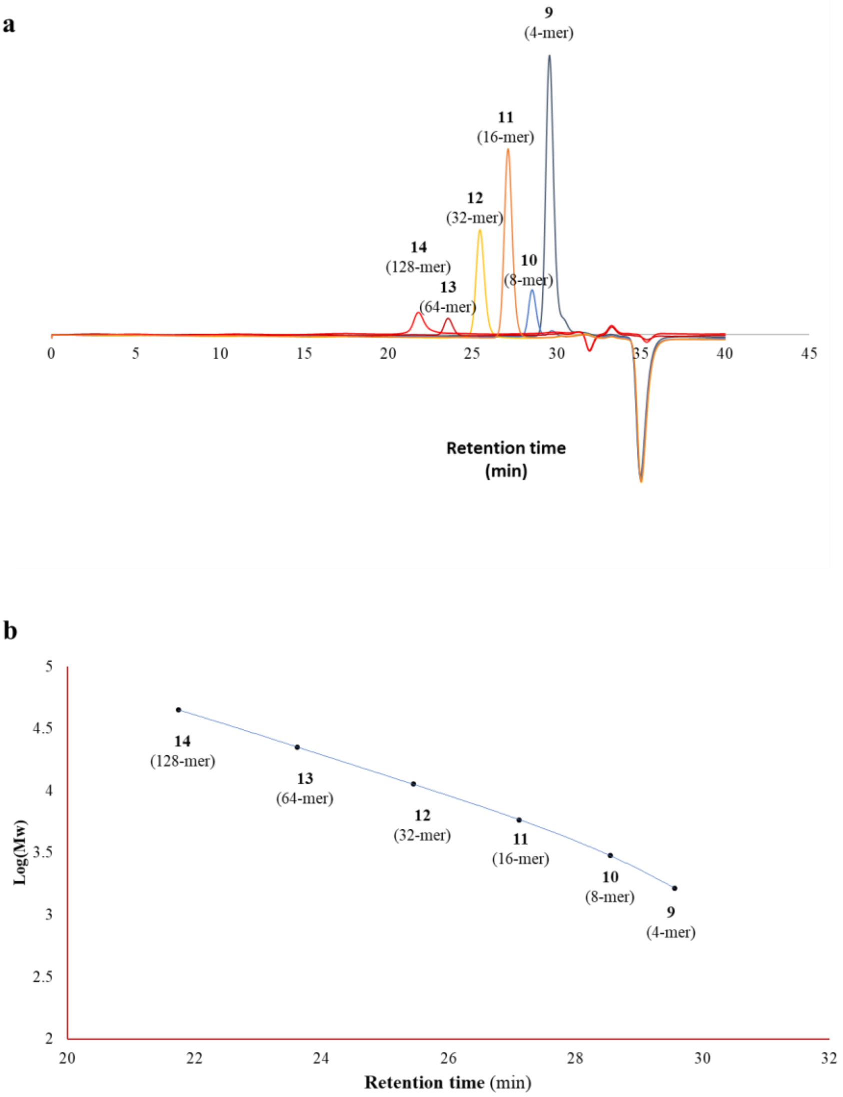

Supplementary Figure 2. Measurement of Mw by gel permeation chromatography (GPC). a Overlaid GPC traces of the fully protected glycan **9** (4-mer), **10** (8-mer), **11** (16-mer), **12** (32-mer), **13** (64-mer), and **14** (128-mer). **b** The plot of Log(Mw) over retention time. The relative molecular weights of the fully protected glycans were measured by conventional GPC, using a system equipped with a Waters 1515 Isoratic HPLC pump, a Waters 2414 refractive index detector, and a set of Waters Styragel columns HR3 (500-30,000), HR4 (5000-600,000), and HR5 (50,000-4,000,000), 7.8 × 300 mm, particle size: 5 µm. GPC measurements were carried out at 35 °C using THF as eluent with a flow rate of 1.0 mL/min. The system was calibrated with linear polystyrene standards.

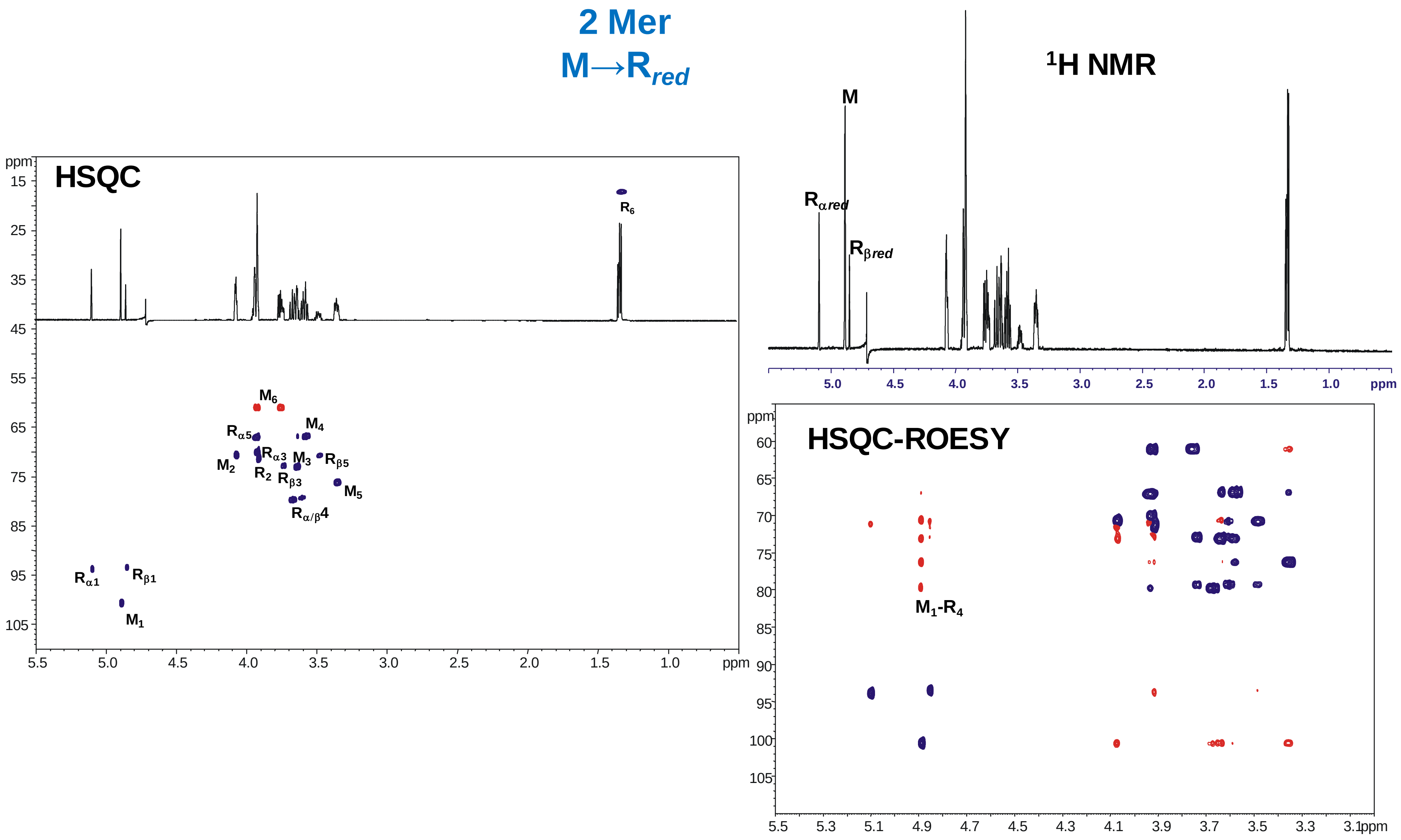

Supplementary Figure 3. 2D NMR spectra of 2-mer 1.

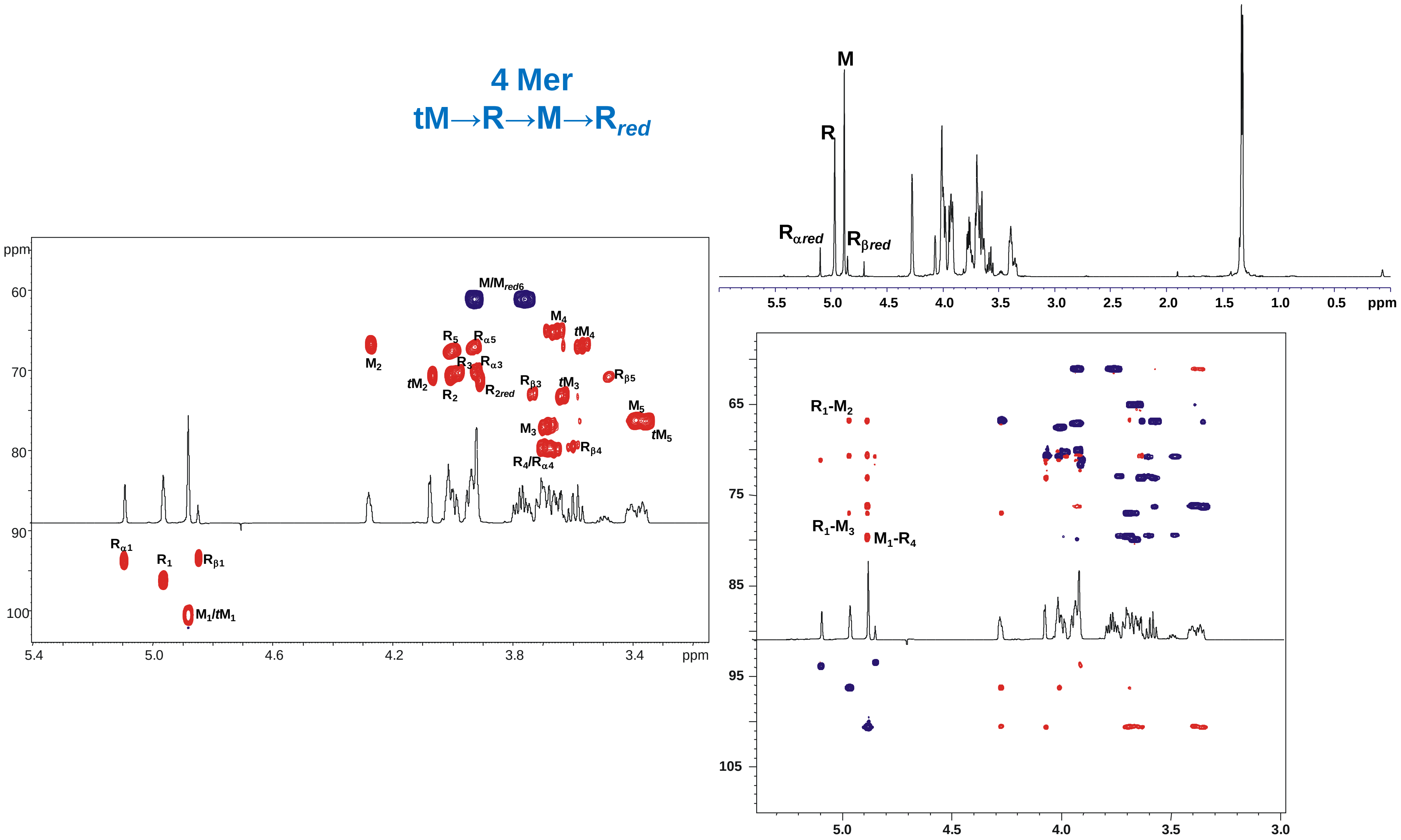

Supplementary Figure 4. 2D NMR spectra of 4-mer 2.

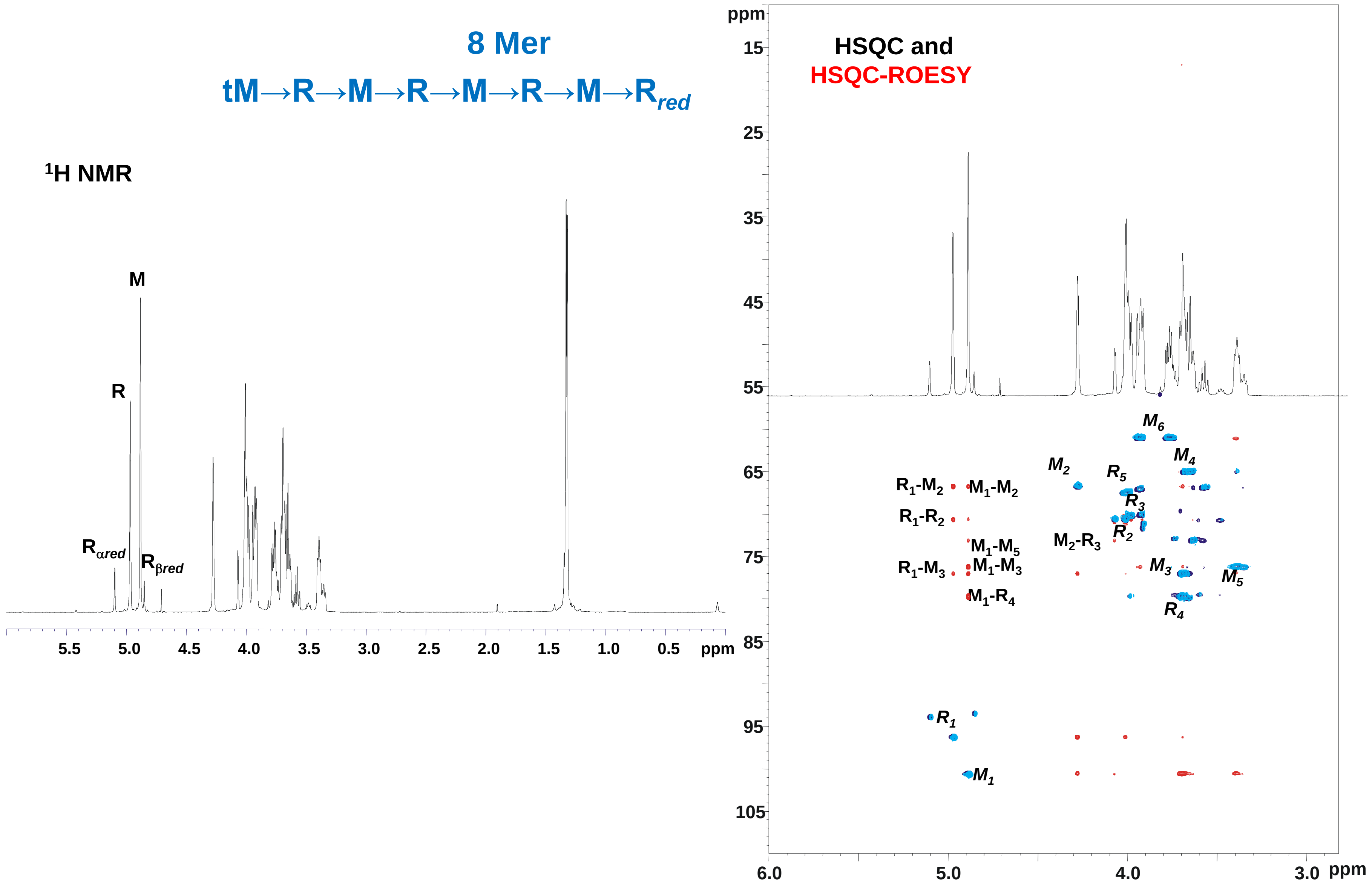

Supplementary Figure 5. 2D NMR spectra of 8-mer 3.

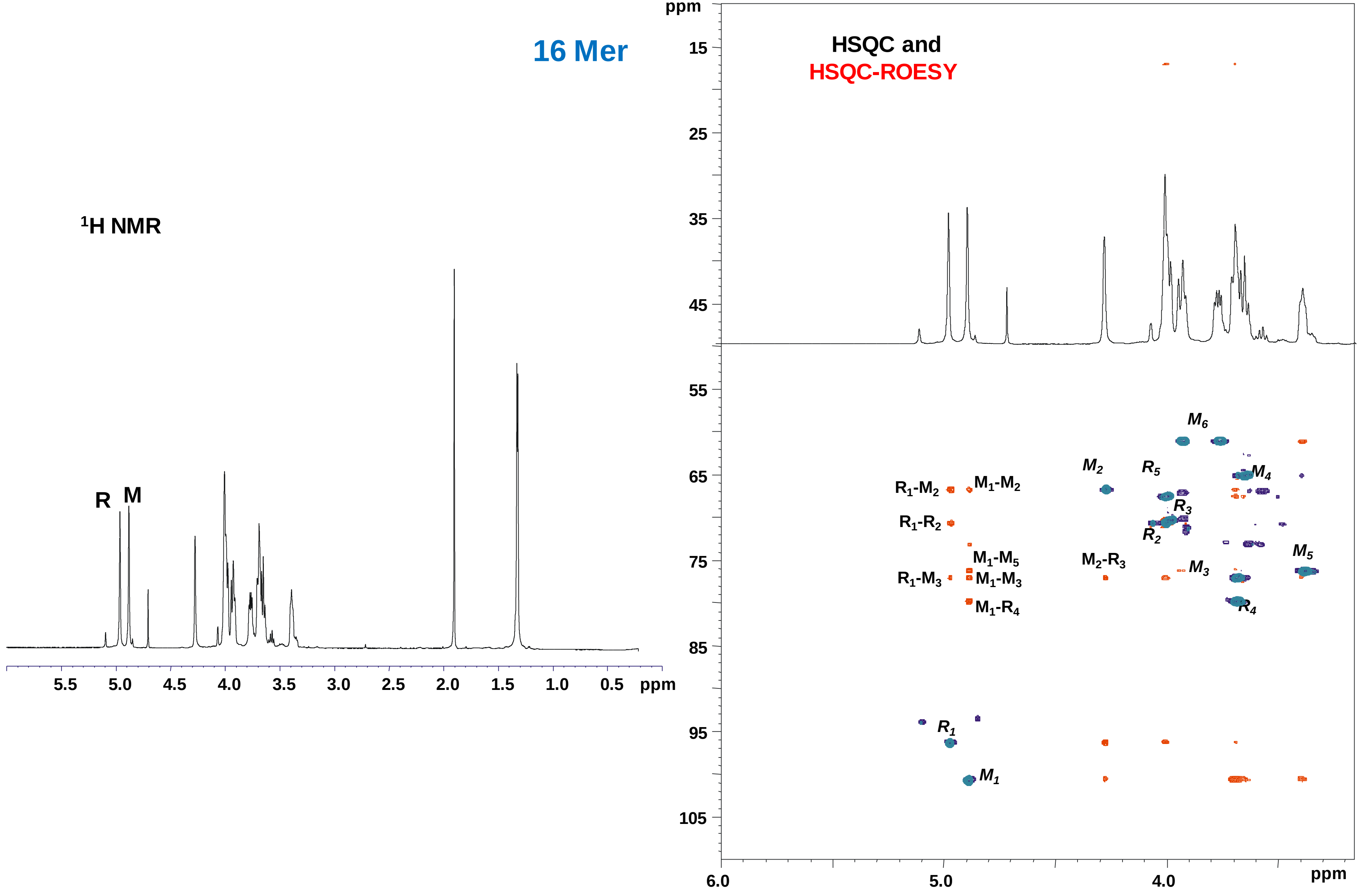

Supplementary Figure 6. 2D NMR spectra of 16-mer 4.

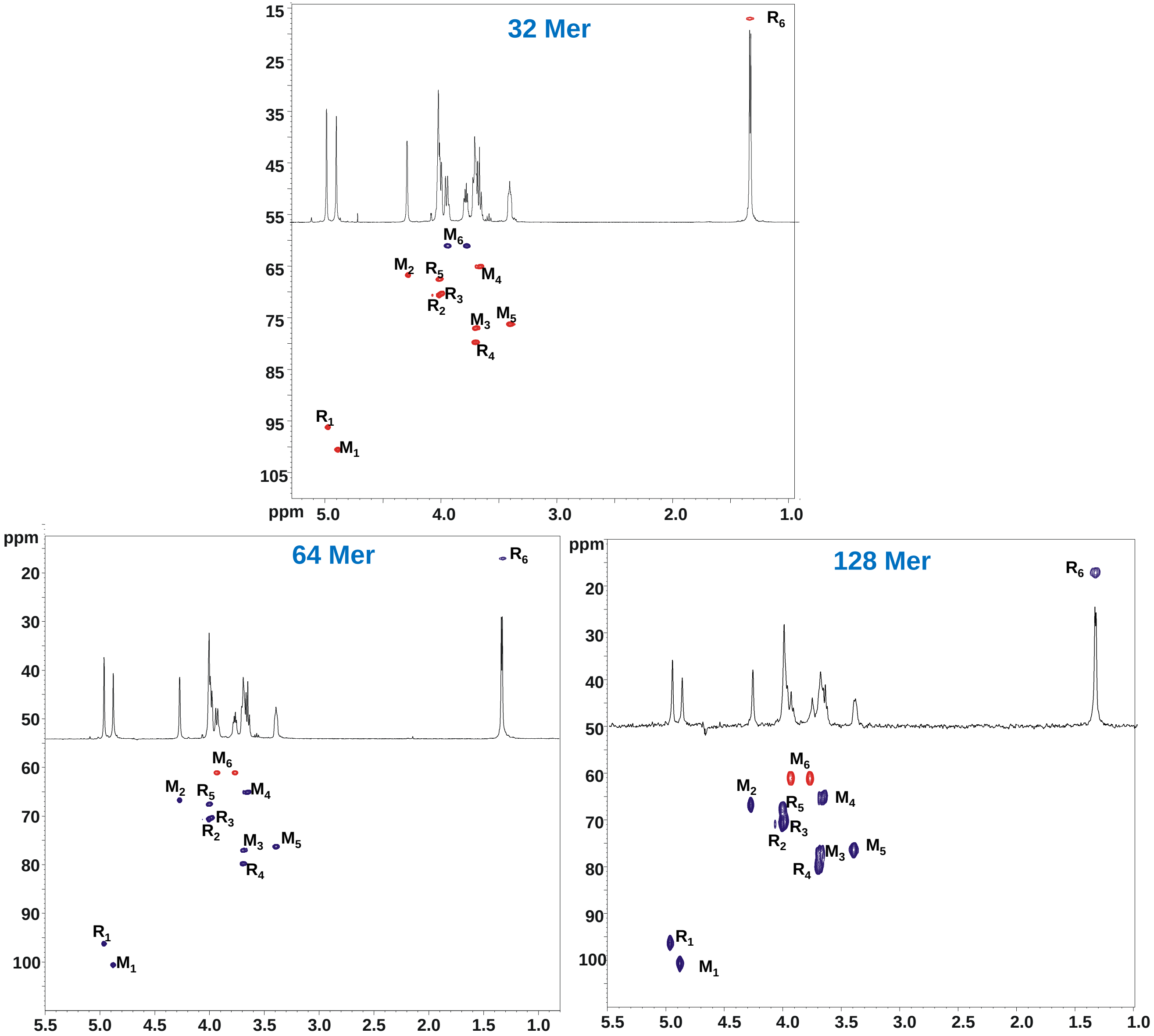

Supplementary Figure 7. 2D NMR spectra of 32-mer 5, 64-mer 6 and 128-mer 7.

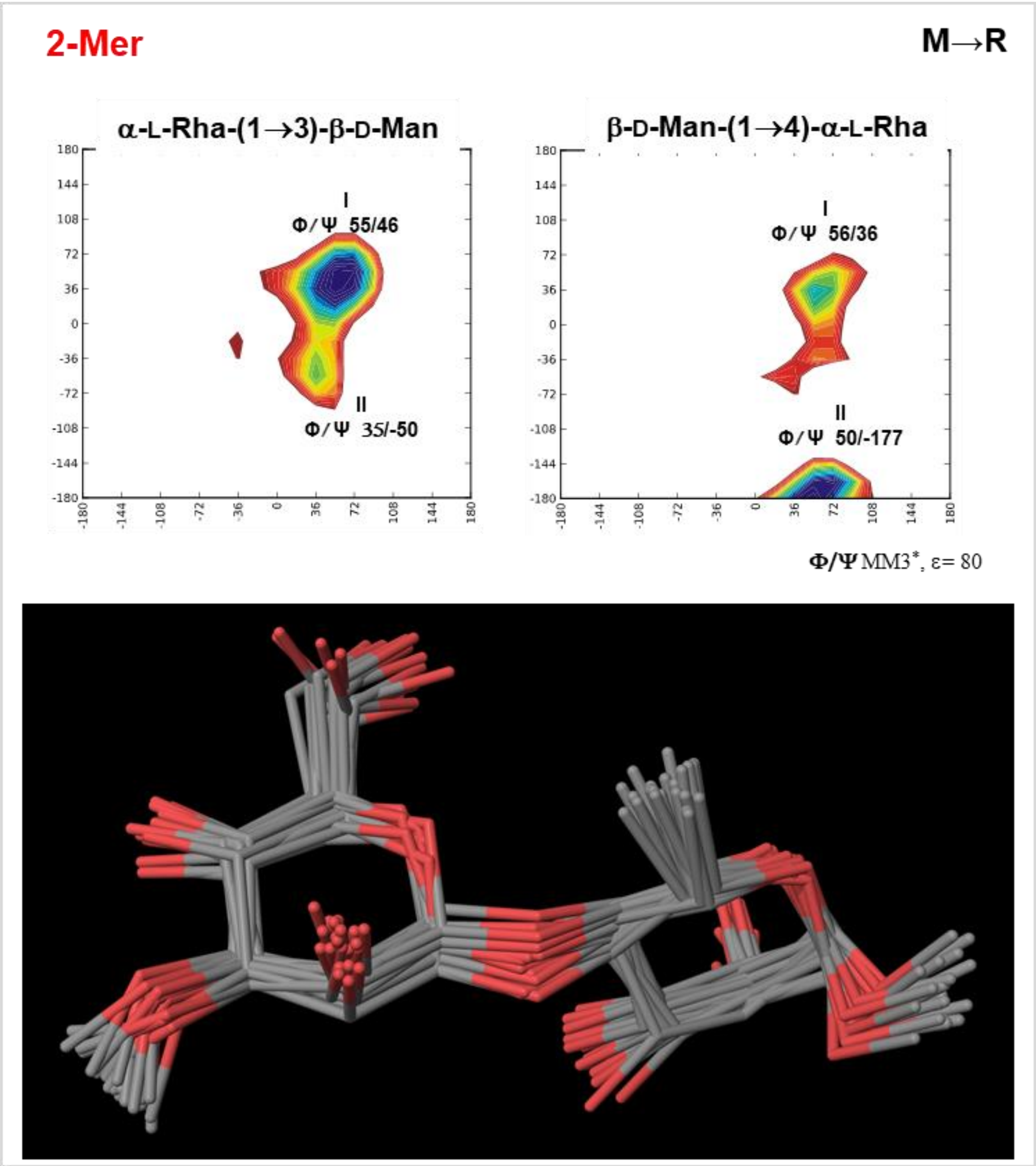

Supplementary Figure 8. The adiabatic energy maps for the glycosidic torsions φ (H1-C1-O-CX') and ψ (C1-O-CX'-HX') of 2-mer 1.

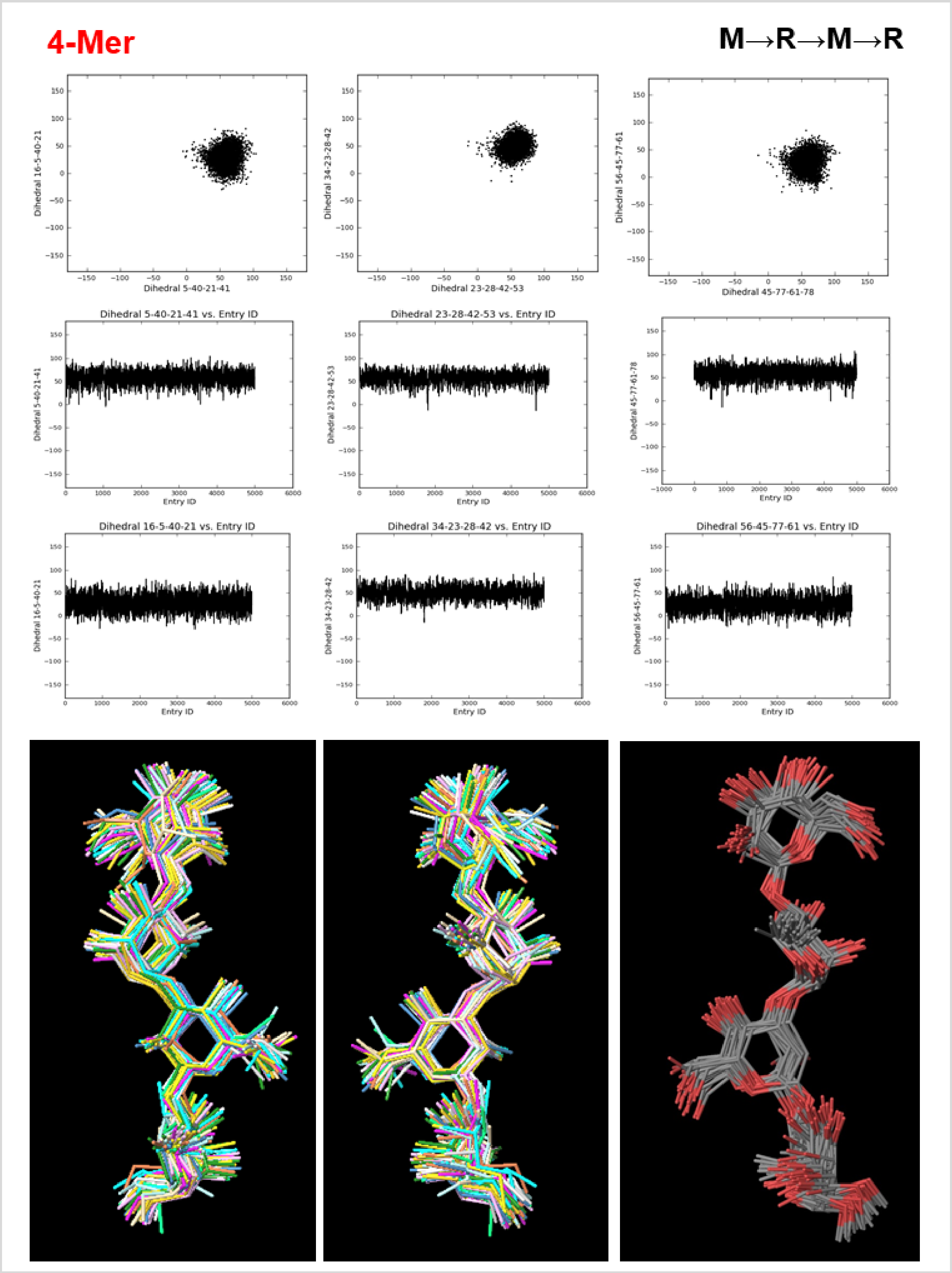

Supplementary Figure 9. Trajectories and Φ/Ψ scatter plots of the glycosidic linkages of 4-mer 2.

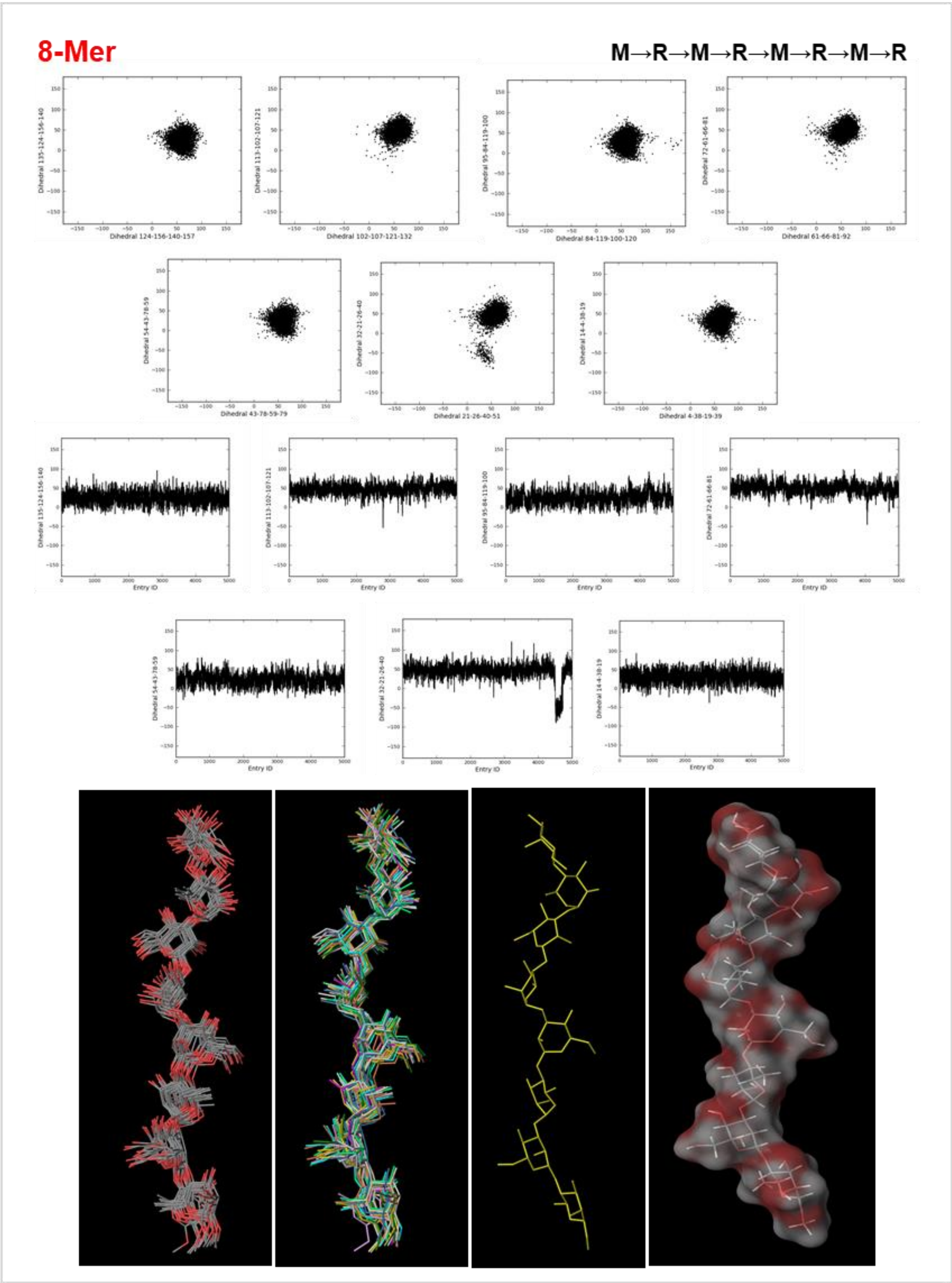

Supplementary Figure 10. Trajectories and Φ/Ψ scatter plots of the glycosidic linkages of 8-mer 3.

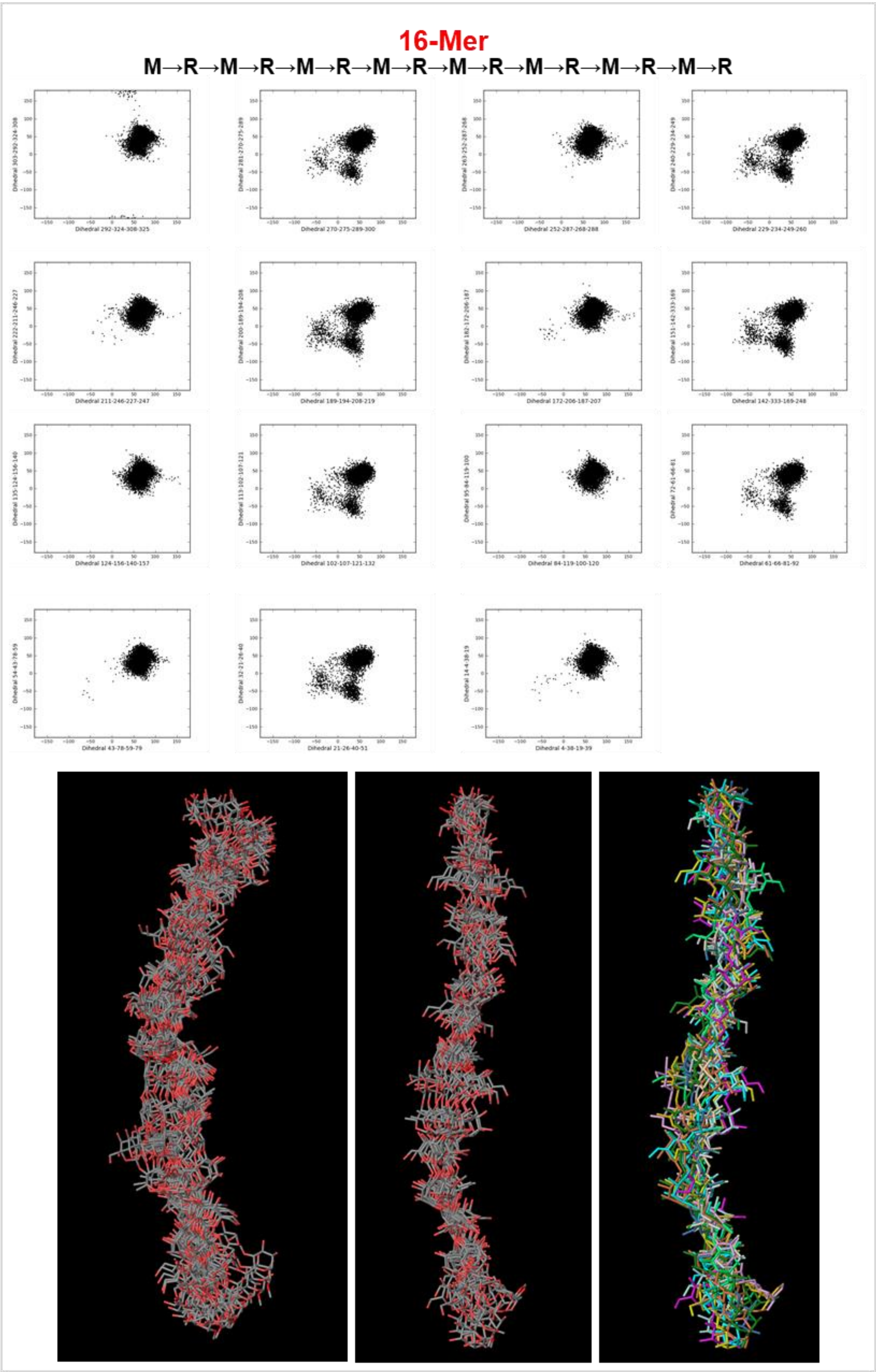

Supplementary Figure 11. Trajectories and Φ/Ψ scatter plots of the glycosidic linkages of 16-mer 4.

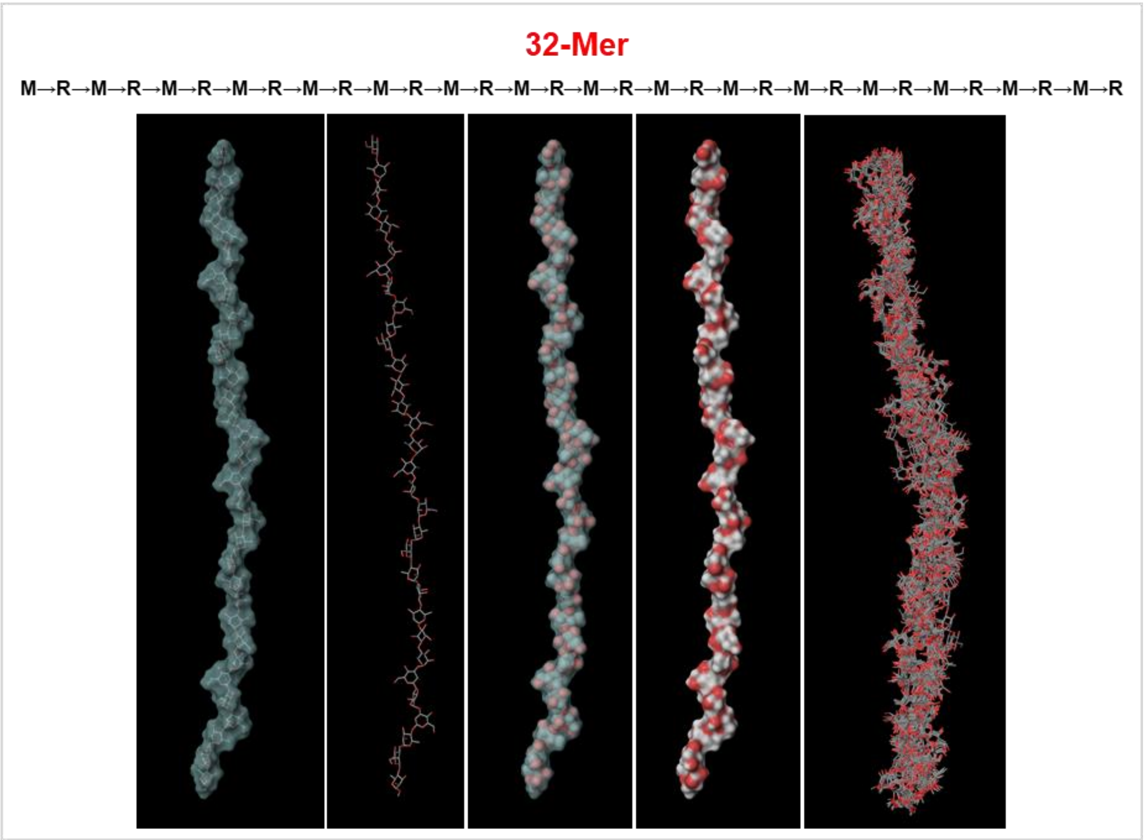

Supplementary Figure 12. Trajectories and Φ/Ψ scatter plots of the glycosidic linkages of 32-mer 5.

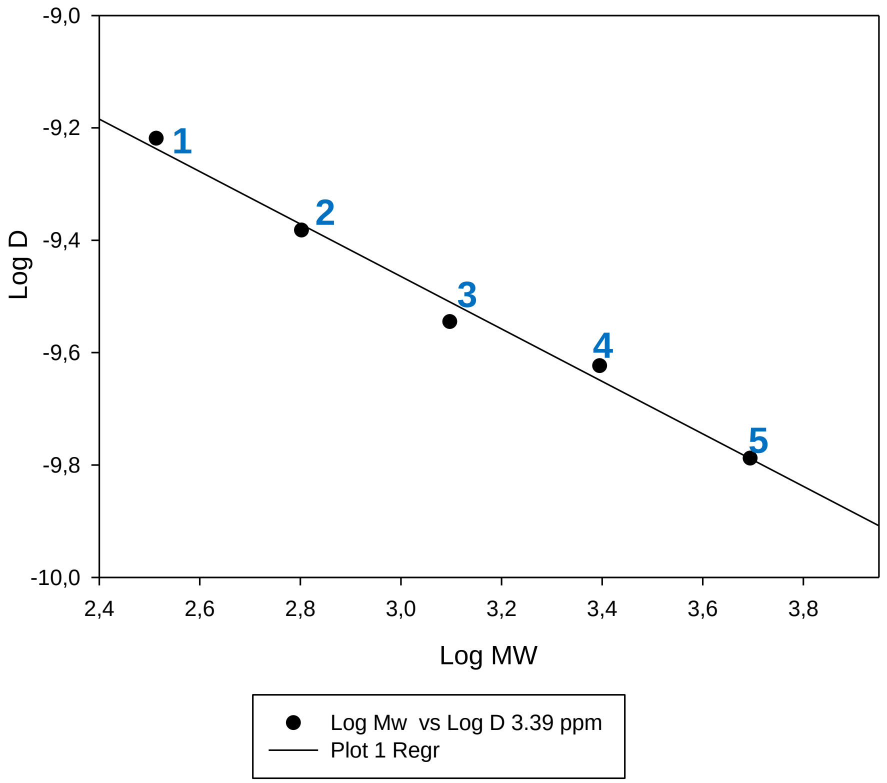

Supplementary Figure 13. Double-logarithmic plot of D against Mw for 2-, 4-, 8-, 16-, 32-mer (glycan 1-5, 100 mL at 308 K in D_2O) as determined by DOSY experiments NMR experiments. The double-logarithmic plot of D against Mw (see also Figure 3c) provided a calibration curve described by the least-squares fitted linear equation:

Log D = -8,064 - (0,467 * Log Mw 3.39 ppm) (1)

By (1) the molecular weight of **6** and **7** was estimated as follows:

Glycan **6** (64-mer): Diffusion coefficient $1.185*10^{-10}$ (Log D -9.93) from (1)

$\Rightarrow$ Log MW 3.995 $\Rightarrow$ Mw 9885 $\Rightarrow$ 64.19 mer

Glycan **7** (128-mer): Diffusion coefficient $8 *10^{-11}$ (Log D -10.09) from (1)

$\Rightarrow$ Log Mw 4.338 $\Rightarrow$ Mw 22855 $\Rightarrow$ 142 mer

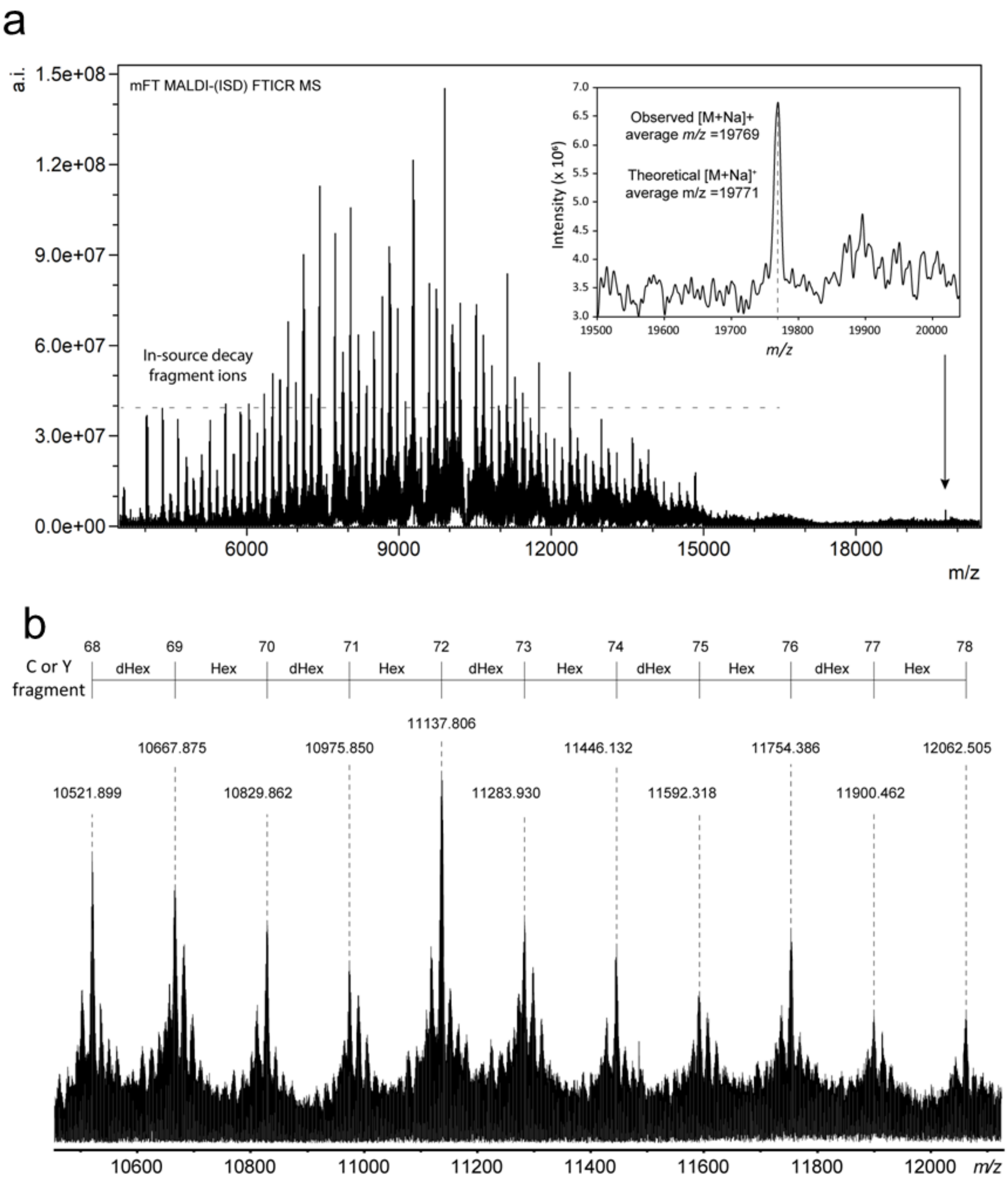

Supplementary Figure 14. **a** MALDI FT-ICR MS spectrum of 128-mer **7**. The intact polysaccharide was detected at a S/N of 9.5 while intense in-source decay (ISD) fragments dominate the spectrum. **b** Enlargement of MALDI FT-ICR MS spectrum of 128-mer **7**. Here, isotopically resolved ISD fragment ions are depicted. The m/z-differences between adjacent fragment ions (either 162 or 146 Th) were in agreement with the masses of hexose (Hex) and deoxyhexose (dHex) residues.

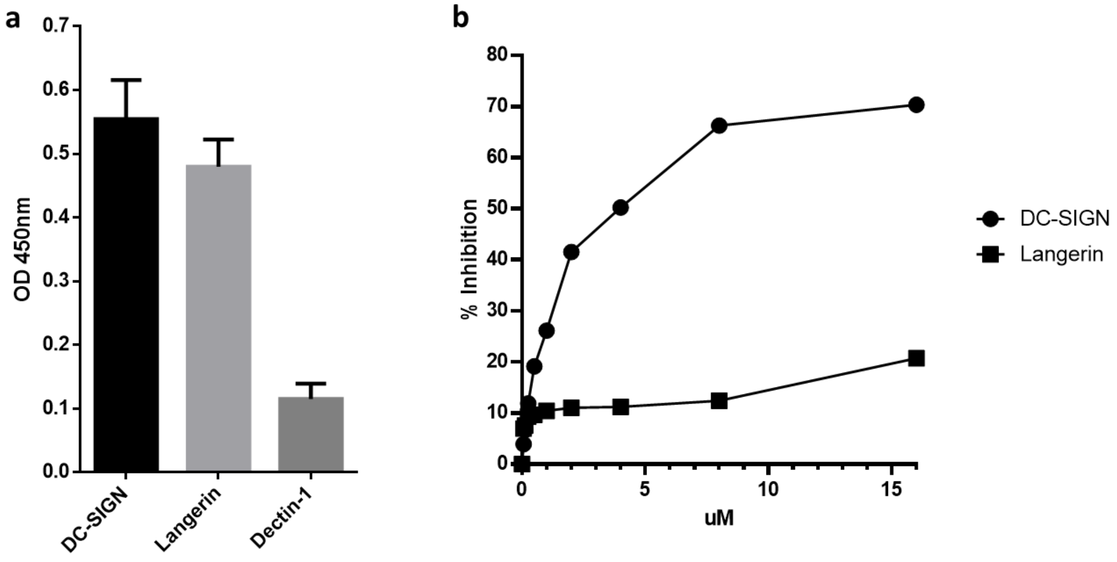

Supplementary Figure 15. Quali-quantitative C-type lectins binding to LPS from *B. vulgatus* detected by ELISA. a Direct ELISA. ELISA wells were coated with LPS from *B. vulgatus* and the qualitative binding to DC-SIGN, Langerin and Dectin-1 was detected by ELISA. The experiment has been performed three times in duplicate with similar results, and data were normalized over signal from BSA-coated wells. Error bars indicate standard deviations. OD: Optical density. **b** Competition ELISA. ELISA plates were coated with purified LPS from *B. vulgatus*, followed by addition of a human Fc chimera of DC-SIGN or Langerin pre-incubated with different concentrations of *B. vulgatus* LPS. The detection of the interaction was evaluated with an anti-human IgG-HRP (horseradish peroxidase) conjugate followed by substrate addition. The LPS from *B. vulgatus* showed a better affinity for DC-SIGN compared with Langerin. Data from one representative experiment (performed in duplicate) of three independent experiments, showing similar results, are shown. Significant differences between DC-SIGN or Langerin binding compared to Dectin-1 binding were calculated showing $p<0.01$. Experimental data were analyzed by a grouped multiple t-test using GraphPad Prism 6.0, followed by using the Sidak-Bonferroni method, with a statistical significance level of alpha=0.05. Error bars represent standard deviation of one representative experiment performed in duplicate.

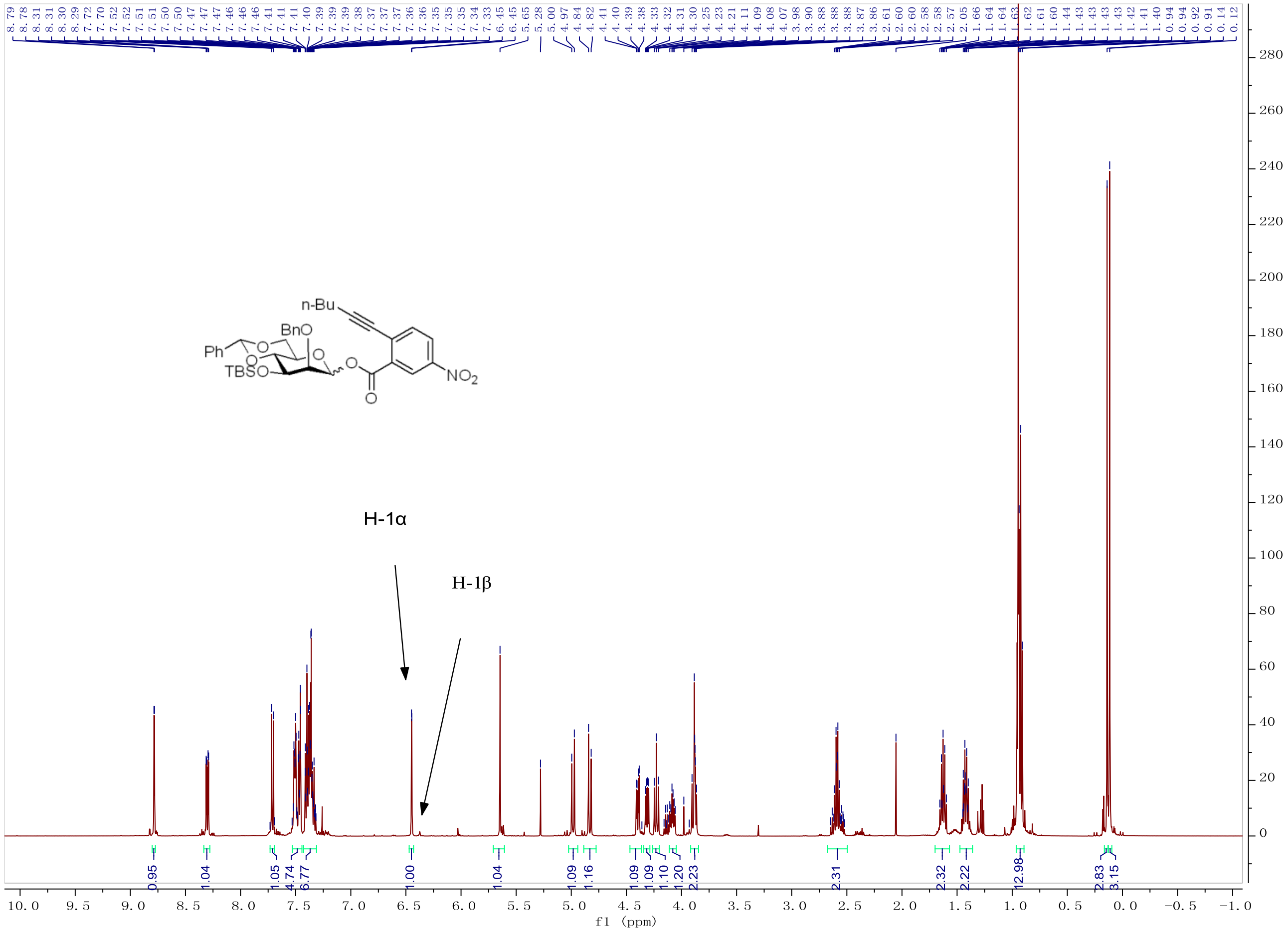

Supplementary Figure 16. ^{1}H-NMR spectrum of compound S4 ($CDCl_3$, 25 °C)

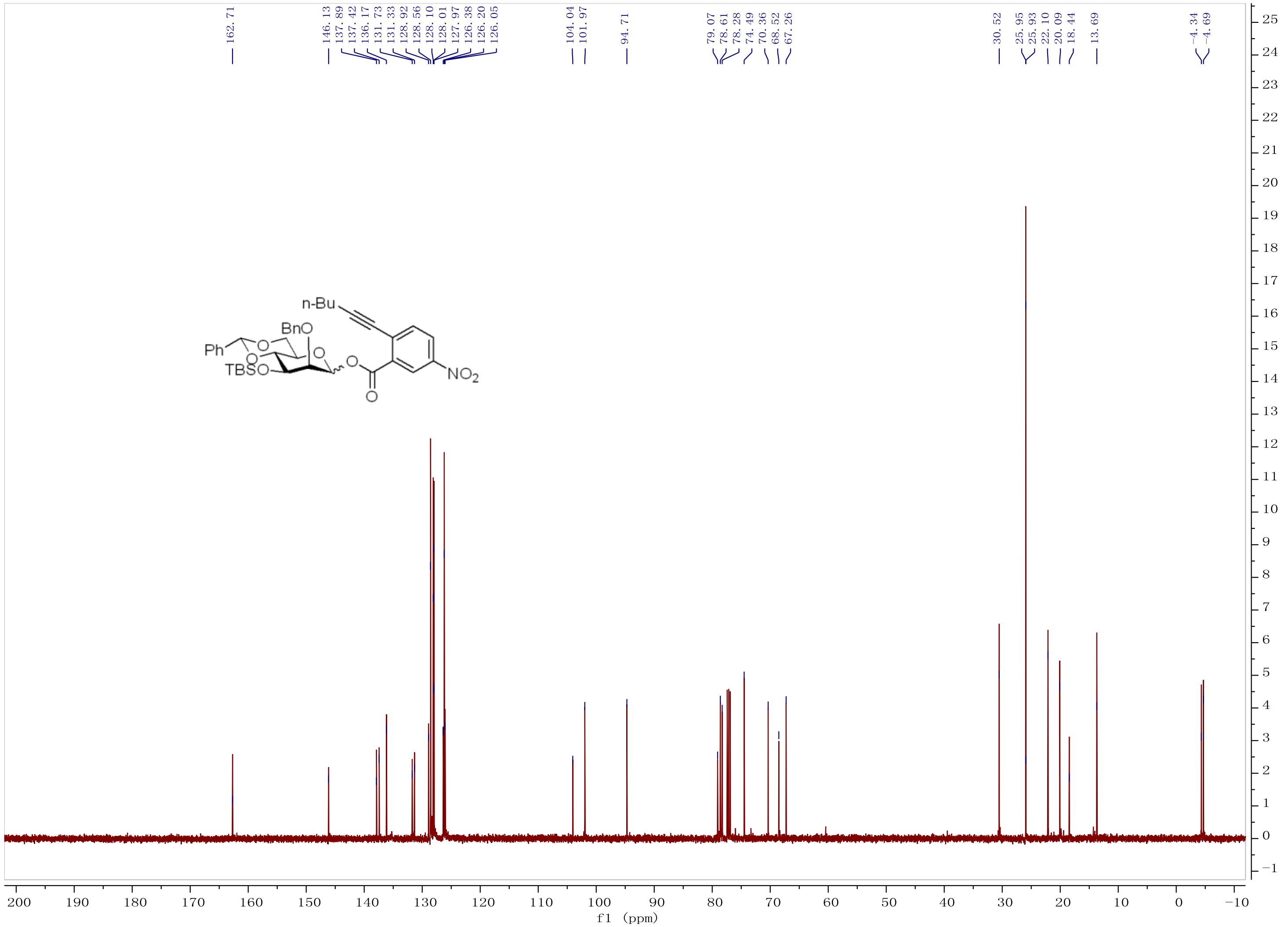

Supplementary Figure 17. ^{13}C-NMR spectrum of compound S4 ($CDCl_3$, 25 ^{o}C)

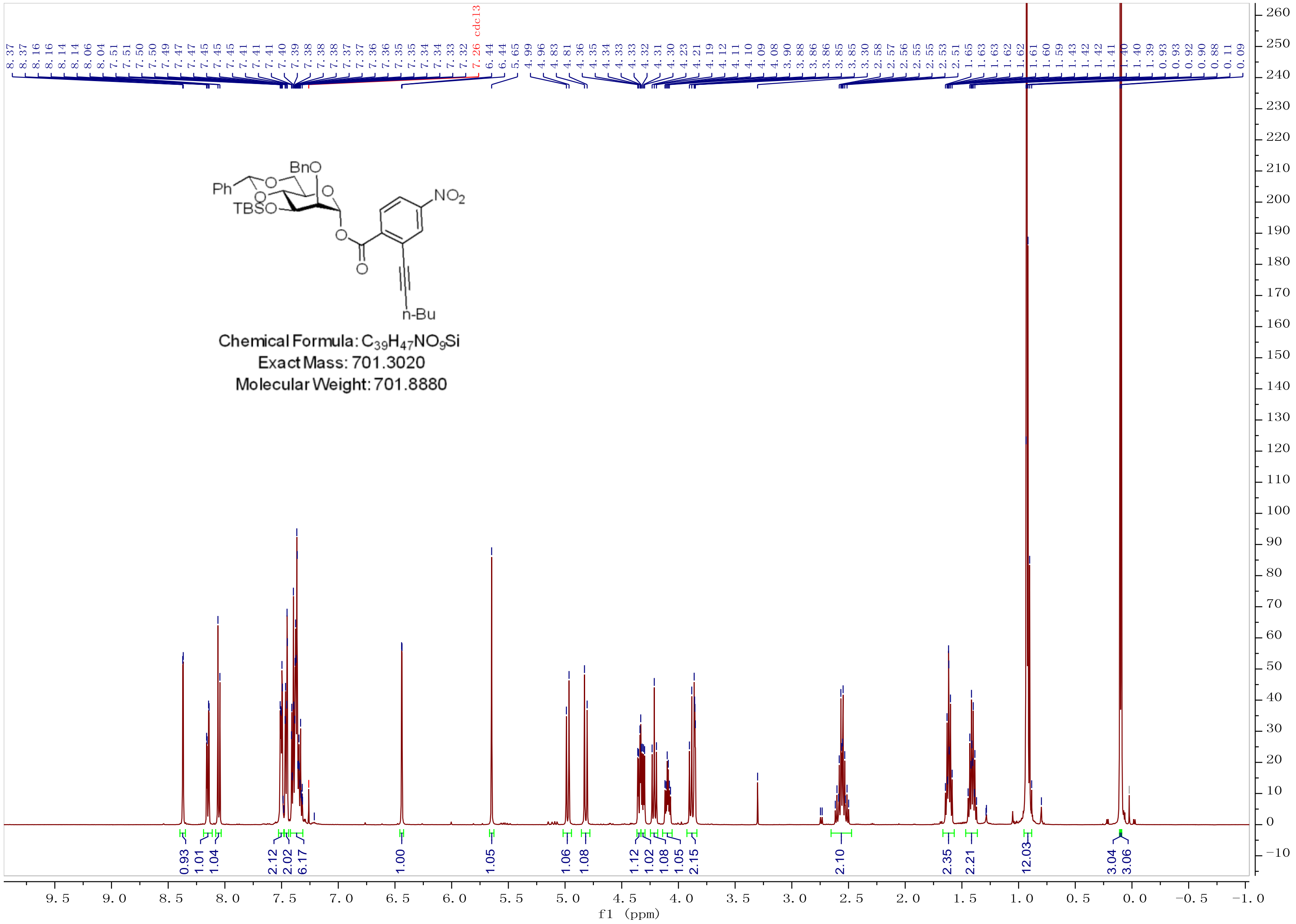

Supplementary Figure 18. ^{1}**H-NMR spectrum of compound S5 ($CDCl_3$, 25 °C)**

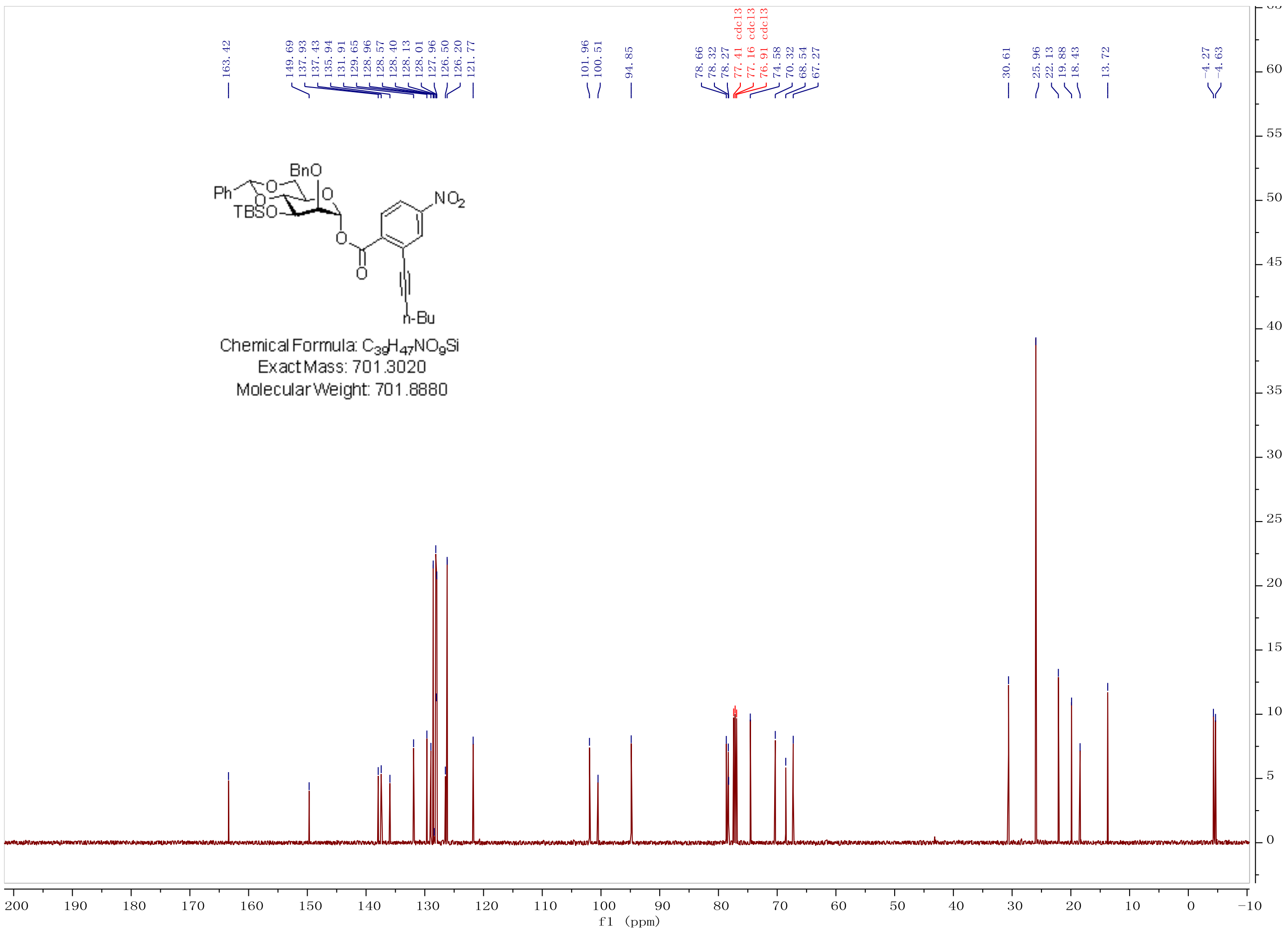

Supplementary Figure 19. **^{13}C-NMR spectrum of compound S5 ($CDCl_3$, 25 °C)**

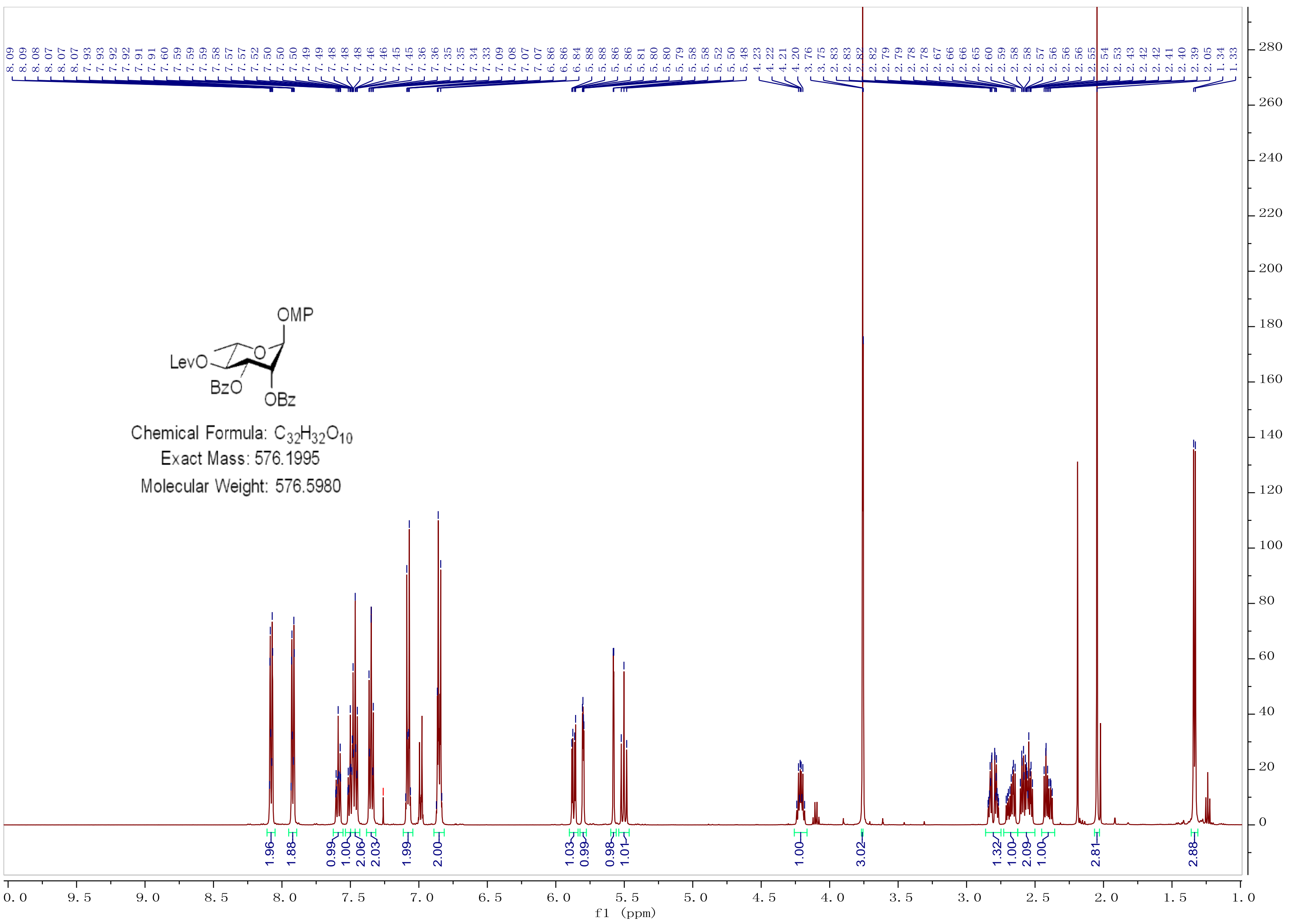

Supplementary Figure 20. ^{1}H-NMR spectrum of compound S8 ($CDCl_3$, 25 °C)

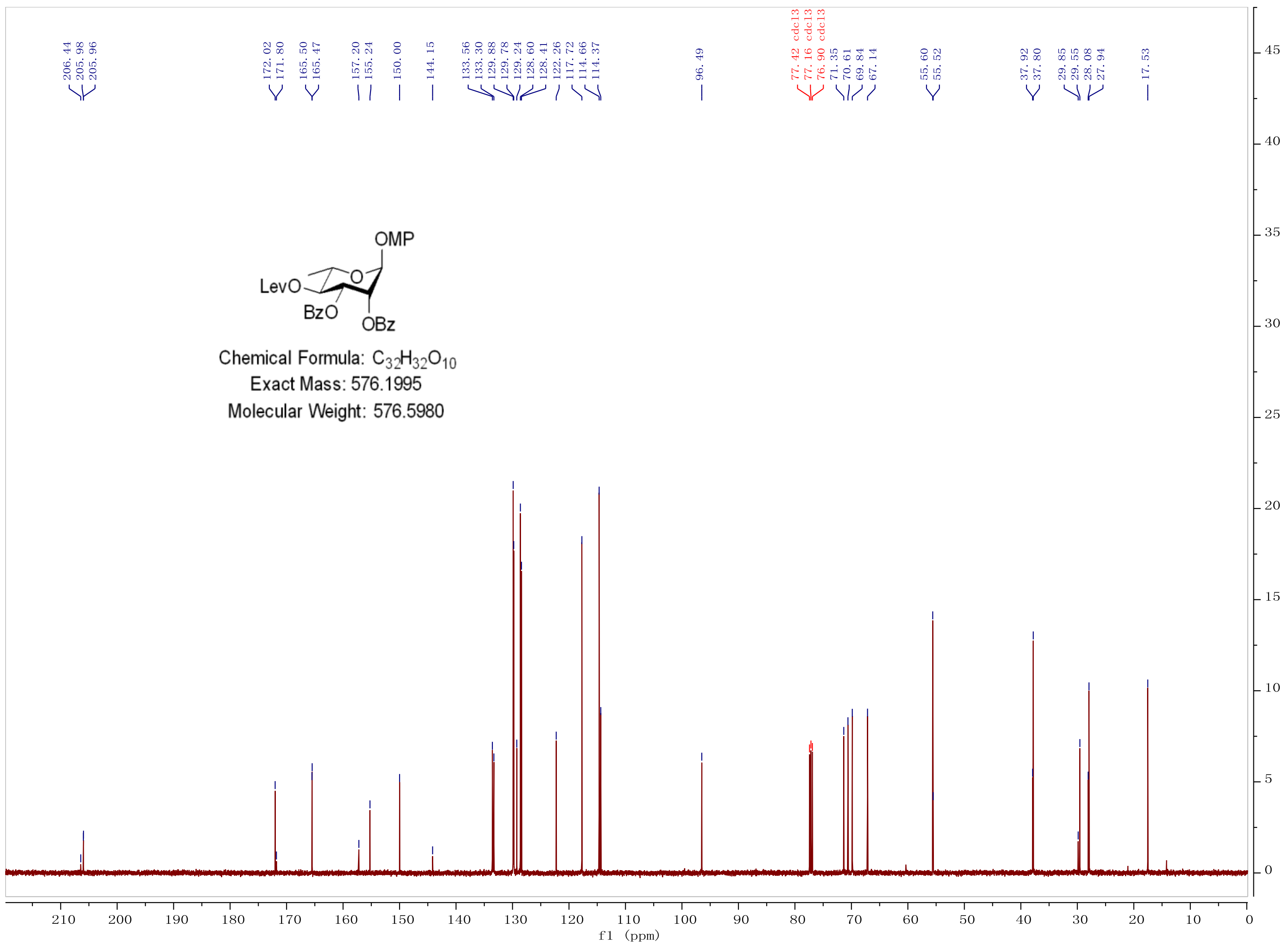

Supplementary Figure 21. ^{13}**C-NMR spectrum of compound S8 (CDCl$_3$, 25 °C)**

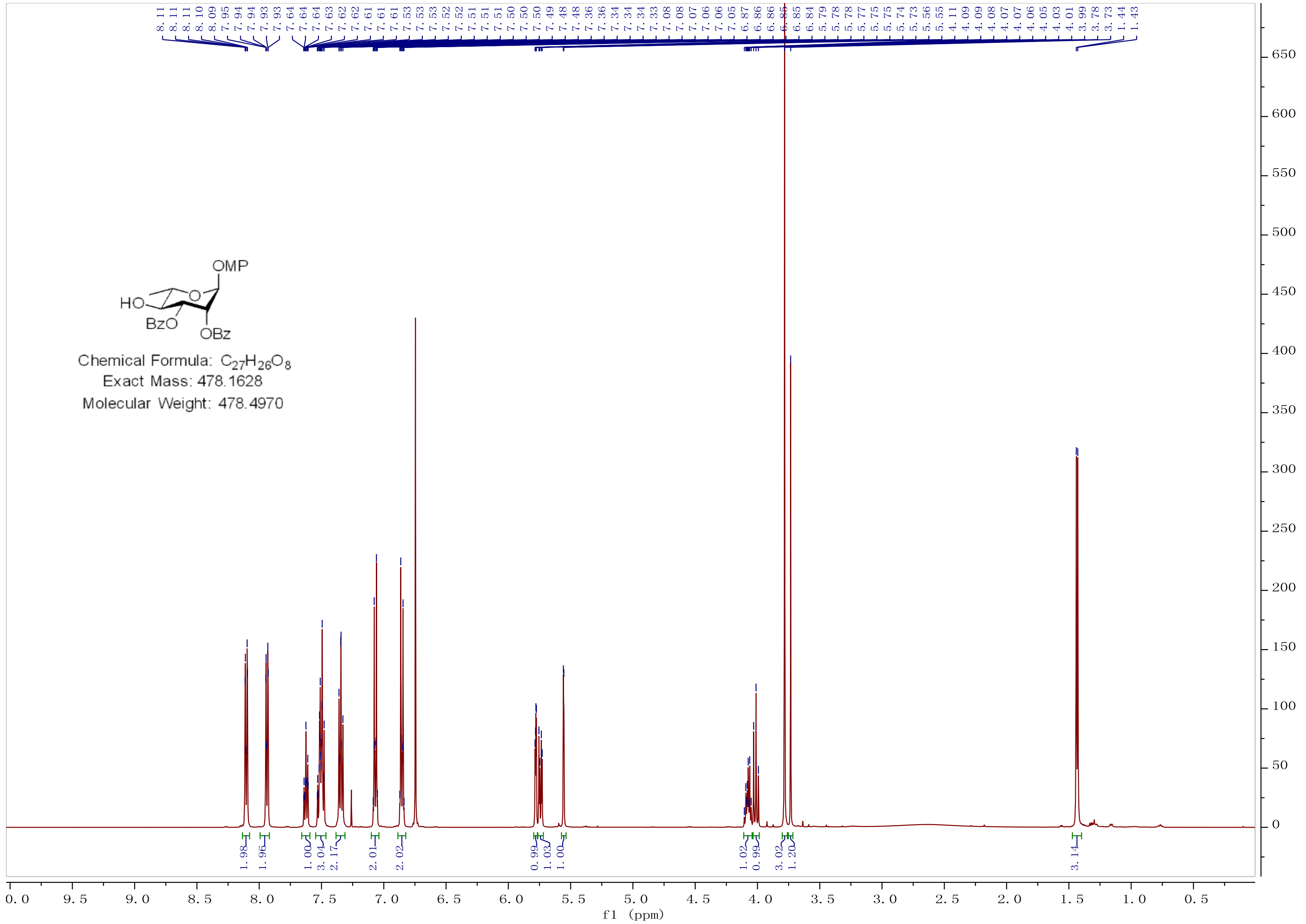

Supplementary Figure 22. ^{1}H-NMR spectrum of compound S6 ($CDCl_3$, 25 °C)

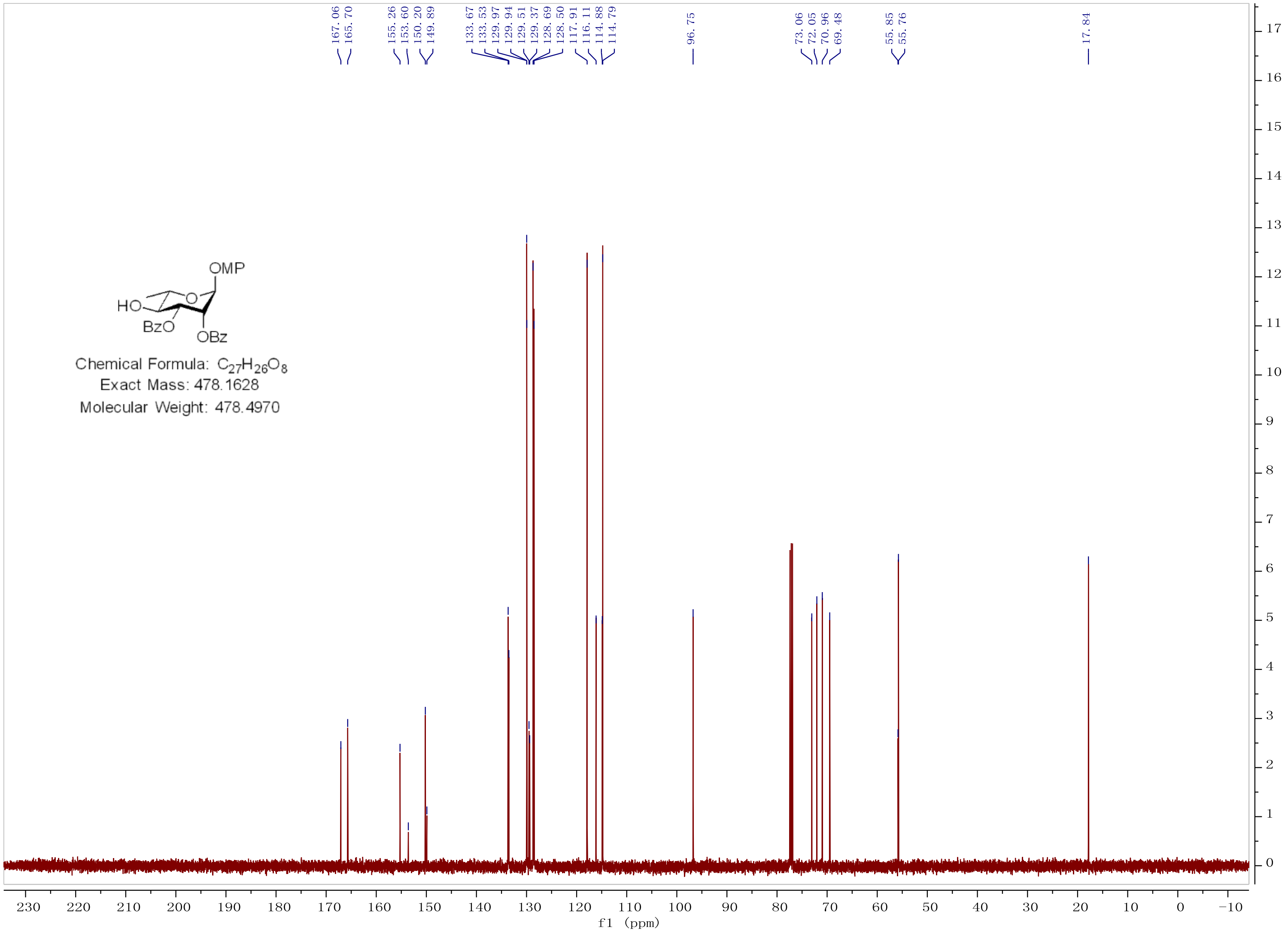

Supplementary Figure 23. ^{13}C-NMR spectrum of compound S6 ($CDCl_3$, 25 °C)

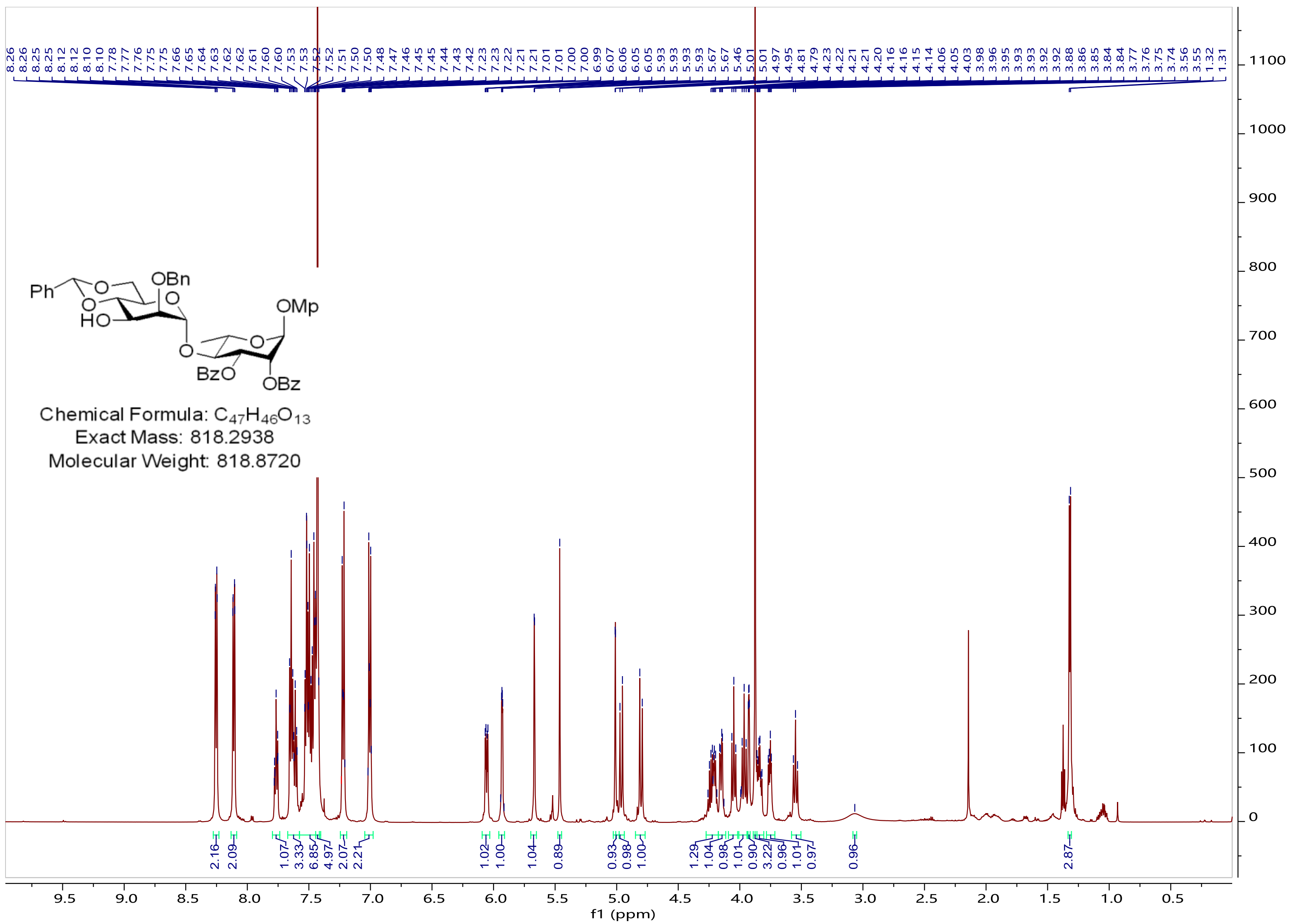

Supplementary Figure 24. ^{1}**H-NMR spectrum of compound S9 ($CDCl_3$, 25 °C)**

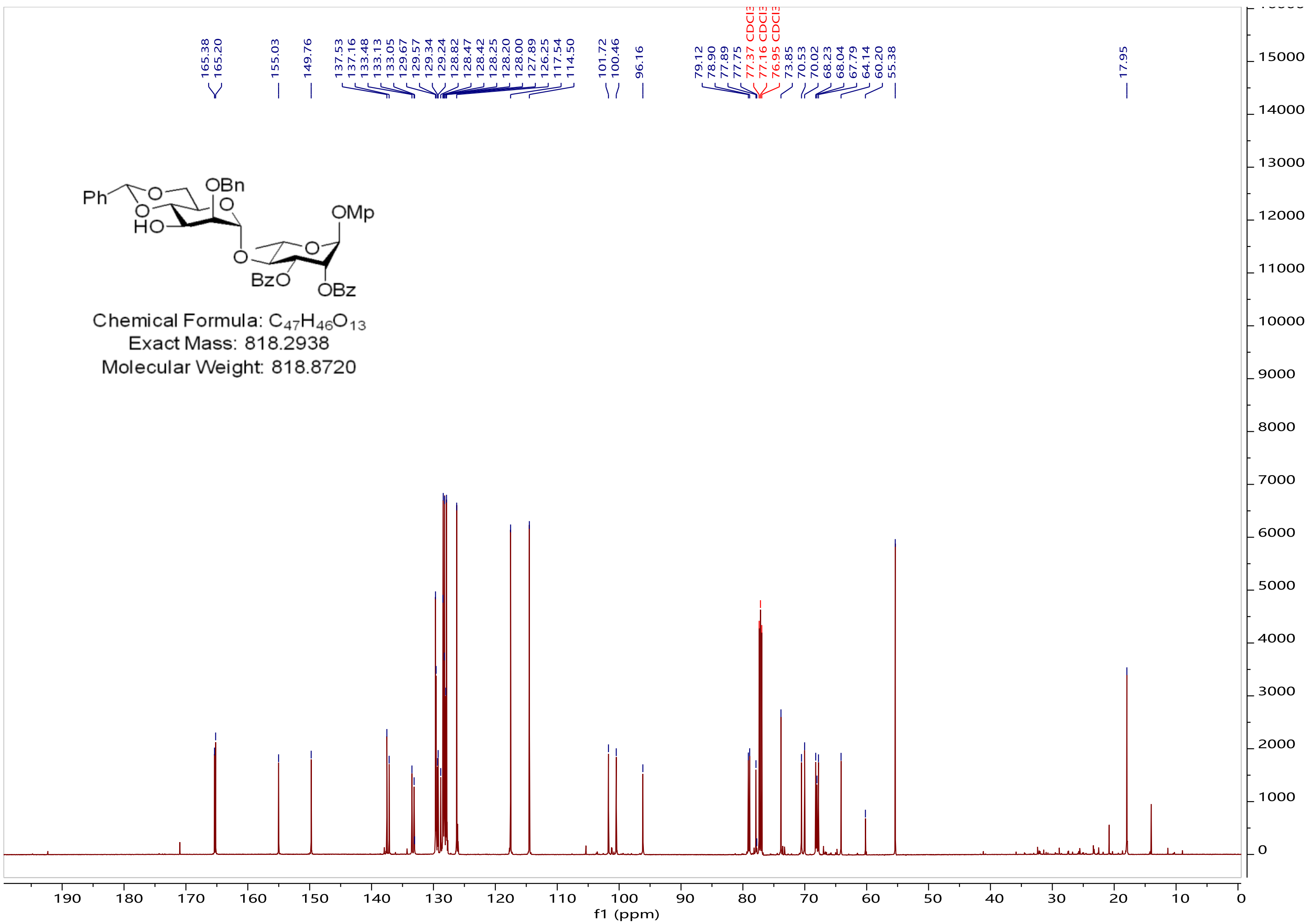

Supplementary Figure 25. ^{13}**C-NMR spectrum of compound S9 (CDCl$_3$, 25 °C)**

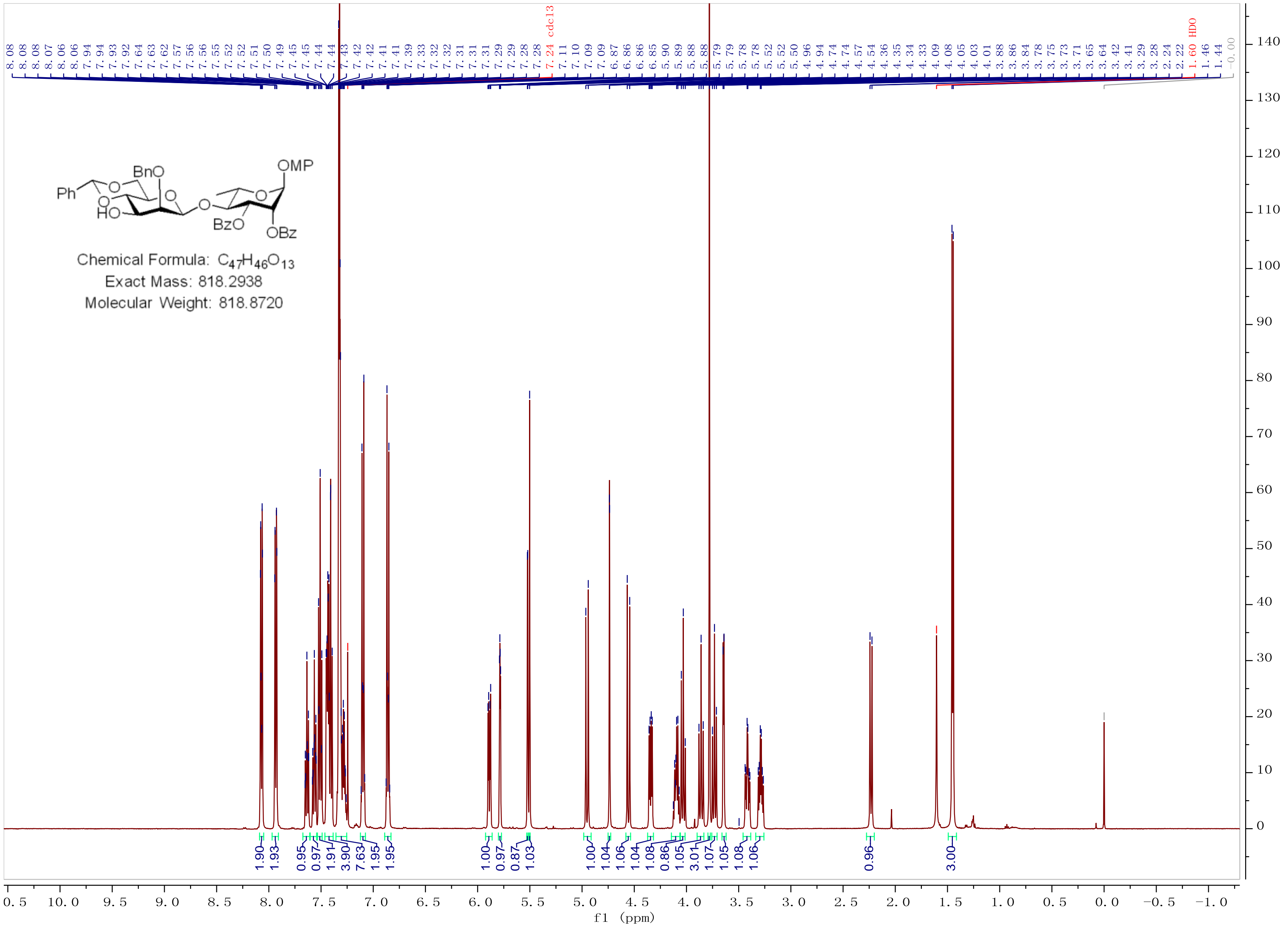

Supplementary Figure 26. ^{1}H-NMR spectrum of compound 8^A ($CDCl_3$, 25 ^oC)

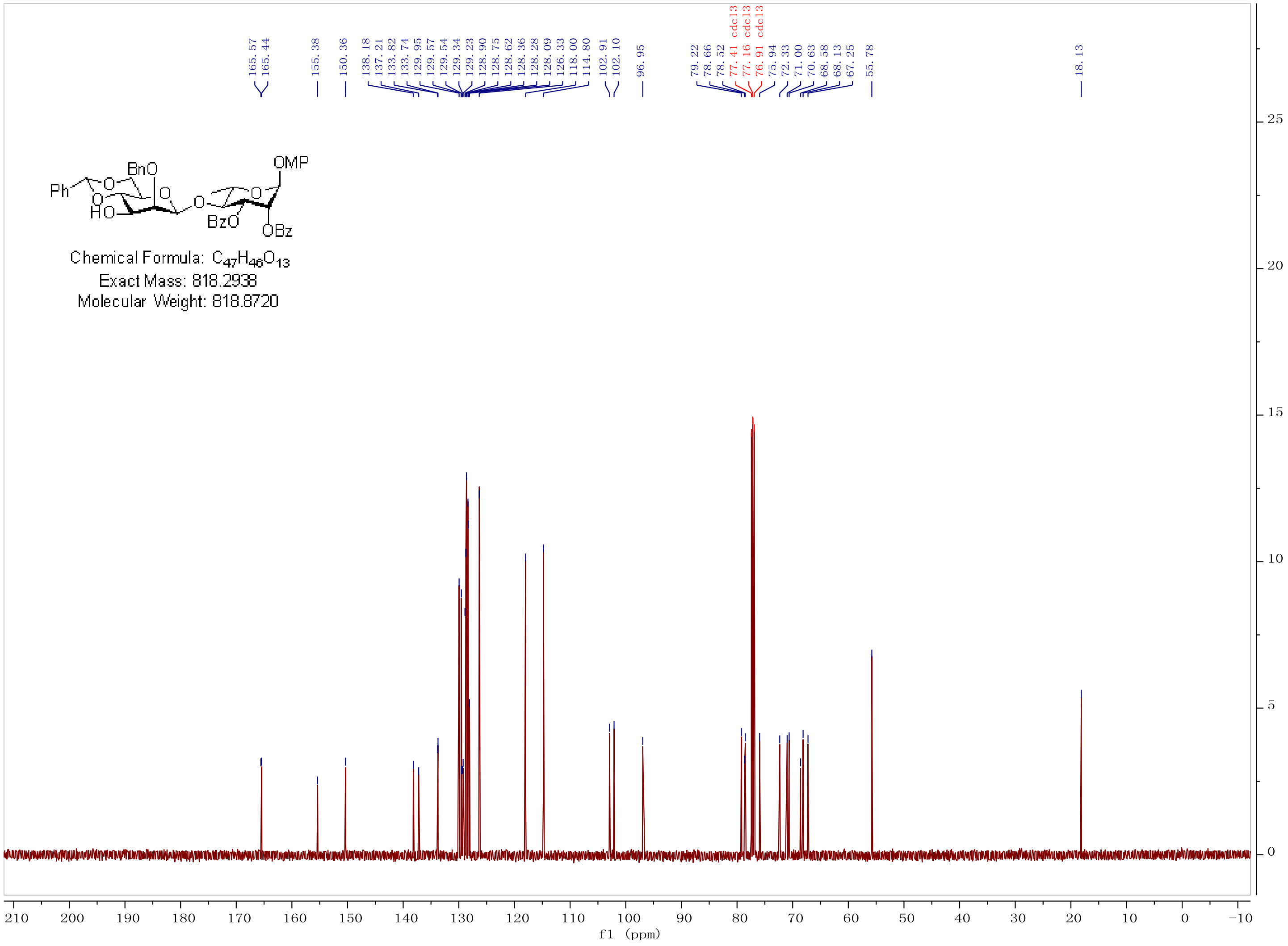

Supplementary Figure 27. ^{13}C-NMR spectrum of compound **8**^{A} ($CDCl_3$, 25 ^{o}C)

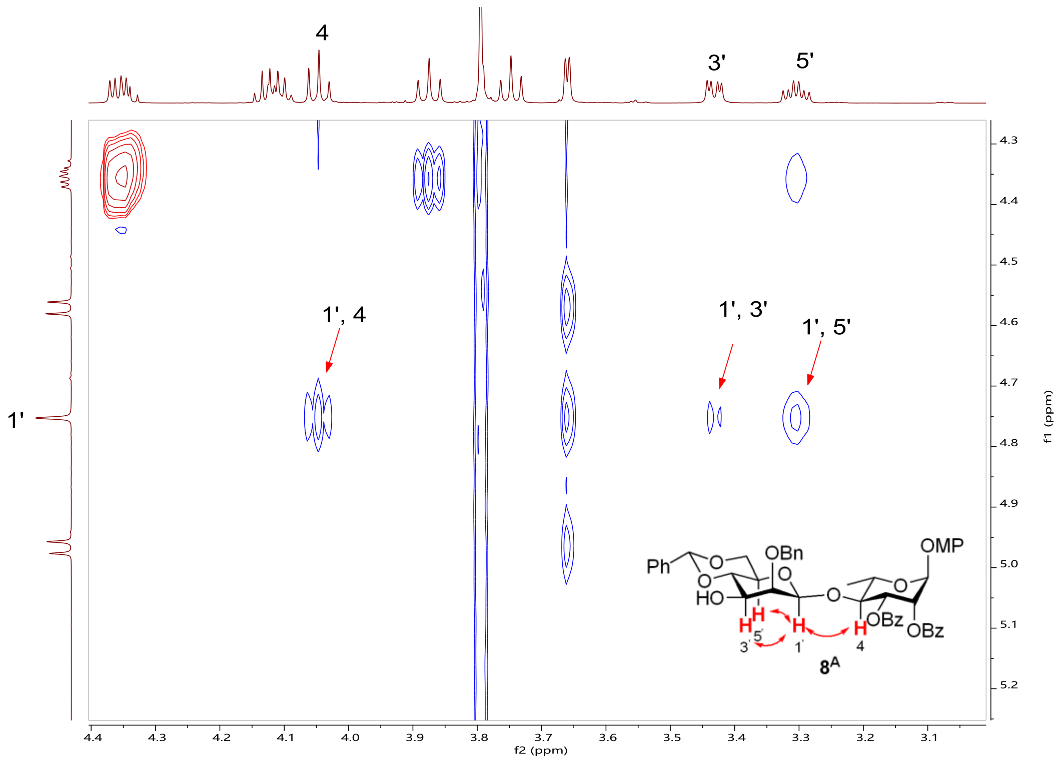

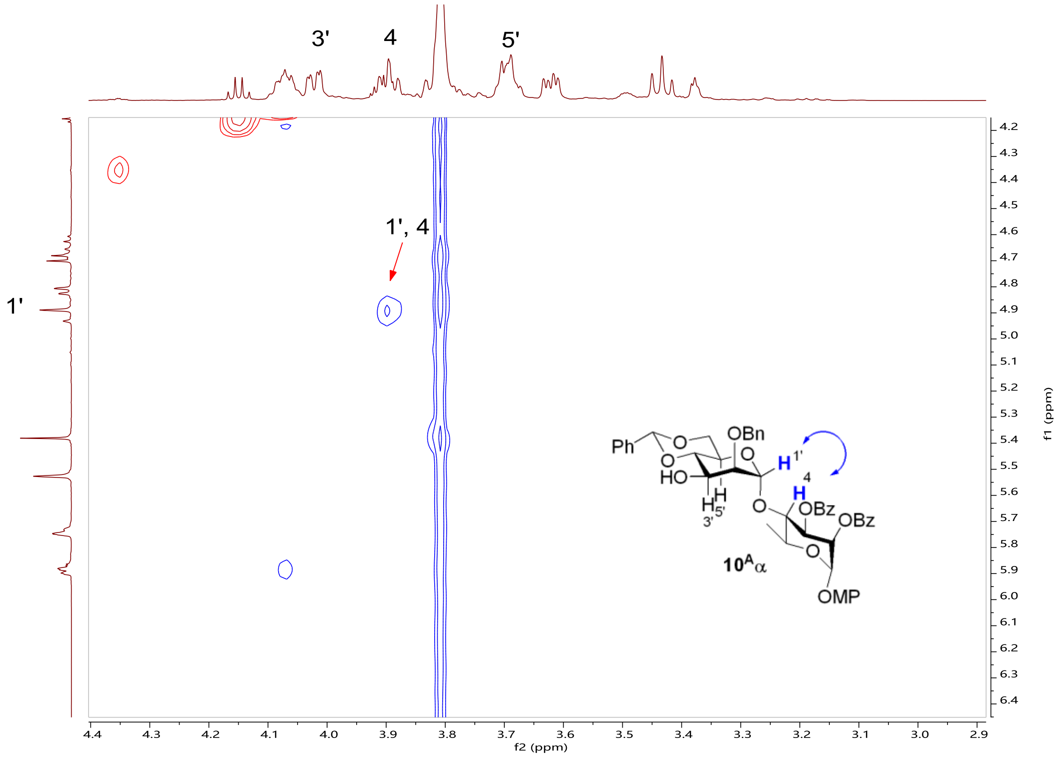

Supplementary Figure 28. NOESY spectrum of 8^{A} and S9

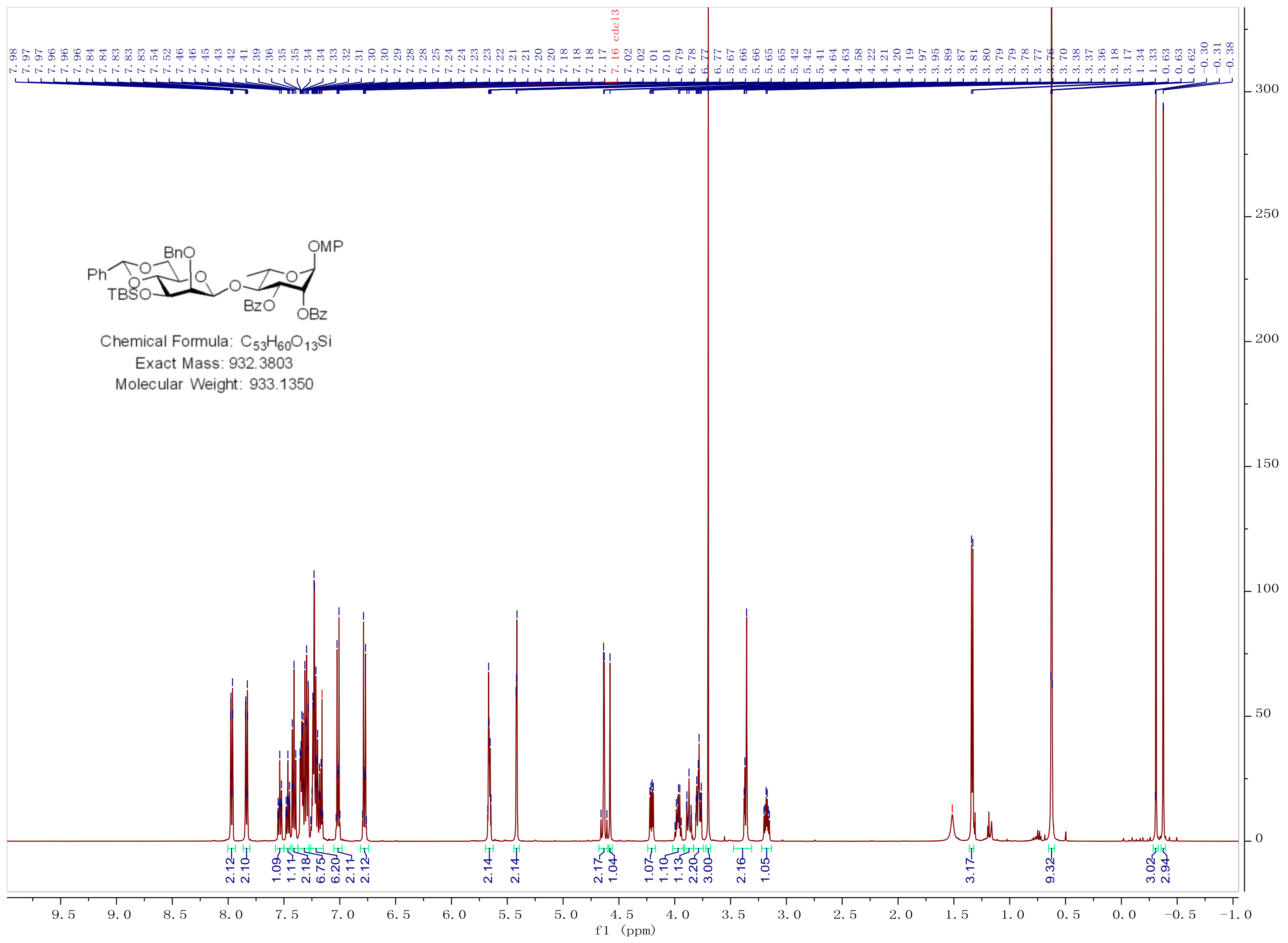

Supplementary Figure 29. **^{1}H-NMR spectrum of disaccharide 8 ($CDCl_3$, 25 ^oC)**

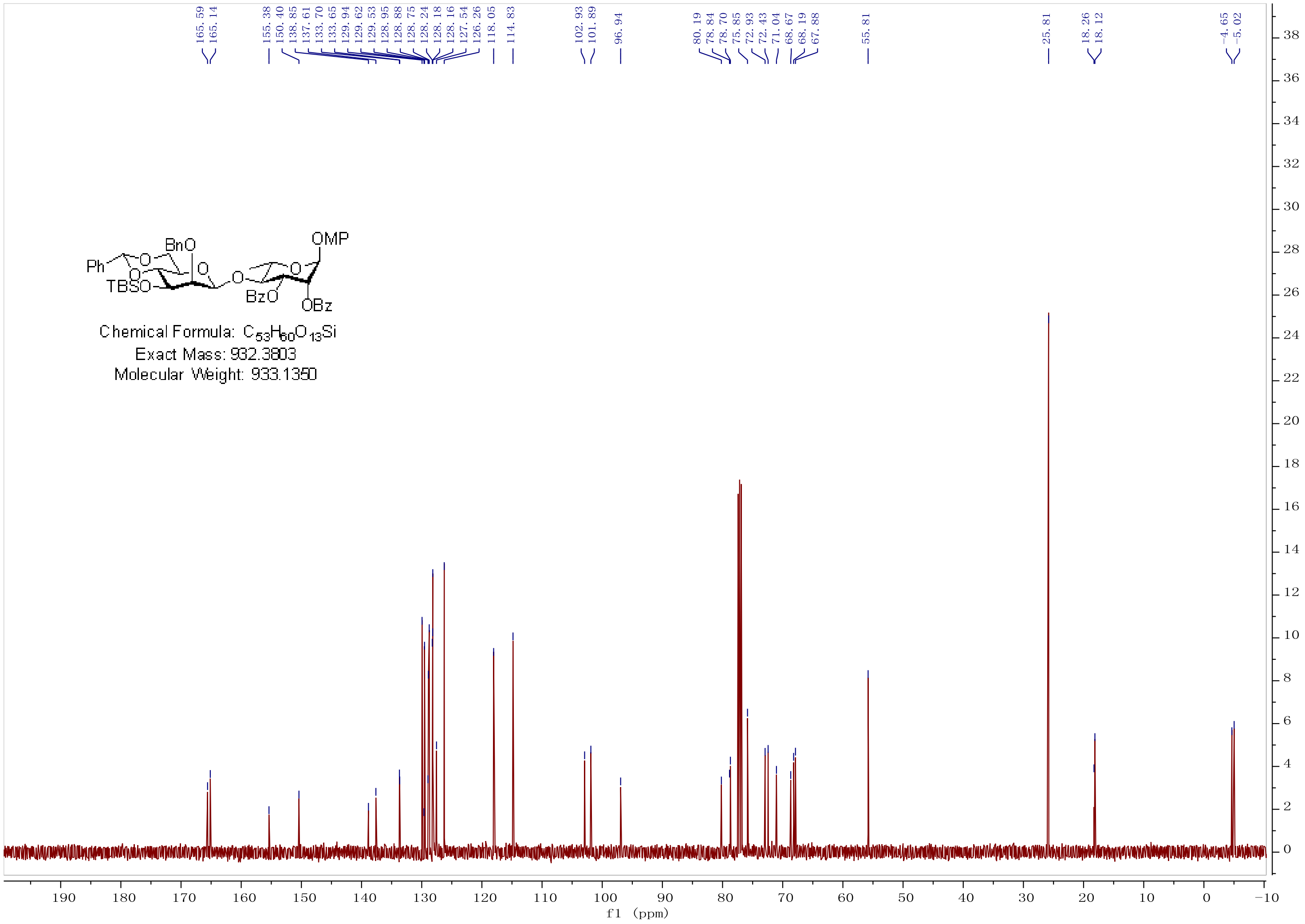

Supplementary Figure 30. ^{13}C-NMR spectrum of disaccharide 8 ($CDCl_3$, 25 ^{o}C)

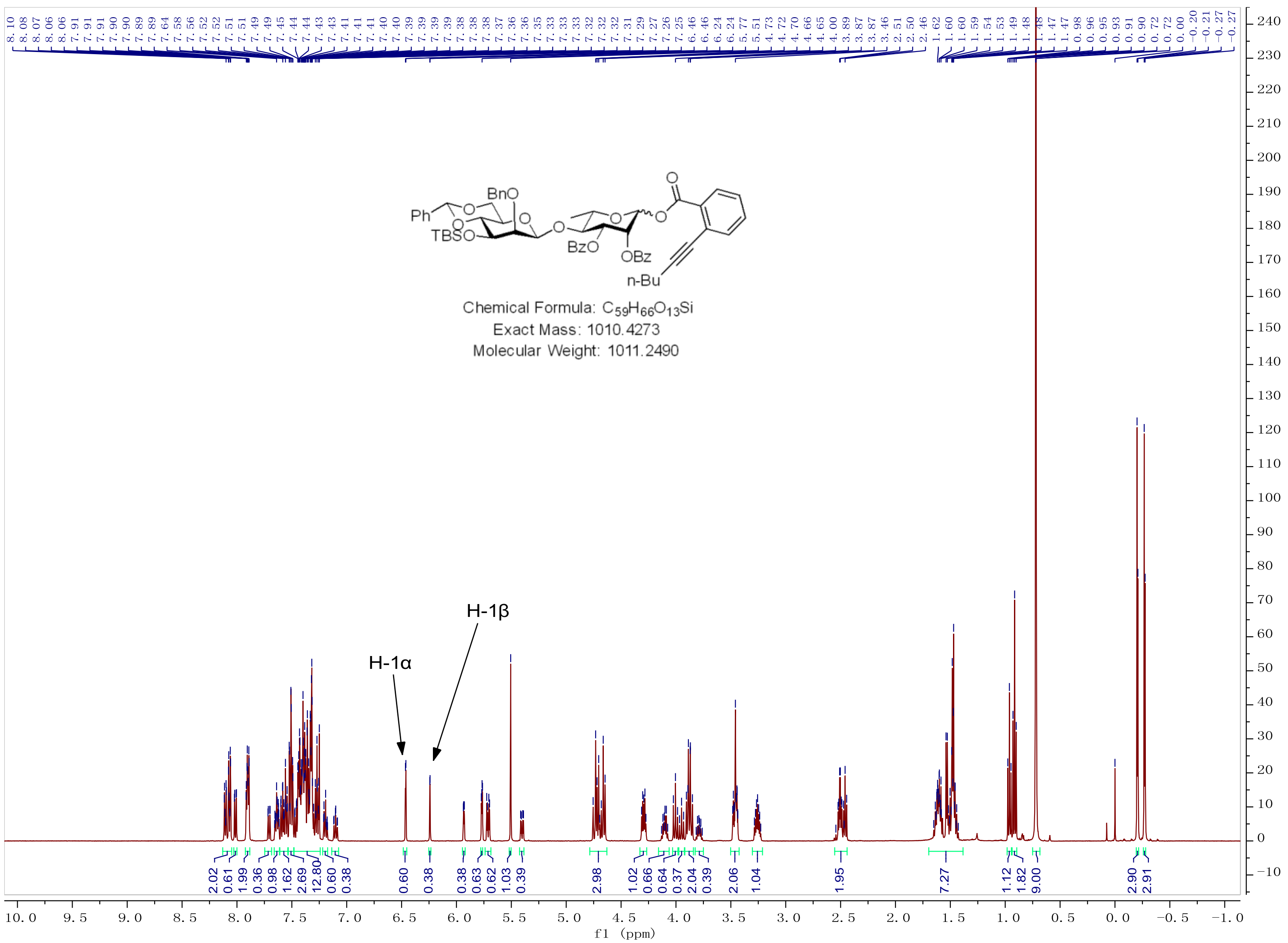

Supplementary Figure 31. ^{1}**H-NMR spectrum of compound 8**[D] **(CDCl**$_3$**, 25 °C)**

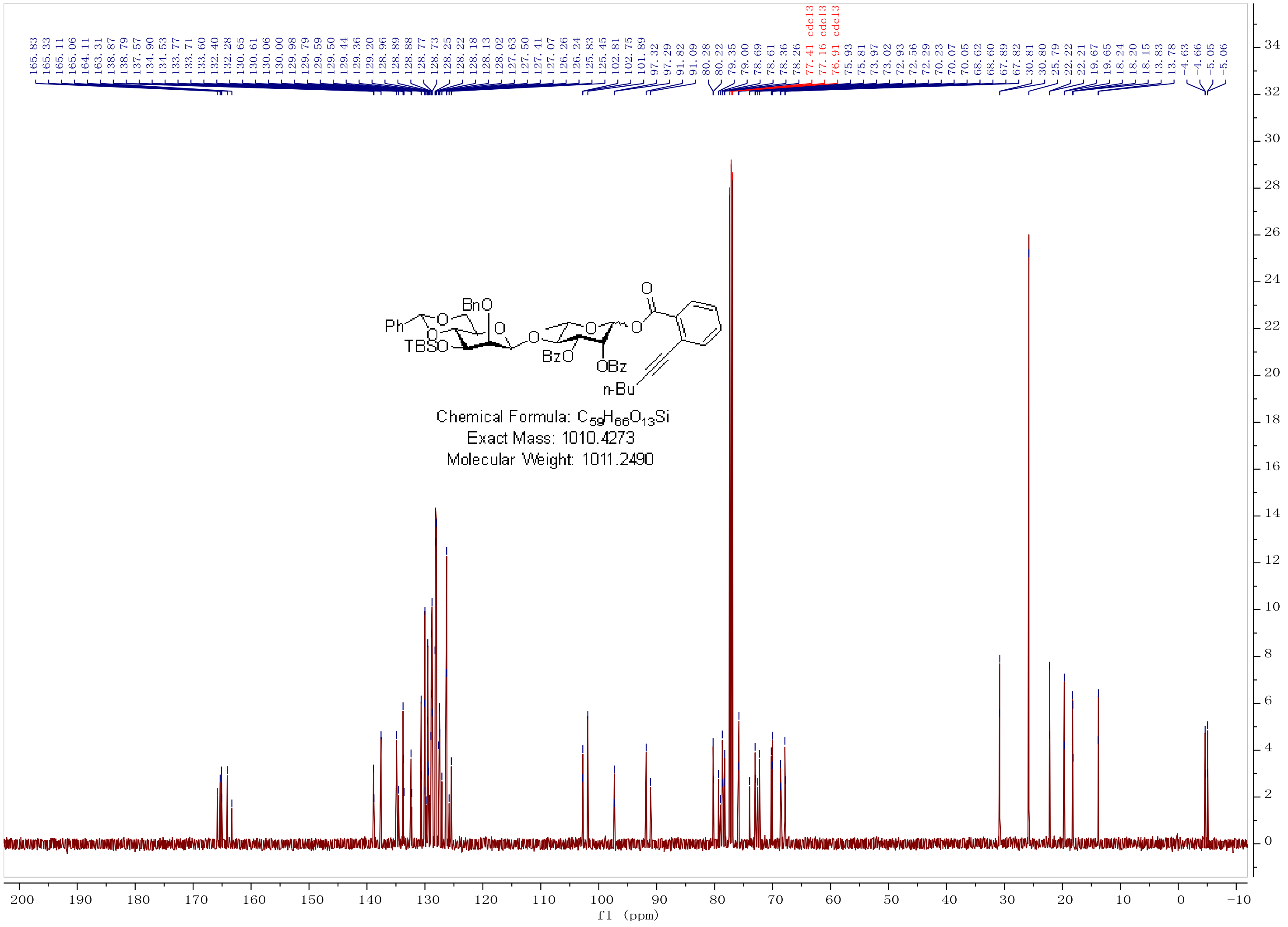

Supplementary Figure 32. ^{13}C-NMR spectrum of compound 8^{D} (CDCl$_3$, 25 °C)

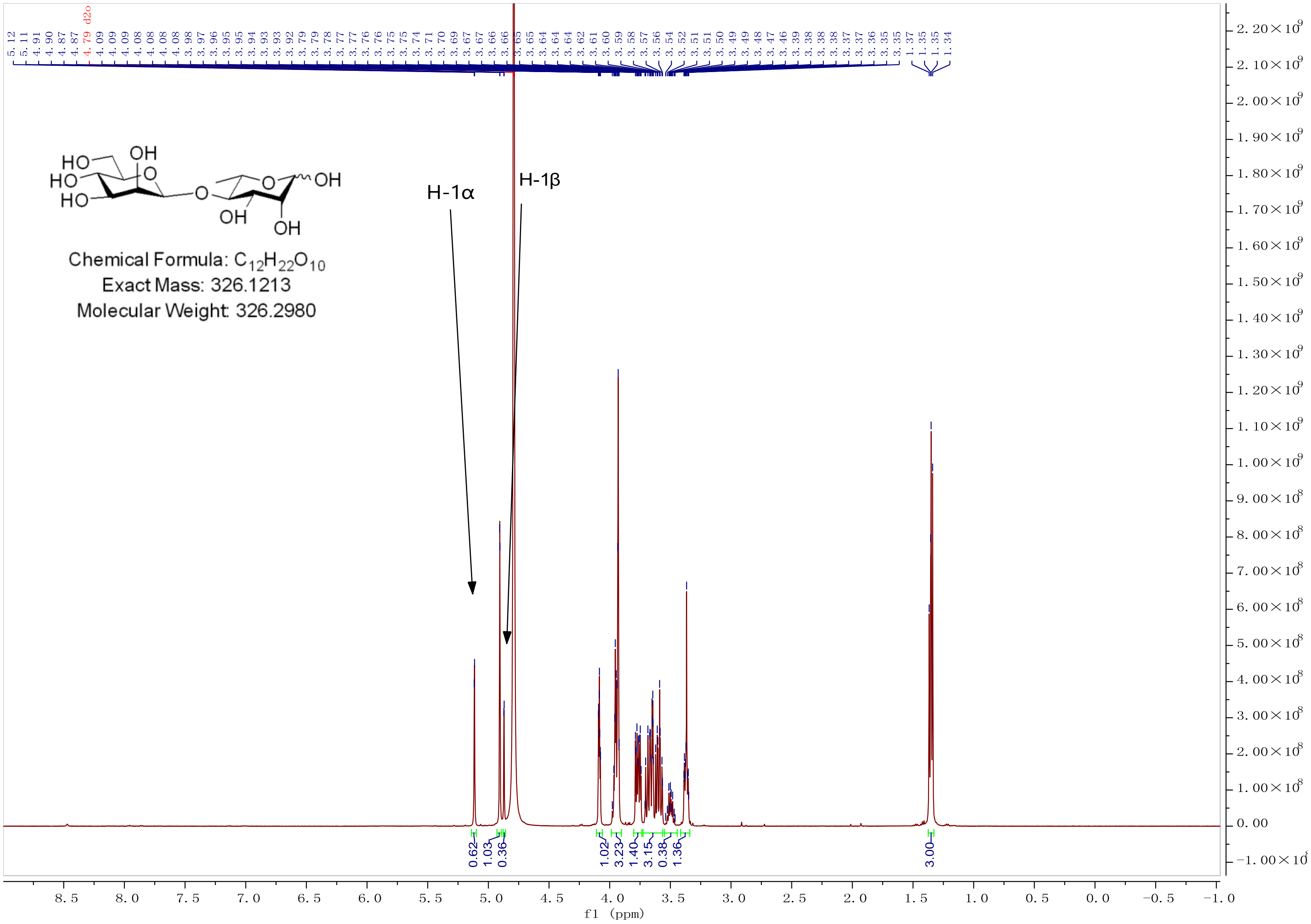

Supplementary Figure 33. **^{1}H-NMR spectrum of disaccharide 1 (D_2O, 25 ^{o}C)**

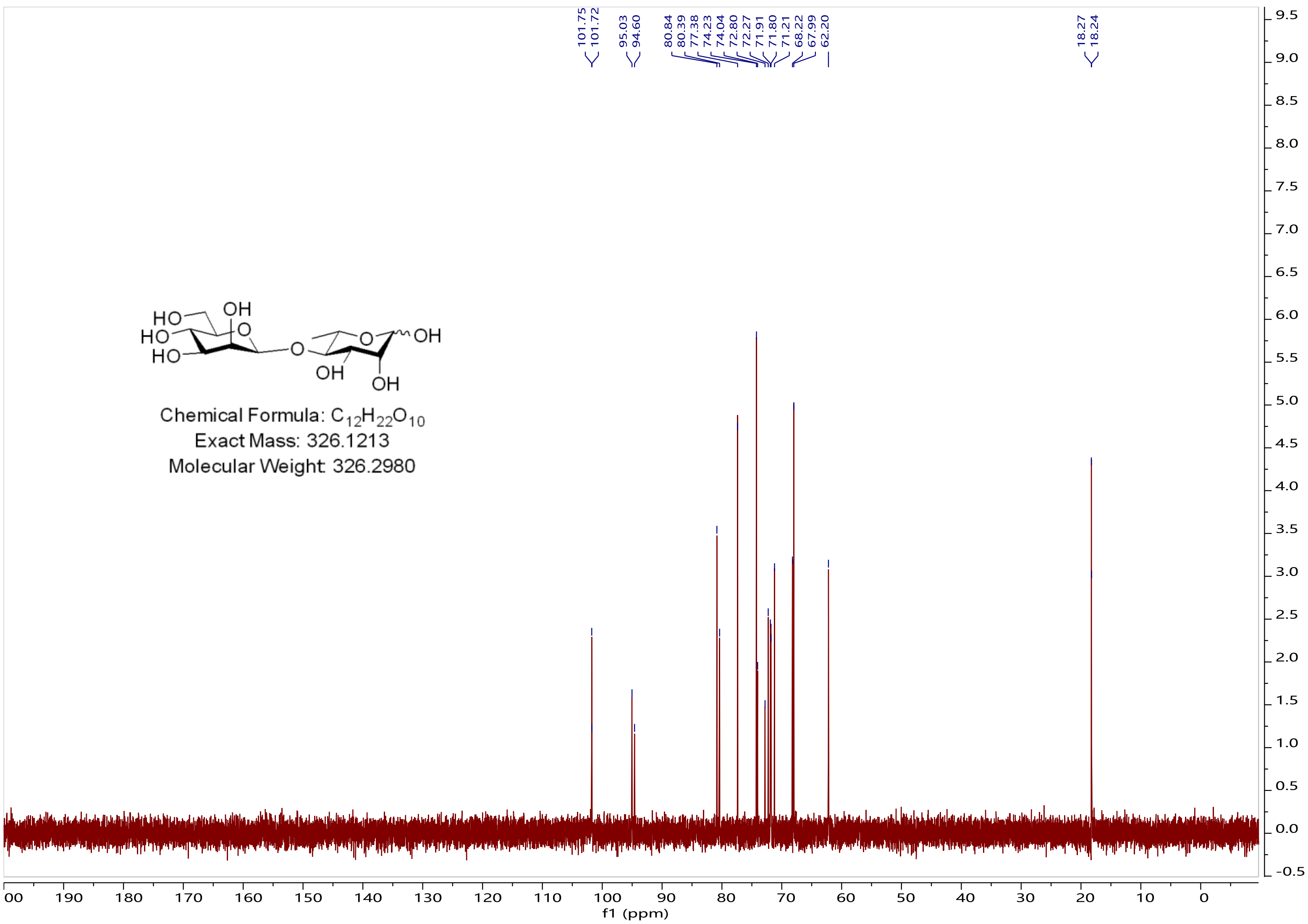

Supplementary Figure 34. ^{13}**C-NMR spectrum of disaccharide 1 (D$_2$O, 25 °C)**

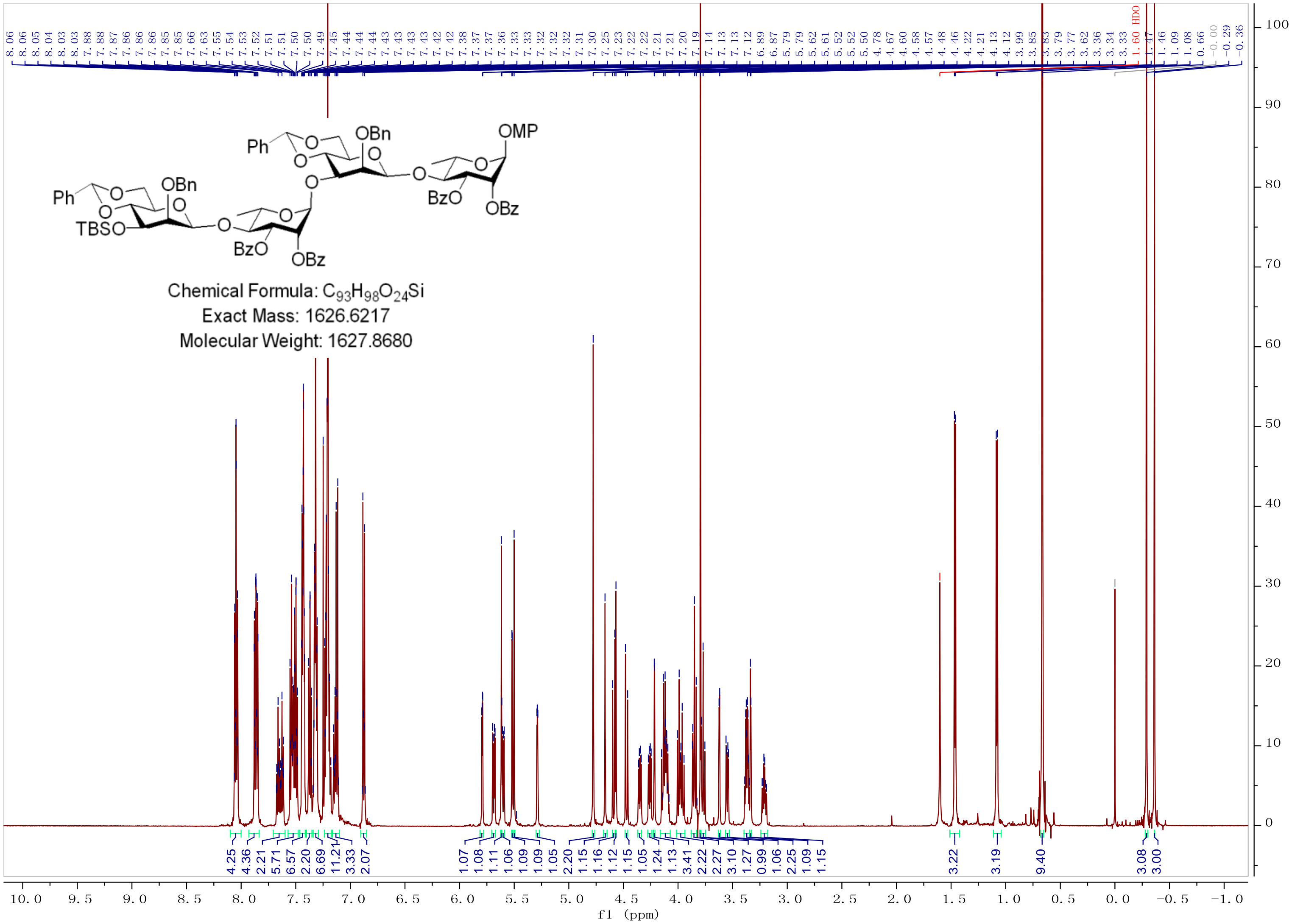

Supplementary Figure 35. ¹H-NMR spectrum of 4-mer 9 ($CDCl_3$, 25 ºC)

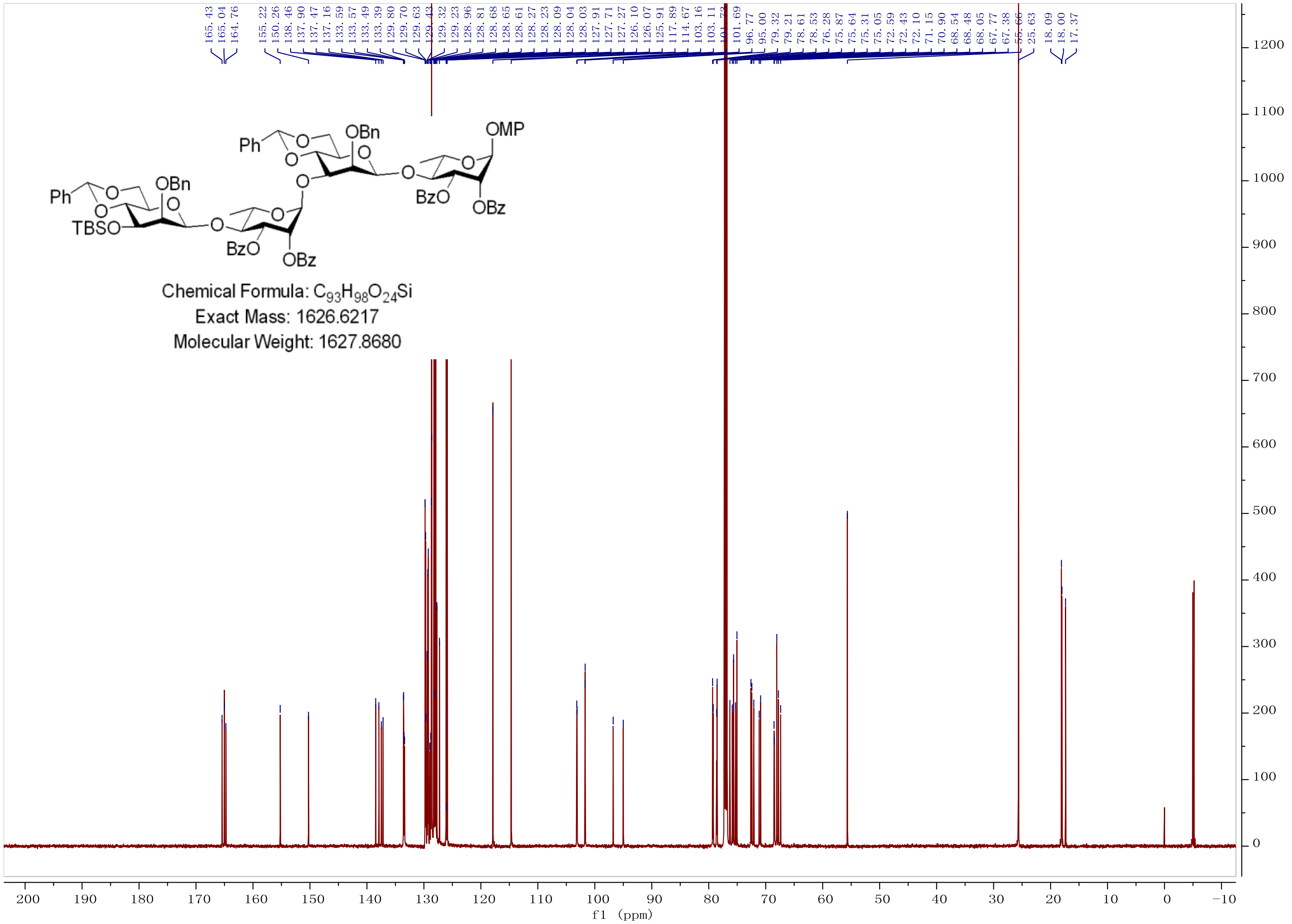

Supplementary Figure 36. ^{13}C-NMR spectrum of 4-mer 9 ($CDCl_3$, 25 °C)

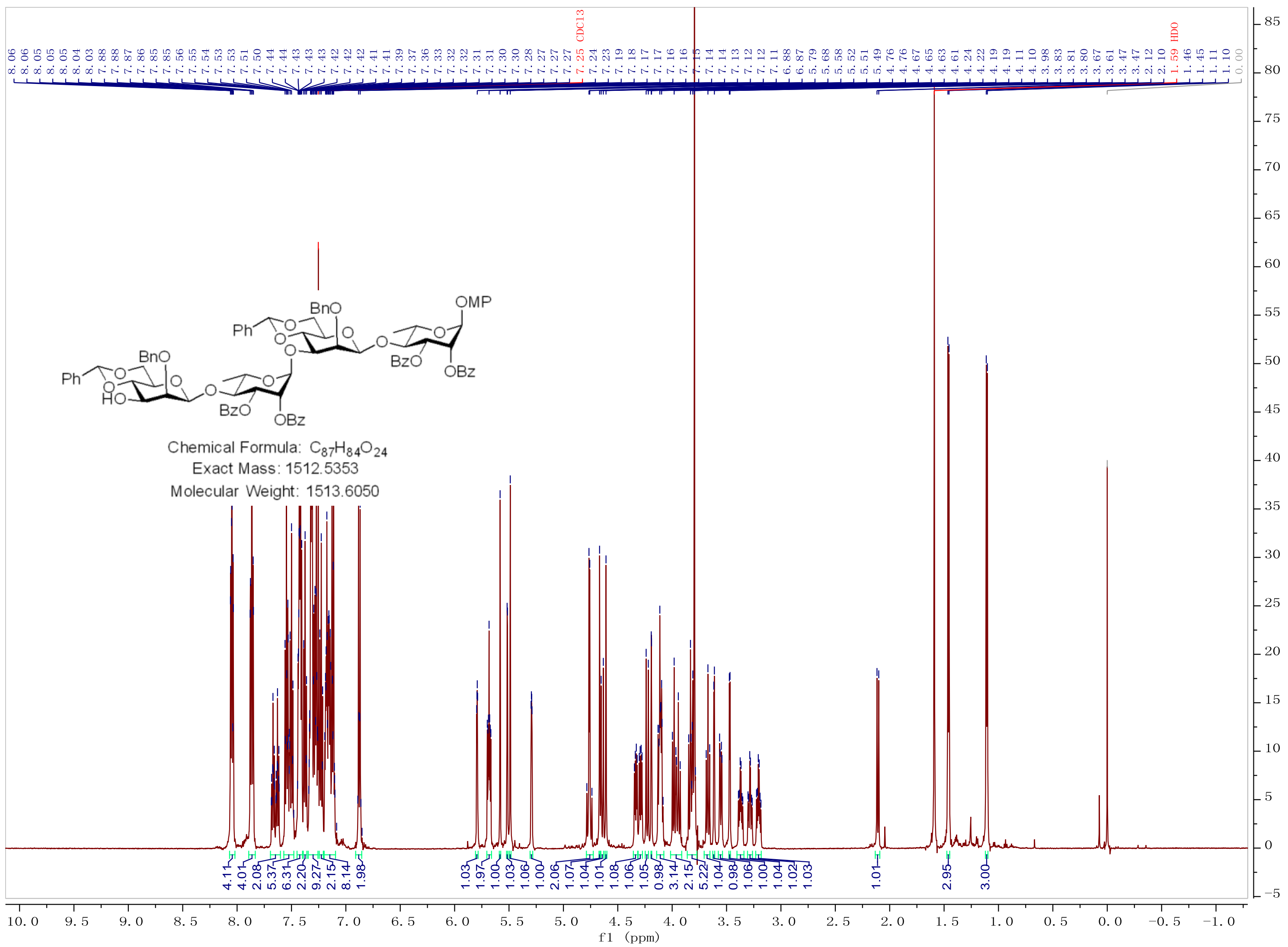

Supplementary Figure 37. ^{1}H-NMR spectrum of compound 9[A] (CDCl$_3$, 25 °C)

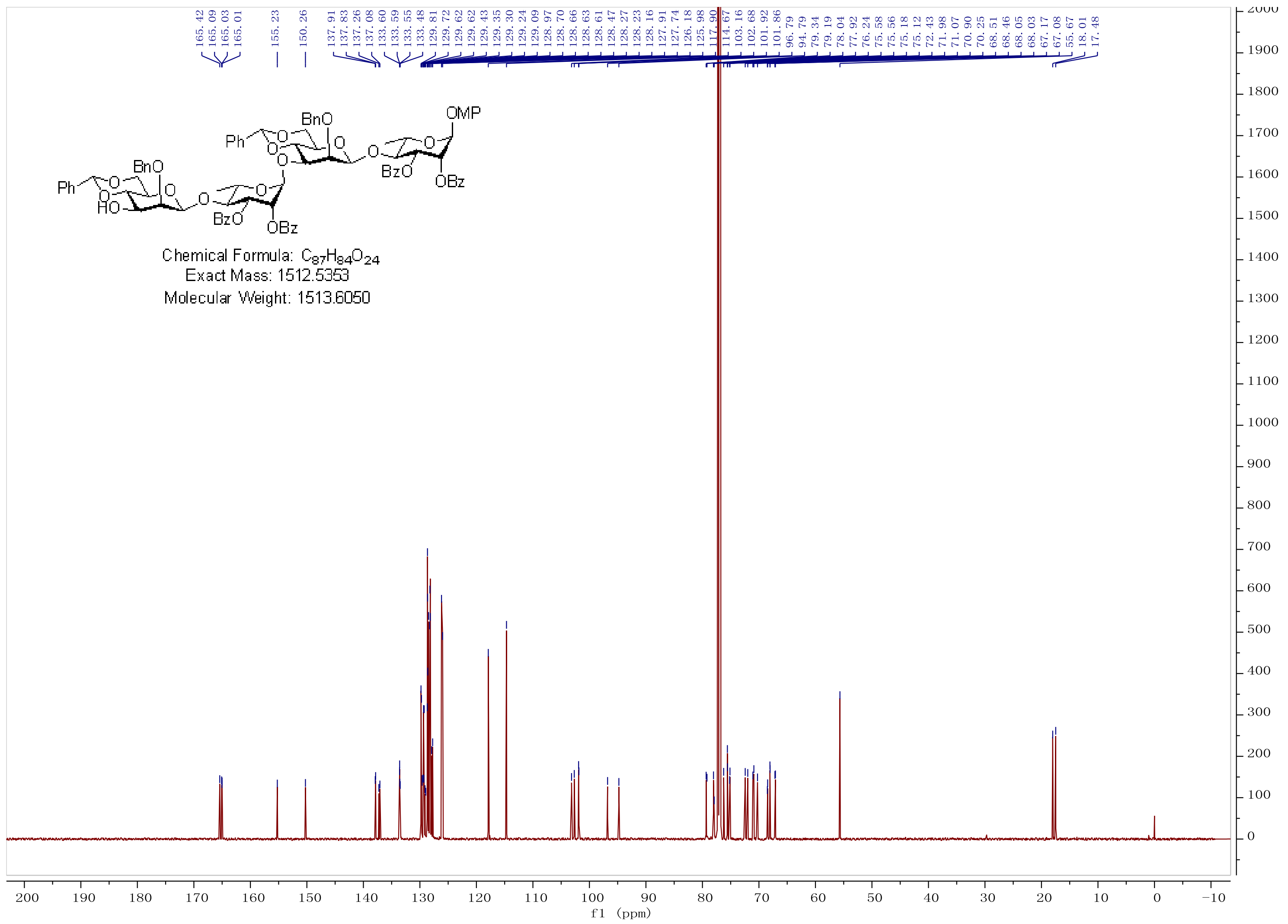

Supplementary Figure 38. **^{13}C-NMR spectrum of compound 9^{A} ($CDCl_3$, 25 ^{o}C)**

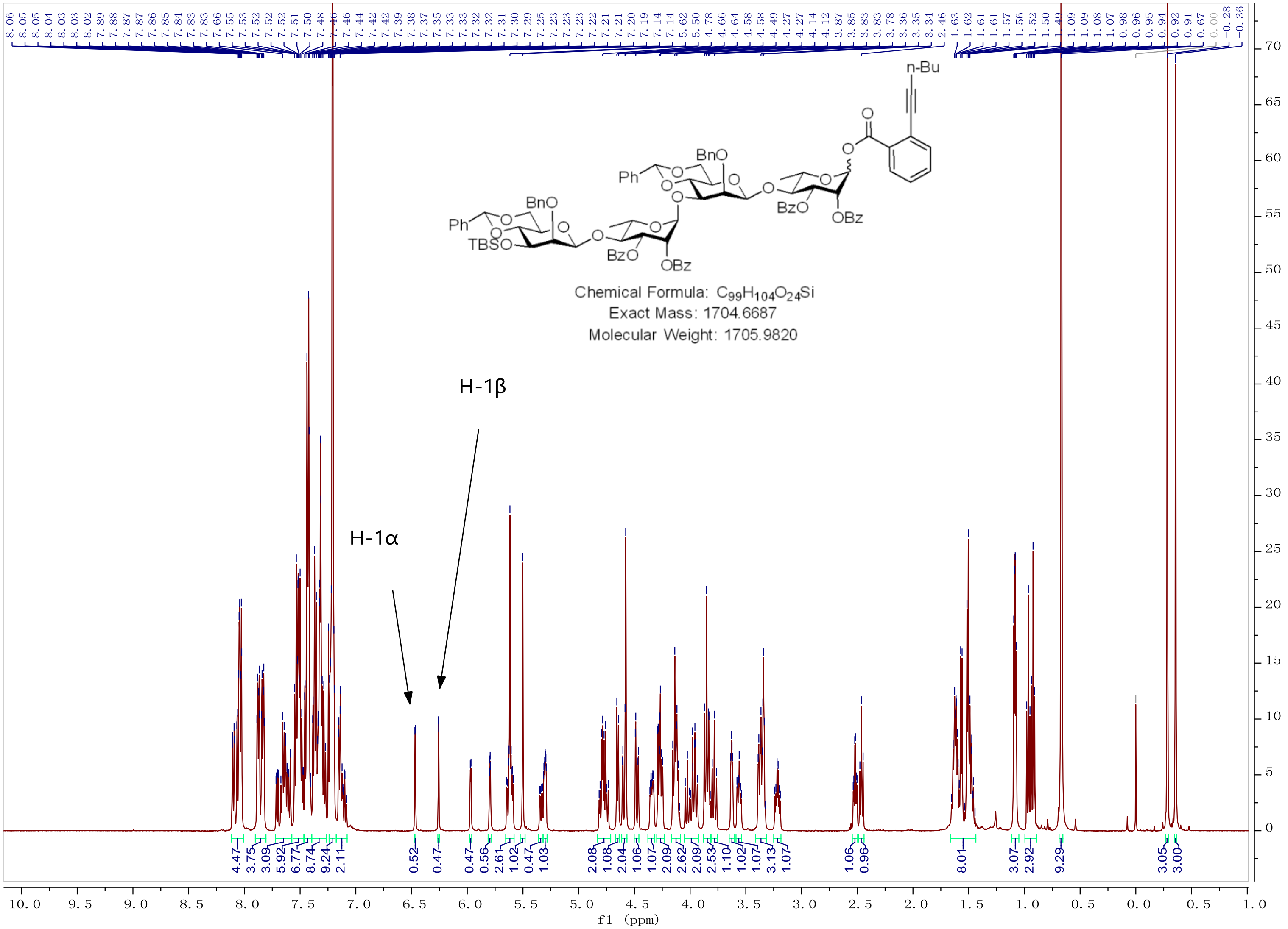

Supplementary Figure 39. ^{1}**H-NMR spectrum of compound 9**^{D} **(CDCl**$_3$**, 25 °C)**

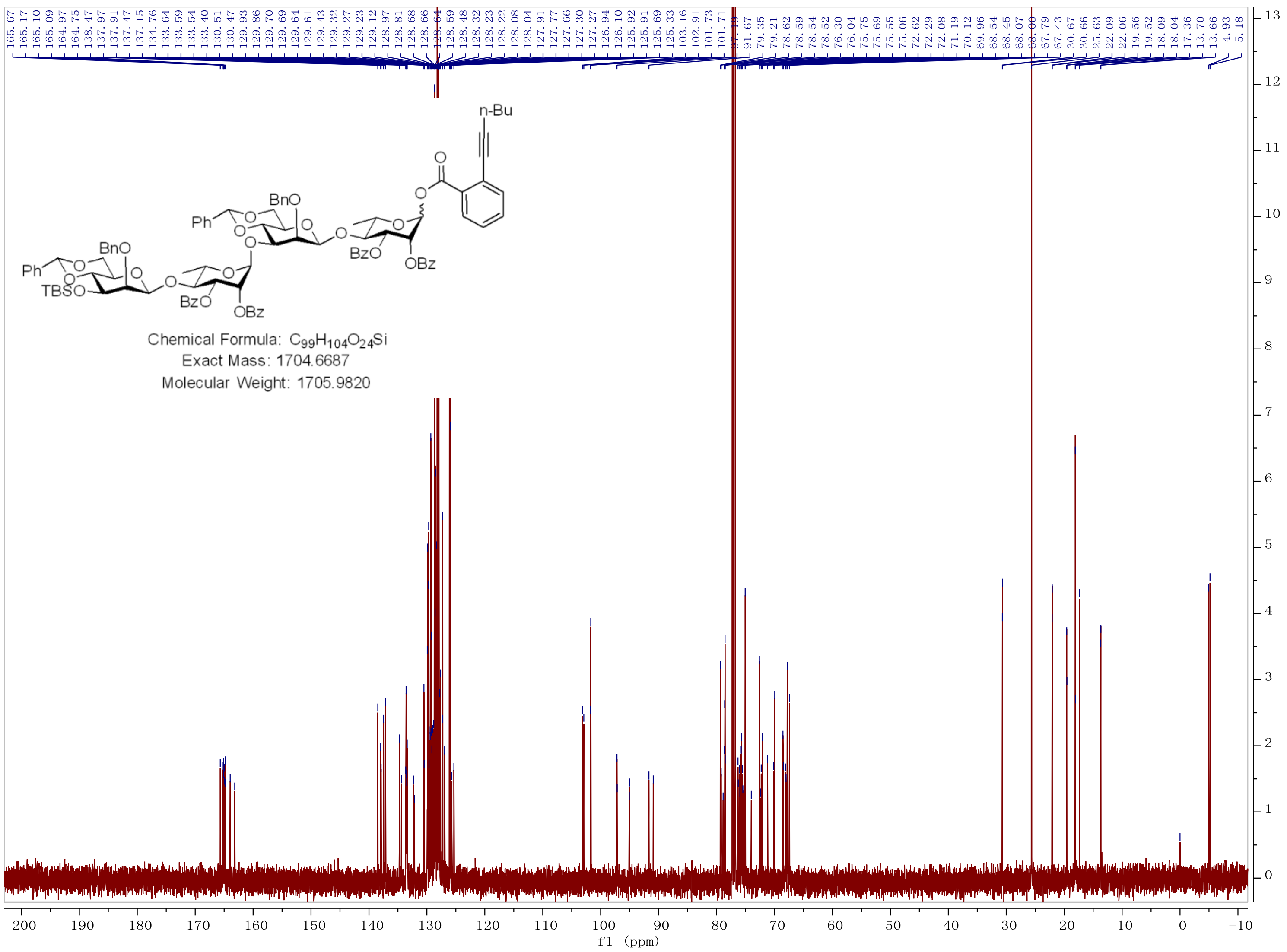

Supplementary Figure 40. ^{13}C-NMR spectrum of compound 9^{D} ($CDCl_3$, 25 ^{o}C)

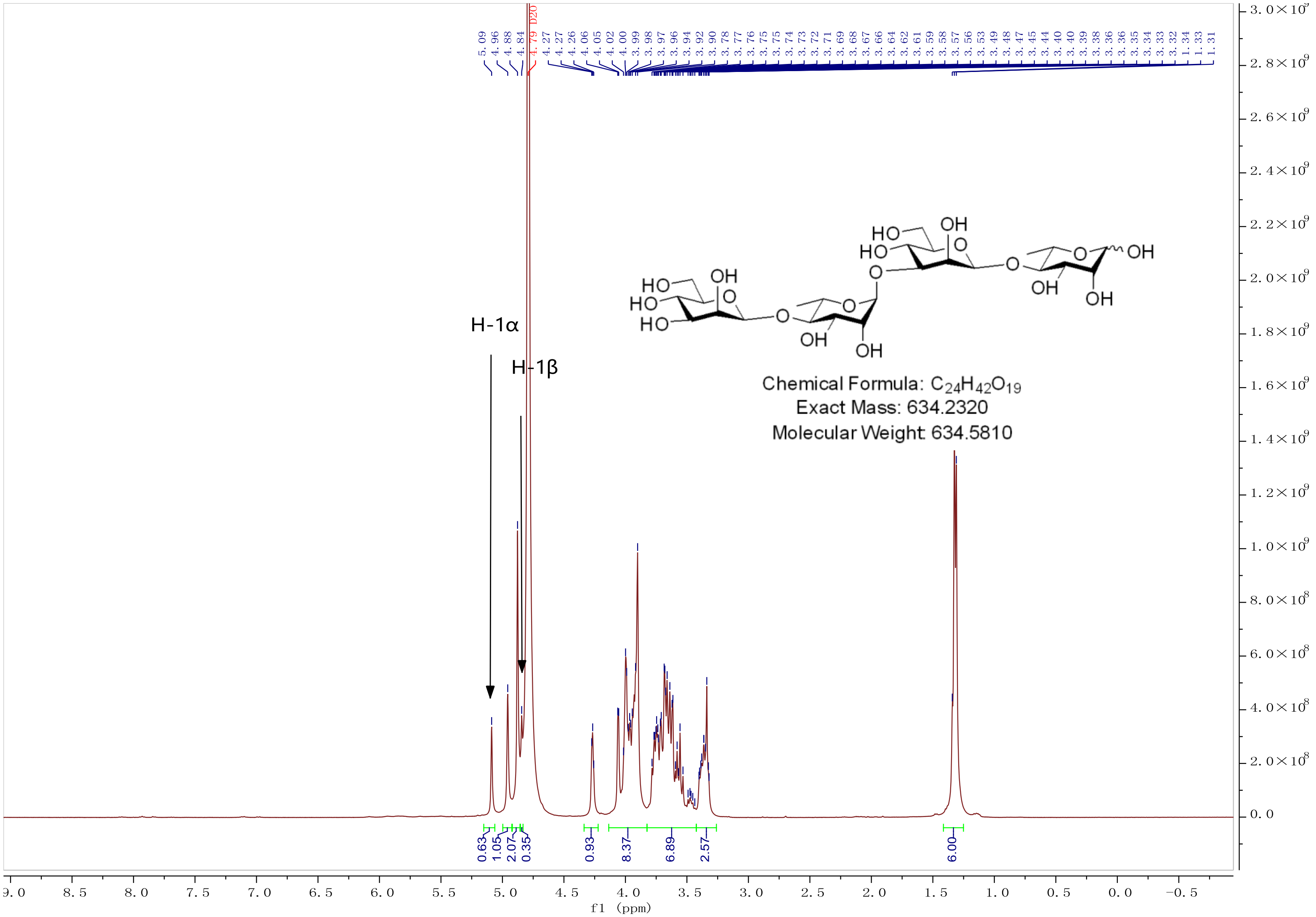

Supplementary Figure 41. ^{1}H-NMR spectrum of 4-mer 2 (D_2O, 25 °C)

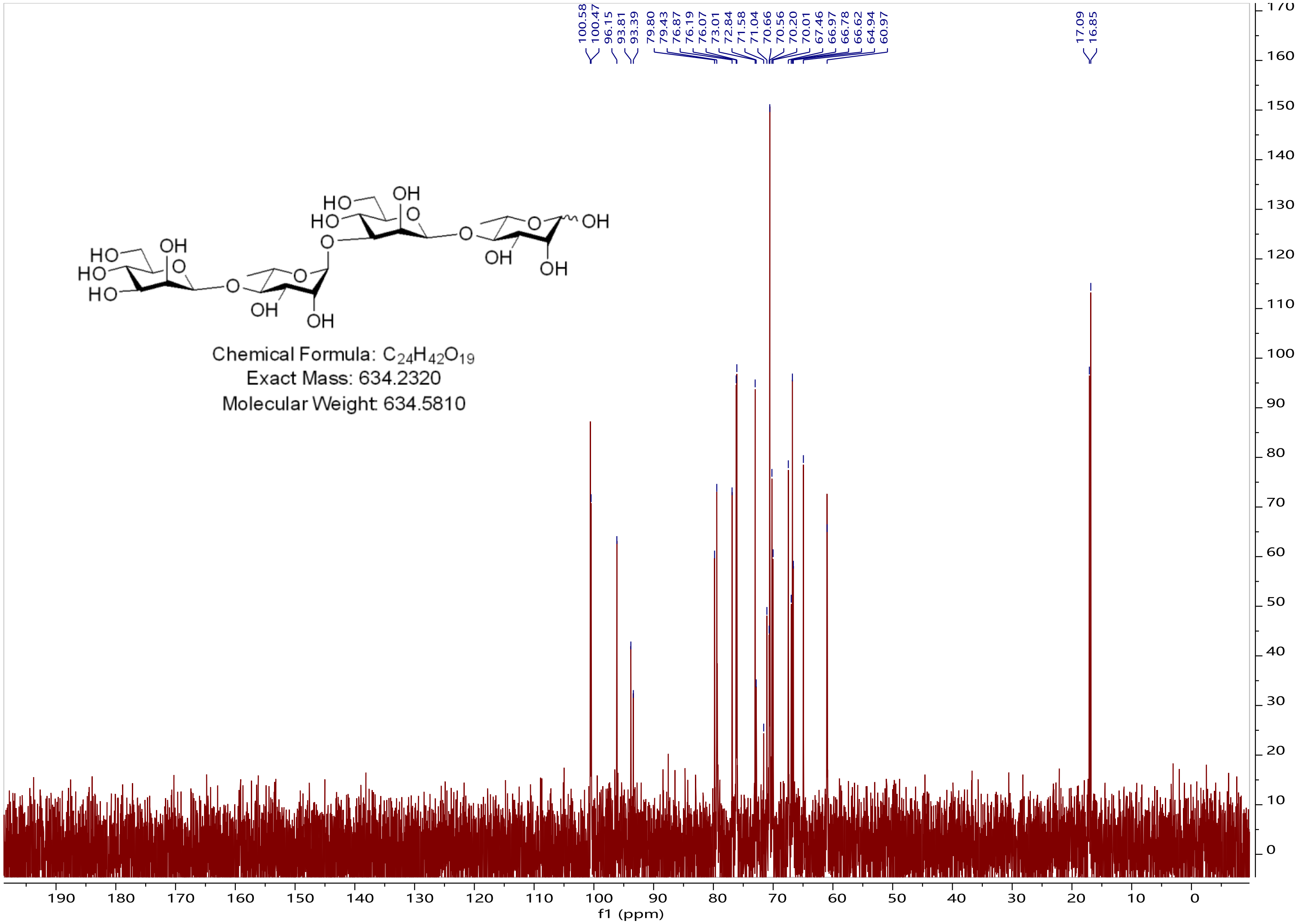

Supplementary Figure 42. ^{13}**C-NMR spectrum of 4-mer 2 (D$_2$O, 25 °C)**

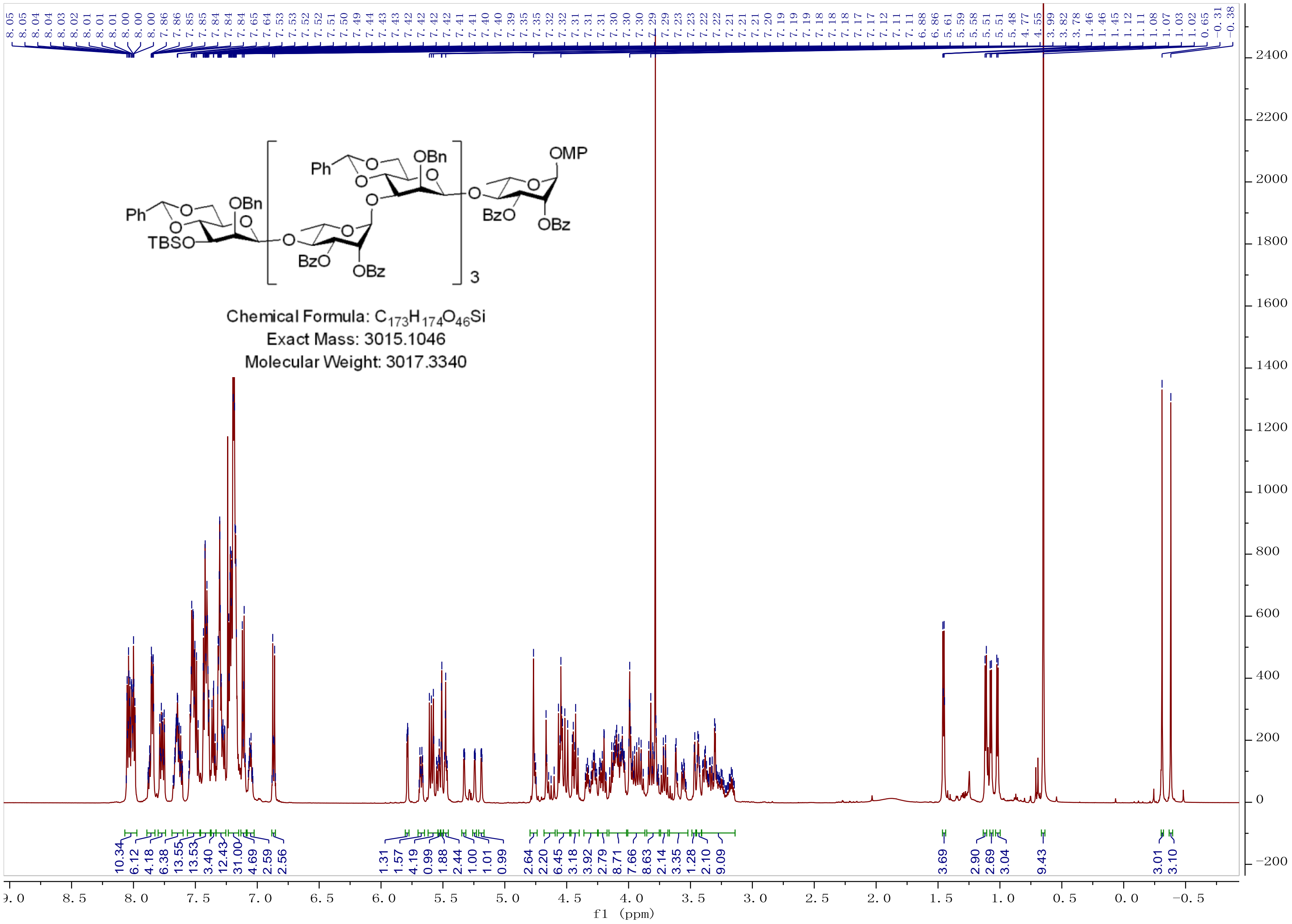

Supplementary Figure 43. ^{1}H-NMR spectrum of 8-mer 10 ($CDCl_3$, 25 ºC)

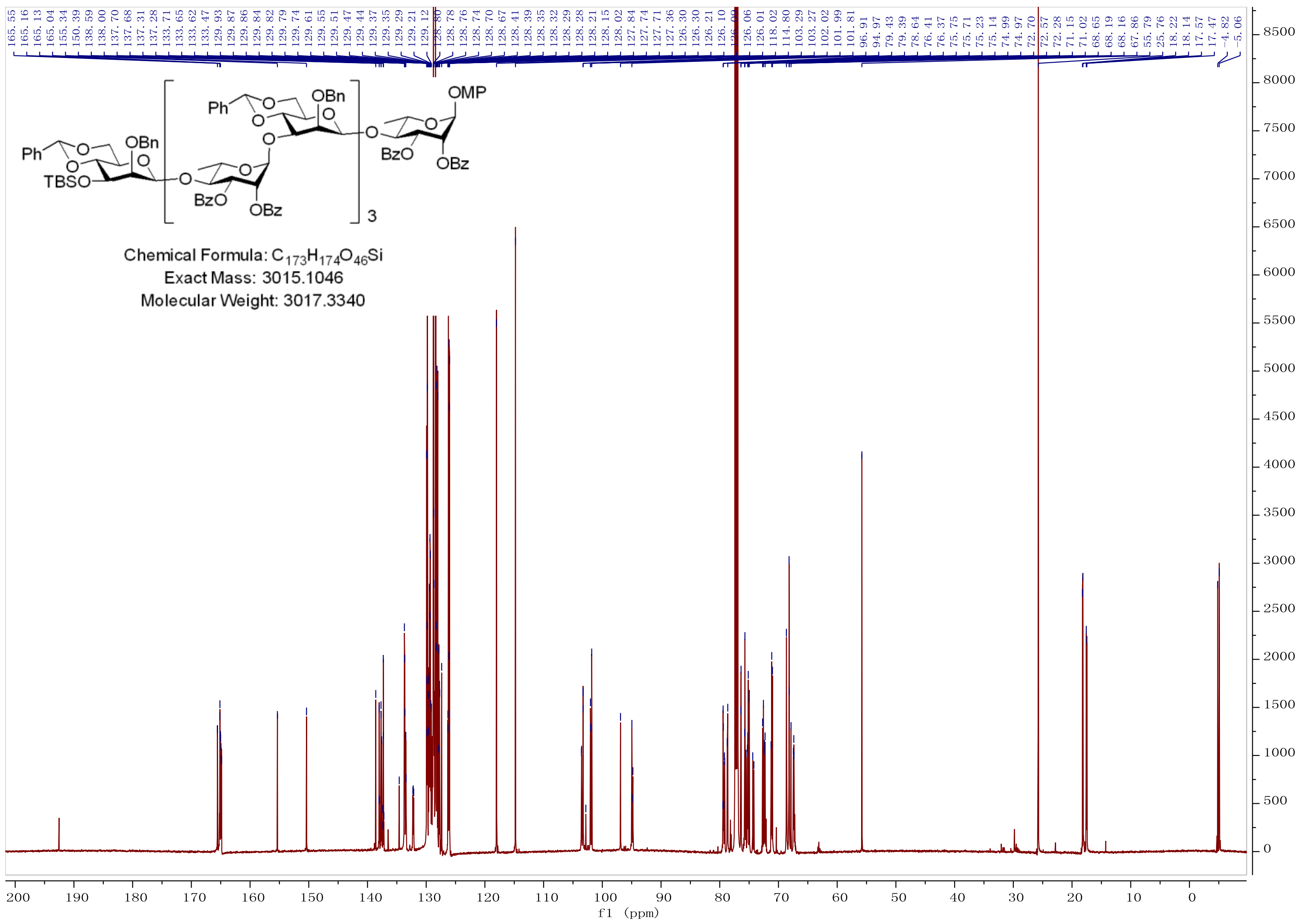

Supplementary Figure 44. ^{13}C-NMR spectrum of 8-mer 10 ($CDCl_3$, 25 ^oC)

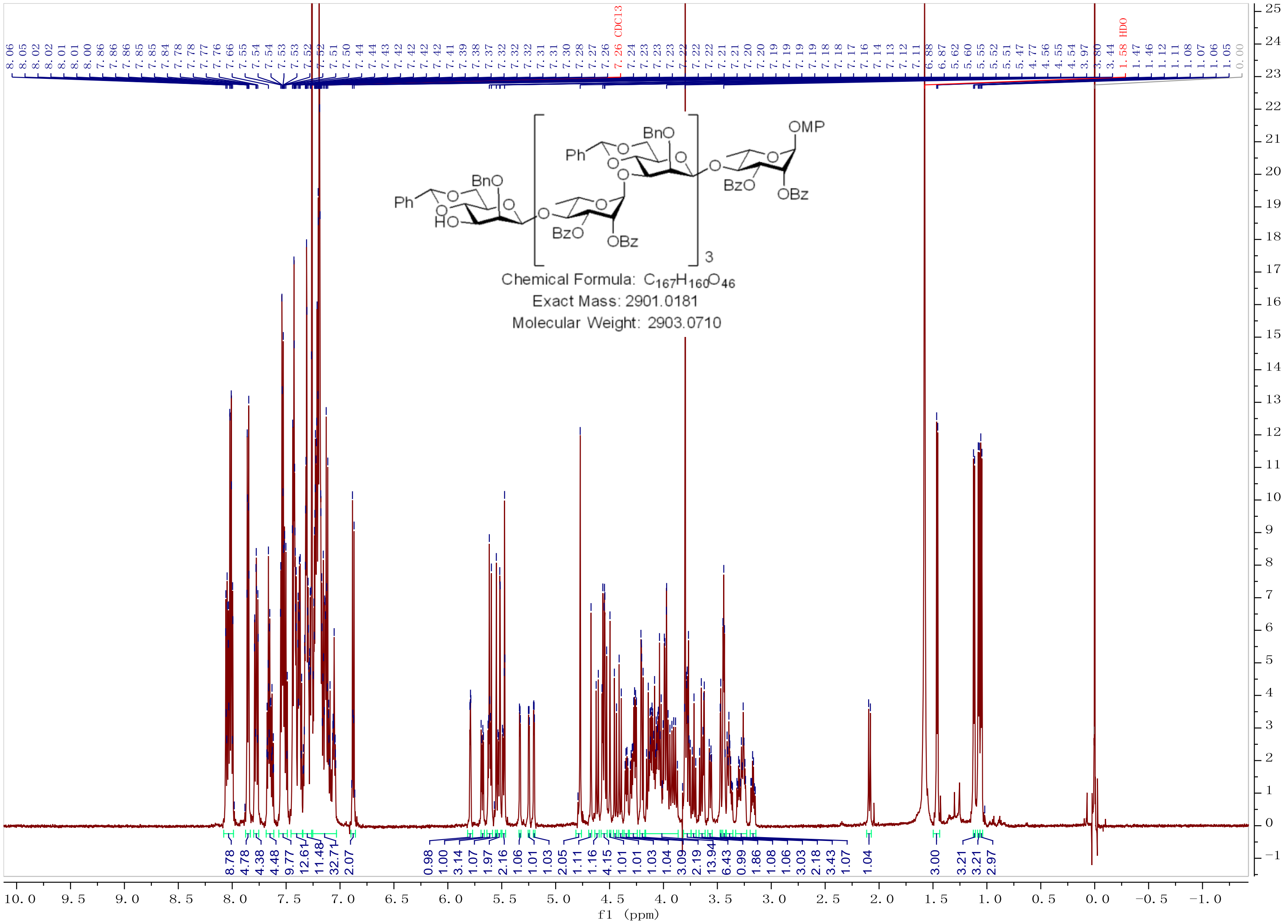

Supplementary Figure 45. ^{1}H-NMR spectrum of compound 10^A ($CDCl_3$, 25 ^{o}C)

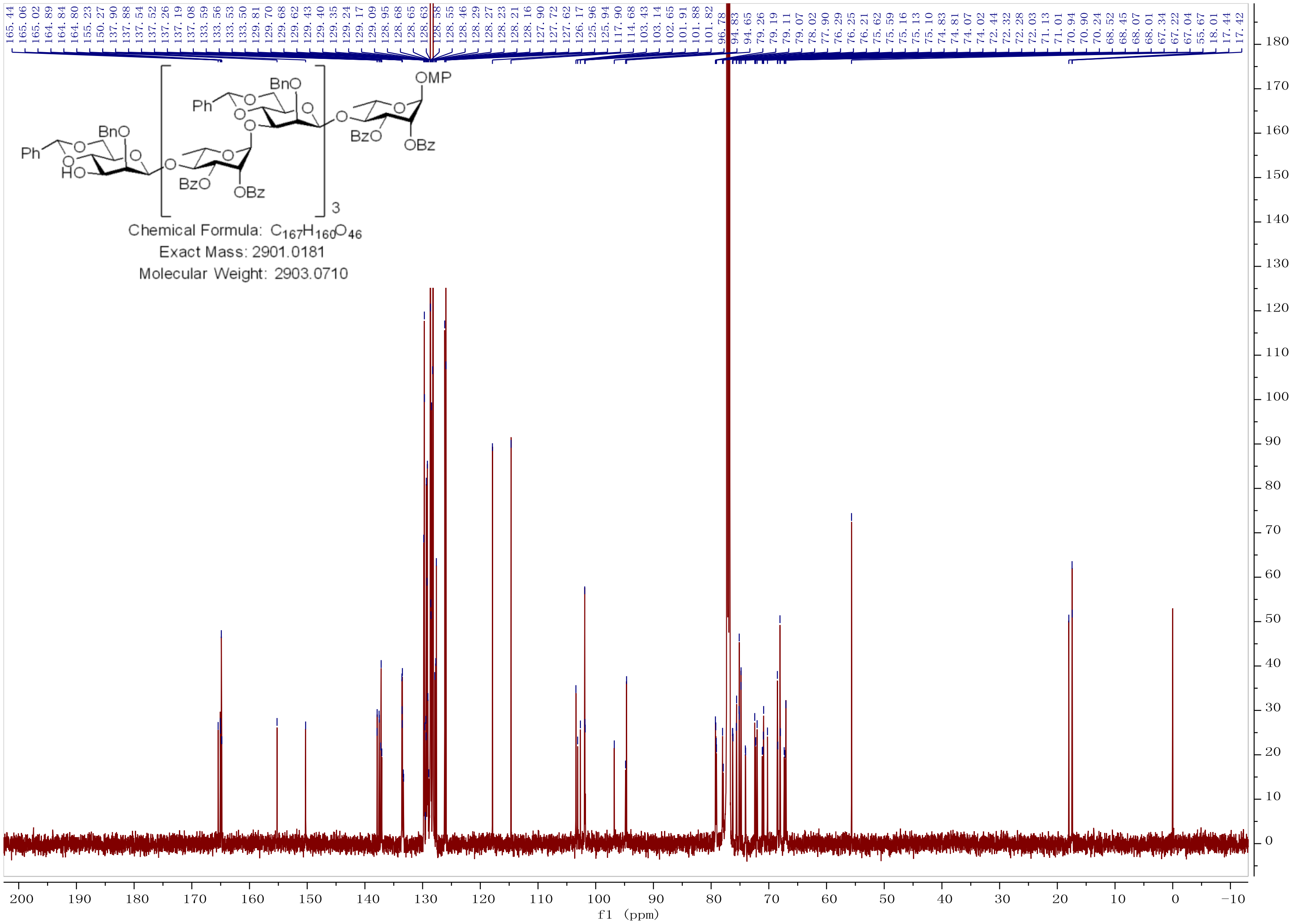

Supplementary Figure 46. ^{13}**C-NMR spectrum of compound 10**^{A} **(CDCl**$_3$**, 25 °C)**

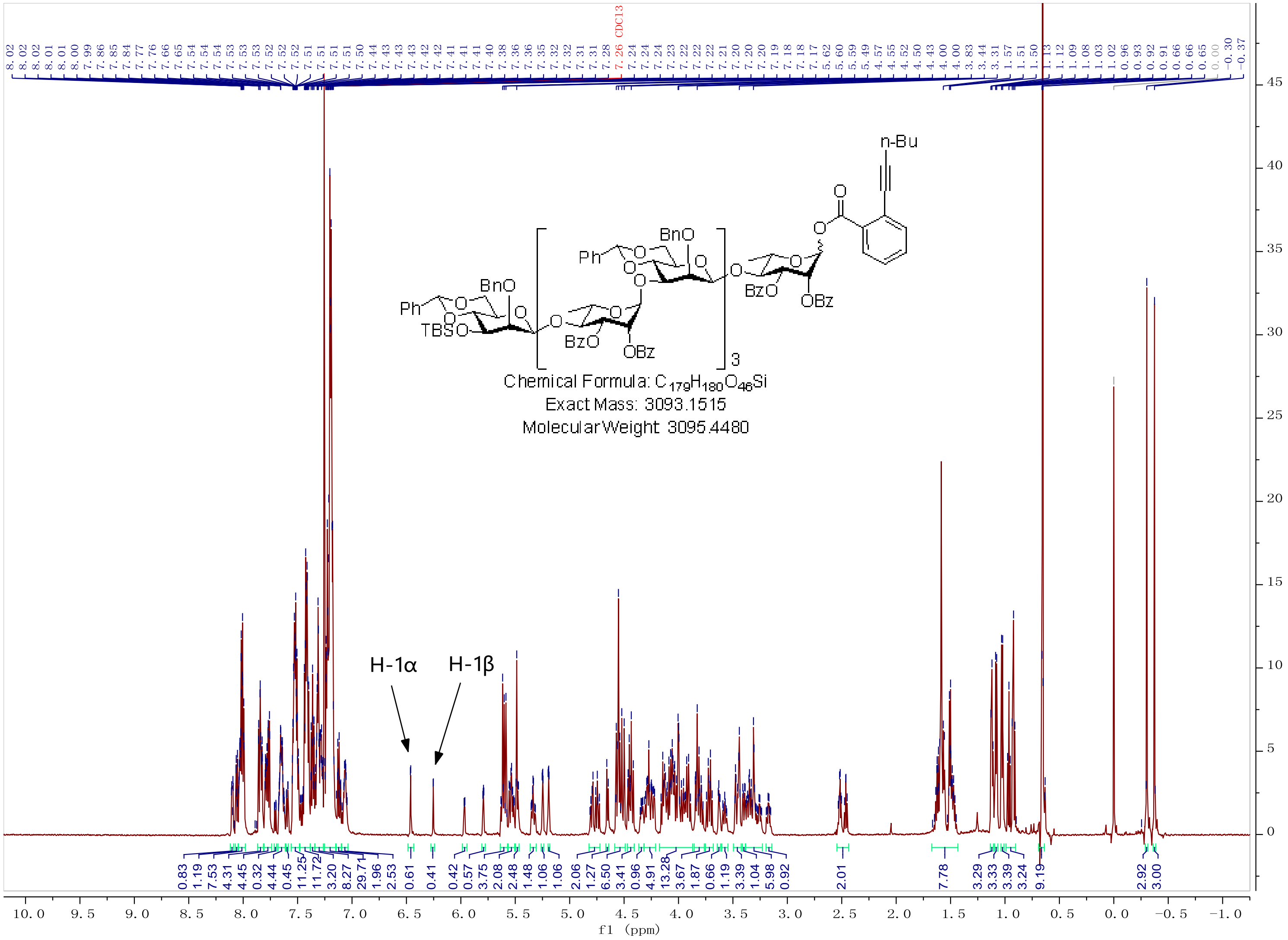

Supplementary Figure 47. ^{1}H-NMR spectrum of compound 10^{D} ($CDCl_3$, 25 ^{o}C).

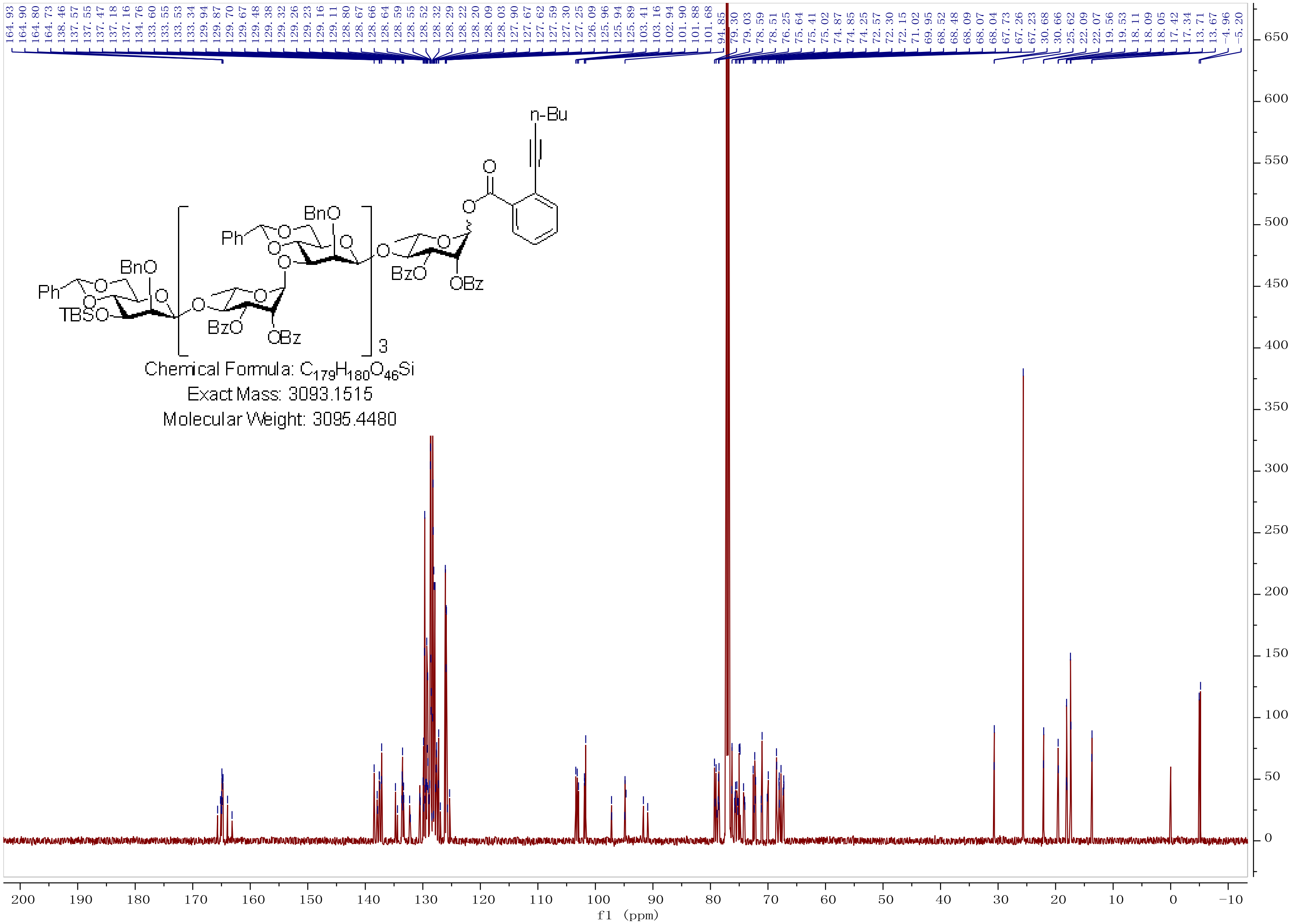

Supplementary Figure 48. ^{13}C-NMR spectrum of compound 10^{D} (CDCl$_3$, 25 °C)

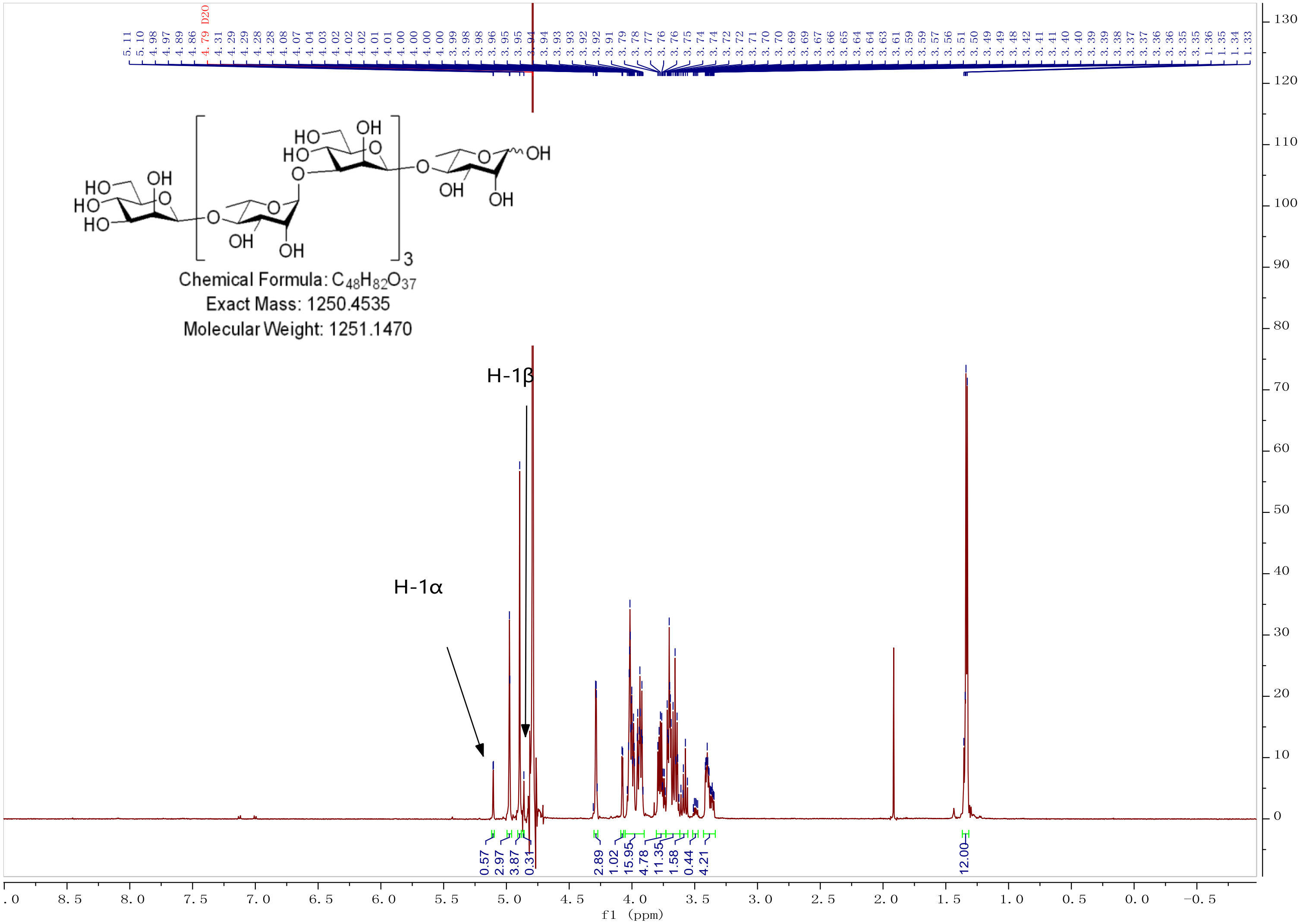

Supplementary Figure 49. ^{1}H-NMR spectrum of 8-mer 3 (D_2O, 25 °C)

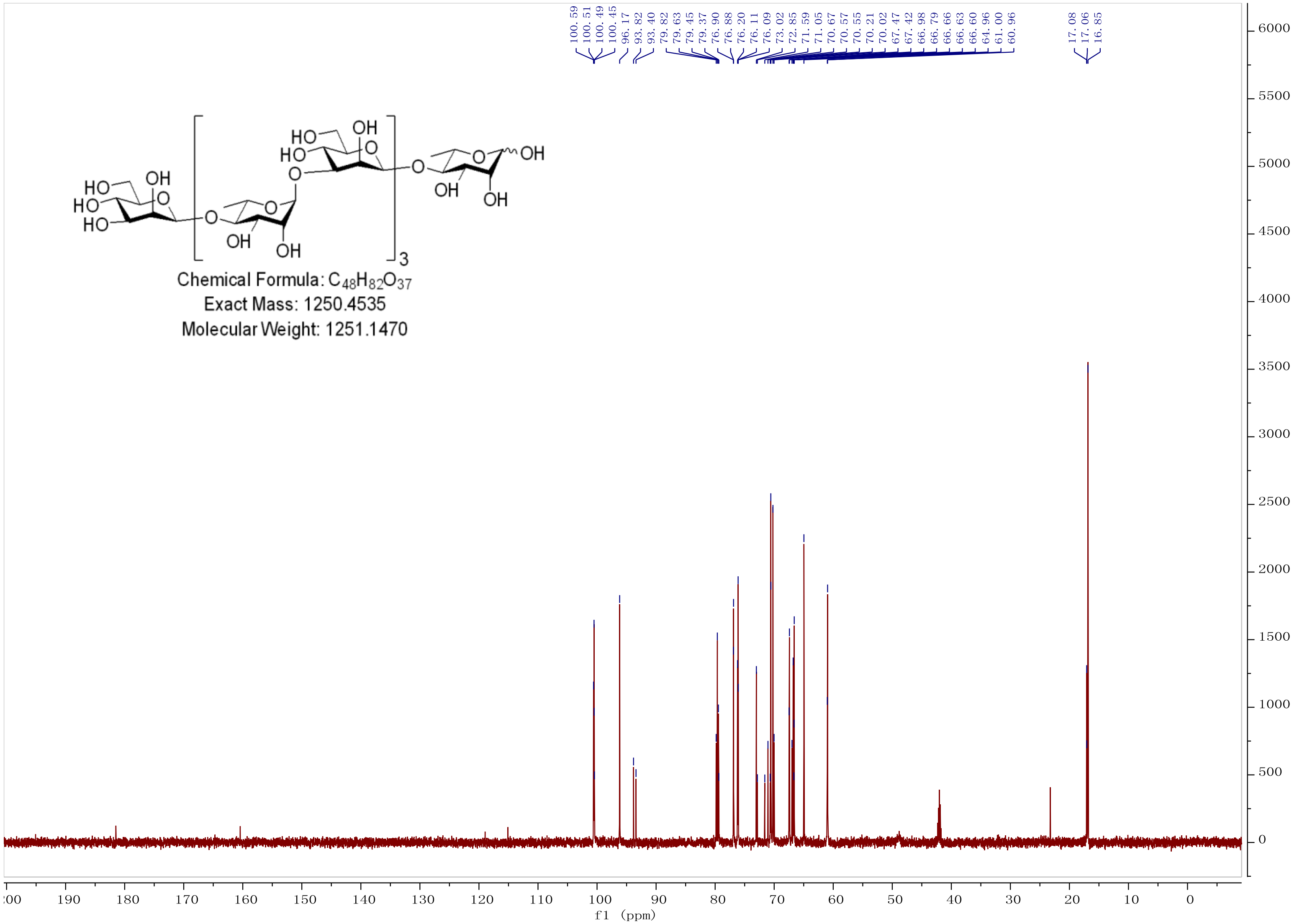

Supplementary Figure 50. ^{13}C-NMR spectrum of 8-mer 3 (D_2O, 25 °C)

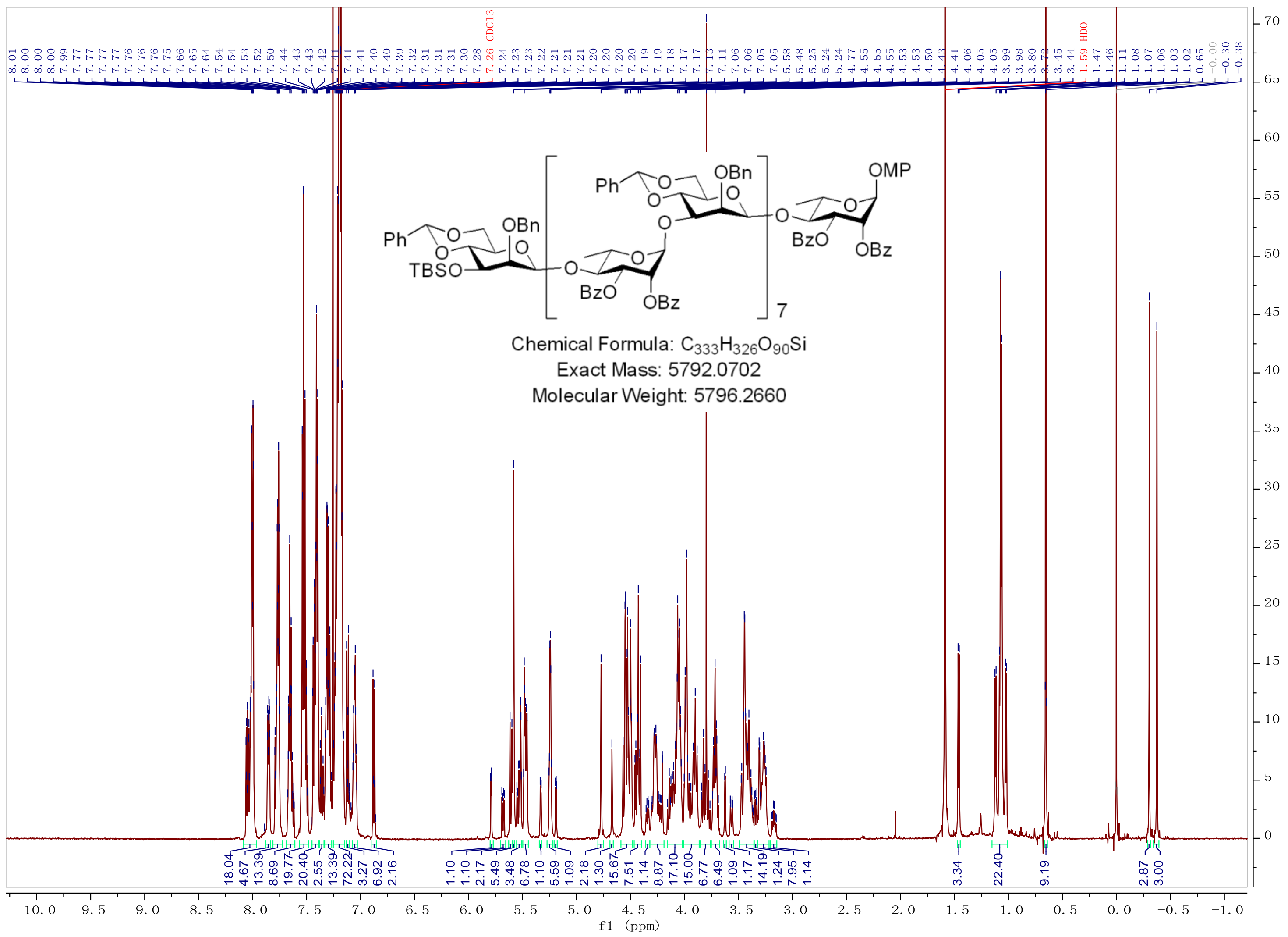

Supplementary Figure 51. **^{1}H-NMR spectrum of 16-mer 11 ($CDCl_3$, 25 °C)**

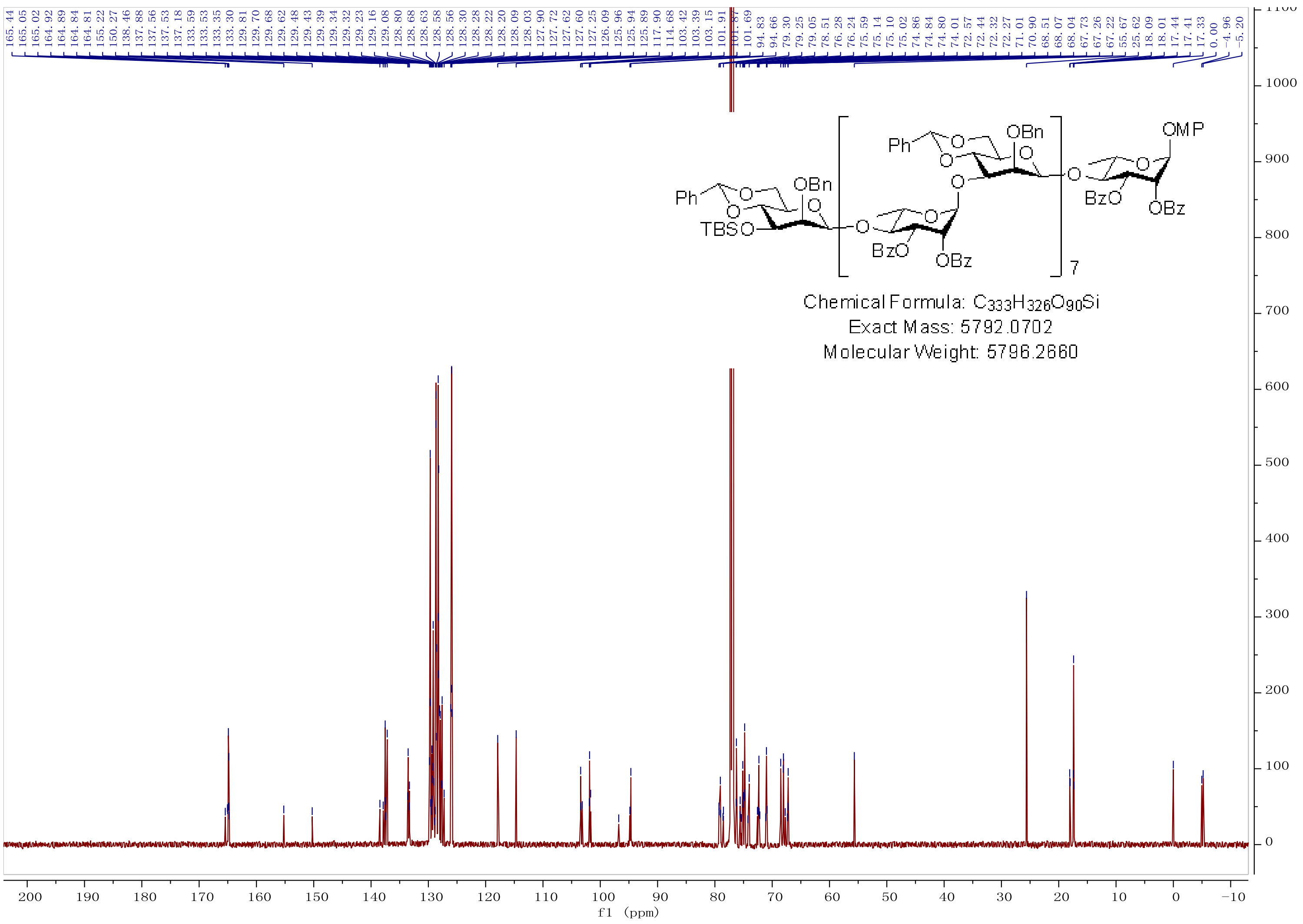

Supplementary Figure 52. ^{13}C-NMR spectrum of 16-mer 11 ($CDCl_3$, 25 ºC)

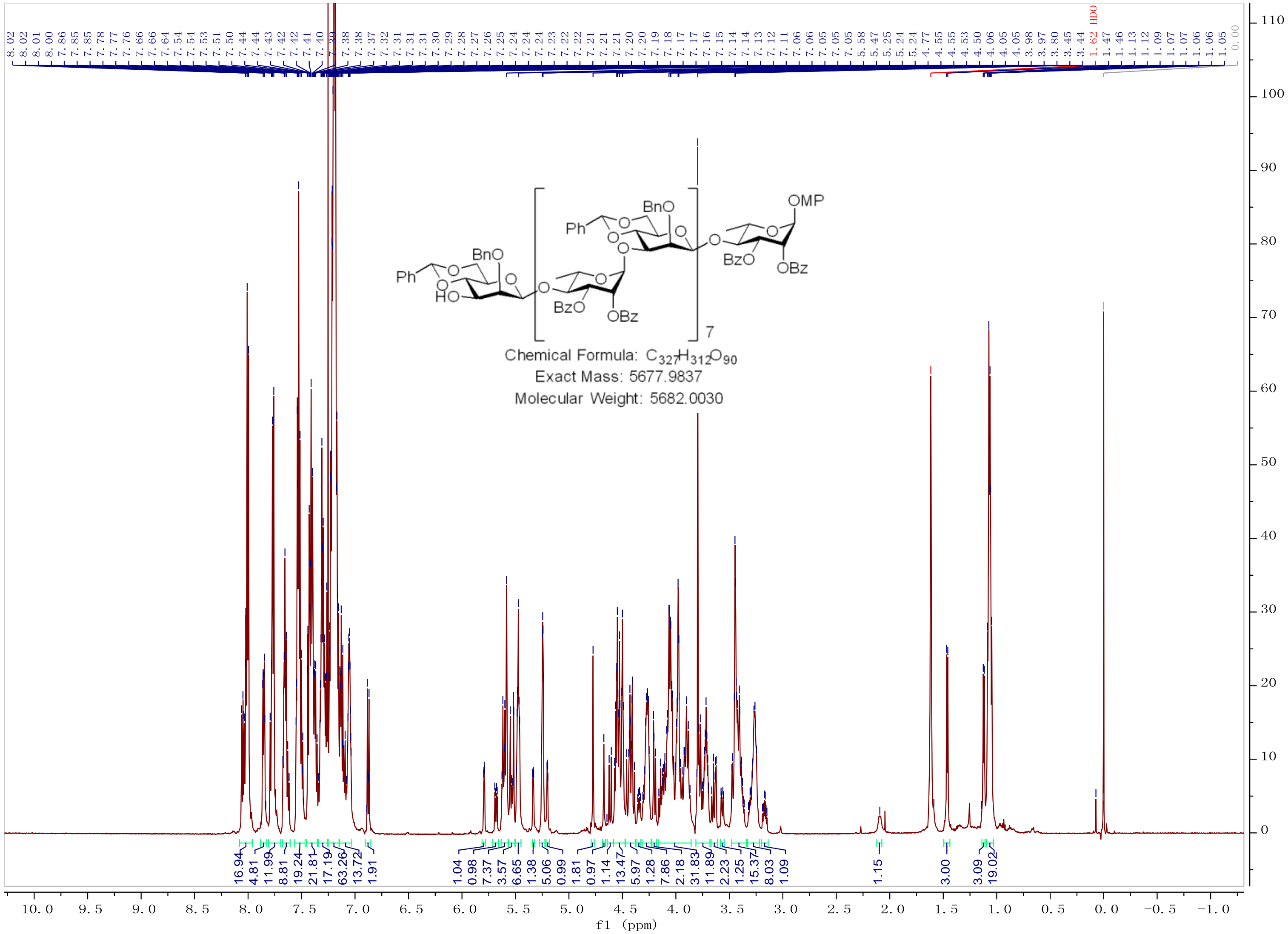

Supplementary Figure 53. ^{1}**H-NMR spectrum of compound 11**^{A} **($CDCl_3$, 25 °C)**

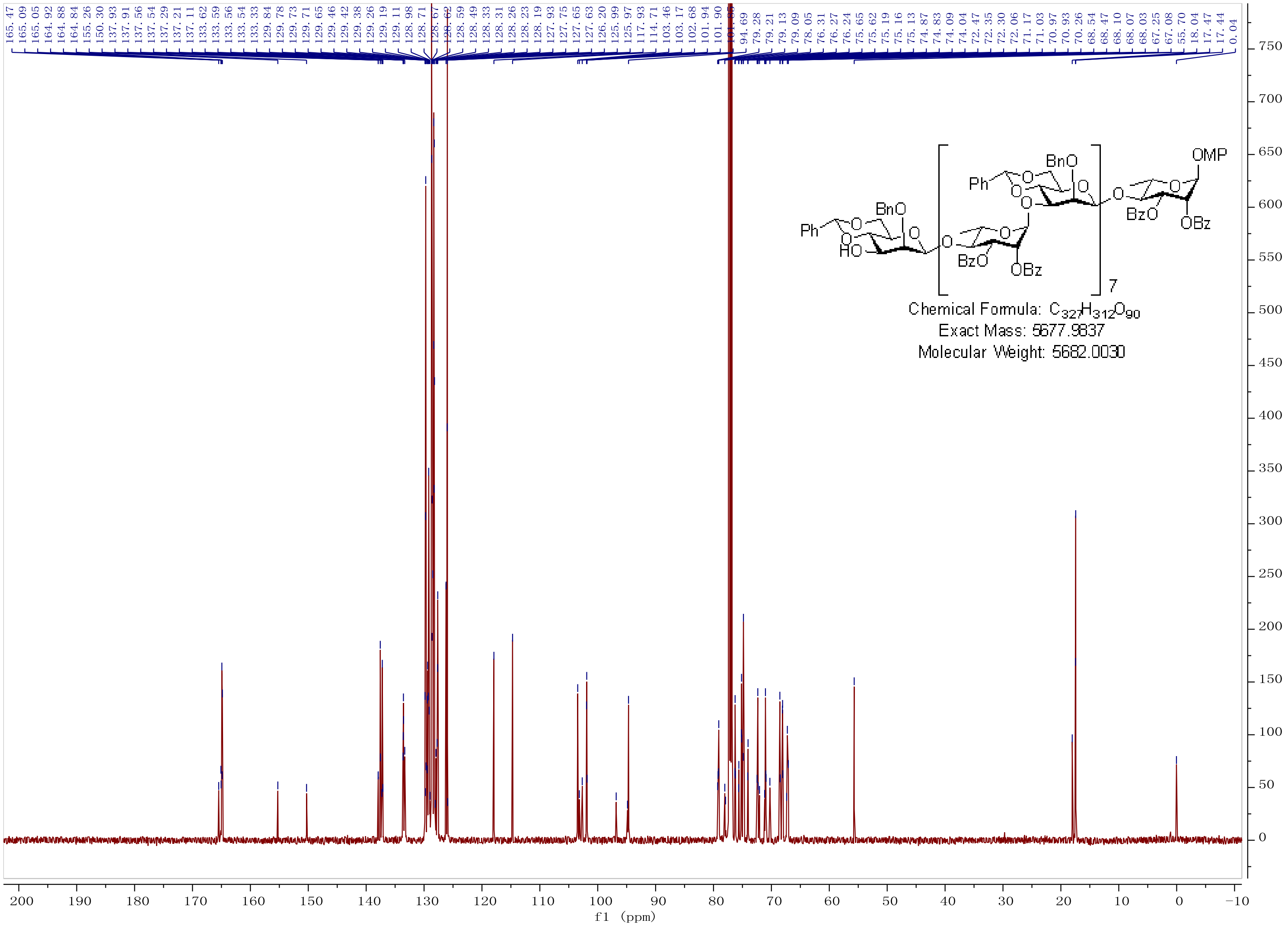

Supplementary Figure 54. ^{13}**C-NMR spectrum of compound 11**^{A} **(CDCl**$_3$**, 25 °C)**

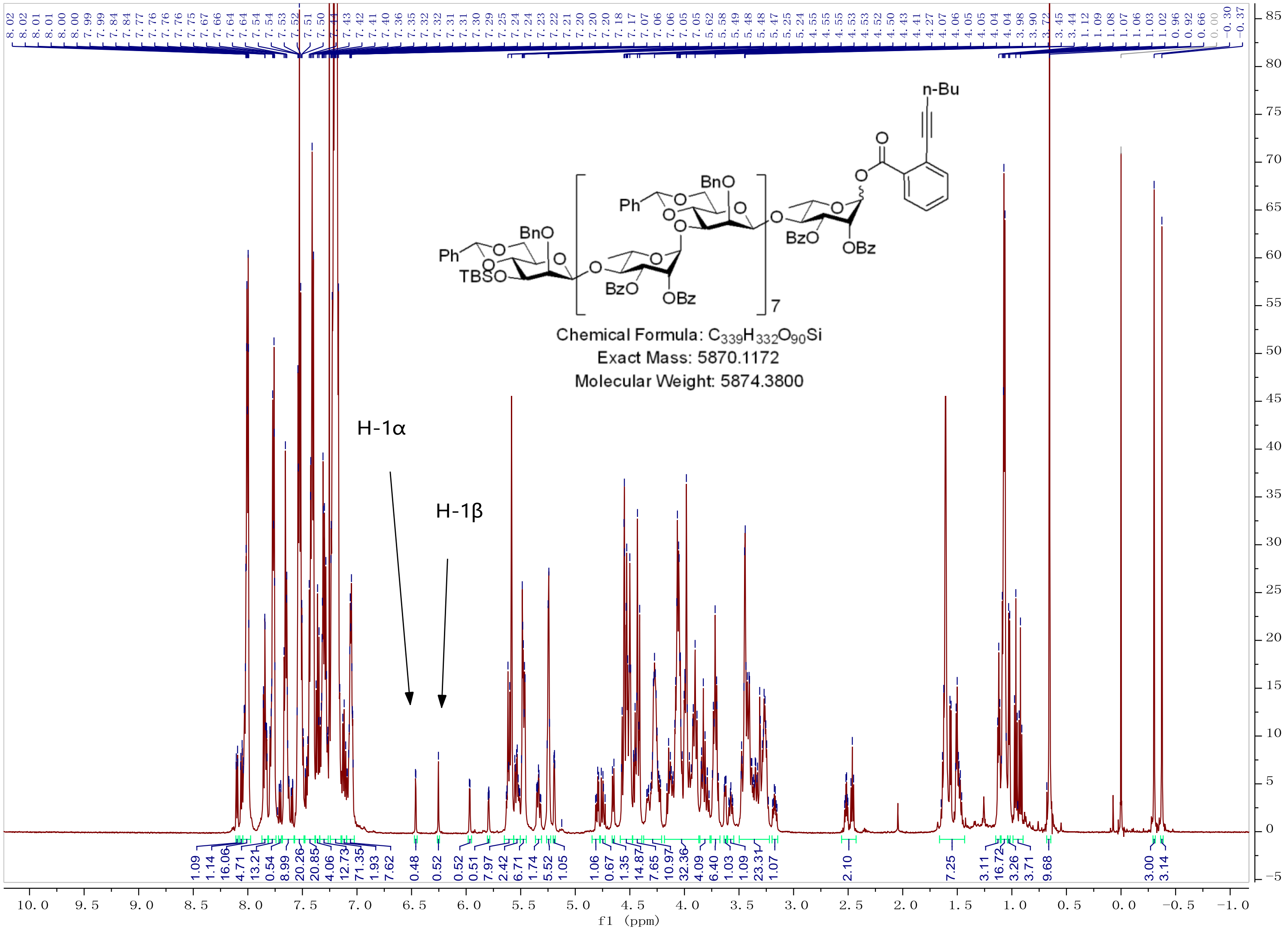

Supplementary Figure 55. ^{1}**H-NMR spectrum of compound 11**^{D} **(CDCl$_3$, 25 °C)**

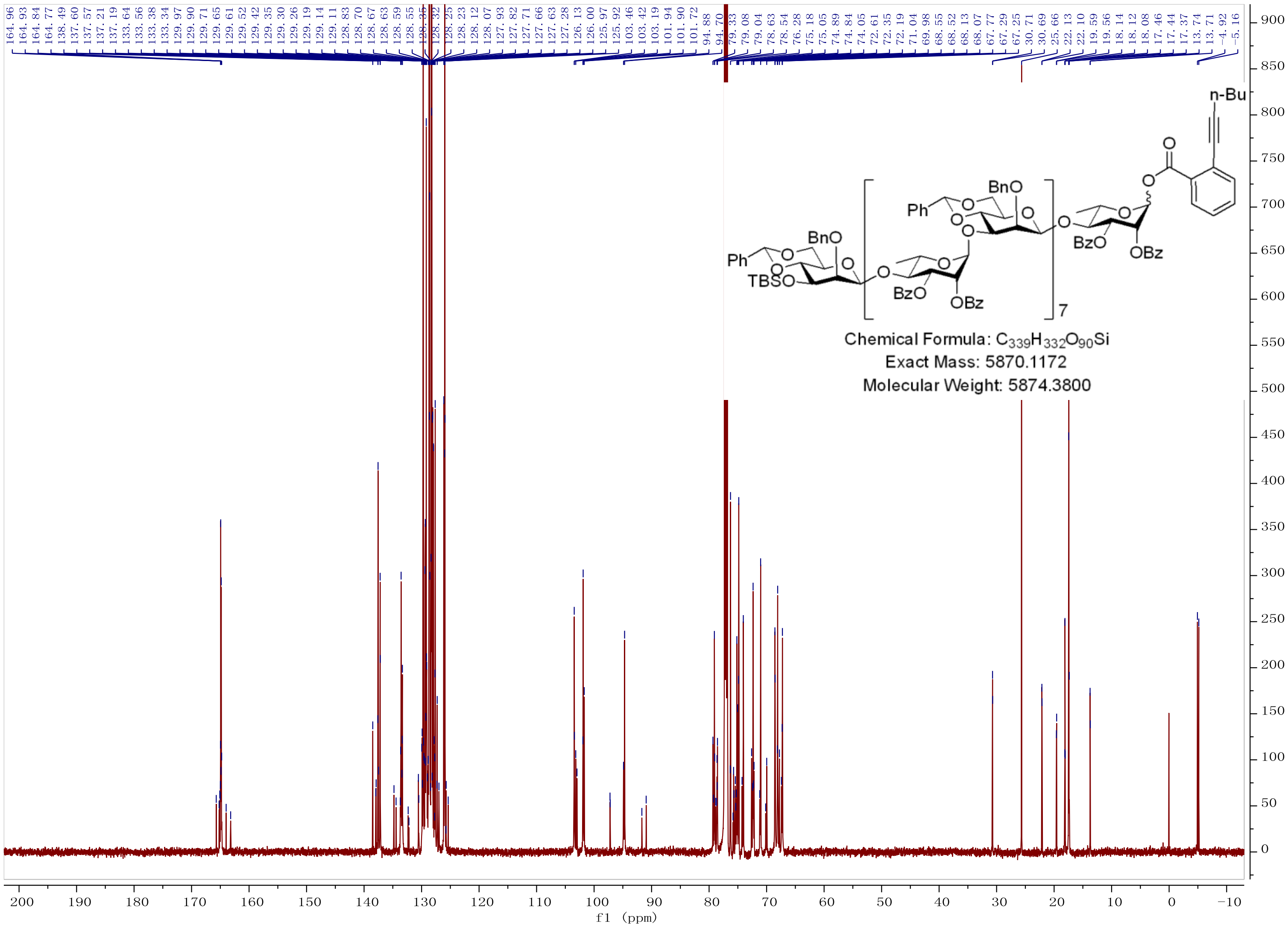

Supplementary Figure 56. ^{13}C NMR-spectrum of compound 11^{D} ($CDCl_3$, 25 °C)

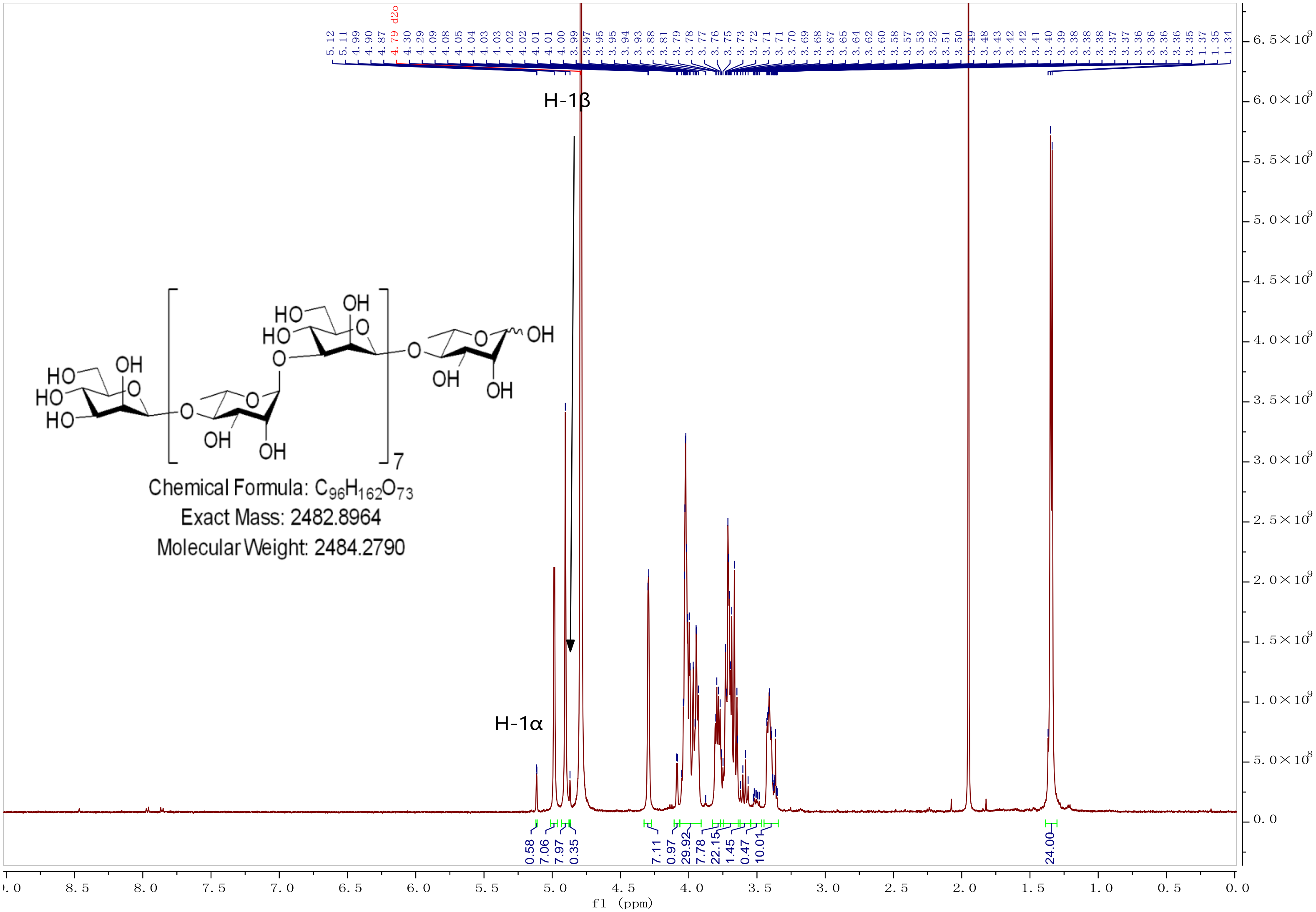

Supplementary Figure 57. ^{1}H-NMR spectra of 16-mer 4 (D_2O, 25 °C)

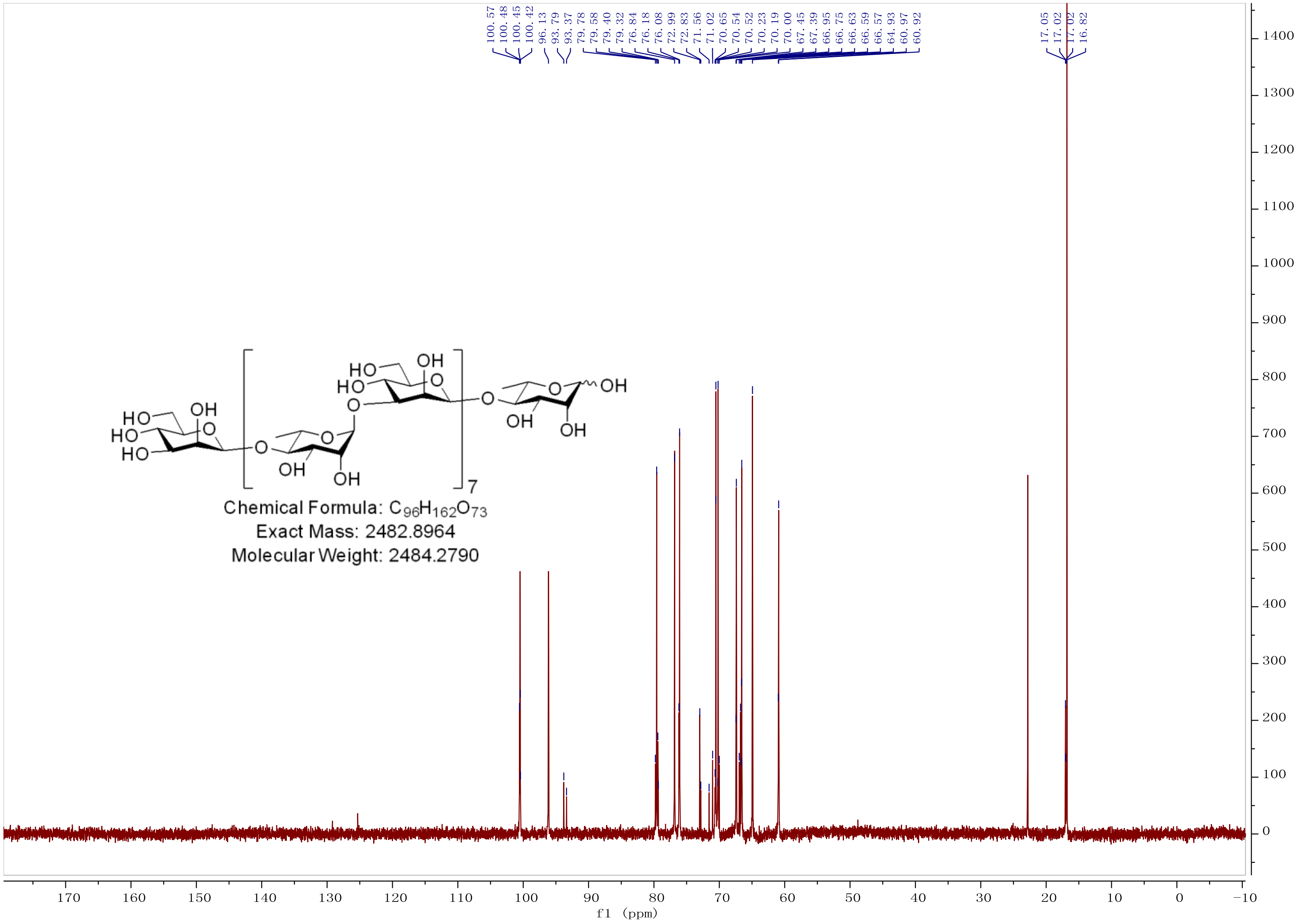

Supplementary Figure 58. ^{13}C-NMR spectrum of 16-mer 4 (D_2O, 25 ^{o}C)

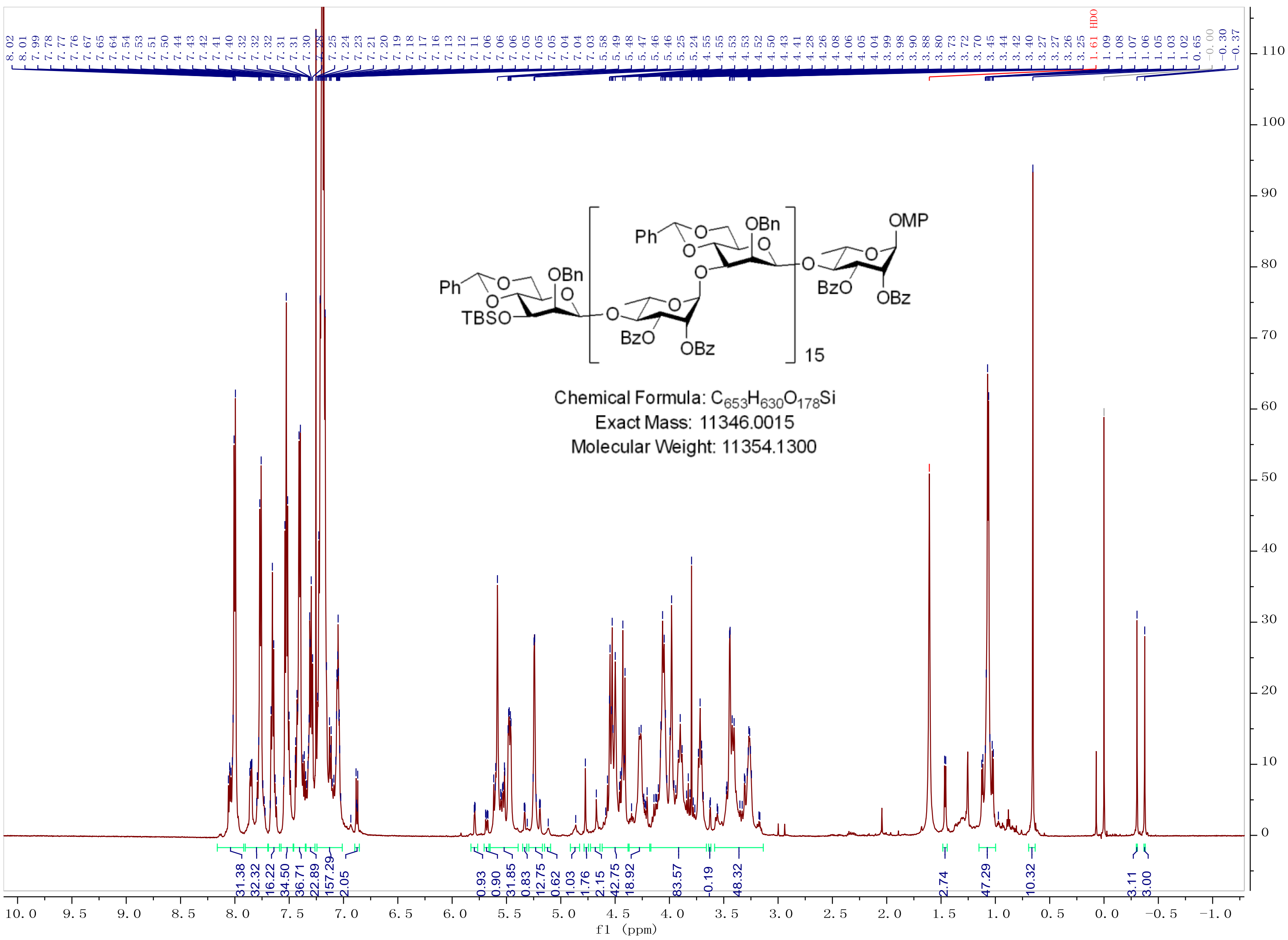

Supplementary Figure 59. ^{1}**H-NMR spectrum of 32-mer 12 (CDCl₃, 25 ºC)**

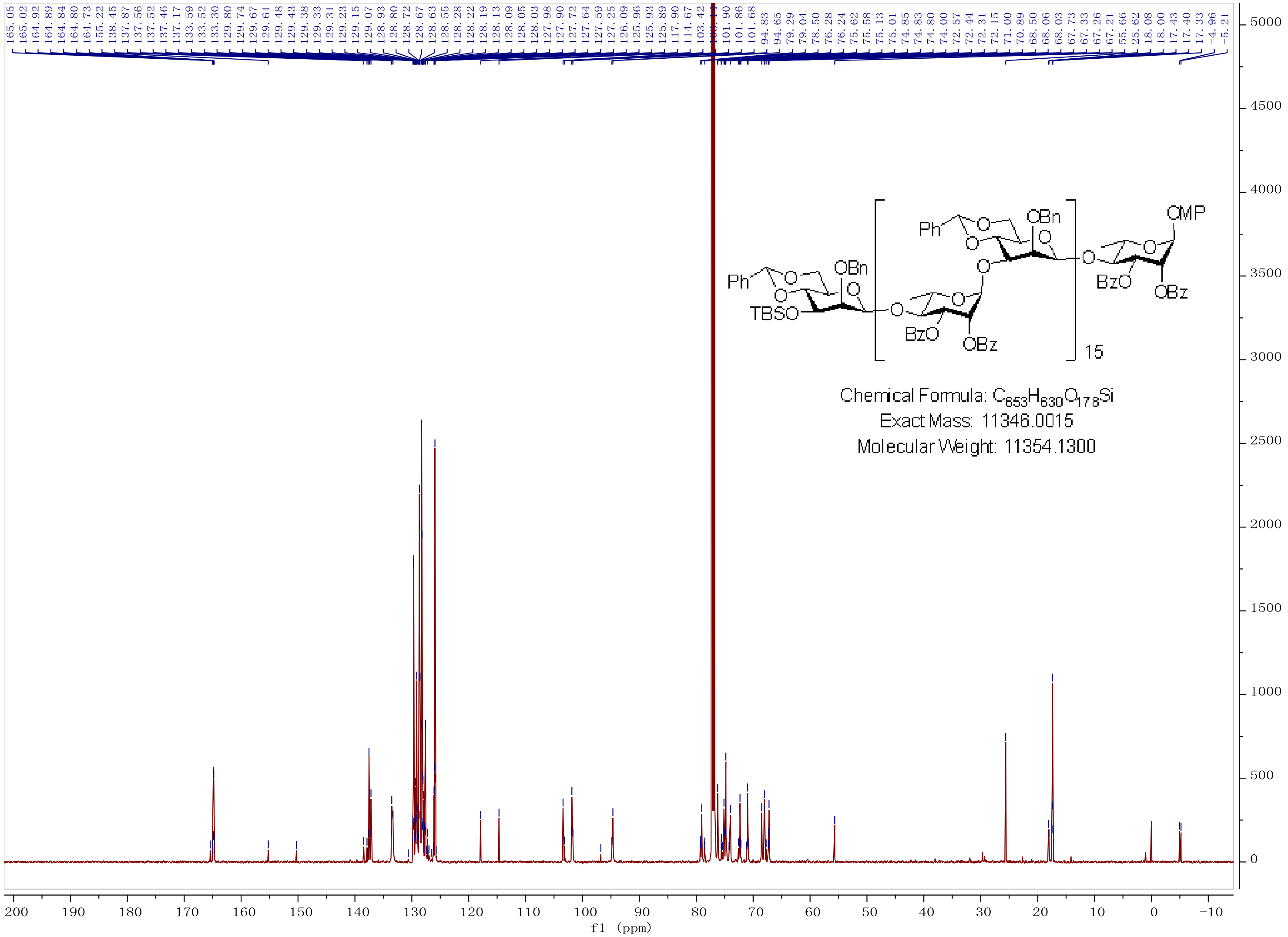

Supplementary Figure 60. ^{13}**C-NMR spectrum of 32-mer 12 (CDCl$_3$, 25 ^{o}C)**

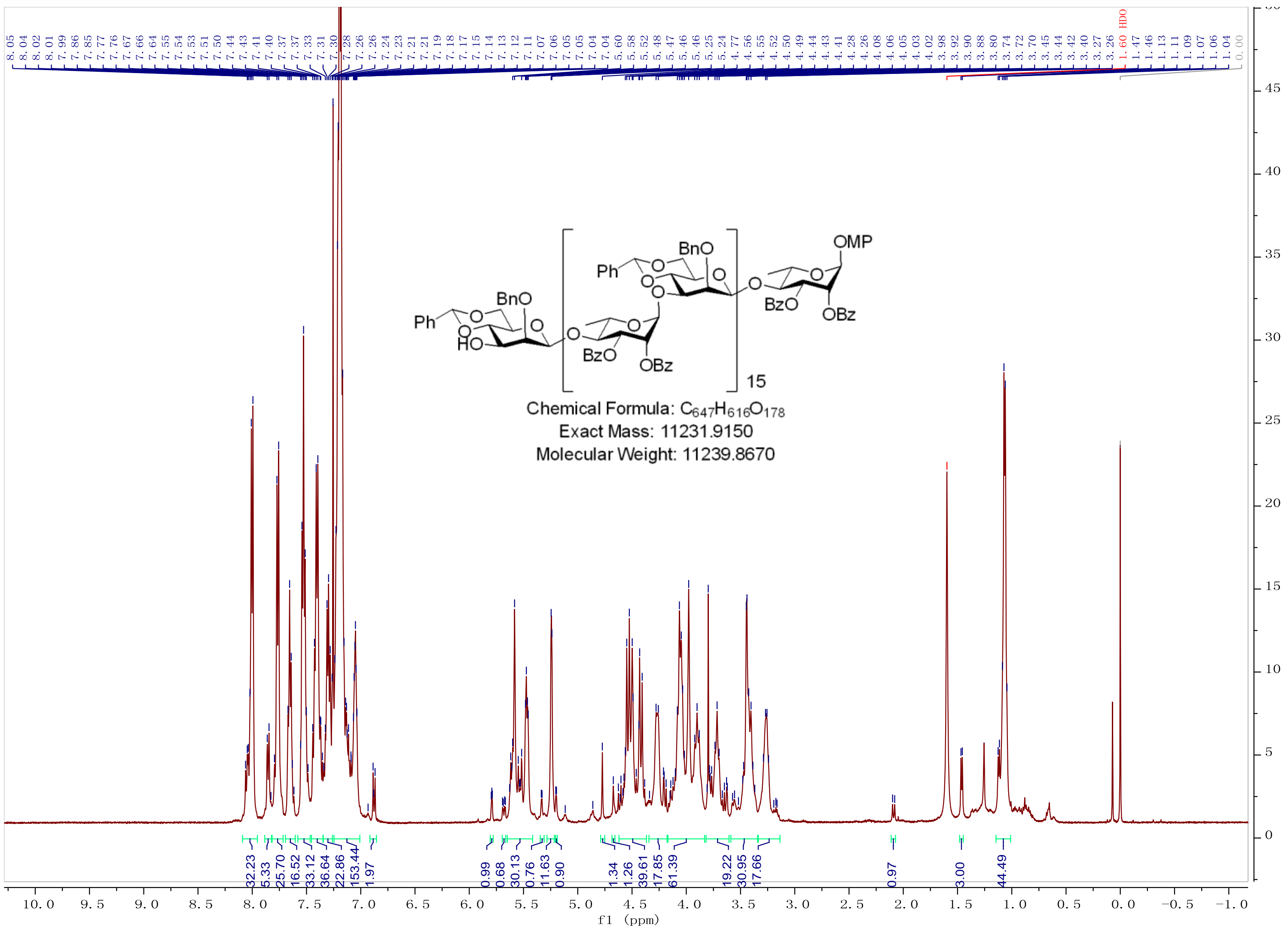

Supplementary Figure 61. ^{1}H-NMR spectrum of compound 12^{A} (CDCl$_3$, 25 °C)

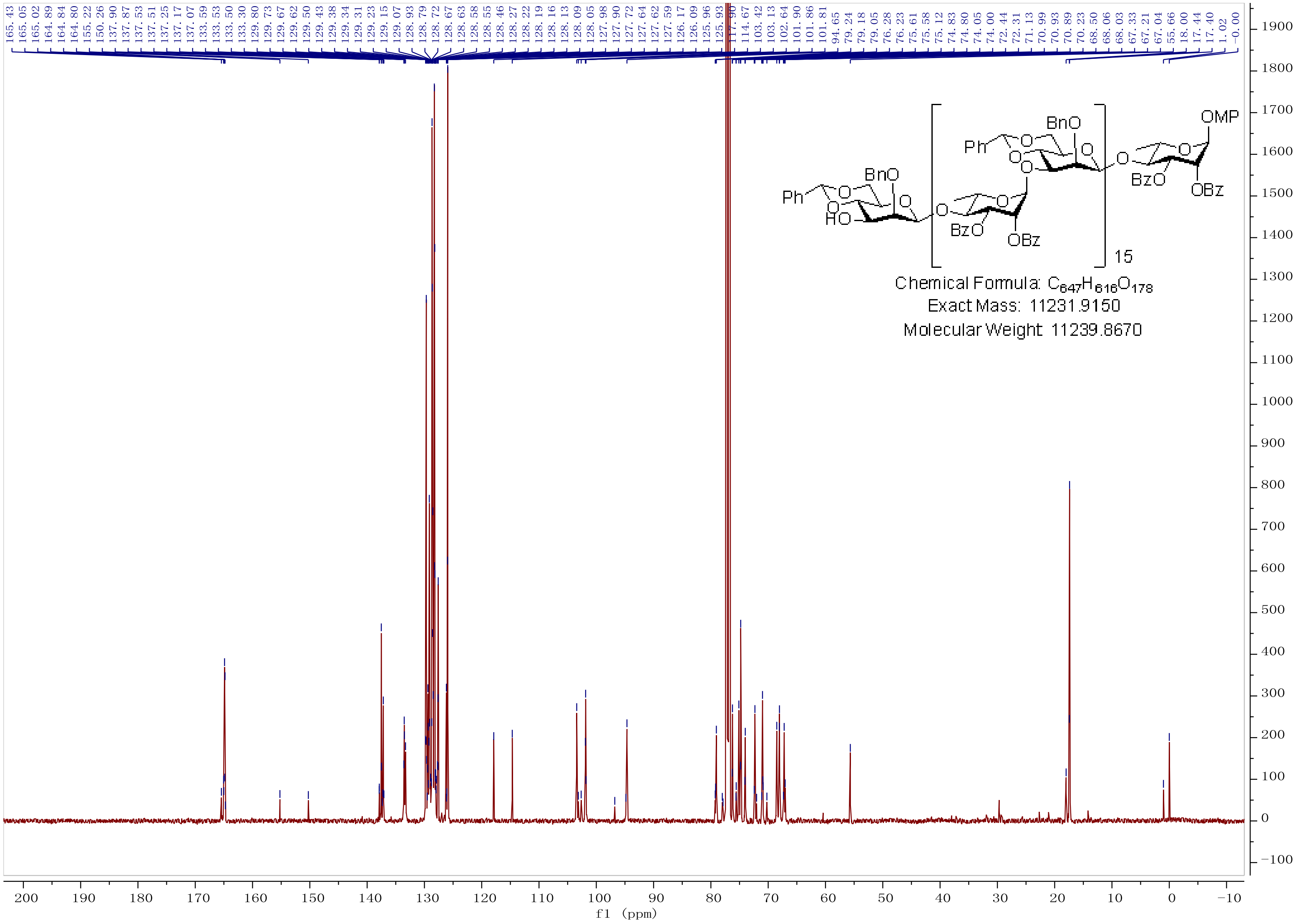

Supplementary Figure 62. ^{13}C-NMR spectrum of compound 12^{A} ($CDCl_3$, 25 °C)

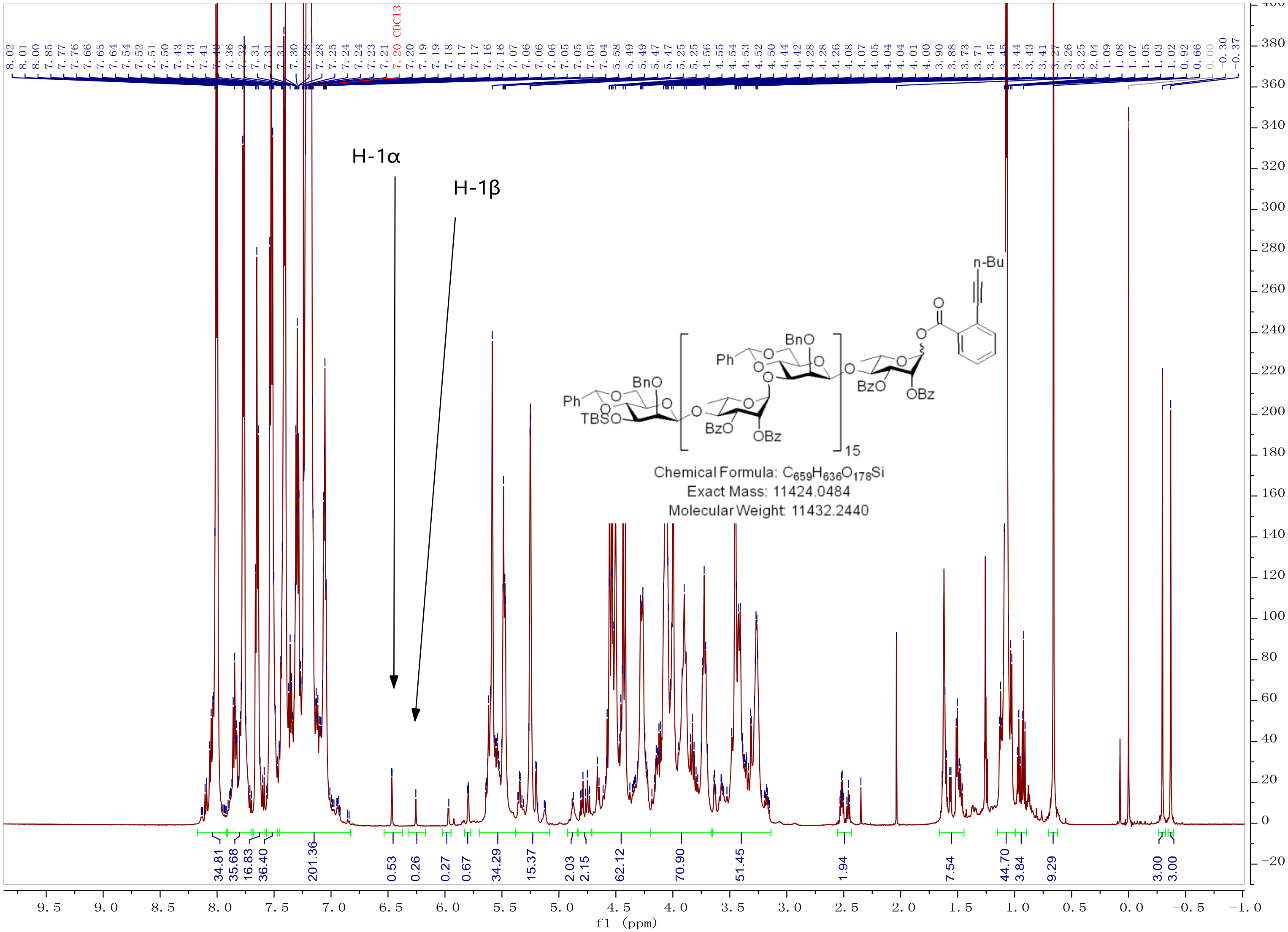

Supplementary Figure 63.^{1}H-NMR spectrum of compound 12[D] ($CDCl_3$, 25 ºC)

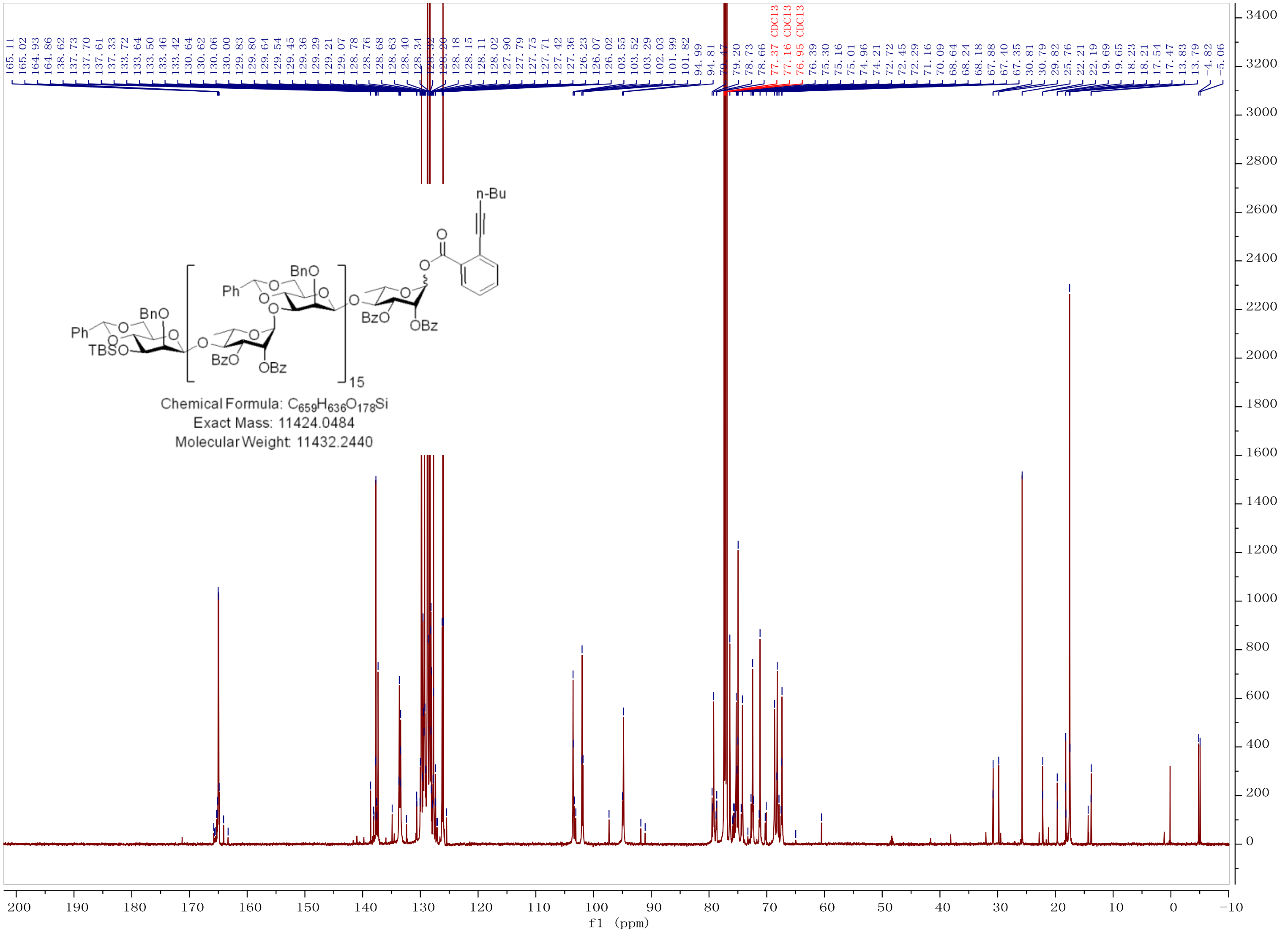

Supplementary Figure 64. ^{13}**C-NMR spectrum of compound 12^{D} ($CDCl_3$, 25 °C)**

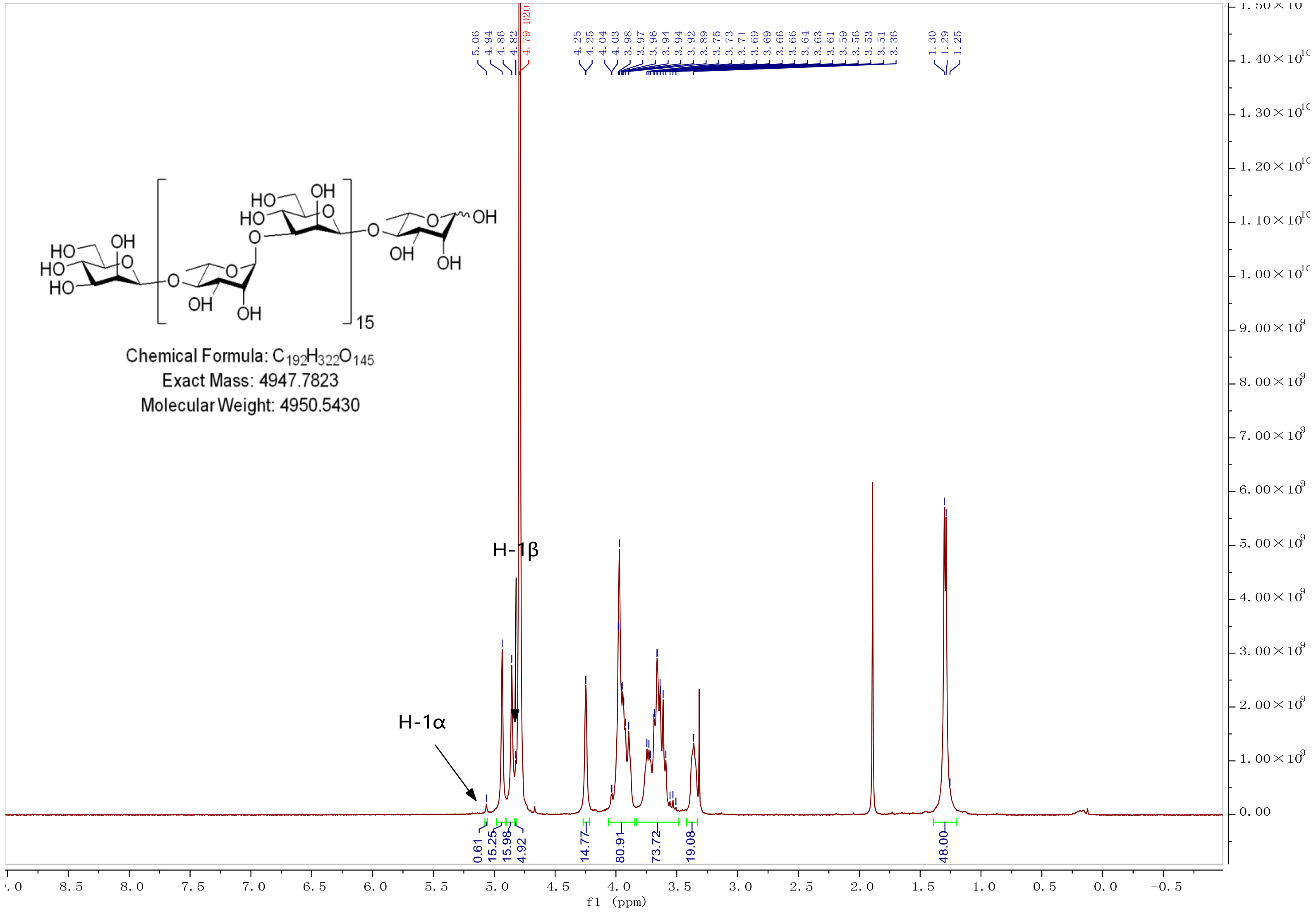

Supplementary Figure 65. ^{1}H-NMR spectrum of 32-mer 5 (D_2O, 25 °C)

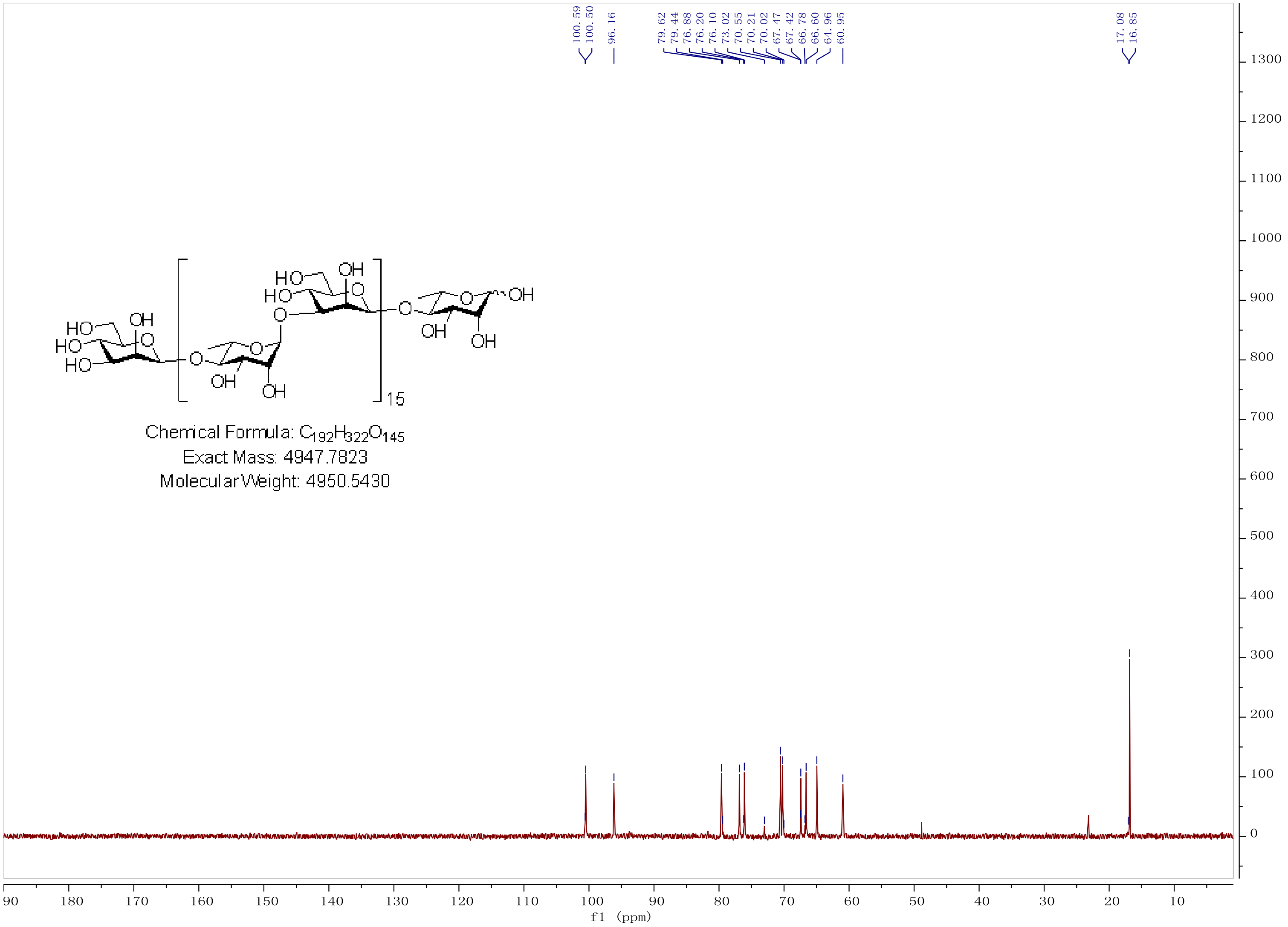

Supplementary Figure 66 .^{13}C-NMR spectrum of 32-mer 5 (D_2O, 25 °C)

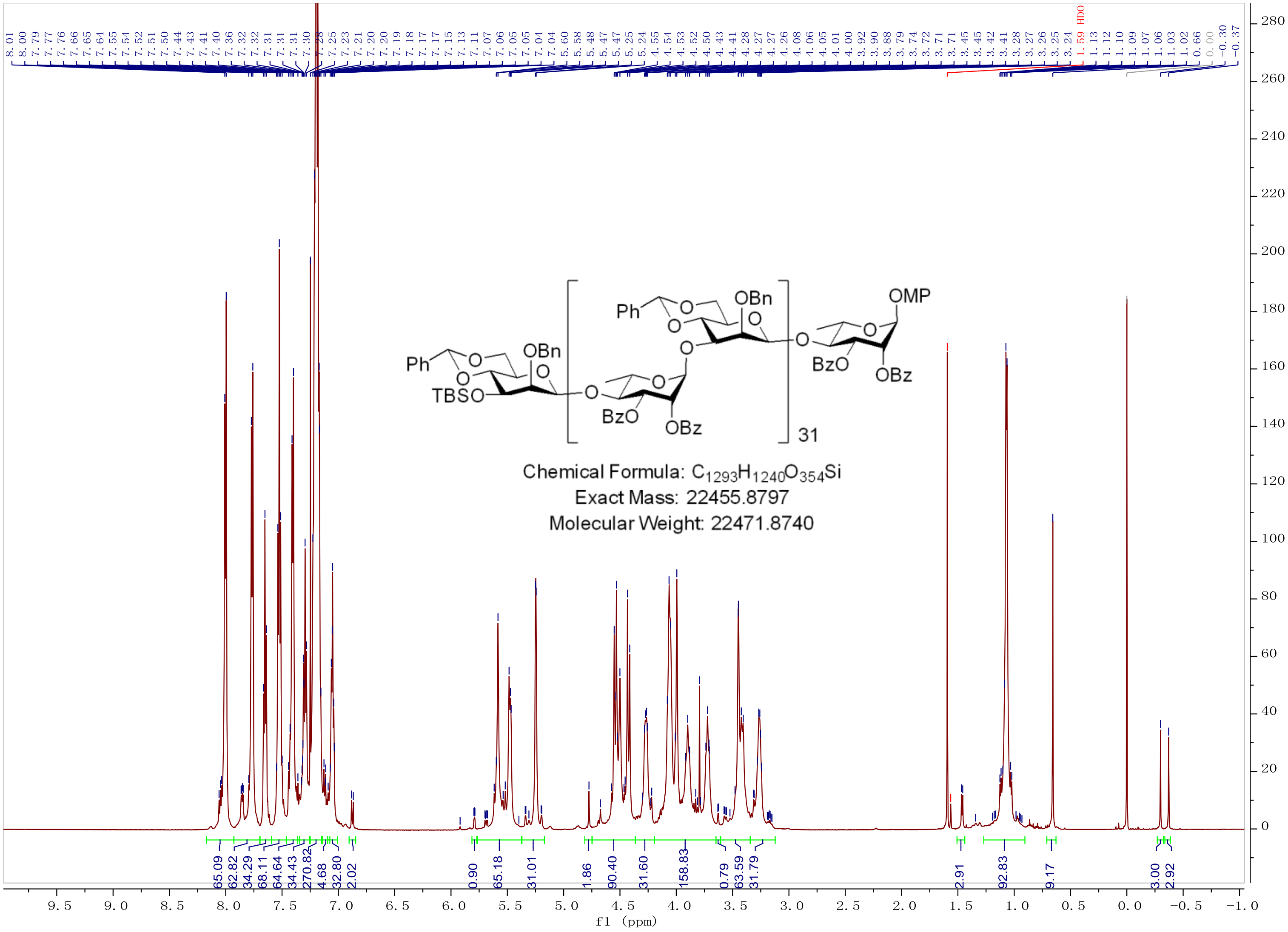

Supplementary Figure 67. ^{1}H-NMR spectrum of 64-mer 13 ($CDCl_3$, 25 ºC)

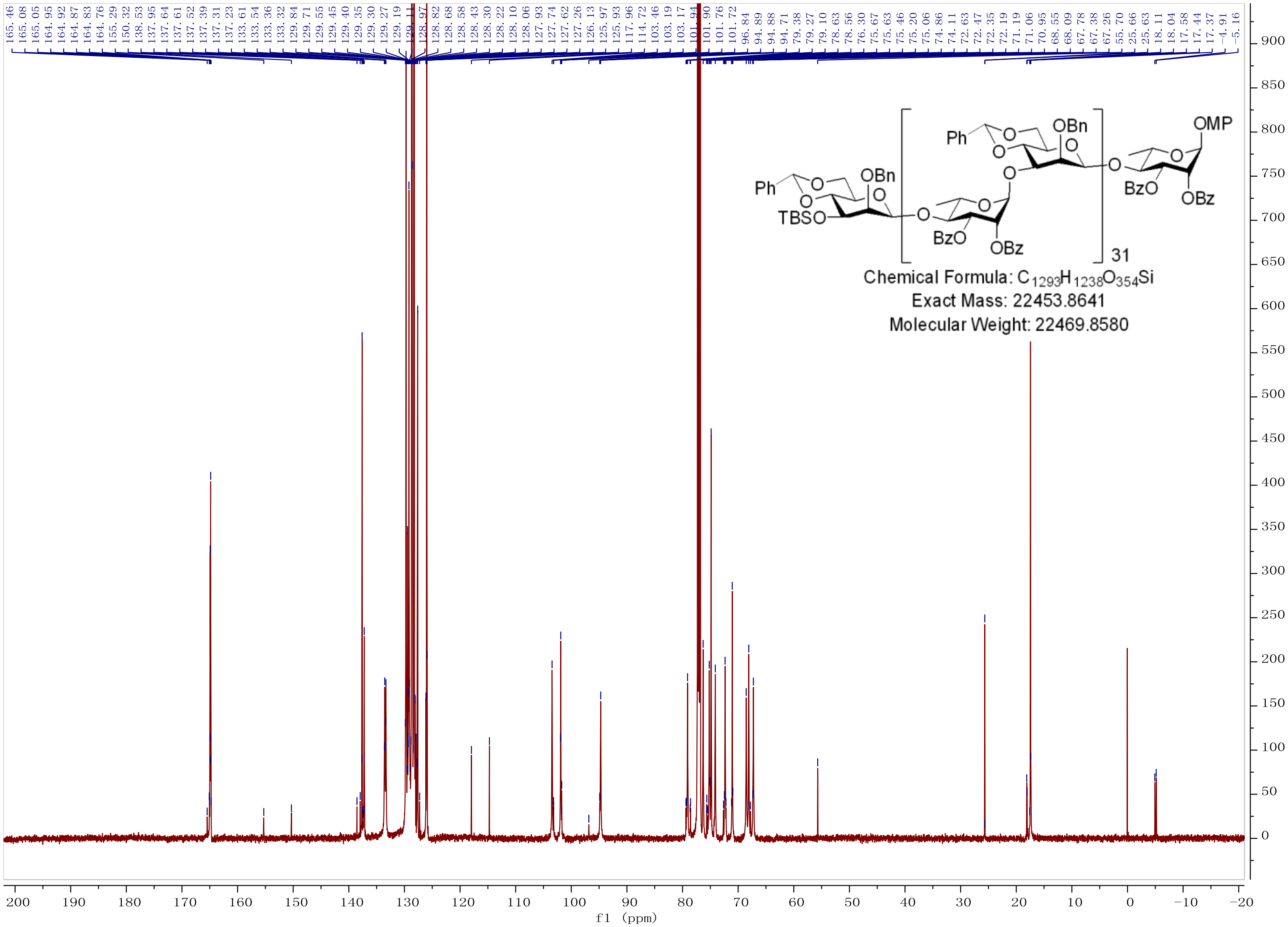

Supplementary Figure 68. ^{13}**C-NMR spectrum of 64-mer 13 (CDCl$_3$, 25 ^{o}C)**

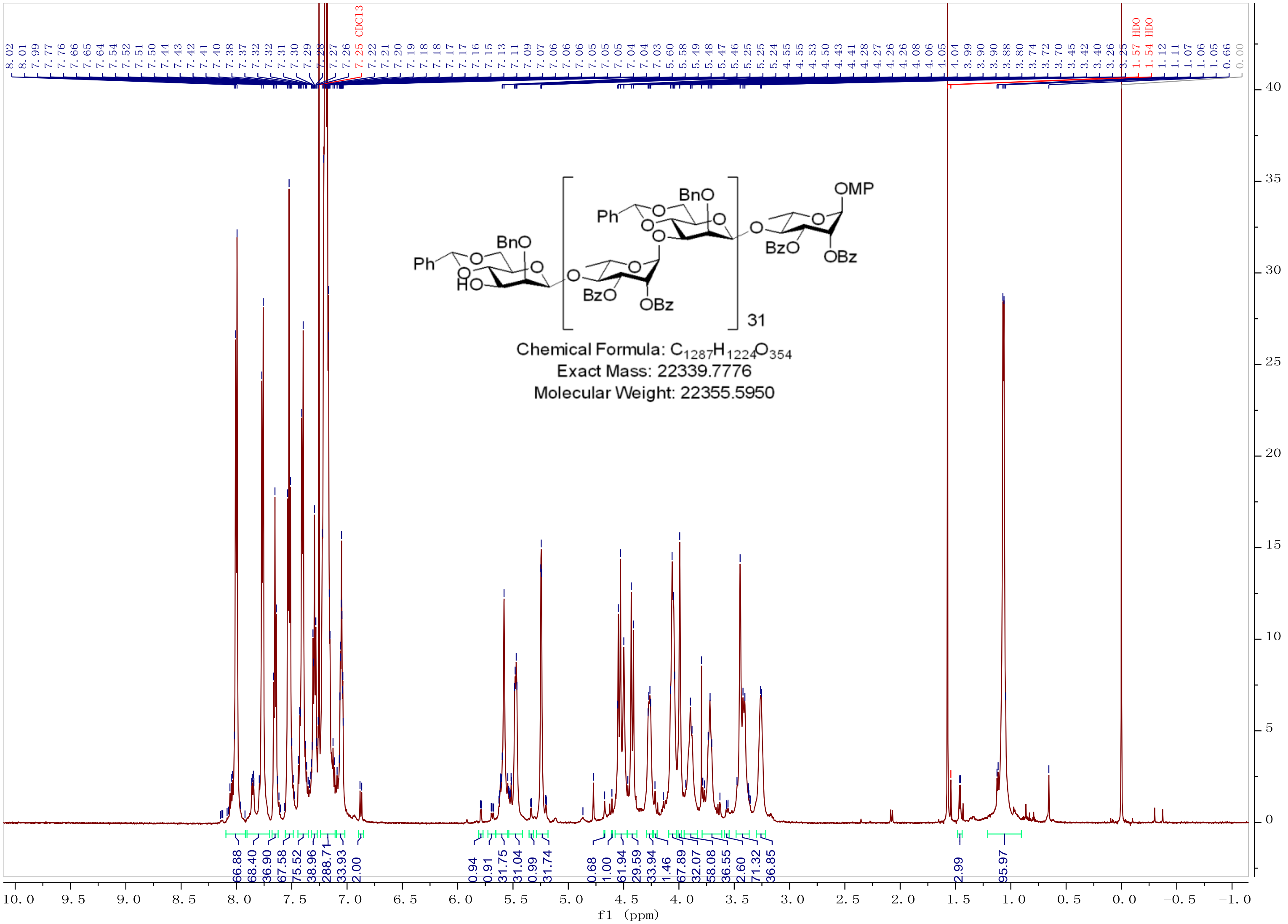

Supplementary Figure 69. ^{1}H-NMR spectrum of compound 13^{A} ($CDCl_3$, 25 °C)

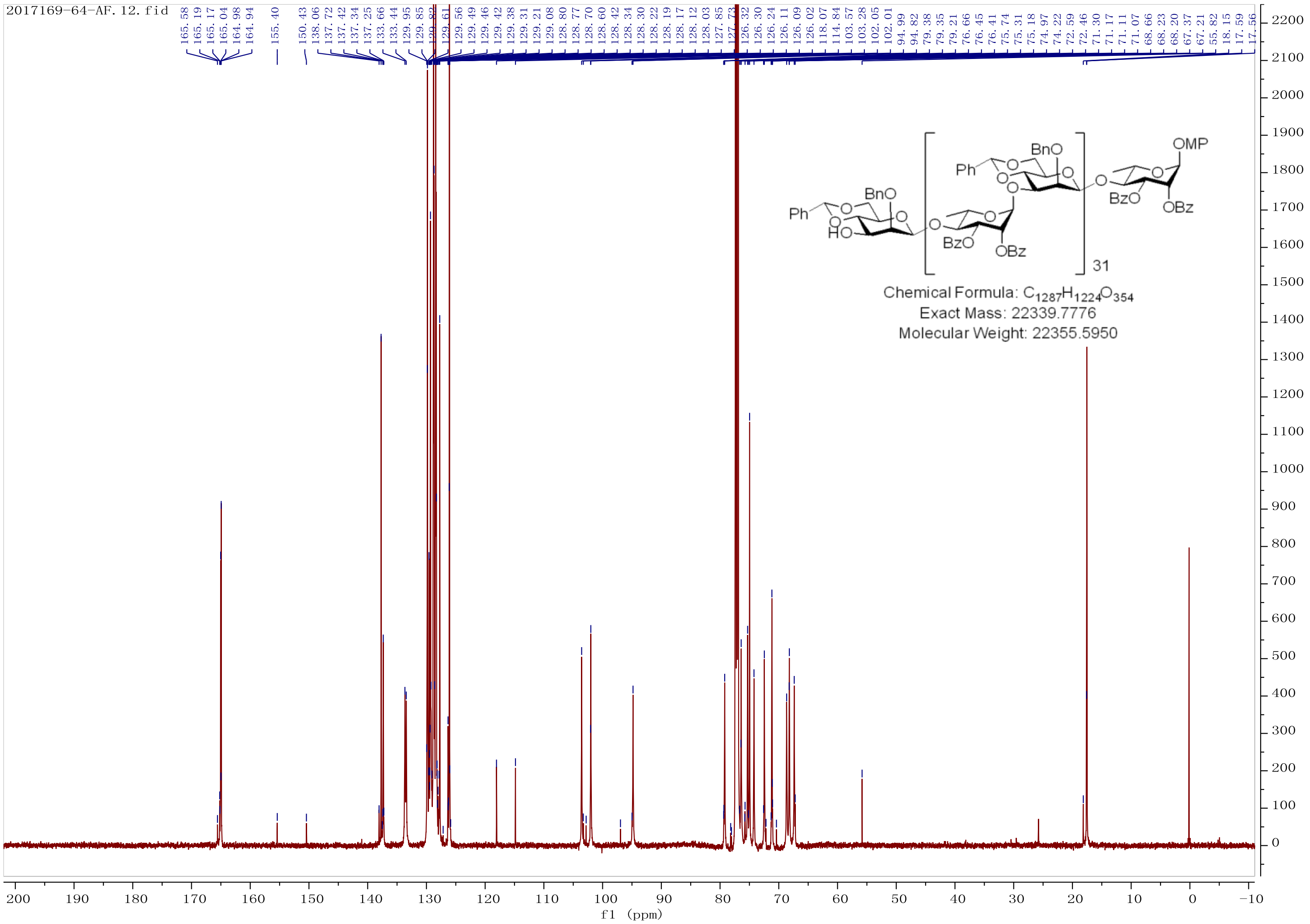

Supplementary Figure 70. ^{13}C-NMR spectrum of compound 13^{A} ($CDCl_3$, 25 ^{o}C)

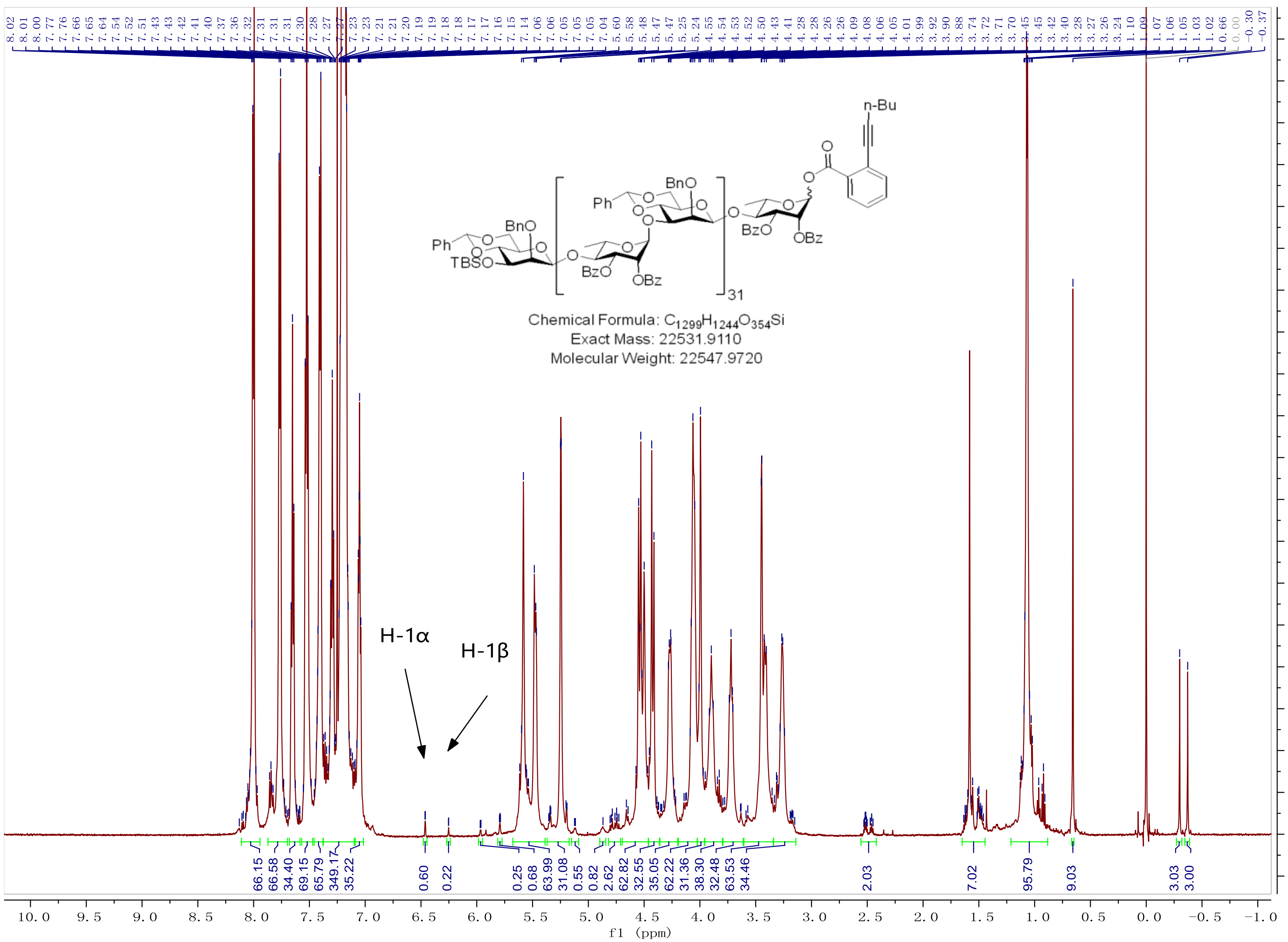

Supplementary Figure 71. ^{1}H-NMR spectrum of compound 13^D ($CDCl_3$, 25 ^{o}C)

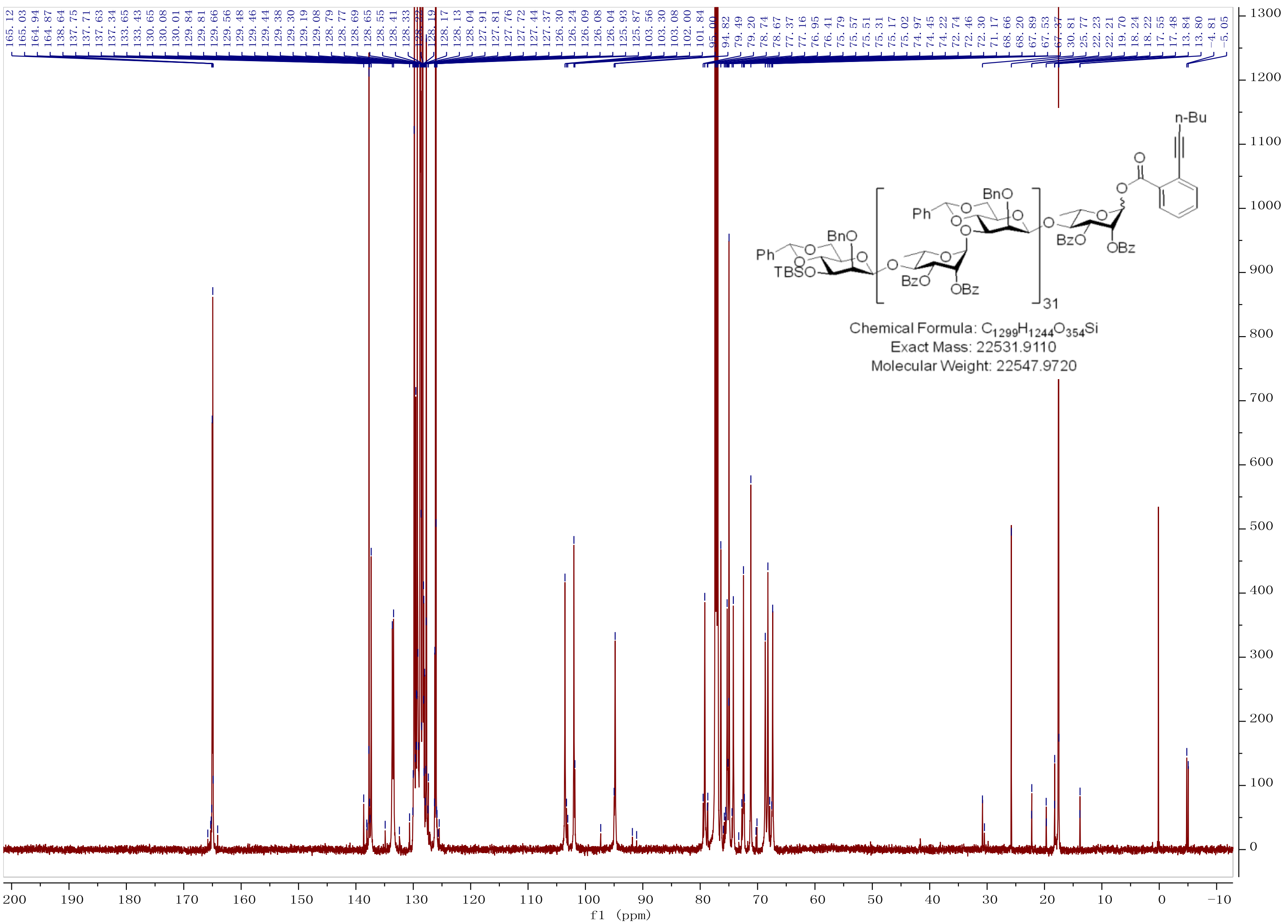

Supplementary Figure 72. ^{13}C-NMR spectrum of compound 13^{D} ($CDCl_3$, 25 °C)

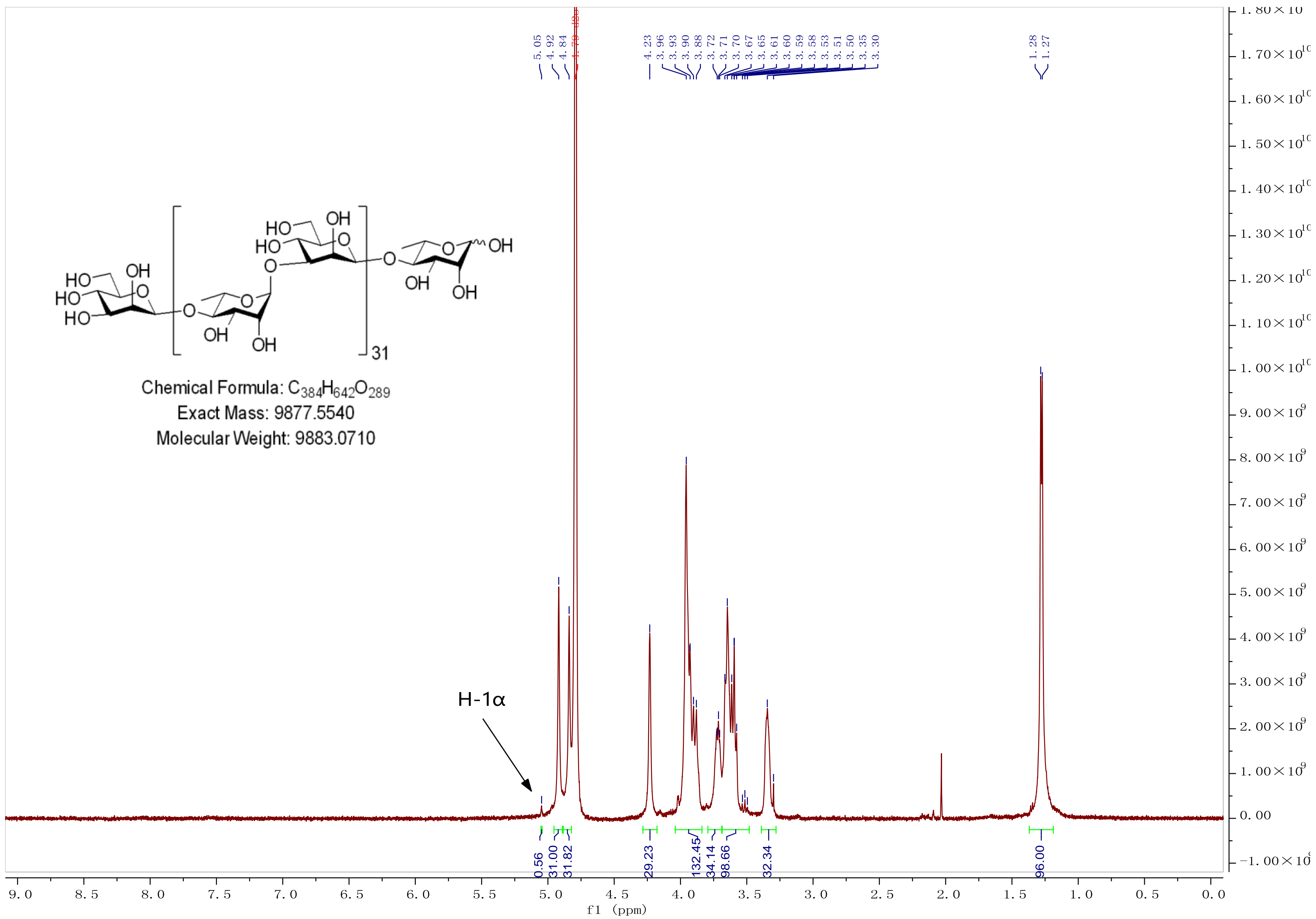

Supplementary Figure 73. ^{1}H-NMR spectrum of 64-mer 6 (D_2O, 25 ^{o}C)

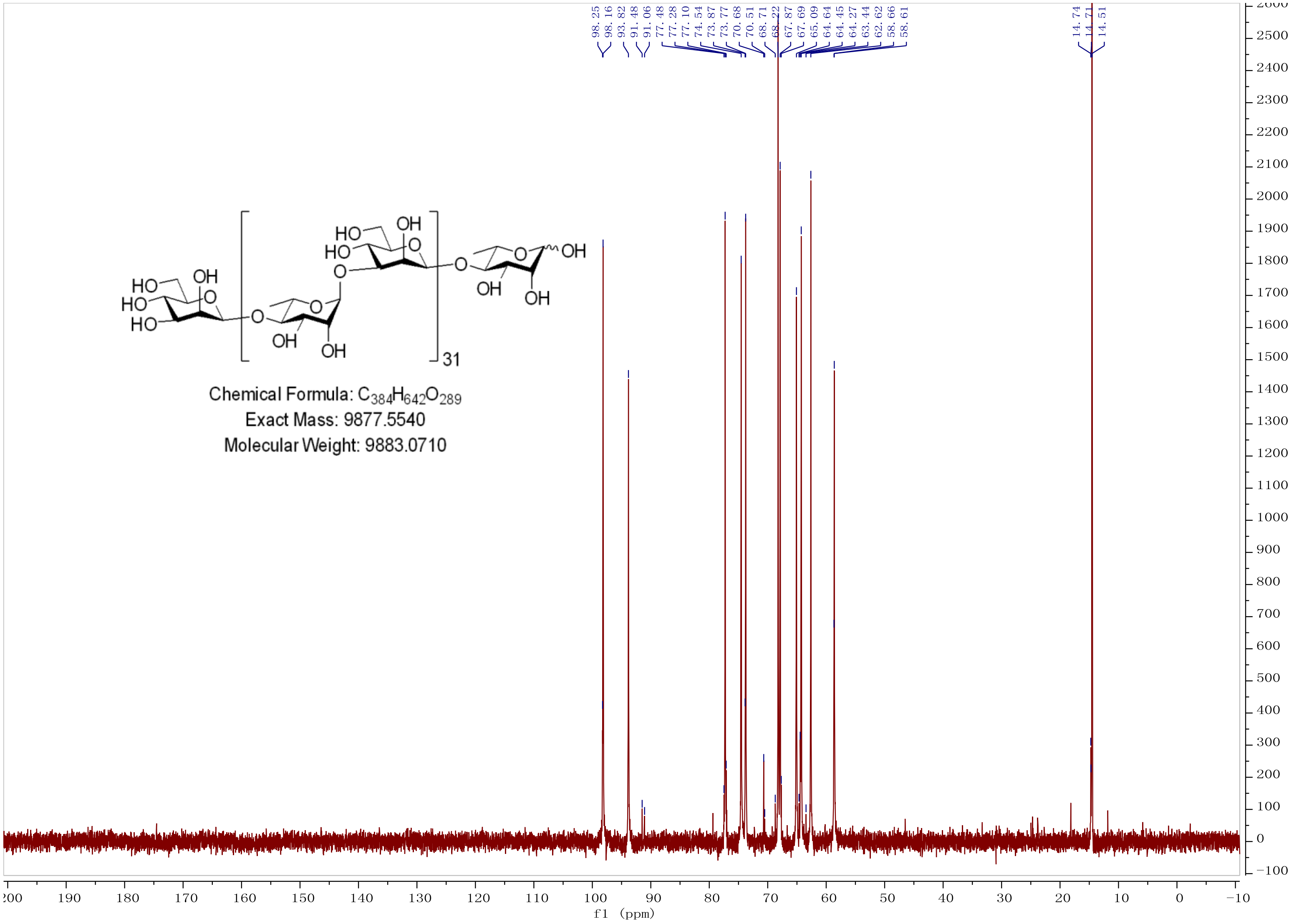

Supplementary Figure 74. ^{13}C-NMR spectrum of 64-mer 6 (D_2O, 25 °C)

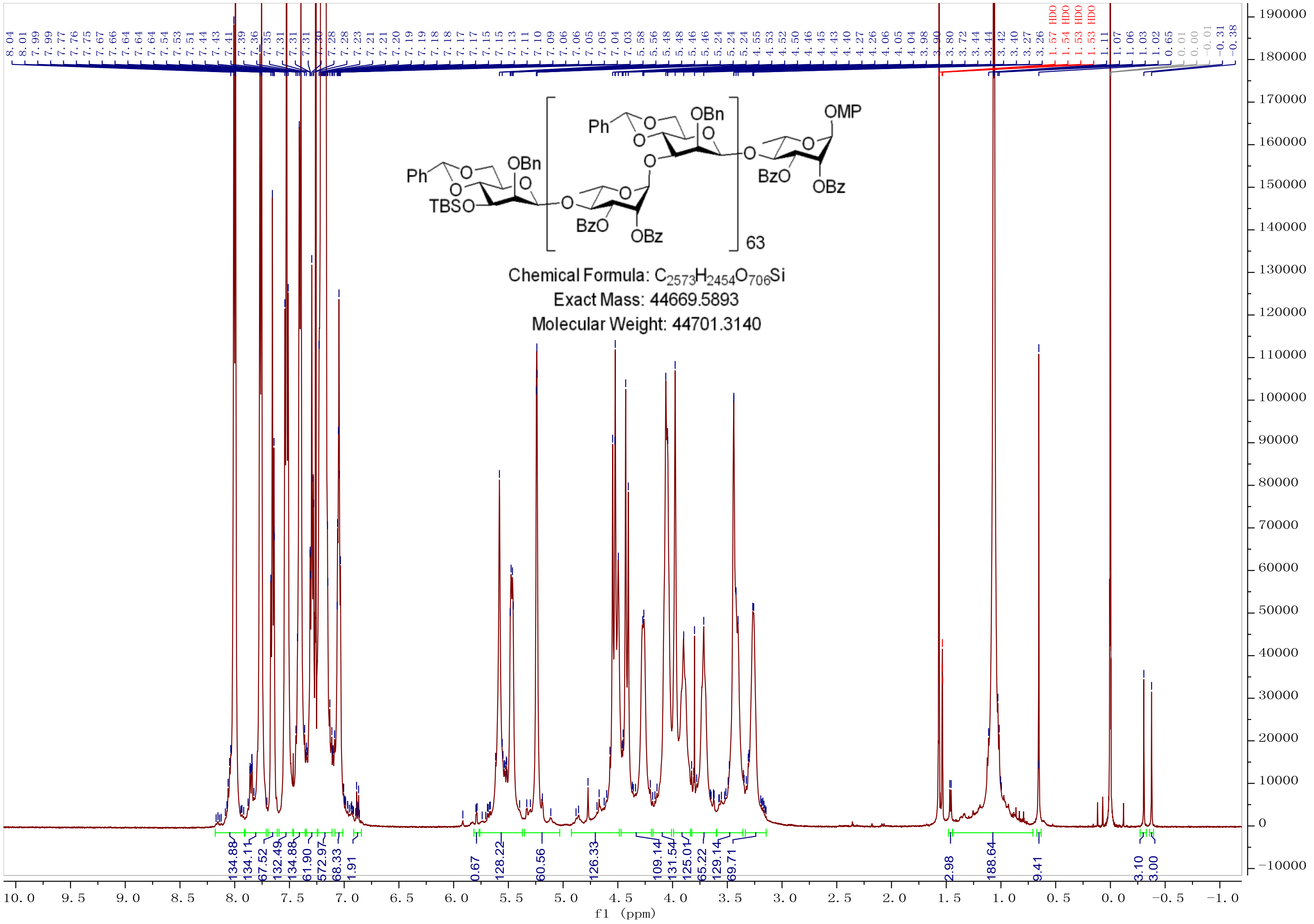

Supplementary Figure 75. ^{1}**H-NMR spectrum of 128-mer 14 ($CDCl_3$, 25 °C)-**

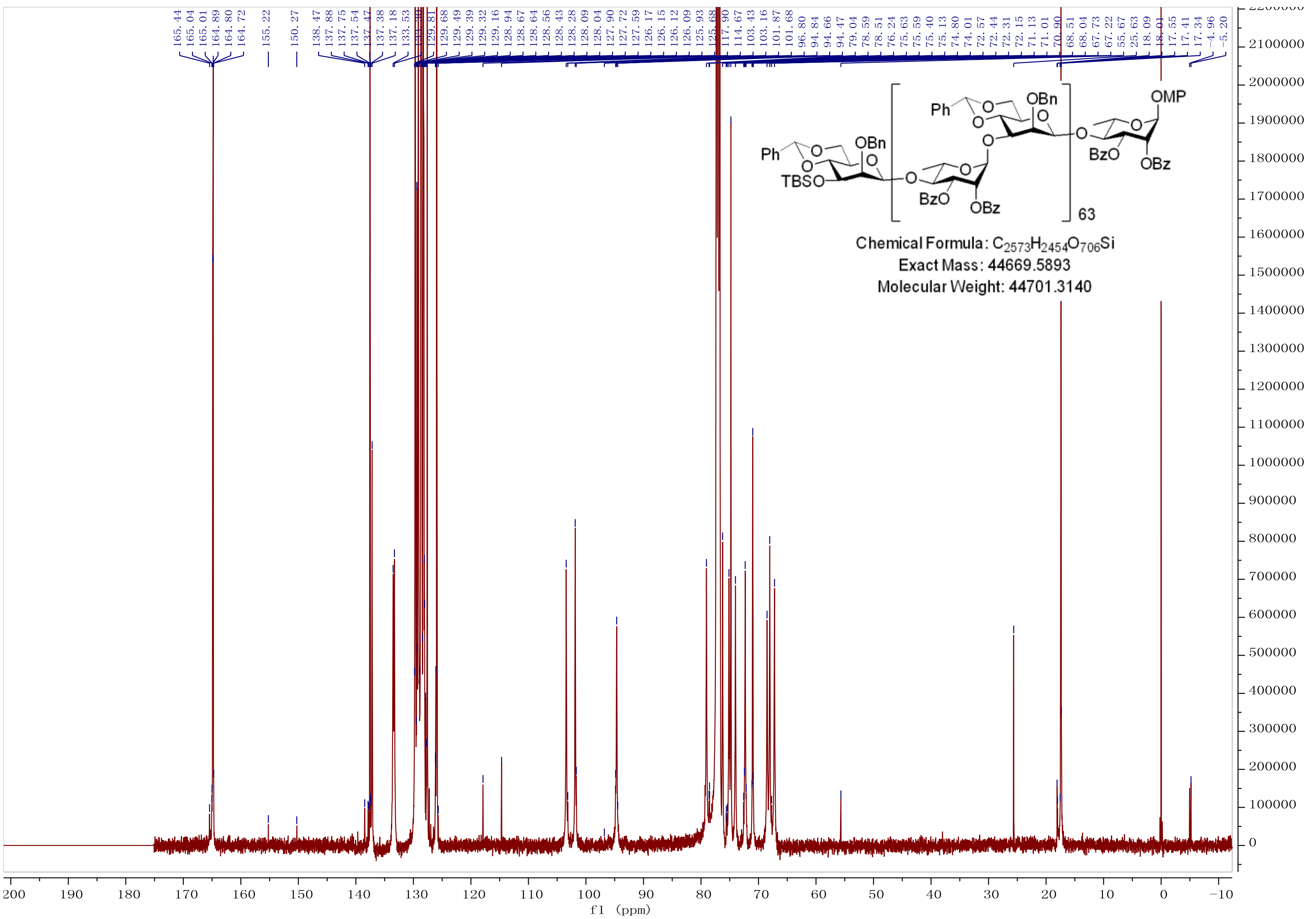

Supplementary Figure 76. ^{13}C-NMR spectrum of 128-mer 14 ($CDCl_3$, 25 °C)

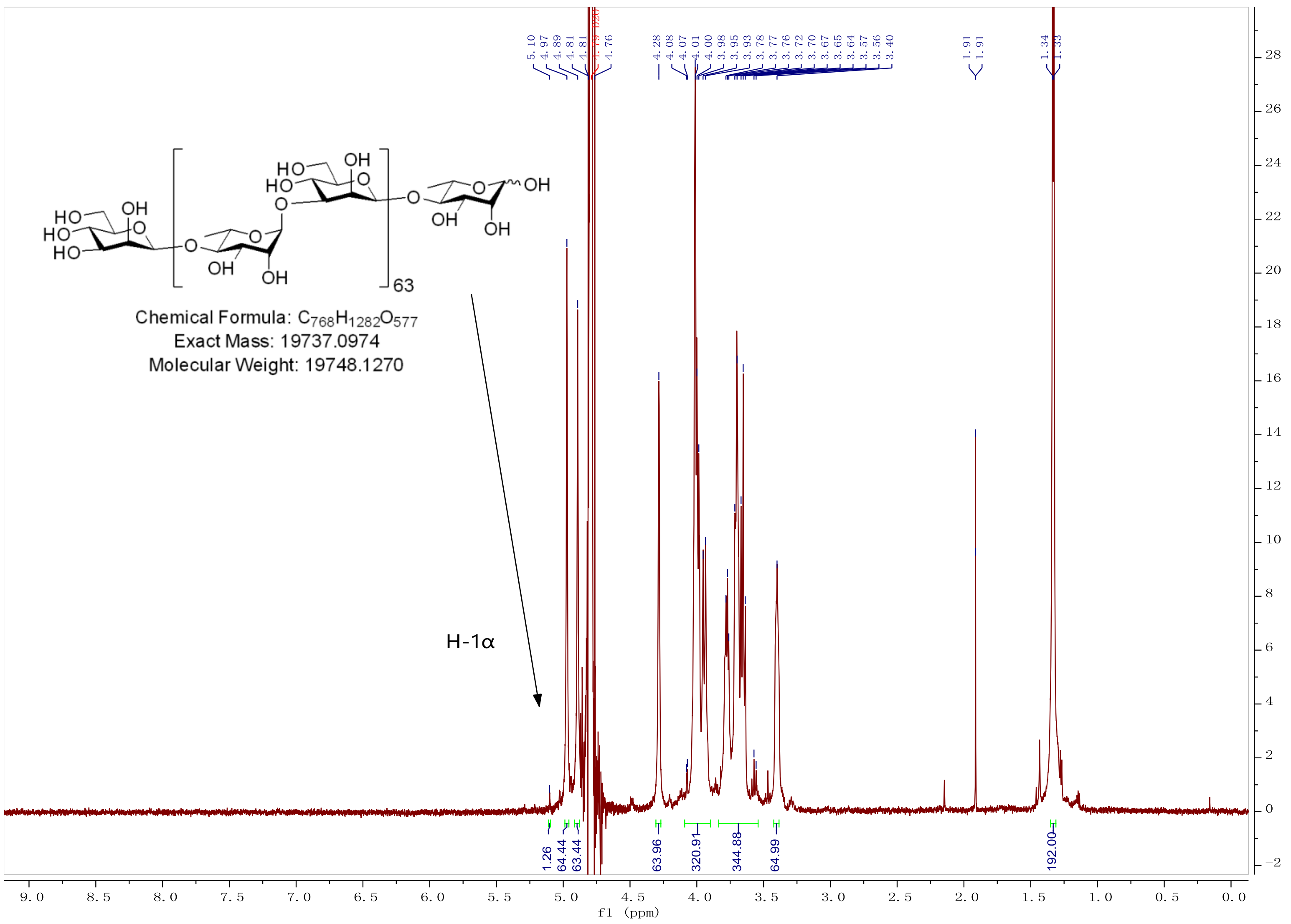

Supplementary Figure 77. **^{1}H-NMR spectrum of 128-mer 7 (D_2O, 25 °C)**

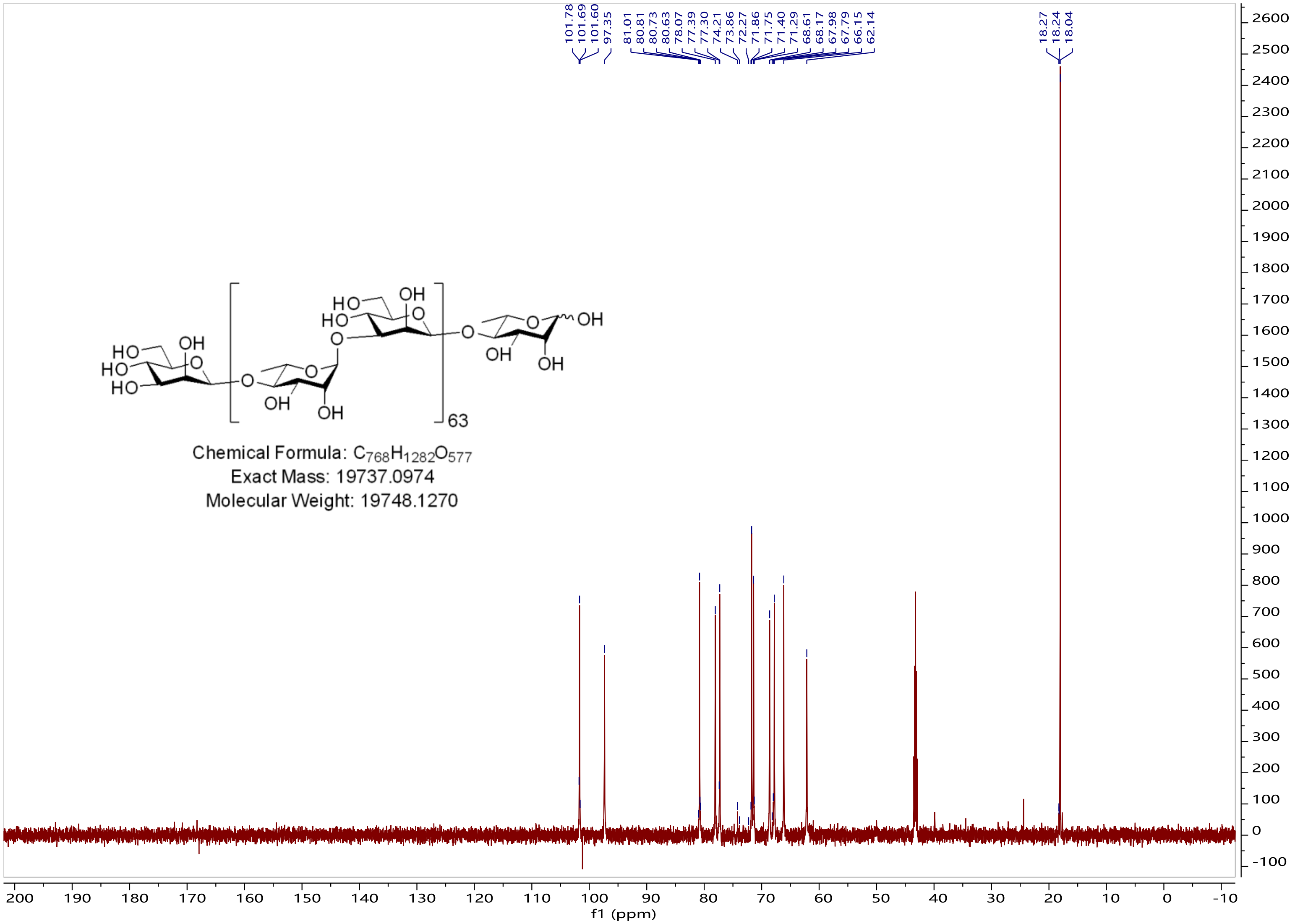

Supplementary Figure 78. ^{13}**C-NMR spectrum of 128-mer 7 (D$_2$O, 25 ^{o}C)**

Data: yb20190228sample2-27_0001:N3 (Manual) Thursday, February 28, 2019 1:39:30 PM Cal:Custom Calibration by Engineer on Thursday, February 28, 2019 1:40:57 PM
Shimadzu MALDI-7090: Tuning Linear, Power 103, P.Ext at 10000.00 (bin 344), Ion Gate Blanking: 1000.00, Laser Diameter: 100

Processed data (averaged) : 0.7 mV [sum=47.0 mV], Smoothed = 3, profiles # 1 - 65

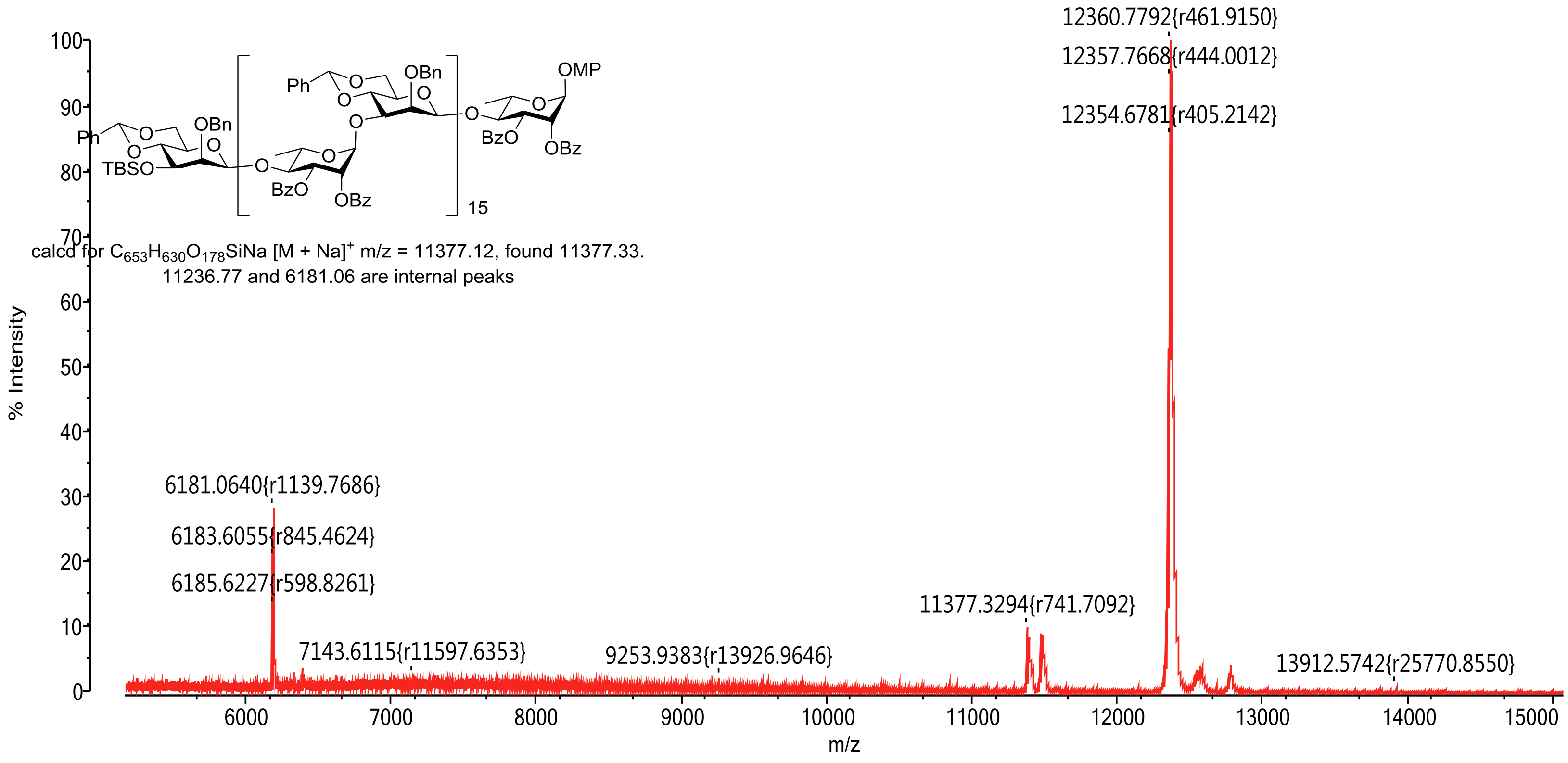

Supplementary Figure 79. MALDI-TOF MS spectrum of 32-mer 12

Data: yb20190228sample2-70_0001:O2 (Manual) Thursday, February 28, 2019 2:07:33 PM Cal:Custom Calibration by Engineer on Thursday, February 28, 2019 2:08:35 PM
Shimadzu MALDI-7090: Tuning Linear, Power 120, P.Ext at 10000.00 (bin 344), Ion Gate Blanking: 1000.00, Laser Diameter: 100

Processed data (averaged) : 0.4 mV [sum=26.3 mV], Smoothed = 3, profiles # 1 - 67

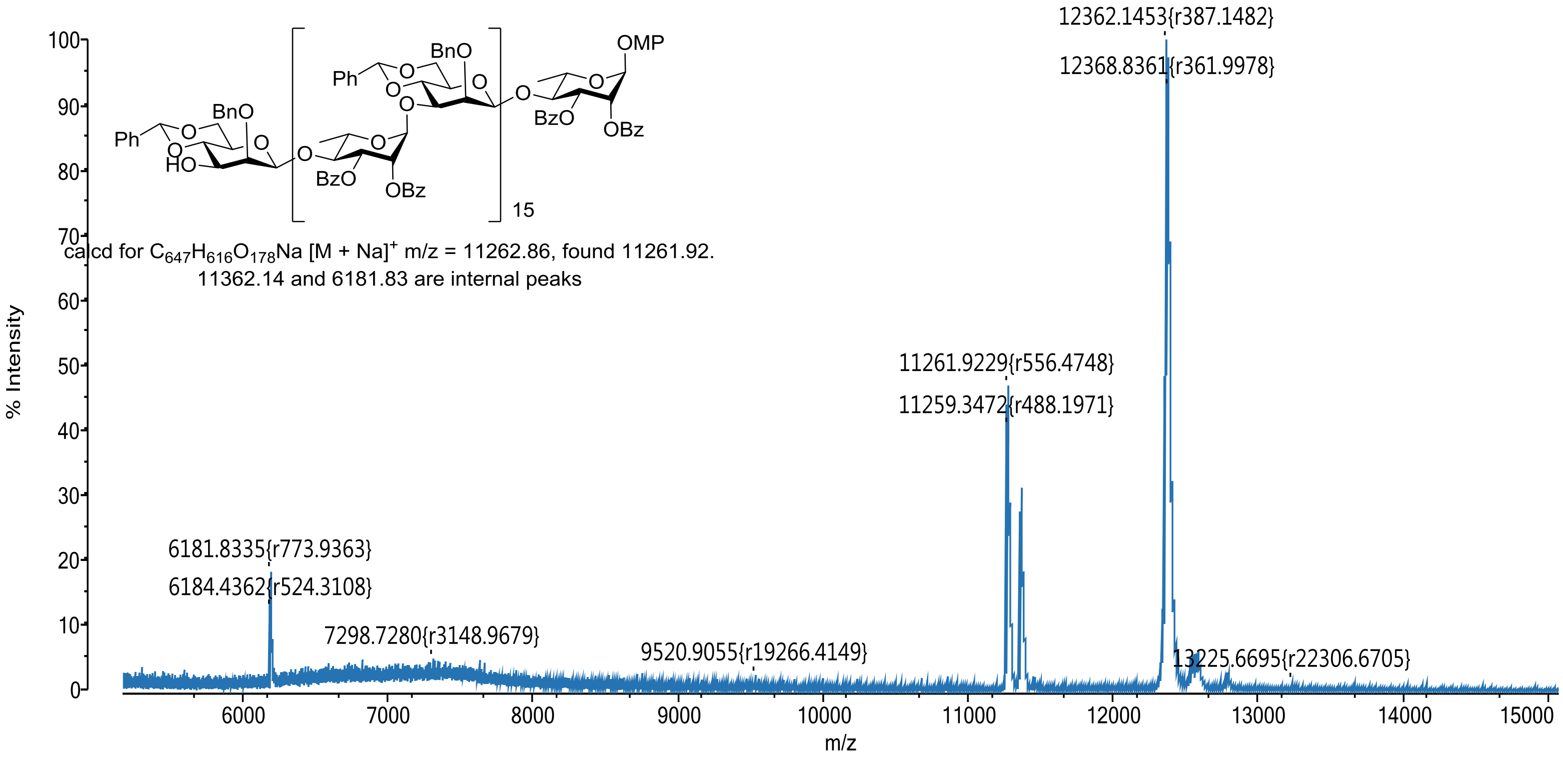

Supplementary Figure 80. MALDI-TOF MS spectrum of compound 12^A

Data: sioc20190408sample2-73_0004:A11 (Manual) Monday, April 08, 2019 3:18:48 PM Cal:Custom Calibration by Engineer on Monday, April 08, 2019 3:37:25 PM
Shimadzu MALDI-7090: Tuning Linear, Power 152, P.Ext at 10000.00 (bin 344), Ion Gate Blanking: 1000.00, Laser Diameter: 300

Processed data (averaged) : 0.0 mV [sum=2.2 mV], Smoothed = 30, profiles # 1 - 100

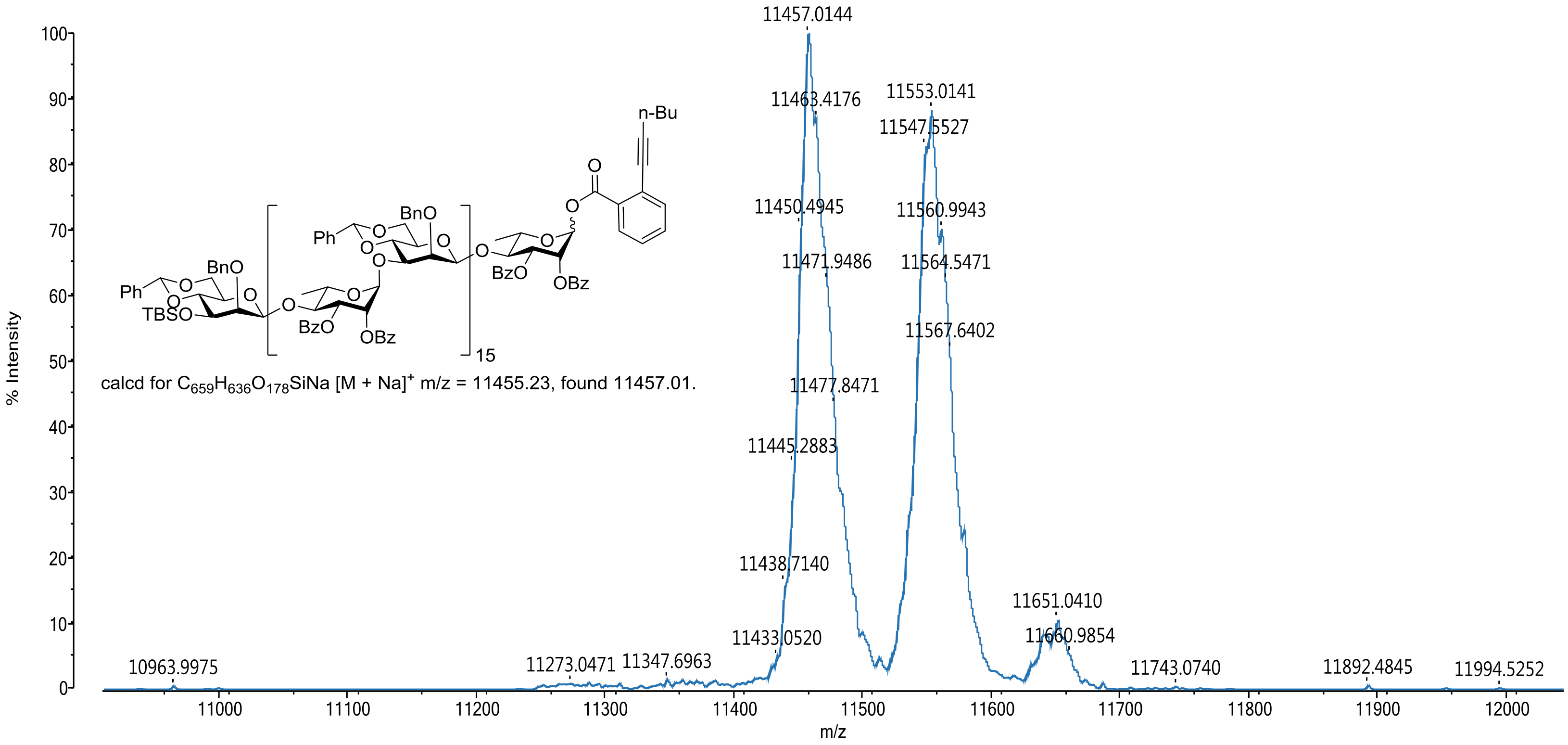

Supplementary Figure 81. MALDI-TOF MS spectrum of compound 12^D

Data: YB20190306SAMPLE2-74_0001:O23 (Manual) Wednesday, March 06, 2019 1:54:33 PM Cal:Custom Calibration by Engineer on Wednesday, March 06, 2019 2:10:01...
Shimadzu MALDI-7090: Tuning Linear, Power 163, P.Ext at 20000.00 (bin 491), Ion Gate Blanking: 1000.00, Laser Diameter: 300
Processed data (averaged) : 0.0 mV [sum=0.1 mV], Smoothed = 25, profiles # 1 - 100

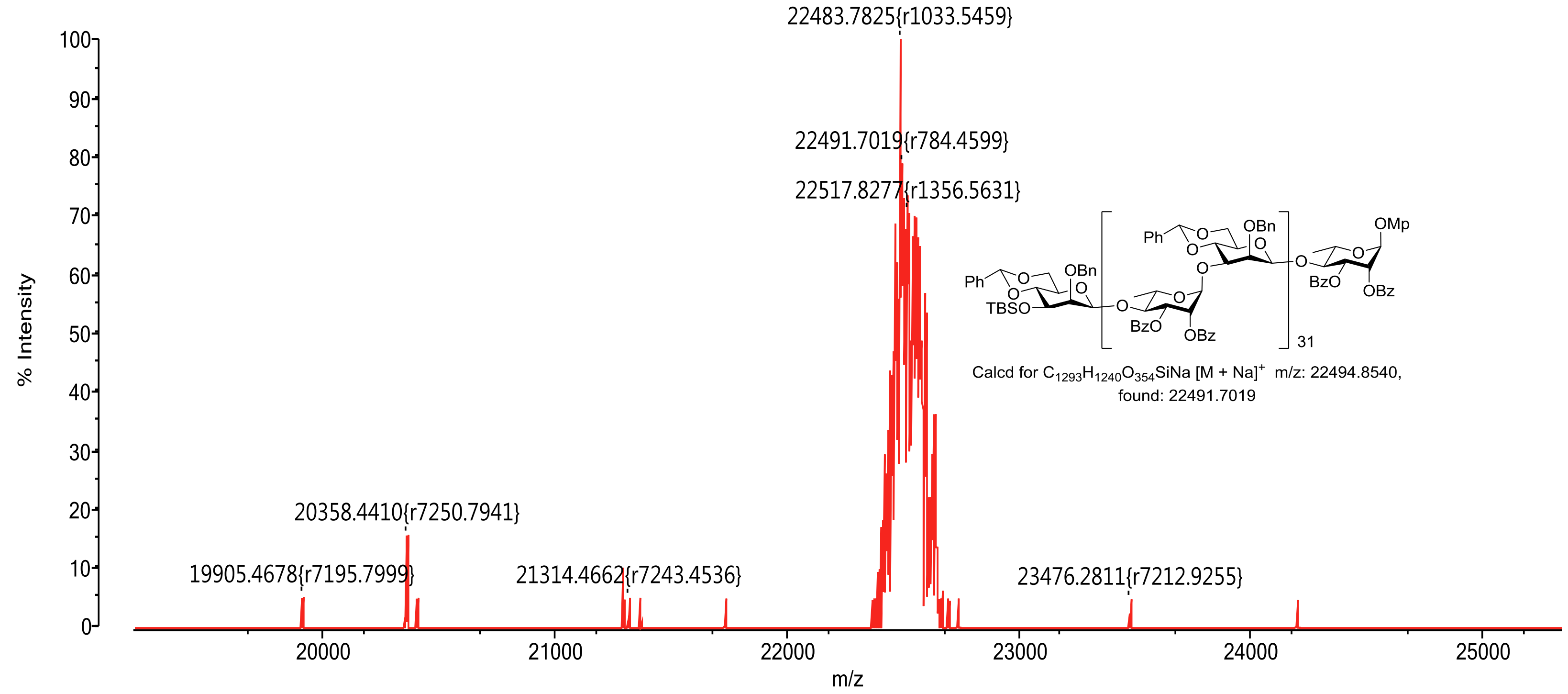

Supplementary Figure 82. MALDI-TOF MS spectrum of 64-mer 13

Data: YB20190306SAMPLE2-80_0001:O17 (Manual) Wednesday, March 06, 2019 2:16:04 PM Cal:Custom Calibration by Engineer on Wednesday, March 06, 2019 2:19:23...
Shimadzu MALDI-7090: Tuning Linear, Power 164, P.Ext at 20000.00 (bin 487), Ion Gate Blanking: 1000.00, Laser Diameter: 300

Processed data (averaged) : 0.0 mV [sum=0.5 mV], Smoothed = 5, profiles # 1 - 100

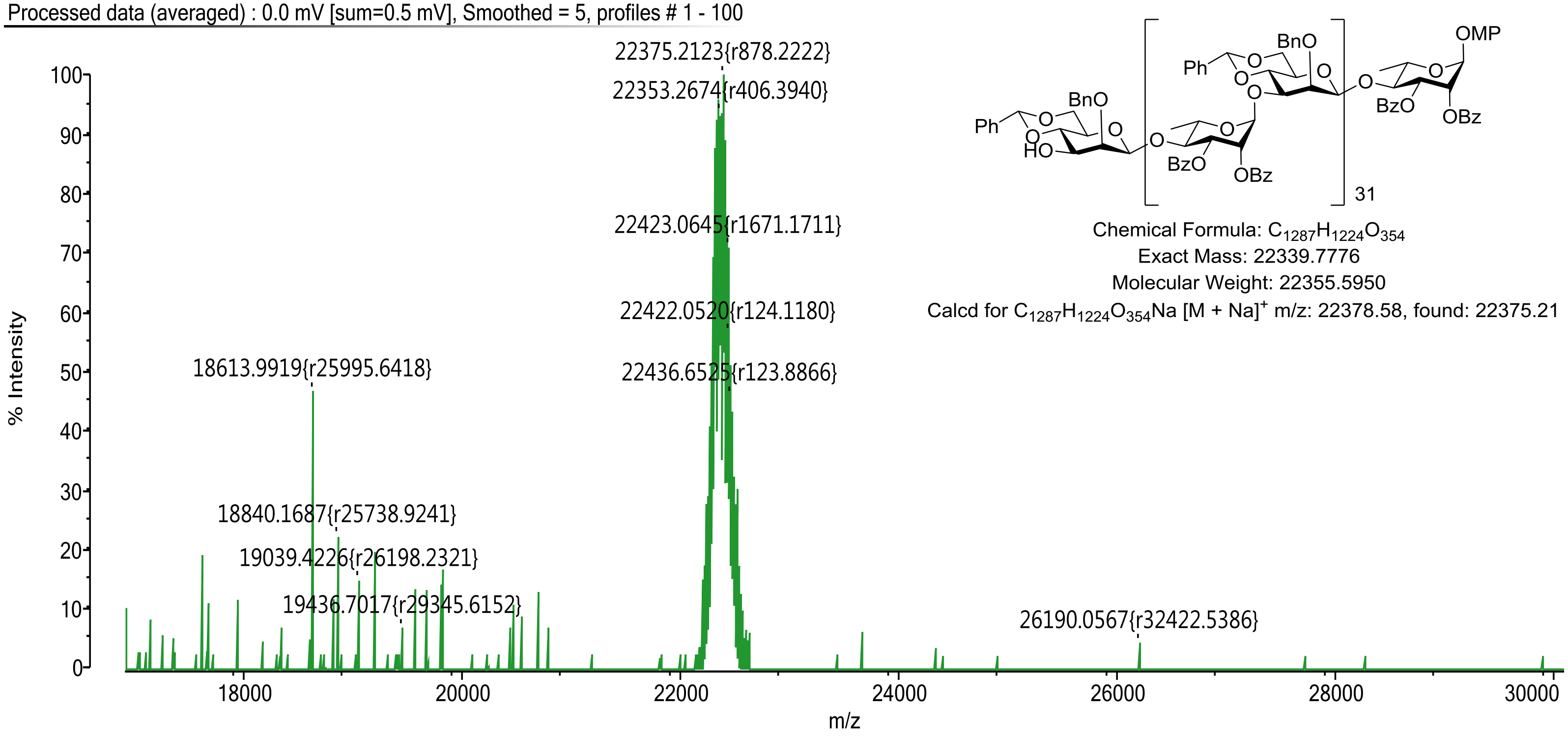

Supplementary Figure 83. MALDI-TOF MS spectrum of compound 13[A]

Data: YB20190306SAMPLE2-82_0002:P16 (Manual) Wednesday, March 06, 2019 2:30:56 PM Cal:Custom Calibration by Engineer on Wednesday, March 06, 2019 2:25:22 PM (Original)
Shimadzu MALDI-7090: Tuning Linear, Power 168, P.Ext at 20000.00 (bin 489), Ion Gate Blanking: 1000.00, Laser Diameter: 300

Processed data (averaged) : 1.0 mV [sum=101.7 mV], Unsmoothed, profiles # 1 - 100

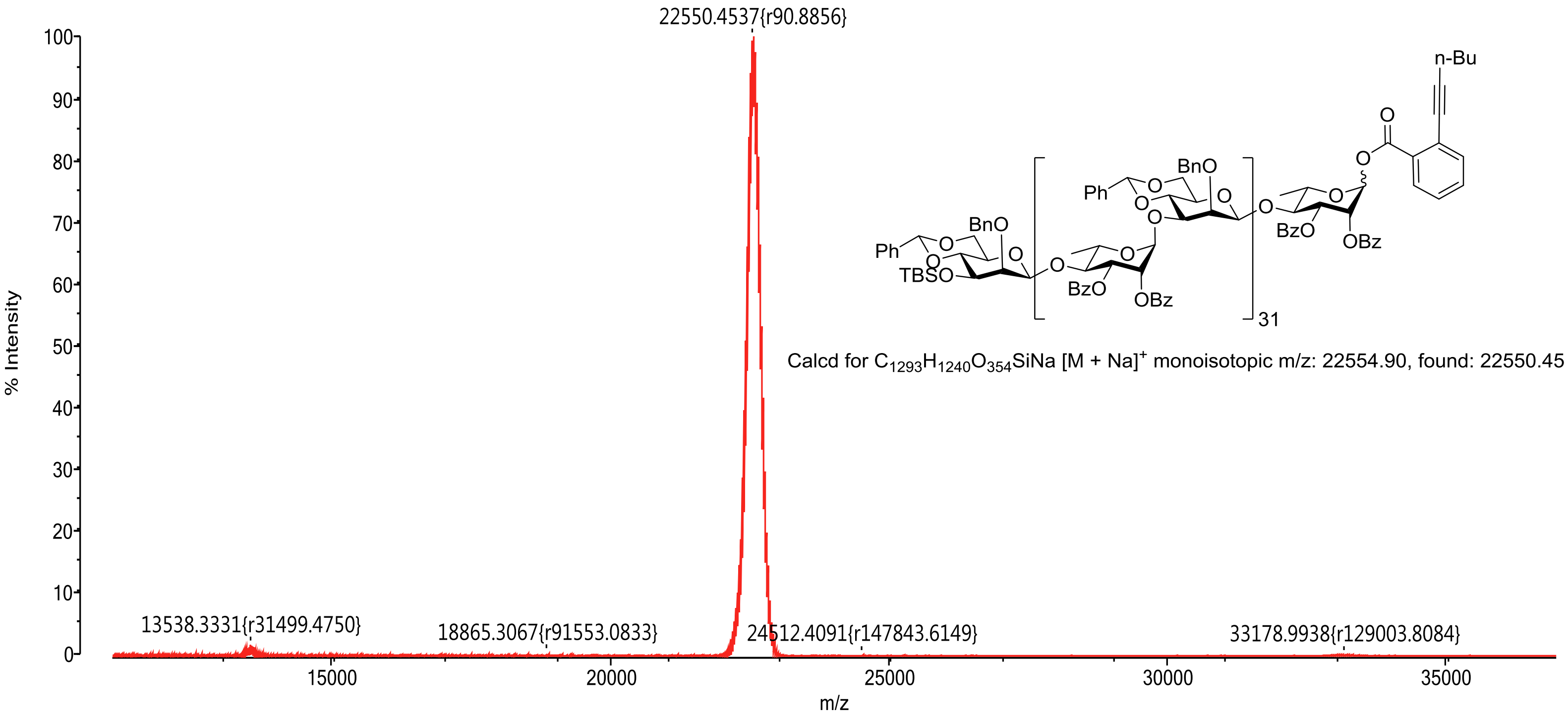

Supplementary Figure 84. MALDI-TOF MS spectrum of compound 13^D^

Data: sioc2019042920f_0004:P3 (Manual) Monday, April 29, 2019 9:33:09 AM Cal:Custom Calibration by Engineer on Monday, April 29, 2019 9:41:13 AM
Shimadzu MALDI-7090: Tuning Linear, Power 150, P.Ext at 40000.00 (bin 691), Ion Gate Blanking: 1000.00, Laser Diameter: 300
Processed data (averaged) : 0.0 mV [sum=0.1 mV], Smoothed = 100, profiles # 1 - 282

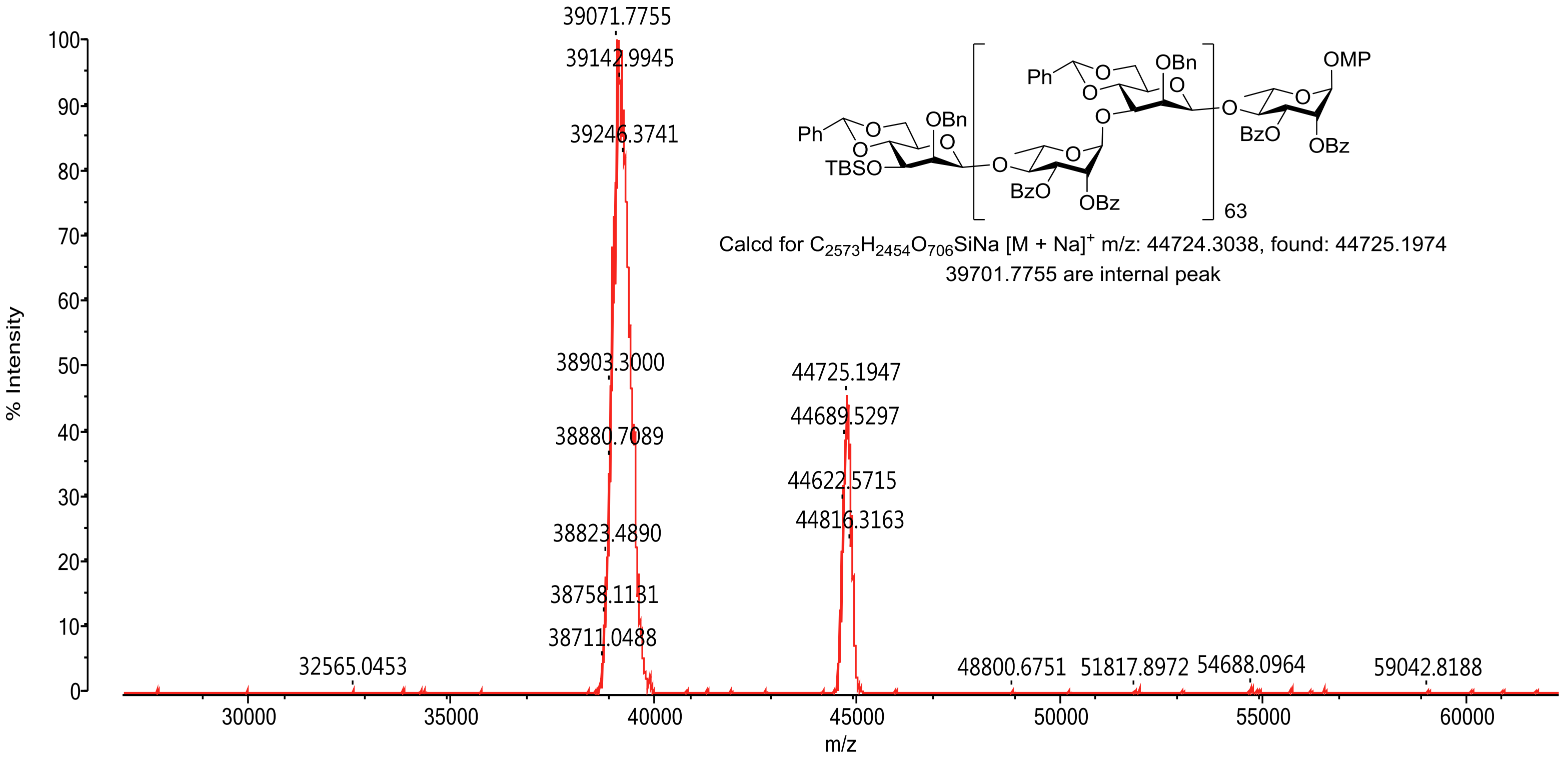

Supplementary Figure 85. MALDI-TOF MS spectrum of 128-mer 14

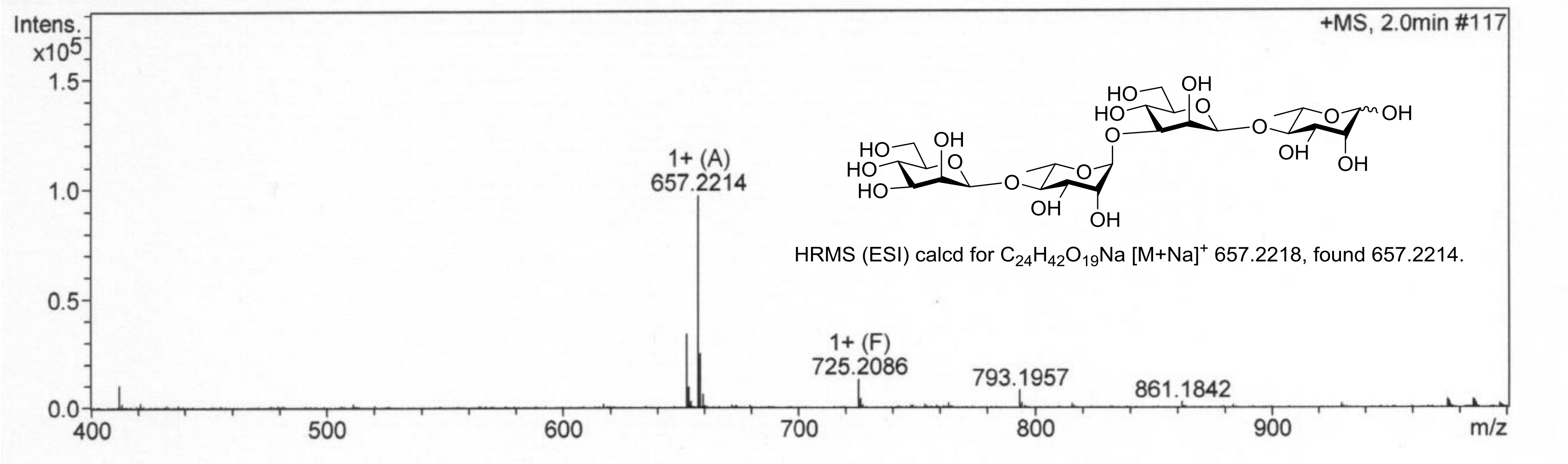

Supplementary Figure 86. HRMS (ESI) spectrum of 4-mer 2

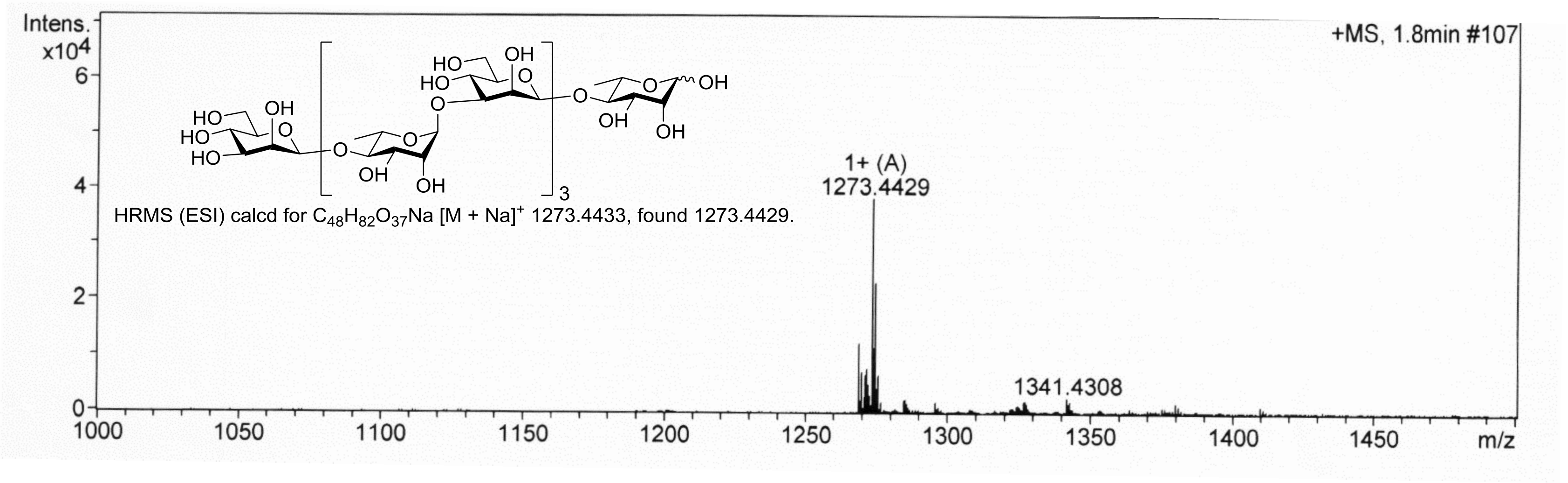

Supplementary Figure 87. HRMS (ESI) spectrum of 8-mer 3

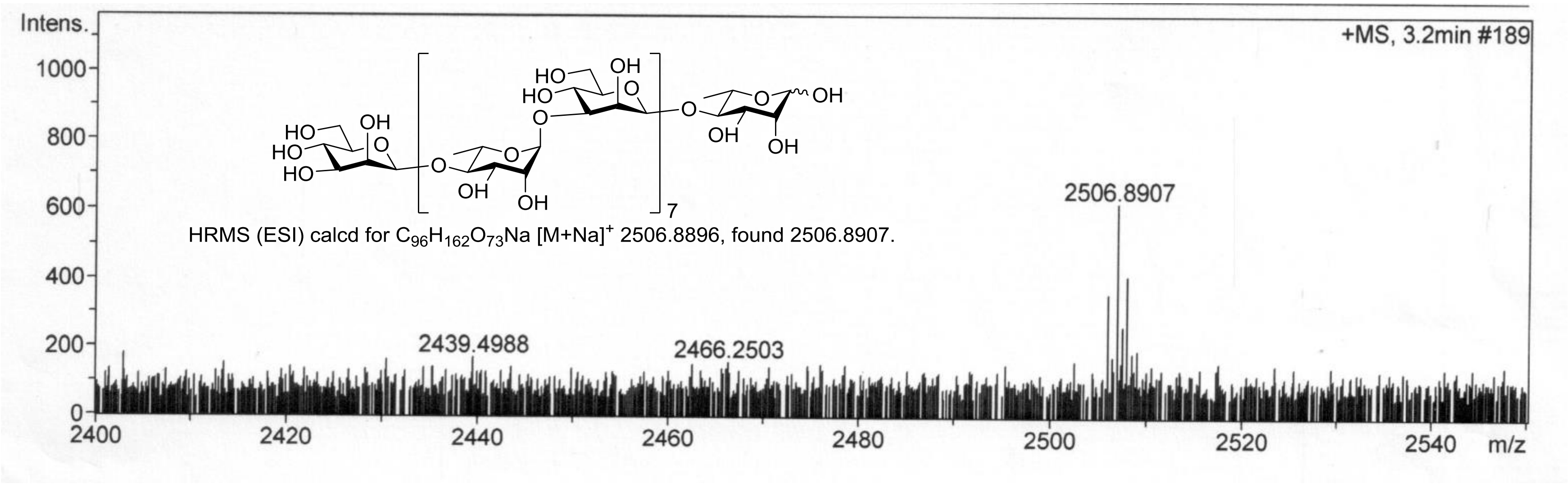

Supplementary Figure 88. HRMS (ESI) spectrum of 16-mer 4

Data: sioc20190429sample18N_0002:P7 (Manual) Monday, April 29, 2019 9:07:48 AM Cal:Custom Calibration by Engineer on Monday, April 29, 2019 9:09:35 AM
Shimadzu MALDI-7090: Tuning Linear, Power 120, P.Ext at 5000.00 (bin 244), Ion Gate Blanking: 500.00, Laser Diameter: 200
Processed data (averaged) : 1.0 mV [sum=20.7 mV], Smoothed = 10, profiles # 1 - 21

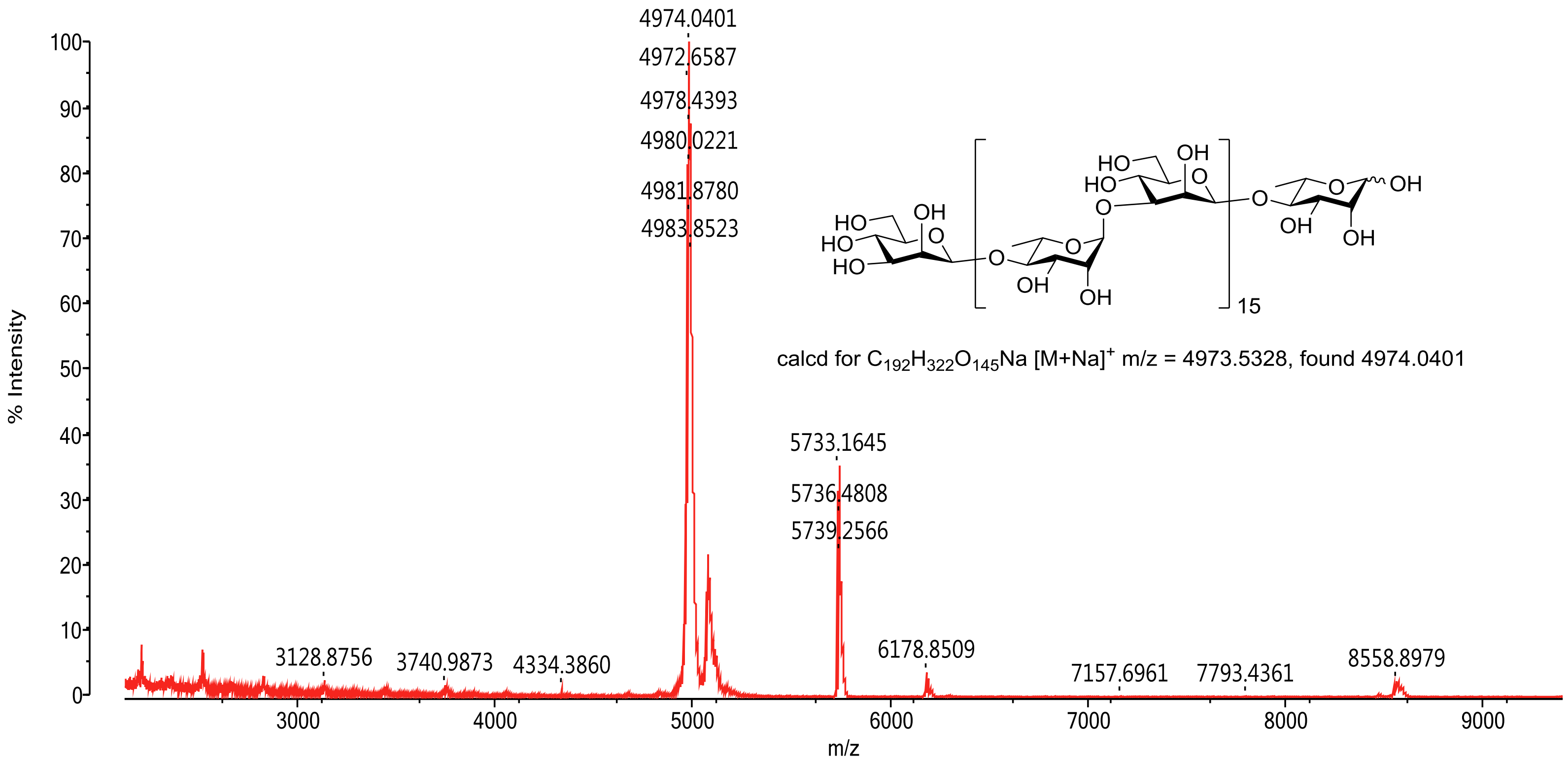

Supplementary Figure 89. MALDI-TOF MS spectrum of 32-mer 5

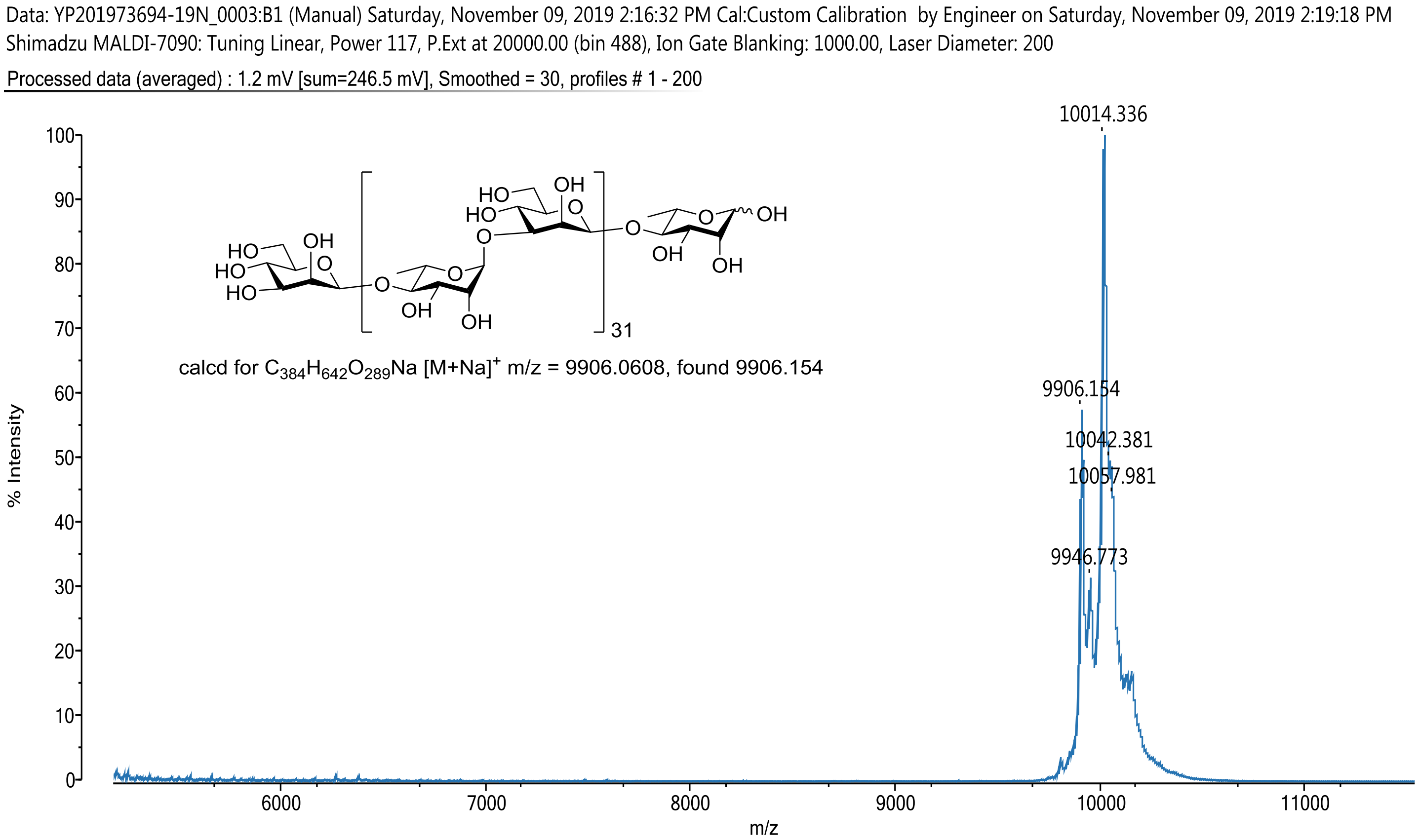

Supplementary Figure 90. MALDI-TOF MS spectrum of 64-mer 6. The peak shape in low-resolution MALDI-TOF MS data is often distorted due to several reasons including the poor homogeneity of the MALDI spots, and the low sensitivity. This led to the detection of some artificial peaks that are not real.

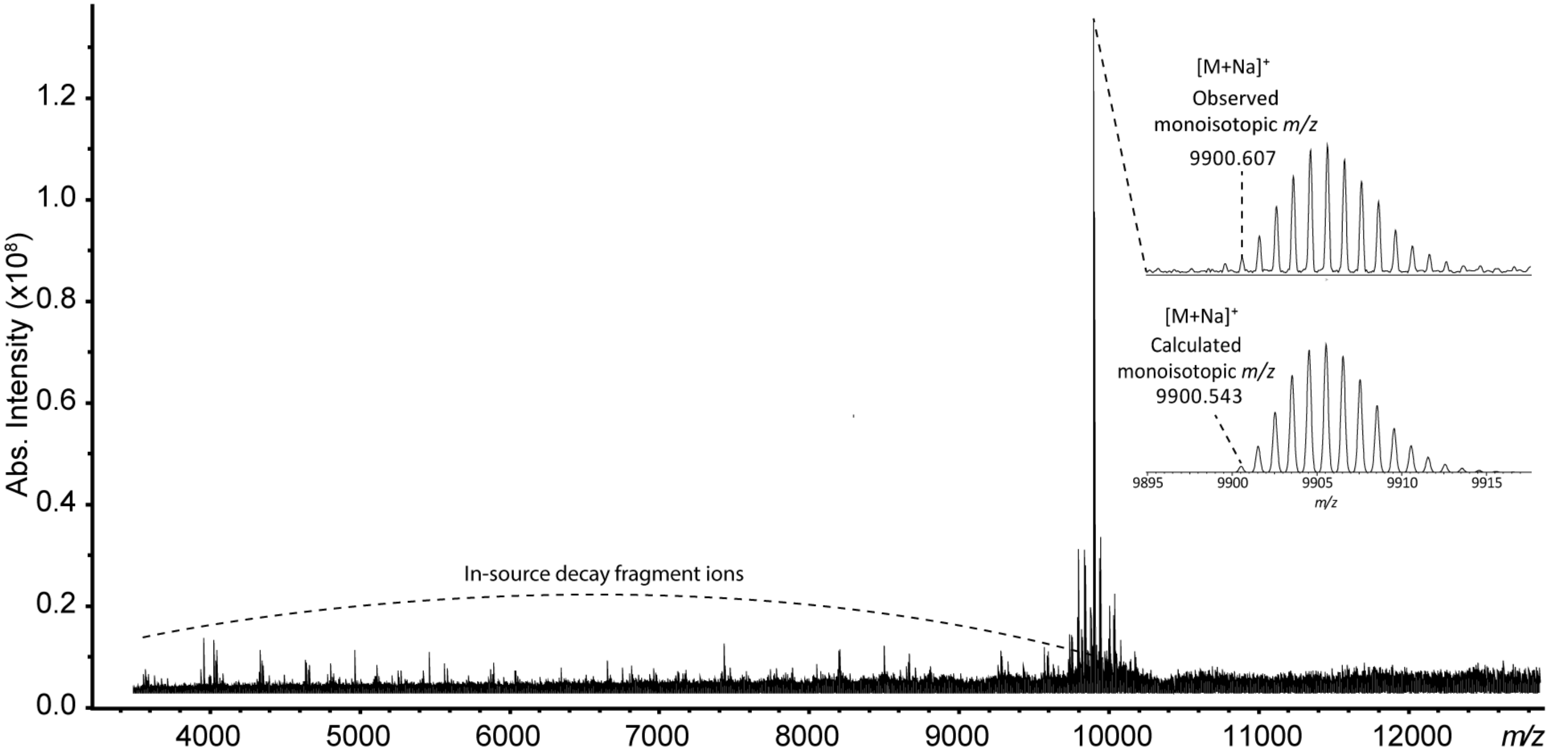

Supplementary Figure 91. MALDI-FT-ICR MS spectrum of 64-mer 6. The MALDI FT-ICR MS spectrum of 64-mer **6** is much cleaner than the previous MALDI-TOF spectrum.

Supplementary Tables

Supplementary Table 1. Studies on β-selective mannopyranosylation, Glycosylation of acceptor S6 with donors S2-S5.

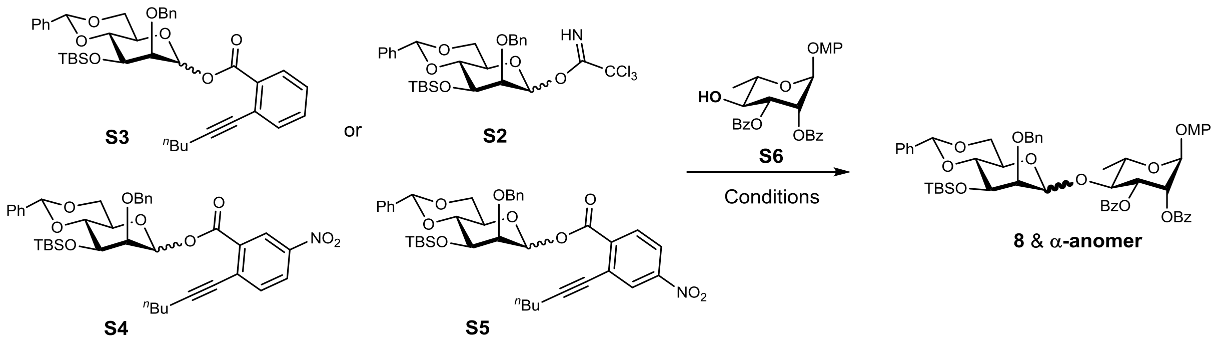

Entry	Donor (equiv.)	Catalyst (eq.) and conditions	Yield (α/β)
1	**S3** (1.2)	PPh_3AuOTf (0.1), CH_2Cl_2, 5Å MS, -20 °C, 4 h	82% (1:1.0)
2	**S3** (1.2)	$PPh_3AuBAr^F{}_4$ (0.1), CH_2Cl_2, 5Å MS, -20 °C, 5 h	32% (1:1.6)
3	**S3** (1.2)	$PPh_3AuBAr^F{}_4$ (0.1), PhCl, 5Å MS, -20 °C, 8 h	55% (1:1.7)
4	**S3** (1.2)	$PPh_3AuBAr^F{}_4$ (0.1), PhCl, 5Å MS, 0 °C, 2 h	77% (1.2:1)
5	**S4** (1.2)	$PPh_3AuBAr^F{}_4$ (0.1), CH_2Cl_2, 5Å MS, -20 °C, 5 h	48% (1:3.5)
6	**S4** (1.2)	$PPh_3AuBAr^F{}_4$ (0.1), PhCl, 5Å MS, -20 °C, 8 h	98% (1:3.5)
7	**S4** (1.2)	$PPh_3AuBAr^F{}_4$ (0.1), PhCl, 5Å MS, -30 °C, 2 h	82% (1:5.7)
8	**S5** (1.2)	$PPh_3AuBAr^F{}_4$ (0.1), PhCl, 5Å MS, -42 °C, 10 h	93% (1:11.5)
9	**S5** (1.2)	$PPh_3AuBAr^F{}_4$ (0.1), CH_2Cl_2, 5Å MS, -20 °C, 5 h	64% (1:6.5)
10	**S2** (1.2)	HOTf (0.5), CH_2Cl_2, 4Å MS, -70 °C, 1.5 h	28% (1:3.4)
11	**S2** (1.2)	HOTf (0.5), CH_2Cl_2, 4Å MS, -50 °C, 1.5 h	40% (1:2.0)
12	**S2** (0.6; 0.8 g scale)	TMSOTf (0.05), CH_2Cl_2, 4Å MS, -50 °C, 1.5 h; reverse addition	90% (1:2.2)
13	**S2** (0.6; 27 g scale)	TMSOTf (0.05), CH_2Cl_2, 4Å MS, -50 °C, 1.5 h; reverse addition	92% (1:1.6)

Supplementary Table 2. Removal of the TBS group at the non-reducing end for the preparation of glycan acceptors

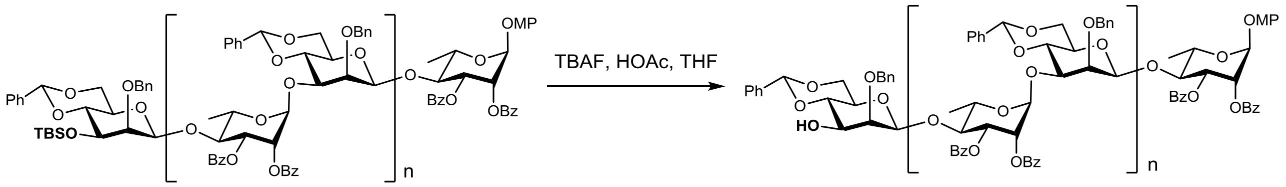

9 (n = 1), **10** (n = 3), **11** (n = 7), **12** (n = 15), **13** (n = 31) → **9^A^** (n = 1), **10^A^** (n = 3), **11^A^** (n = 7), **12^A^** (n = 15), **13^A^** (n = 31)

Substrate	Conditions	Conc.	Time	Eluent for silica gel chromatography	Product
**9** (3.53 g)	TBAF (2.0 eq.), THF (70 mL)	31 mM	12 h	EA/PE = 1:2	**9^A** (2.61 g; 80%)
**10** (1.65 g)	TBAF (4.5 eq.), HOAc (2.0 eq.), THF (11 mL)	50 mM	31 h	EA/PE/CH_2Cl_2 = 1:3:1	**10^A** (1.58 g; 99%)
**11** (1.07 g)	TBAF (5.0 eq.), HOAc (2.0 eq.), THF (6.2 mL)	30 mM	26 h	EA/PE/CH_2Cl_2 = 1:2.5:1	**11^A** (930 mg; 89%)
**12** (0.65 g)	TBAF (5.0 eq.), HOAc (2.0 eq.), THF (4 mL)	14 mM	64 h	EA/PE/CH_2Cl_2 = 1:2:1	**12^A** (570 mg; 88%)
**13** (190 mg)	TBAF (10.0 eq.), HOAc (4.0 eq.), THF (5 mL)	1.7 mM	48 h	EA/PE/CH_2Cl_2 = 1.5:4:3	**13^A** (178 mg; ~85%)[a]

[a] Because of poor solubility, the monitoring and purification of the 64-mer on silica gel turned out to be problematic, leading to **13^A** which contains small amount of impurities as indicated by NMR analysis.

Supplementary Table 3. Preparation of *o*-hexynylbenzoate donors via selective removal of the anomeric MP group and subsequent condensation with *o*-hexynylbenzoic acid

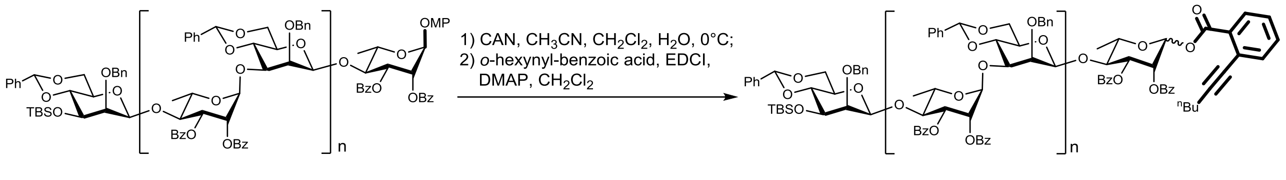

Substrate	Deprotection of MP (°C)	Condensation (rt)	Eluent for silica gel chromatography	Product
**9** (5.35 g)	CAN (4.0 eq.), $CH_3CN/CH_2Cl_2/H_2O$ (100/54/27 mL); *c* = 18 mM	hemiacetal (4.46 g), acid (1.5 eq.), EDCI (1.5 eq.), DMAP (1.0 eq.), CH_2Cl_2 (58 mL); *c* = 50 mM, 12 h	EA/PE = 1:2	$\mathbf{9^D}$ (4.10 g, 82%)
**10** (2.55 g)	CAN (4.0 eq.), $CH_3CN/CH_2Cl_2/H_2O$ (21/18/7 mL); *c* = 18 mM	hemiacetal (2.26 g), acid (1.5 eq.), EDCI (2.5 eq.), DMAP (2.5 eq.), CH_2Cl_2 (16 mL); *c* = 50 mM, 19 h	EA/PE/CH_2Cl_2 = 1:3:1	$\mathbf{10^D}$ (2.10 g, 81%)
**11** (1.78 g)	CAN (4.0 eq.), $CH_3CN/CH_2Cl_2/H_2O$ (12/6/2 mL); *c* = 16 mM	hemiacetal (1.51 g), acid (1.5 eq.), EDCI (2.5 eq.), DMAP (2.5 eq.), CH_2Cl_2 (10 mL); *c* = 27 mM, 18 h	EA/PE/CH_2Cl_2 = 1:2.5:1	$\mathbf{11^D}$ (1.18 g, 75%)
**12** (320 mg)	CAN (17 eq.), $CH_3CN/CH_2Cl_2/H_2O$ (6.5/5/1 mL); *c* = 2.2 mM	hemiacetal (237 mg), acid (1.5 eq.), EDCI (2.5 eq.), DMAP (2.5 eq.), CH_2Cl_2 (10 mL); *c* = 2.1 mM, 27 h	EA/PE/CH_2Cl_2 = 1:2:1	$\mathbf{12^D}$ (216 mg, 75%)
**13** (320 mg)	CAN (36 eq.), $CH_3CN/CH_2Cl_2/H_2O$ (4/10/1 mL); *c* = 0.95 mM	hemiacetal (301 mg), acid (7.5 eq.), EDCI (12.5 eq.), DMAP (12.5 eq.), CH_2Cl_2 (10 mL); *c* = 1.35 mM, 10 h	EA/PE/CH_2Cl_2 = 1.5:4:3	$\mathbf{13^D}$ (283 mg, 88%)[a]

[a] Because of poor solubility, the monitoring and purification of the 64-mer on silica gel turned out to be problematic, leading to $\mathbf{13^D}$ which contains a small amount of impurities as indicated by NMR analysis.

Supplementary Table 4. Preparation of glycans via the gold(I)-catalyzed [2^n+2^n] glycosylation

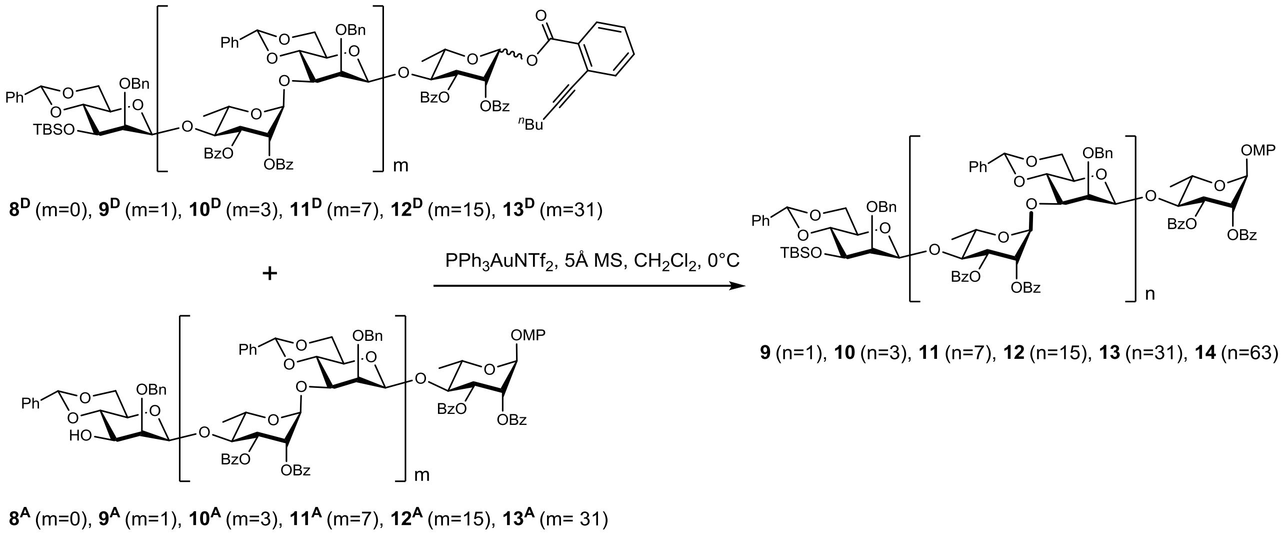

Donor<br>Acceptor	Amounts of promoter and CH_2Cl_2	Conc. based on acceptor	Reaction time	Eluent for silica gel chromatography	Product
**8^D** (6.85 g, 1.0 eq.)<br>**8^A** (5.65 g, 1.0 eq.)	$Ph_3PAuNTf_2$ (501 mg, 0.1 eq.), CH_2Cl_2 (100 mL)	67 mM	3 h	EA/PE = 1:3	**9** (9.74 g, 92%)
**9^D** (3.28 g, 1.3 eq.)<br>**9^A** (2.24 g, 1.0 eq.)	$Ph_3PAuNTf_2$ (219 mg, 0.2 eq.), CH_2Cl_2 (55 mL)	35 mM	5.5 h	EA/PE = 1:2	**10** (4.19 g, 94%)
**10^D** (2.10 g, 1.3 eq.)<br>**10^A** (1.53 g, 1.0 eq.)	$Ph_3PAuNTf_2$ (78 mg, 0.2 eq.), CH_2Cl_2 (26 mL)	20 mM	2 h	EA/PE/CH_2Cl_2 = 1:3:1	**11** (2.85 g, 93%)
**11^D** (1.08 g, 1.2 eq.)<br>**11^A** (0.85 g, 1.0 eq.)	$Ph_3PAuNTf_2$ (22 mg, 0.2 eq.), CH_2Cl_2 (8 mL)	19 mM	10 h	EA/PE/CH_2Cl_2 = 1:2.5:1	**12** (1.72 g, 92%)
**12^D** (343 mg, 1.2 eq.)<br>**12^A** (293 g, 1.0 eq.)	$Ph_3PAuNTf_2$ (3.9 mg, 0.2 eq.), CH_2Cl_2 (3.5 mL)	7.4 mM	12 h	EA/PE/CH_2Cl_2 = 1:2:1[a]	**13** (556 mg, 89%)
**13^D** (113 mg, 1.4 eq.)<br>**13^A** (80 mg, 1.0 eq.)	$Ph_3PAuNTf_2$ (0.53 mg, 0.2 eq.), CH_2Cl_2 (1 mL)	3.6 mM	19 h	EA/PE/CH_2Cl_2 = 1.5:4:3[a]	**14** (114 mg, 74%)

[a] The purification was performed on gel permeation chromatography (GPC), given the low solubility of the products in any solvent except CH_2Cl_2 and $CHCl_3$.

Supplementary Table 5. Preparation of glycans via global deprotection

**Sub.**	**Removal of MP** (°C)	**Benzoylation** (rt)	**Debenzylation** (1 atm H_2, rt)	**Zemplén de-benzoylation** (CH_3ONa, CH_3OH, rt)	**Repetitive debenzylation** (1 atm H_2, rt)	**Repetitive debenzylation** (1 atm H_2, rt)	**Prod.**
**8**[A] 181 mg	CAN (5 eq.), $MeCN/H_2O$ (6/1.5 mL); *c* = 26 mM, 2 min	BzCl (2.2 eq.), TEA (8.8 eq.), DMAP (0.1 eq.), DCM (1 mL); *c* = 194 mM, 3.5 h	10% Pd/C (100 mg), CH_3OH/HOAc (7/0.05 mL); 24 h	pH = 11, 24 h; Sephadex LH-20 ($MeOH/H_2O$ = 1:1)	-	-	**1** 30 mg 47%
**9**[A] 170 mg	CAN (10 eq.), $MeCN/H_2O$ (10/1.2 mL); *c* = 10 mM, 4 min	BzCl (5 eq.), TEA (10 eq.), DMAP (0.1 eq.), DCM (1.5 mL); *c* = 75 mM, 3 h	10% Pd/C (143 mg), CH_3OH/HOAc/ EA (2/0.2/2 mL); 24 h	pH = 11, 24 h; Sephadex LH-20 ($MeOH/H_2O$ = 1:1)	-	-	**2** 46 mg 64%
**10**[A] 46 mg	CAN (5 eq.), $MeCN/H_2O$ (8/1 mL); *c* = 2 mM, 6 h	BzCl (5 eq.), TEA (10 eq.), DMAP (10 eq.), DCM (1.5 mL); *c* = 11 mM, 11 h	10% Pd/C (206 mg), CH_3OH/HOAc/ EA (2/0.2/2 mL); 24 h	pH = 11, 18 h; Sephadex LH-20 ($MeOH/H_2O$ = 1:1)	-	-	**3** 16 mg 54%
**11**[A] 43 mg	CAN (24 eq.), MeCN/DCM /H_2O (2/1/0.5 mL); *c* = 2.5 mM, 2 mins	BzCl (7 eq.), TEA (14 eq.), DMAP (21 eq.), DCM (2 mL); *c* = 3.8 mM, 1.5 h	10% Pd/C (163 mg), CH_3OH/HOAc/ EA (2/0.3/4 mL); 24 h	pH = 11, 18 h; Sephadex G-25 (H_2O)	10% Pd/C (4.8 mg), CH_3OH/H_2O /HOAc (1/1/0.2 mL); 11 h	-	**4** 4 mg 18%
**12**[A] 58 mg	CAN (38 eq.), MeCN/DCM/ H_2O (3/3.5/0.5 mL); *c* = 0.74 mM, 30 mins	BzCl (10 eq.), TEA (20 eq.), DMAP (30 eq.), DCM (2 mL); *c* = 2.6 mM, 12 h	10% Pd/C (30 mg), 20% $Pd(OH)_2/C$ (30 mg), $CH_3OH/CHCl_3$ /H_2O/HOAc (1/2/0.1/0.1 mL); 24 h	pH = 11, 24 h; Sephadex G-25 (H_2O)	10% Pd/C (36 mg), 20% $Pd(OH)_2/C$ (40 mg), CH_3OH/H_2O /HOAc (3.5/0.2/0.2 mL); 19 h	10% Pd/C (4 mg), 20% $Pd(OH)_2/C$ (4 mg), CH_3OH/H_2O (1/1 mL), 24 h	**5** 6 mg 23%
**13**[A] 27 mg	CAN (216 eq.), MeCN/DCM/ H_2O	BzCl (43 eq.), TEA (87 eq.), DMAP (132 eq.), DCM (2 mL);	10% Pd/C (60 mg), 20% $Pd(OH)_2/C$ (60 mg), $CH_3OH/CHCl_3$	pH = 11, 17 h; Sephadex G-25 (H_2O)	10% Pd/C (4.0 mg), 20% $Pd(OH)_2/C$ (4.0 mg), CH_3OH/H_2O (1.5/1.5);	10% Pd/C (4 mg), 20% $Pd(OH)_2/C$ (4 mg), CH_3OH/H_2O /HOAc	**6** 3 mg 28%

	(4.5/4/0.5 mL); c = 0.13 mM, 2 h	c = 0.6 mM, 12 h	/H_2O/HOAc (1.5/3/0.1/0.1 mL); 50 h		15 h	(2/2/0.1 mL); 21 h	
**14**[a] 45 mg	CAN (440 eq.), MeCN/DCM/H_2O (8/5/0.5 mL); c = 0.07 mM, 1.5 h	BzCl (160 eq.), TEA (210 eq.), DMAP (160 eq.), DCM (2 mL); c = 0.5 mM, 12 h	10% Pd/C (80 mg), 20% $Pd(OH)_2$/C (80 mg), $H_3OH/CHCl_3$/H_2O/HOAc (1.5/3/0.1/0.15 mL); 48 h	pH = 11, 17 h; Sephadex LH-60 (H_2O)	10% Pd/C (12 mg), 20% $Pd(OH)_2$/C (6.0 mg), CH_3OH/H_2O/HOAc (1.5/1.5/0.1 mL); 24 h	-	**7** 3 mg 15%

[a] The TBS ether on **14** was firstly removed with TBAF using a similar procedure as that used for the preparation of the acceptors.

Supplementary Table 6. NMR assignments of 32-, 16-, 8-, 4-, 2-mer.

(^{1}H and ^{13}C chemical shifts (ppm) in D_2O, 308 K; 64-mer and 128-mer share the same values as 32-mer)

32-mer	R ^{1}H and ^{13}C		M ^{1}H and ^{13}C	
1	4.97(*s*)	96.2	4.88(*s*)	100.59
	$^1J_{H1.C1}$= 170.0 Hz		$^1J_{H1.C1}$= 162.4 Hz	
2	4.01 (*s*)	70.59	4.28	66.7
3	3.986 (*dd*)	70.31	3.69	76.98
4	3.70	79.74	3.66 (*t*)	65.07
5	4.01	67.5	3.405 (*m*)	76.19
6	1.33	17.07	3.97/3.77 *(dd)*	61.08

16-mer	R ^{1}H and ^{13}C		M ^{1}H and ^{13}C	
1	4.96(*s*)	96.2	4.88(*s*)	100.54
	$^1J_{H1.C1}$= 170.3 Hz		$^1J_{H1.C1}$= 162.5 Hz	
2	4.00 (*s*)	70.57	4.27	66.7
3	3.98 (*dd*)	70.32	3.68	76.97
4	3.69	79.73	3.65 (*t*)	65.07
5	4.00	67.5	3.39 (*m*)	76.22
6	1.32	17.12	3.93/3.77 *(dd)*	61.03

8-mer	R ^{1}H and ^{13}C		M ^{1}H and ^{13}C		R$_{red}$ ^{1}H and ^{13}C		*t*-M ^{1}H and ^{13}C	
1	4.96(*s*)	96.1	4.87(*s*)	100.54	α 5.11/ β 4.84	α 93.9/ β 93.4	4.88	100.6
	$^1J_{H1.C1}$= 170.4 Hz		$^1J_{H1.C1}$= 163.5 Hz					
2	4.01 (*s*)	70.58	4.27	66.7	3.90	71.29	4.06 (s)	66.73
3	3.98 (*dd*)	70.25	3.69	76.99	β 3.73/ α 3.92	β 72.9/ α 70.0	3.63	73.09
4	3.69	79.67	3.64 (*t*)	65.03	α 3.66/ β 3.59	α79.8/ β 79.44	3.56 (t)	66.83
5	4.00	67.4	3.39 (*m*)	76.15	α 3.93/ β 3.48	α 67.01/ 70.86	3.35 (m)	76.26
6	1.33	17.04	3.93/ 3.76 (*t / dd*)	61.02	1.34	17.04	3.75/ 3.92	61.02

4-mer	R ^{1}H and ^{13}C		M ^{1}H and ^{13}C		R$_{red}$ ^{1}H and ^{13}C		*t*-M ^{1}H and ^{13}C	
1	4.96(*s*)	96.2	4.88(*s*)	100.55	α 5.09/ β 4.84	α 93.8/ β 93.52	4.88	100.65
	$^1J_{H1.C1}$= 170.4 Hz		$^1J_{H1.C1}$= 163.5 Hz					
2	4.00 (*s*)	70.7	4.27	66.96	3.91	71.47	4.06 (s)	70.86
3	3.98 (*dd*)	70.35	3.69	77.05	α 3.92/β 3.73	α 70.0/β 72.9	3.63	73.12
4	3.69	79.59	3.65 (*t*)	65.27	α 3.66/ β 3.60	α79.89/ β 79.4	3.57 (t)	66.88
5	4.00	67.48	3.38 (*m*)	76.4	α 3.93/ β 3.48	α 67.20/ β 70.83	3.35 (m)	76.27
6	1.32	17.07	3.92/ 3.75 (*t / dd*)	61.1	α 1.33/ β 1.34	17.07	3.75/ 3.92	61.02

2-mer	M ^{1}H and ^{13}C		R$_{red}$ ^{1}H and ^{13}C	
1	4.89(*s*)	100.63	α 5.00/ β 4.85	α 93.8/ β 93.4
	$^1J_{H1.C1}$= 163.5 Hz			
2	4.07	70.66	3.91	71.37
3	3.74	73.09	α 3.92/β 3.74	α 70.1/β 72.8
4	3.57 (*t*)	66.75	α 3.60/ β 3.67	α79.2/ β 79.6
5	3.35 (*m*)	76.2	α 3.92/ β 3.48	α 66.98/ β 70.6
6	3.92/ 3.76 (*t / dd*)	61.0	1.33	17.1

Supplementary Table 7. a Calculated distances (from MM calculations) for the **M-4R** and **R-3Mb** disaccharides composing the repeating units; **b** Experimental and calculated *inter*-proton distances of 2-, 4-, 8-, 16-, 32-mer.

a

M- 4R	exo-Φ/syn-Ψ (Φ/Ψ ca. 56/36)	exo-Φ/anti-Ψ (Φ/Ψ ca. 50/-177)	R-3M	
**M1-R4**	2.77	3.70	**R1-M2**	2.31
**M1-R3**	3.43	3.38	**R1-M3**	2.870
**M1-R5**	4.51	2.35		

b

	32 Mer		16 Mer		8 Mer		4 Mer		2 Mer	
	Exp. (ROESY)	Calculated	Exp. (HS-RO)	Calculated	Exp. (HS-RO)	Calculated	Exp. (ROESY)	Calculated	Exp. (ROESY)	Calculated
**M1-R4**	2.48	2.680 ± 0.34	2.52	2.78 ± 0.34	2.58	2.70 ± 0.285	2.72	2.68 ± 0.27	2.76	2.77 ± 0.22
**M1-R3**	3.46	3.44± 0.41	3.27	3.46± 0.41	3.42	3.52 ± 0.33	3.40	3.55±0.31	3.31	3.42± 0.41
**M1-R5**	----	4.48 ± 0.15	-----	4.47 ± 0.147	------	4.50 ± 0.14	------	4.51±0.13	------	4.50±0.12
**R1-M3**	2.68	2.82 ± 0.32	2.87	2.83 ± 0.32	2.83	2.90 ± 0.29	2.73	2.90±0.26		
**R1-M2**	2.20	2.38 ± 0.48	2.38	2.39 ± 0.49	2.31	2.28 ± 0.28	2.38	2.23±0.18		

Supplementary Methods

General methods for synthesis and characterization

Reactions were carried out in glassware, unless otherwise noted. Crushed 4Å or 5Å molecular sieves were activated through flame-drying under high vacuum immediately prior to use. All chemicals were purchased as reagent grade and used without further purification, unless otherwise noted. Analytical thin-layer chromatography was performed using Merck pre-coated silica gel 60 F-254 plates. Compound spots were visualized by UV light (254 nm) and immersion into a solution of 5% H_2SO_4 in ethanol, followed by hot air gun heating. Column chromatography was performed on silica gel (200-300 mesh). Gel filtration was performed on Sephadex LH-60, LH-20, or G25 (J & K). Optical rotations were obtained on Anton Paar MCP 5500 polarimeter at 589 nm (Na). NMR spectra were recorded on Bruker AM 400, Varian 500, or Agilent 600 MHz NMR spectrometers at 25 ℃. Chemical shifts (δ) are reported in ppm and coupling constants in Hz. Solvents were $CDCl_3$, CD_3OD, or D_2O and their residual solvent peaks as internal standards. Assignments of resonances in ^{1}H and ^{13}C NMR spectra were done using ^{1}H-^{1}H COSY, HSQC, and HMBC experiments. Splitting patterns are indicated as s (singlet), d (doublet), t (triplet), q (quartet), and br s (broad singlet) for ^{1}H NMR data. HRMS experiments were performed on a Bruker maXis 4G spectrometer. MALDI-TOF mass spectra were performed on Shimadzu MALDI-7090 with dithranol as the matrix and Cytochrome C (Equine) or Aldolase (Rabbit muscle) as internals. Recycle preparative gel permeation chromatography (GPC) were performed on a JAI-9201 series HPLC (columns: 1H and 3H, UV detector: JACSO MD2010, eluting solvent: $CHCl_3$, flow rate: 3.5 mL/min, pressure: 21 MPa).

Abbreviations list

Ag_2O	Silver oxide
Bn	Benzyl
Bz	Benzoyl
CAN	Cerium (IV) diammonium nitrate
DMAP	4,4-Dimethylaminopyridine
EtOAc or EA	Ethyl acetate

EDCI	1-(3-Dimethylaminopropyl)-3-ethylcarbodiimide hydrochloride
equiv. or eq.	Equivalent
Et	Ethyl
ESI	Electron spray ionization
GPC	Gel permeation chromatography
HOAc	Acetic acid
m/z	Ratio of mass to charge
MALDI	Matrix-assisted laser desorption-ionization
Me	Methyl
MeCN	Acetonitrile
MP	4-Methoxyphenyl
MS	Molecular sieves
Mw	Molecular weight
NMR	Nuclear magnetic resonance
NTf_2	Bis[(trifluoromethyl)sulfonyl]imide
Ph	Phenyl
$BAr^F{}_4$<br>rt	Tetrakis[3,5-bis(trifluoromethyl)phenyl]borate<br>Room temperature
TBAF	Tetrabutylammonium fluoride
TBS	*Tert*-Butyldimethylsilyl
TBSOTf	*Tert*-Butyldimethylsilyltrifluoromethane sulfonate
Tf	(Trifluoromethyl)sulfonyl
TfOH	Trifluoromethanesulfonic acid
THF	Tetrahydrofuran
TLC	Thin layer chromatography
TMSOTf	Trimethylsilyl trifluoromethane sulfonate

2-*O*-Benzyl-4,6-*O*-benzylidene-3-*O*-*tert*-butyldimethylsilyl-D-mannopyranosyl donors S2-S5

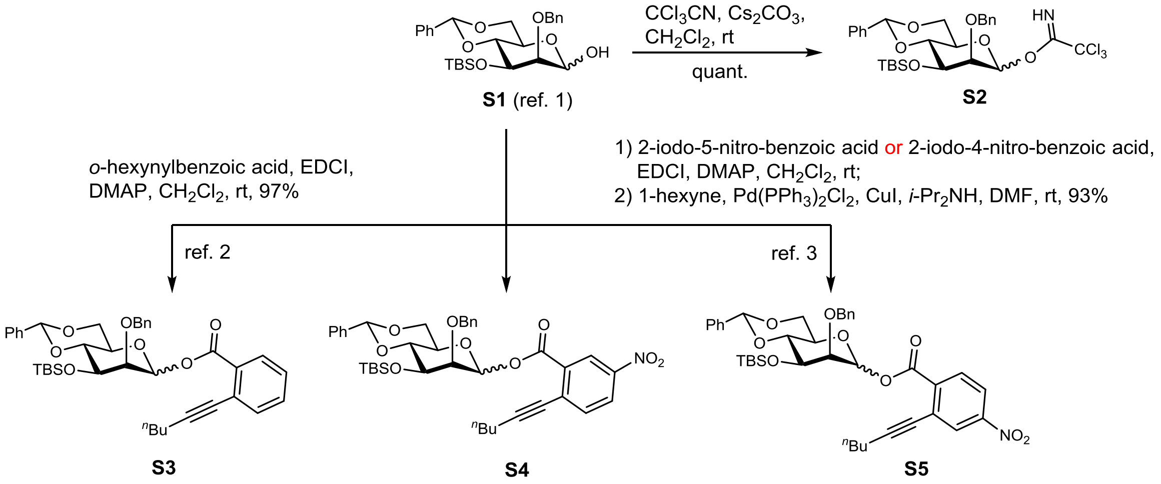

To a mixture of hemiacetal **S1**[1] (720 mg, 1.52 mol) and Cs_2CO_3 (49 mg, 0.15 mmol) in CH_2Cl_2 (20 mL), was added CCl_3CN (611 µL, 6.09 mmol) at rt. The mixture was stirred for 6 h and filtered through Celite. The solution was concentrated *in vacuo* to give imidate **S2** as a yellowish syrup, which was used for glycosylation without further purification.

To a solution of hemiacetal **S1** (236 mg, 0.05 mmol), EDCI (155 mg, 0.60 mmol), and DMAP (6.0 mg, 0.05 mmol) in anhydrous CH_2Cl_2 (1.5 mL), was added *ortho*-hexynylbenzoic acid (121 mg, 0.60 mmol) at rt. The mixture was stirred at rt overnight, and was then diluted with EtOAc and washed with sat. aq. $NaHCO_3$. The organic layer was dried over anhydrous Na_2SO_4, filtered, and concentrated. The residue was purified by silica gel column chromatography (petroleum ether/EtOAc, 8:1) to give compound **S3**[2] (319 mg, 97%, α/β = 1:1.5) as a white foam.

To a mixture of hemiacetal **S1** (320 mg, 0.68 mmol), 2-iodo-5-nitro-benzoic acid (238 mg, 0.81 mmol), EDCI (156 mg, 0.81 mmol), and DMAP (9 mg, 0.07 mmol), was added anhydrous CH_2Cl_2 (6 mL)[3]. The mixture was stirred at rt overnight, and was then diluted with EtOAc and washed with water, sat. aq. $NaHCO_3$, and brine, respectively. The organic layer was dried over anhydrous Na_2SO_4 and concentrated *in vacuo*. The residue was purified by silica gel column chromatography (petroleum ether/EtOAc, 8:1) to give a yellowish foam. A mixture of the foam above (250 mg, 0.33 mmol), $Pd(PPh_3)_2Cl_2$ (21 mg,

0.03 mmol), copper iodide (12 mg, 0.06 mmol), and *i*-Pr_2NH (141 μL, 1.0 mmol) in degassed DMF (10 mL) was degassed for 10 min, to which 1-hexyne (69 μL, 0.60 mmol) was added. The mixture was stirred at rt overnight, and was then quenched with sat. aq. NH_4Cl. The mixture was diluted with EtOAc, and was then washed with sat. aq. $NaHCO_3$ and brine, respectively. The organic layer was dried over anhydrous Na_2SO_4 and concentrated *in vacuo*. The residue was purified by silica gel column chromatography (petroleum ether/EtOAc, 8:1) to afford **S4** (221 mg, 93% for two steps) as a yellowish syrup. R_f = 0.50; ^{1}H NMR (500 MHz, $CDCl_3$) δ 8.78 (d, *J* = 2.4 Hz, 1H), 8.30 (dd, *J* = 8.6, 2.5 Hz, 1H), 7.71 (d, *J* = 8.6 Hz, 1H), 7.51 (dd, *J* = 7.4, 2.4 Hz, 2H), 7.49–7.44 (m, 2H), 7.42–7.32 (m, 6H), 6.45 (d, *J* = 1.8 Hz, 1H), 5.65 (s, 1H), 4.98 (d, *J* = 11.7 Hz, 1H), 4.83 (d, *J* = 11.8 Hz, 1H), 4.40 (dd, *J* = 9.8, 3.3 Hz, 1H), 4.31 (dd, *J* = 10.3, 4.8 Hz, 1H), 4.23 (t, *J* = 9.7 Hz, 1H), 4.09 (tt, *J* = 9.6, 4.7 Hz, 1H), 3.91–3.86 (m, 2H), 2.68–2.50 (m, 2H), 1.70–1.57 (m, *J* = 7.3 Hz, 2H), 1.48–1.36 (m, 2H), 0.96–0.89 (m, 12H), 0.14 (s, 3H), 0.12 (s, 3H); ^{13}C NMR (126 MHz, $CDCl_3$) δ 162.71, 146.13, 137.89, 137.42, 136.17, 131.73, 131.33, 128.92, 128.56, 128.10, 128.01, 127.97, 126.38, 126.20, 126.05, 104.04, 101.97, 94.71, 79.07, 78.61, 78.28, 74.49, 70.36, 68.52, 67.26, 30.52, 25.95, 25.93, 22.10, 20.09, 18.44, 13.69, -4.34, -4.69; HRMS (ESI) calcd for $C_{39}H_{48}O_9NSi$ $[M + NH_4]^+$ 702.3092, found 702.3091.

To a mixture of hemiacetal **S1** (1.00 g, 2.10 mmol), 2-iodo-4-nitro-benzoic acid (744 mg, 2.50 mmol), EDCI (480 mg, 2.50 mmol), and DMAP (26 mg, 0.21 mmol), was added anhydrous CH_2Cl_2 (20 mL)[3]. After being stirred at rt overnight, the mixture was diluted with EtOAc and washed with water, sat. aq. $NaHCO_3$, and brine, respectively. The organic layer was dried over anhydrous Na_2SO_4 and concentrated *in vacuo*. The residue was purified by silica gel column chromatography (petroleum ether/EtOAc, 8:1) to give a white foam. A mixture of the foam above (770 mg, 1.03 mmol), $Pd(PPh_3)_2Cl_2$ (70 mg, 0.10 mmol), copper iodide (39 mg, 0.21 mmol), and *i*-Pr_2NH (478 μL, 3.40 mmol) in degassed DMF (15 mL) was degassed for 10 min, to which 1-hexyne (212 μL, 1.85 mmol) was added. The mixture was stirred at rt overnight, and was then quenched with sat. aq. NH_4Cl. The mixture was diluted with EtOAc, and was then washed by sat. aq. $NaHCO_3$ and brine, respectively. The organic layer was dried over anhydrous Na_2SO_4 and concentrated *in vacuo*. The residue was purified by silica gel column chromatography (petroleum

ether/EtOAc, 8:1) to afford **S5** (701 mg, 93% for two steps) as a yellowish syrup. R_f = 0.50; ^{1}H NMR (500 MHz, $CDCl_3$) δ 8.37 (d, *J* = 2.3 Hz, 1H), 8.15 (dd, *J* = 8.7, 2.3 Hz, 1H), 8.05 (d, *J* = 8.6 Hz, 1H), 7.53–7.48 (m, 2H), 7.48–7.44 (m, 2H), 7.42–7.31 (m, 6H), 6.44 (d, *J* = 1.8 Hz, 1H), 5.65 (s, 1H), 4.98 (d, *J* = 11.7 Hz, 1H), 4.82 (d, *J* = 11.7 Hz, 1H), 4.35 (dd, *J* = 9.8, 3.3 Hz, 1H), 4.31 (dd, *J* = 10.3, 4.8 Hz, 1H), 4.21 (t, *J* = 9.6 Hz, 1H), 4.09 (td, *J* = 9.8, 4.8 Hz, 1H), 3.93–3.84 (m, 2H), 2.65–2.47 (m, 2H), 1.67–1.57 (m, 2H), 1.41 (m, 2H), 0.92 (m, 12H), 0.11 (s, 3H), 0.09 (s, 3H); ^{13}C NMR (126 MHz, $CDCl_3$) δ 163.42, 149.69, 137.93, 137.43, 135.94, 131.91, 129.65, 128.96, 128.57, 128.40, 128.13, 128.01, 127.96, 126.50, 126.20, 121.77, 101.96, 100.51, 94.85, 78.66, 78.32, 78.27, 77.41, 77.16, 76.91, 74.58, 70.32, 68.54, 67.27, 30.61, 25.96, 22.13, 19.88, 18.43, 13.72, -4.27, -4.63; HRMS (ESI) calcd for $C_{39}H_{47}NO_9SiNa$ $[M + Na]^+$ 724.2918, found 724.2898.

p-Methoxyphenyl 2,3-di-*O*-benzoyl-α-L-rhamnopyranoside (S6)

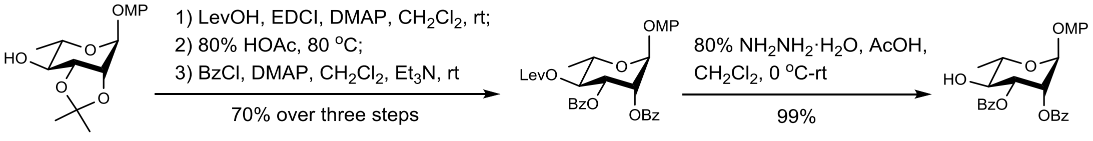

S7 (ref. 4) **S8** **S6**

To a mixture of 4-methoxyphenyl 2,3-*O*-isopropylidene-α-L-rhamnopyranoside (**S7**)[4] (23.6 g, 75.8 mmol), EDCI (17.4 g, 91.0 mmol), and DMAP (0.93 g, 7.6 mmol) in CH_2Cl_2 (120 mL), was added levulinic acid (9.32 mL, 91.0 mmol) at 0 °C. The mixture was stirred at rt overnight, and was then diluted with CH_2Cl_2, and washed with water, sat. aq. $NaHCO_3$, and brine, respectively. The organic layer was dried over anhydrous Na_2SO_4 and concentrated *in vacuo*. The resulting residue was used for the next step without further purification.

A mixture of the residue above in 80% aq. HOAc (130 mL) was heated at 80 °C. After consumption of the substrate, the solvent was removed *in vacuo*. The resulting residue was used for the next step directly.

To a mixture of the residue above and DMAP (0.58 g, 4.76 mmol) in Et_3N/CH_2Cl_2 (66 mL/60 mL), was added benzoyl chloride (13.7 mL, 119 mmol) at 0 °C. The mixture was stirred at rt overnight, and was then diluted with CH_2Cl_2, and washed with water, sat. aq. $NaHCO_3$, and brine, respectively. The organic layer was dried over anhydrous Na_2SO_4

and concentrated *in vacuo*. The residue was purified by silica gel column chromatography (petroleum ether/EtOAc, 2:1) to give compound **S8** (29.2 g, 70% over three steps) as a yellowish foam. R_f = 0.60; $[\alpha]_D^{25}$ = 25.9 (*c* 1.0, $CHCl_3$); ^1H NMR (500 MHz, $CDCl_3$) δ 8.11–8.05 (m, 2H), 7.95–7.89 (m, 2H), 7.63–7.55 (m, 1H), 7.54–7.47 (m, 1H), 7.46 (t, *J* = 7.8 Hz, 2H), 7.38–7.31 (m, 2H), 7.11–7.04 (m, 2H), 7.02–6.95 (m, 1H), 6.85 (dd, *J* = 9.2, 3.0 Hz, 3H), 5.87 (dd, *J* = 10.1, 3.5 Hz, 1H), 5.80 (dd, *J* = 3.5, 1.8 Hz, 1H), 5.58 (d, *J* = 1.9 Hz, 1H), 5.50 (t, *J* = 10.0 Hz, 1H), 4.21 (dq, *J* = 9.8, 6.2 Hz, 1H), 3.76 (d, *J* = 2.5 Hz, 3H), 2.86–2.75 (m, 1H), 2.68 (m, 1H), 2.63–2.50 (m, 2H), 2.40 (m, 1H), 2.19 (s, 1H), 2.05 (s, 3H), 1.34 (d, *J* = 6.2 Hz, 3H); ^{13}C NMR (126 MHz, $CDCl_3$) δ 206.45, 205.99, 205.97, 172.03, 171.81, 165.51, 165.48, 157.21, 155.25, 150.00, 144.16, 133.57, 133.31, 129.89, 129.79, 129.25, 128.61, 128.42, 122.27, 117.73, 114.67, 114.38, 96.50, 77.42, 77.17, 76.91, 71.36, 70.62, 69.85, 67.15, 55.61, 55.53, 37.92, 37.81, 29.86, 29.56, 28.09, 27.95, 17.54, 14.21; HRMS (ESI) calcd for $C_{32}H_{32}O_{10}Na$ $[M + Na]^+$ 599.1888, found 599.1890.

To a solution of **S8** (27.4 g, 47.6 mmol) and HOAc (8.16 mL, 142 mmol) in CH_2Cl_2 (300 mL), was added hydrazinehydrate (80% aq., 6.93 mL, 142 mmol) at 0 ^oC. The mixture was stirred at rt for 5.5 h. After being quenched with acetone (10 mL), the mixture was diluted with CH_2Cl_2, and washed with water, sat. aq. $NaHCO_3$, and brine, respectively. The organic layer was dried over anhydrous Na_2SO_4 and concentrated *in vacuo*. The residue was purified by silica gel column chromatography (petroleum ether/EtOAc, 2:1) to give compound **S6** (23.0 g, 99%) as a colorless syrup. R_f = 0.55; $[\alpha]_D^{25}$ = -23.2 (*c* 1.0, $CHCl_3$); ^1H NMR (500 MHz, $CDCl_3$) δ 8.13–8.06 (m, 2H), 7.97–7.89 (m, 2H), 7.66–7.59 (m, 1H), 7.57–7.46 (m, 3H), 7.39–7.31 (m, 2H), 7.11–7.03 (m, 2H), 6.89–6.82 (m, 2H), 5.78 (dd, *J* = 3.4, 1.8 Hz, 1H), 5.74 (dd, *J* = 9.6, 3.5 Hz, 1H), 5.56 (d, *J* = 1.8 Hz, 1H), 4.08 (dq, *J* = 9.4, 6.0 Hz, 1H), 4.01 (t, *J* = 9.5 Hz, 1H), 3.78 (s, 3H), 1.43 (d, *J* = 6.1 Hz, 3H); ^{13}C NMR (126 MHz, $CDCl_3$) δ 167.06, 165.70, 155.26, 153.60, 150.20, 149.89, 133.67, 133.53, 129.97, 129.94, 129.51, 129.37, 128.69, 128.50, 117.91, 116.11, 114.88, 114.79, 96.75, 73.06, 72.05, 70.96, 69.48, 55.76, 17.84; HRMS (ESI) calcd for $C_{27}H_{26}O_8Na$ $[M + Na]^+$ 501.1520, found 501.1522.

General procedure for the gold(I)-catalyzed glycosylation (Supplementary Table 1 entries 1-9)

A mixture of the donor (**S3-S5**), acceptor (**S6**), PPh_3AuCl, and freshly activated 5Å MS in anhydrous solvent was stirred at rt for 20 min, and was then cooled down to the reaction temperature for 10 min, to which $AgBAr^F{}_4$ (0.45 M in Et_2O) or AgOTf was added. The stirring was continued until TLC showed the donor was fully consumed. The reaction was quenched with PPh_3. The mixture was filtered and concentrated. The resulting residue was purified by silica gel column chromatography (petroleum ether/EtOAc, 4:1) to give the coupled disaccharide **8** and its α-anomer as a white foam. The anomeric ratio of the disaccharides was determined by ^{1}H NMR analysis.

General procedure for the HOTf catalyzed glycosylation (Supplementary Table 1 entries 10 and 11)

A mixture of imidate donor **S2**, acceptor **S6**, and freshly activated 4Å MS in anhydrous CH_2Cl_2 was stirred at rt for 20 min, and was then cooled down to the reaction temperature for 10 min. HOTf was added, and the stirring was continued until TLC showed that the donor was fully consumed. After being quenched with Et_3N, the reaction mixture was filtered and concentrated. The resulting residue was purified by silica gel column chromatography (petroleum ether/EtOAc, 4:1) to give the coupled disaccharide **8** and its α-anomer as a white foam. The anomeric ratio of the disaccharides was determined by ^{1}H NMR analysis.

An optimal procedure (entry 8) for the preparation of disaccharide **8** was found, with 2-hexynyl-4-nitro-benzoate **S5** as donor, $PPh_3AuBAr^F{}_4$ (0.1 equiv.) as catalyst, PhCl as solvent, in the presence of 5Å MS at -42 ^oC in 10 h, which led to the coupled disaccharides in 93% yield with an excellent β/α ratio of 11.5:1. Nevertheless, considering the cost of the procedure, an inexpensive route (entry 13) was used for large scale preparation of disaccharide **8**. Although the glycosylation of **S6** with imidate donor **S2** (0.05 equiv. TMSOTf, CH_2Cl_2, 4Å MS, -50 ^oC, 1.5 h) led to the disaccharides in a moderate β/α selectivity of 1.6:1 (92% yield), subsequent removal of the 3'-O-TBS group led to conveniently separable isomers **8^A** and **S9**. Compound **8^A**, the desired disaccharide acceptor, was easily converted back to the TBS ether **8**. This practical route was described below (Supplementary Figure 1).

A decagram-scale synthetic approach to disaccharide 8 along with acceptor 8^A (Supplementary Figure 1)

A mixture of acceptor **S6** (14.5 g, 30.2 mmol) and 4Å MS (20 g) in a round-bottom flask equipped with a Teflon-coated magnetic stir bar was dried in high vacuum at rt for 4 h, and then anhydrous CH_2Cl_2 (90 mL) was added. The mixture was stirred at rt for 15 min, and was then cooled to -50 °C, to which TMSOTf (0.33 mL, 1.5 mmol) was added, followed by addition of an anhydrous CH_2Cl_2 (133 mL) solution of **S2** (20.5 g, 33.2 mmol) by syringe pump (flow rate: 6 mL/min). The resulting mixture was stirred at -50 °C for 0.5 h. After addition of Et_3N (10 mL), the mixture was filtrated through a pad of Celite. The filtrate was concentrated *in vacuo* to give a residue, which was purified by silica gel column chromatography (petroleum ether/EtOAc, 4:1) to give the coupled disaccharide **8** and its α-anomer (27.0 g, 92%, β/α = 1.6:1 based on ^{1}H NMR analysis) as a white foam.

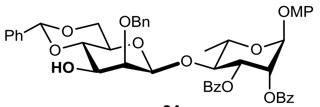

8A

To a solution of the disaccharide **8** and its α-anomer (27.0 g, β/α = 1.6:1, 28.9 mmol) in anhydrous THF (500 mL), was added TBAF (1.0 M THF, 58 mL, 58.0 mmol). The mixture was stirred at rt for 9 h, and was then diluted with EtOAc and washed with sat. aq. $NaHCO_3$ and brine, respectively. The organic layer was dried over anhydrous Na_2SO_4 and concentrated *in vacuo*. The residue was purified by silica gel column chromatography (petroleum ether/EtOAc/CH_2Cl_2, 4:1:1) to give **8^A** (12.9 g, 52%) and **S9** (8.0 g, 32%) as white foams. The anomeric configurations were determined by NMR analysis.

8^A: R_f = 0.40; $[\alpha]_D^{20}$ = -35.1 (*c* 3.0, $CHCl_3$); ^{1}H NMR (600 MHz, $CDCl_3$) δ 8.11–8.06 (m, 2H), 7.97–7.92 (m, 2H), 7.68–7.62 (m, 1H), 7.61–7.55 (m, 1H), 7.52 (t, *J* = 7.8 Hz, 2H), 7.48–7.44 (m, 2H), 7.42 (t, *J* = 7.8 Hz, 2H), 7.36–7.32 (m, 3H), 7.32–7.27 (m, 1H), 7.14–7.08 (m, 2H), 6.90–6.84 (m, 2H), 5.90 (dd, *J* = 9.5, 3.5 Hz, 1H), 5.80 (dd, *J* = 3.5, 1.9 Hz, 1H), 5.54 (d, *J* = 1.9 Hz, 1H), 5.52 (s, 1H), 4.97 (d, *J* = 11.7 Hz, 1H), 4.75 (d, *J* = 1.1 Hz, 1H), 4.57 (d, *J* = 11.7 Hz, 1H), 4.36 (dd, *J* = 10.4, 4.9 Hz, 1H), 4.16–4.07 (m, 1H), 4.05 (t, *J* = 9.5 Hz, 1H), 3.87 (t, *J* = 10.3 Hz, 1H), 3.79 (s, 3H), 3.75 (t, *J* = 9.5 Hz, 1H), 3.66 (d, *J* = 3.7 Hz, 1H), 3.43 (dd, *J* = 9.8, 3.7 Hz, 1H), 3.30 (td, *J* = 9.7, 4.9 Hz, 1H), 1.47 (d, *J* = 6.1 Hz, 3H); ^{13}C NMR (151 MHz, $CDCl_3$) δ 165.56, 165.44, 155.36, 150.34, 138.16, 137.20, 133.82, 133.74, 129.94, 129.94, 129.55, 129.53, 129.32, 129.22, 128.89, 128.74,

128.61, 128.35, 128.27, 128.08, 126.32, 117.98, 114.78, 102.90, 102.09, 96.93, 79.21, 78.64, 78.51, 77.37, 77.16, 76.95, 75.93, 72.32, 70.98, 70.61, 68.59, 68.56, 68.12, 68.03, 67.23, 60.51, 55.76, 18.12; HRMS (ESI) calcd for $C_{47}H_{46}O_{13}Na$ $[M + Na]^+$ 841.2836, found 841.2828.

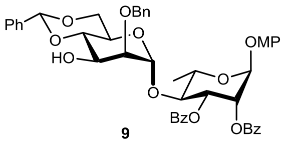

9

S9: R_f = 0.30; $[\alpha]_D^{20}$ = 41.2 (*c* 8.0, $CHCl_3$); ^1H NMR (600 MHz, $CDCl_3$) δ 8.28–8.23 (m, 2H), 8.13–8.09 (m, 2H), 7.80–7.74 (m, 1H), 7.63 (m, 3H), 7.58–7.40 (m, 6H), 7.43 (s, 4H), 7.24–7.19 (m, 2H), 7.05–6.98 (m, 2H), 6.06 (dd, *J* = 9.6, 3.6 Hz, 1H), 5.93 (dd, *J* = 3.7, 1.8 Hz, 1H), 5.67 (d, *J* = 1.8 Hz, 1H), 5.46 (s, 1H), 5.01 (d, *J* = 1.6 Hz, 1H), 4.96 (d, *J* = 12.2 Hz, 1H), 4.80 (d, *J* = 12.0 Hz, 1H), 4.27–4.17 (m, 1H), 4.15 (dd, *J* = 10.0, 3.4 Hz, 1H), 4.05 (t, *J* = 9.5 Hz, 1H), 3.96 (t, *J* = 9.7 Hz, 1H), 3.93 (dd, *J* = 3.6, 1.5 Hz, 1H), 3.88 (s, 3H), 3.84 (td, *J* = 9.8, 4.8 Hz, 1H), 3.76 (dd, *J* = 10.0, 4.8 Hz, 1H), 3.55 (t, *J* = 10.1 Hz, 1H), 3.07 (s, 1H), 1.32 (d, *J* = 6.2 Hz, 3H); ^{13}C NMR (151 MHz, $CDCl_3$) δ 165.75, 165.57, 155.40, 150.13, 137.90, 137.53, 133.85, 133.50, 133.42, 130.04, 129.94, 129.71, 129.61, 129.19, 128.84, 128.79, 128.61, 128.57, 128.37, 128.26, 126.62, 117.91, 114.87, 102.09, 100.83, 96.53, 79.49, 79.27, 78.25, 78.12, 77.74, 77.53, 77.32, 74.22, 70.90, 70.39, 68.60, 68.41, 68.16, 64.51, 60.57, 55.75, 18.32; HRMS (ESI) calcd for $C_{47}H_{50}NO_{13}$ $[M + NH_4]^+$ 836.3277, found 836.3277.

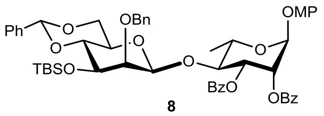

8

To a solution of **8**[A] (7.22 g, 8.8 mmol) and imidazole (4.20 g, 61.7 mmol) in anhydrous CH_2Cl_2 (44 mL), was added TBSCl (6.64 g, 44.1 mmol). The resulting solution was stirred at rt overnight, and was then quenched with CH_3OH. The mixture was diluted with EtOAc, and then washed with water, sat. aq. $NaHCO_3$, and brine, respectively. The organic layer was dried over anhydrous Na_2SO_4 and concentrated *in vacuo*. The residue was purified by silica gel column chromatography (petroleum ether/EtOAc, 8:1) to give **8** (7.88 g, 96%) as a white foam. R_f = 0.40; $[\alpha]_D^{20}$ = -28.1 (*c* 1.0, $CHCl_3$); ^1H NMR (500 MHz, $CDCl_3$) δ 8.10–8.04 (m, 2H), 7.95–7.91 (m, 2H), 7.67–7.62 (m, 1H), 7.59–7.55 (m, 1H), 7.51 (t, *J* = 7.7 Hz, 2H), 7.46–7.38 (m, 6H), 7.36–7.26 (m, 6H), 7.14–7.09 (m, 2H), 6.90–6.86 (m, 2H),

5.78–5.74 (m, 2H), 5.53–5.51 (m, 2H), 4.74 (d, J = 2.8 Hz, 2H), 4.68 (s, 1H), 4.31 (dd, J = 10.4, 4.8 Hz, 1H), 4.07 (m, 1H), 4.00–3.95 (m, 1H), 3.92–3.85 (m, 2H), 3.80 (s, 3H), 3.48–3.45 (m, 2H), 3.28 (td, J = 9.7, 4.8 Hz, 1H), 1.44 (d, J = 6.2 Hz, 3H), 0.73 (s, 9H), -0.21 (s, 3H), -0.27 (s, 3H); ^{13}C NMR (126 MHz, $CDCl_3$) δ 165.59, 165.14, 155.38, 150.40, 138.85, 137.61, 133.70, 133.65, 129.94, 129.62, 129.53, 128.95, 128.88, 128.75, 128.24, 128.18, 128.16, 127.54, 126.26, 118.05, 114.83, 102.93, 101.89, 96.94, 80.19, 78.84, 78.70, 75.85, 72.93, 72.43, 71.04, 68.67, 68.19, 67.88, 55.81, 25.81, 18.26, 18.12, -4.65, -5.02; HRMS (ESI) calcd for $C_{53}H_{60}O_{13}Si$ $[M + Na]^+$ 955.3698, found 955.3692.

Disaccharide donor 8^D

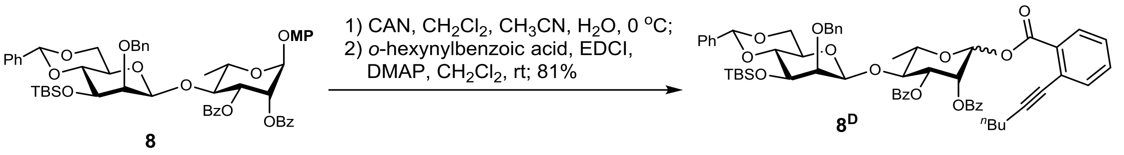

To a solution of **8** (7.65 g, 8.20 mmol) in CH_2Cl_2/MeCN/H_2O (133 mL/240 mL/67 mL), was added ammonium ceric nitrate (18.0 g, 32.80 mmol) at 0 °C. The mixture was stirred at 0 °C for 30 min. The mixture was diluted with EtOAc and washed with sat. aq. $NaHCO_3$ and brine, respectively. The organic layer was dried over anhydrous Na_2SO_4 and concentrated *in vacuo*. The residue was purified by silica gel column chromatography (petroleum ether/EtOAc, 8:1) to give a reddish foam (6.92 g).

To a mixture of the residue above (6.92 g, 8.37 mmol), *ortho*-hexynylbenzoic acid (2.54 g, 12.60 mmol), EDCI (2.40 g, 12.60 mmol), and DMAP (1.02 g, 8.37 mmol), was added anhydrous CH_2Cl_2 (160 mL). After being stirred at rt for 2 h, the mixture was diluted with EtOAc, and was then washed with water, sat. aq. $NaHCO_3$, and brine, respectively. The organic layer was dried over anhydrous Na_2SO_4 and concentrated *in vacuo*. The residue was purified by silica gel column chromatography (petroleum ether/EtOAc, 8:1) to give **8^D** (6.85 g, 83% for two steps, α/β = 1.6:1) as a white foam. R_f = 0.40; ^{1}H NMR (500 MHz, $CDCl_3$) δ 8.17–8.00 (m, 3H), 8.00–7.88 (m, 2H), 7.74 (d, J = 7.9 Hz, 0.38H), 7.69–7.20 (m, 19H), 7.15–7.09 (m, 0.38H), 6.51 (d, J = 2.0 Hz, 0.60H), 6.28 (s, 0.38H), 5.98 (d, J = 3.4 Hz, 0.38H), 5.81 (dd, J = 3.5, 2.0 Hz, 0.60H), 5.76 (dd, J = 9.6, 3.5 Hz, 0.60H), 5.54 (s, 1H), 5.45 (dd, J = 9.6, 3.3 Hz, 0.38H), 4.79 (d, J = 11.6 Hz, 1H), 4.74 (t, J = 10.0 Hz, 1H), 4.70 (d, J = 8.2 Hz, 1H), 4.33 (dt, J = 9.7, 4.7 Hz, 1H), 4.20–4.10 (m, 1H), 4.02

(m, 1H), 3.96–3.86 (m, 2H), 3.86–3.78 (m, 0.34H), 3.54–3.46 (m, 2H), 3.29 (tt, J = 9.6, 4.7 Hz, 1H), 2.60–2.44 (m, 2H), 1.70–1.47 (m, 7H), 0.97 (m, 3H), 0.78–0.74 (m, 9H), -0.14– -0.19 (m, 3H), -0.21– -0.25 (m, 3H); ^{13}C NMR (126 MHz, $CDCl_3$) δ 165.72, 165.22, 164.99, 164.02, 163.23, 138.77, 138.69, 137.48, 134.78, 134.41, 133.67, 133.61, 133.50, 132.28, 132.17, 130.55, 130.53, 129.95, 129.88, 129.69, 129.48, 129.39, 129.34, 129.27, 129.11, 128.85, 128.79, 128.77, 128.67, 128.62, 128.15, 128.12, 128.07, 128.03, 127.91, 127.82, 127.53, 127.40, 127.31, 126.96, 126.32, 126.16, 126.15, 125.71, 125.32, 102.72, 102.66, 101.80, 97.20, 97.17, 91.75, 91.02, 80.19, 80.14, 79.28, 78.92, 78.60, 78.52, 78.28, 78.19, 77.36, 77.11, 76.85, 75.84, 75.72, 73.89, 72.94, 72.84, 72.47, 72.20, 70.14, 69.98, 68.52, 67.80, 67.74, 30.71, 25.90, 25.70, 22.11, 19.58, 19.56, 18.14, 18.11, 18.06, 13.74, 13.69, -4.71, -4.73, -5.13, -5.14; HRMS (ESI) calcd for $C_{59}H_{66}O_{13}SiNa$ $[M + Na]^+$ 1033.4165, found 1033.4172.

2-mer 1

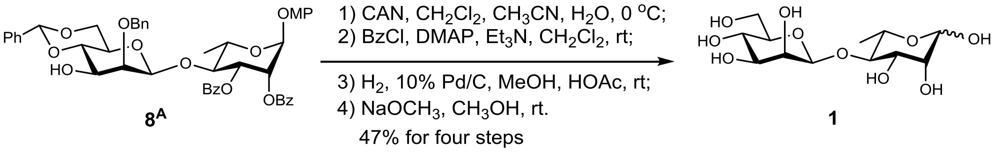

A solution of disaccharide **8^A^** (181 mg, 0.19 mmol) in MeCN (6 mL) was stirred at 0 °C. Ammonium ceric nitrate (531 mg, 0.97 mmol) was dissolved in H_2O (1.5 mL) at 0 °C. The latter solution was added into the former via syringe. The mixture was stirred at 0 °C for 2 min, and was then diluted with CH_2Cl_2 and washed with sat. aq. $NaHCO_3$ and brine, respectively. The organic layer was dried over anhydrous Na_2SO_4 and concentrated *in vacuo*. The residue was used in the next step without further purification.

In order to prevent undesired reactions of the resultant aldehyde, especially in the presence of high loading of the Pd catalyst and long duration of the hydrogenolysis, an additional transformation was performed to protect the hemiacetal with a benzoyl group.

Thus, to a solution of the residue above, DMAP (2 mg, 17.0 μmol), Et_3N (0.237 mL, 1.71 mmol) in CH_2Cl_2 (1 mL), was added BzCl (49.4 μL, 0.43 mmol). The mixture was stirred at rt for 3.5 h, and was then diluted with CH_2Cl_2 and washed with sat. aq. $NaHCO_3$,

and brine, respectively. The organic layer was dried over anhydrous Na_2SO_4 and concentrated *in vacuo*. The residue was purified by silica gel column chromatography (petroleum ether/EtOAc, 3:1) to give a yellowish foam (146 mg).

The foam above was dissolved in a mixed solvent of MeOH/HOAc (7 mL/1 drop) containing 10% Pd/C (100 mg, wetted with 55% H_2O). The mixture was stirred under H_2 atmosphere (1 atm) at rt for 24 h, and was then filtrated through a pad of Celite. The filtrate was concentrated. The residue was purified by silica gel column chromatography ($MeOH/CH_2Cl_2$, 1:20) to give a yellowish foam (78 mg).

The residue above (48 mg) was added into $NaOCH_3/CH_3OH$ (3 mL, pH = ~11). The mixture was stirred at rt for 24 h, and was then neutralized with Amberlyst $15H^+$ resin and filtered. The filtrate was concentrated *in vacuo*. The residue was purified by gel filtration (Sephadex LH–20, $MeOH/H_2O$, 1:1) to afford disaccharide **1** (23 mg, 47% for four steps; α/β = 1.7:1 based on ^1H NMR analysis) as a glassy solid or white powder after lyophilization. ^1H NMR (500 MHz, D_2O) δ 5.12 (d, *J* = 1.4 Hz, 0.62H), 4.93–4.89 (m, 1H), 4.87 (d, *J* = 1.1 Hz, 0.36H), 4.11–4.06 (m, 1H), 3.99–3.90 (m, 3H), 3.80–3.74 (m, 1.36H), 3.73–3.56 (m, 3H), 3.50 (dq, *J* = 9.4, 6.1 Hz, 0.36H), 3.42–3.34 (m, 1.36H), 1.35 (m, 3H); ^{13}C NMR (126 MHz, D_2O) δ 101.75, 101.72, 95.03, 94.60, 80.84, 80.39, 77.38, 74.23, 74.04, 72.80, 72.27, 71.91, 71.80, 71.21, 68.22, 67.99, 62.20, 18.27, 18.24; HRMS (ESI) calcd for $C_{12}H_{22}O_{10}Na$ $[M + Na]^+$ 349.1111, found 349.1106.

Preparation of 4-mer 9, donor 9^D, acceptor 9^A, and 4-mer 2

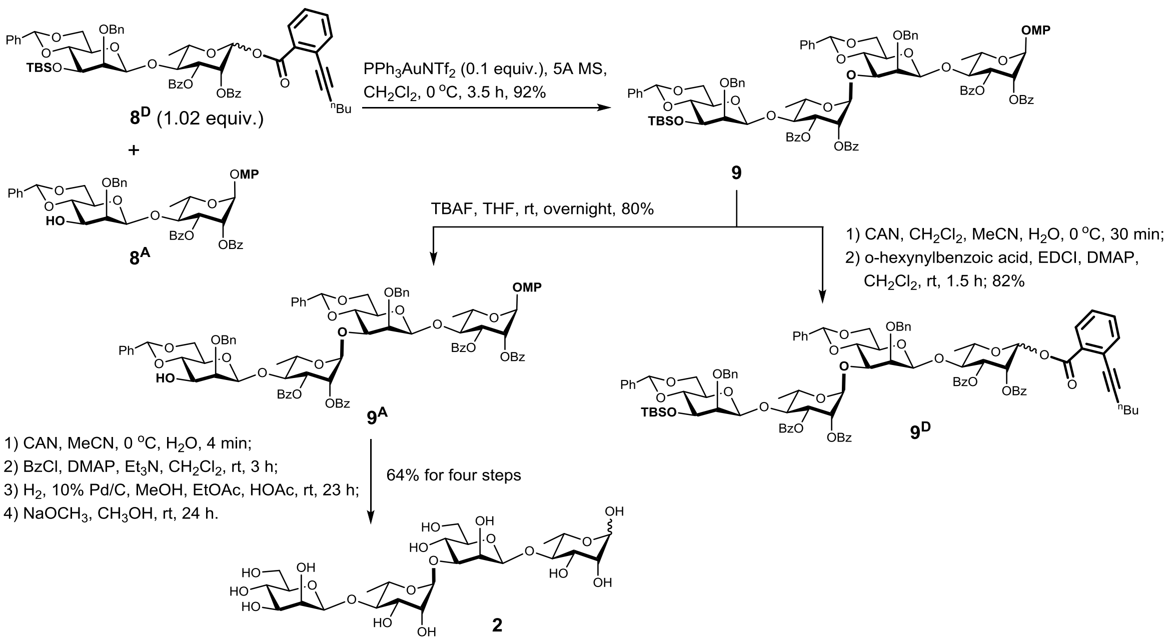

4-mer 9

A mixture of disaccharide donor **8^D^** (6.85 g, 6.77 mmol) and acceptor **8^A^** (5.65 g, 6.90 mmol) in a Schlenk flask equipped with a Teflon-coated magnetic stir bar was dried in high vacuum at 40 °C for 8 h, and then 5Å MS (10 g) and anhydrous CH_2Cl_2 (100 mL) were added. The mixture was stirred at rt for 15 min, and was then cooled to 0 °C, to which $Ph_3PAuNTf_2$ (500 mg, 0.68 mmol) was added. The resulting mixture was stirred at 0 °C for 3.5 h. After addition of Ph_3P (500 mg) and Et_3N (0.5 mL), the mixture was filtrated through a pad of Celite. The filtrate was concentrated *in vacuo* to give a residue, which was purified by silica gel column chromatography (petroleum ether/EtOAc, 3:1) to give 4-mer **9** (9.74 g, 92%) as a white foam. R_f = 0.30; $[\alpha]_D^{25}$ = -30.5 (*c* 1.0, $CHCl_3$); ^{1}H NMR (500 MHz, $CDCl_3$) δ 8.05–7.98 (m, 4H), 7.87–7.79 (m, 4H), 7.64–7.53 (m, 2H), 7.53–7.37 (m, 11H), 7.36–7.22 (m, 9H), 7.22–7.14 (m, 3H), 7.09 (m, 3H), 6.88–6.77 (m, 2H), 5.77 (dd, *J* = 3.5, 1.9 Hz, 1H), 5.68 (dd, *J* = 9.6, 3.4 Hz, 1H), 5.62–5.55 (m, 2H), 5.49 (d, *J* = 1.9 Hz, 1H), 5.46 (s, 1H), 5.28 (dd, *J* = 3.3, 1.7 Hz, 1H), 4.76 (s, 2H), 4.65 (s, 1H), 4.60–4.53 (m, 2H), 4.45 (d, *J* = 11.9 Hz, 1H), 4.32 (dd, *J* = 10.4, 4.7 Hz, 1H), 4.26–4.18 (m, 2H), 4.15–4.02 (m, 3H), 4.01–3.88 (m, 2H), 3.86–3.74 (m, 3H), 3.73 (s, 3H), 3.60 (d, *J* = 3.1 Hz, 1H), 3.53 (dd, *J* = 10.0, 3.1 Hz, 1H), 3.38–3.30 (m, 3H), 3.18 (td, *J* = 9.7, 4.9 Hz, 1H), 1.44 (d, *J* = 6.2 Hz, 3H), 1.07 (d, *J* = 6.2 Hz, 3H), 0.64 (s, 9H), -0.31 (s, 3H), -0.39 (s, 3H); ^{13}C NMR (126 MHz, $CDCl_3$) δ 165.48, 165.12, 165.10, 164.83, 155.33, 150.32, 138.54, 137.99,

137.55, 137.26, 133.64, 133.55, 133.45, 129.85, 129.76, 129.71, 129.53, 129.51, 129.39, 129.34, 129.30, 129.00, 128.87, 128.72, 128.68, 128.34, 128.28, 128.16, 128.09, 127.99, 127.78, 127.35, 126.18, 126.00, 117.99, 114.77, 103.24, 103.21, 101.79, 96.89, 95.10, 79.44, 79.36, 78.73, 78.61, 76.38, 75.97, 75.72, 75.44, 75.14, 72.70, 72.53, 72.21, 71.23, 71.00, 68.61, 68.55, 68.14, 67.86, 67.47, 55.70, 25.72, 18.16, 18.08, 17.46, -4.84, -5.09; HRMS (ESI) calcd for $C_{93}H_{98}O_{24}SiNa$ $[M + Na]^+$ 1649.6110, found 1649.6106.

4-mer donor $\mathbf{9^D}$

A solution of 4-mer **9** (5.35 g, 3.28 mmol) in CH_2Cl_2/MeCN (54 mL/100 mL) was stirred at 0 °C. Ammonium ceric nitrate (7.20 g, 13.1 mmol) was dissolved in H_2O (27 mL) at 0 °C. The latter solution was added into the former via syringe, and the mixture was stirred at 0 °C for 30 min. The mixture was diluted with CH_2Cl_2 and washed with sat. aq. $NaHCO_3$ and brine, respectively. The organic layer was dried over anhydrous Na_2SO_4 and concentrated *in vacuo*. The residue was purified by silica gel column chromatography (petroleum ether/EtOAc, 1:3) to give the corresponding hemiacetal (4.46 g, 92%) as a yellowish foam.

To a mixture of the hemiacetal (4.46 g, 2.93 mmol), EDCI (0.84 g, 4.39 mmol), DMAP (0.36 g, 2.93 mmol), and *ortho*-hexynylbenzoic acid (0.89 g, 4.39 mmol), was added CH_2Cl_2 (58 mL). The mixture was stirred at rt for 1.5 h, and was then diluted with CH_2Cl_2 and washed with sat. aq. $NaHCO_3$. The organic layer was dried over anhydrous Na_2SO_4 and concentrated *in vacuo*. The residue was purified by silica gel column chromatography (petroleum ether/EtOAc, 3:1) to give $\mathbf{9^D}$ (4.10 g, 82% for two steps; α/β = 1.1:1) as a white foam. R_f = 0.50; ^1H NMR (500 MHz, $CDCl_3$) δ 8.16–8.00 (m, 5H), 7.90–7.86 (m, 2H), 7.85–7.81 (m, 2H), 7.73–7.58 (m, 3H), 7.56–7.47 (m, 6H), 7.47–7.40 (m, 7H), 7.40–7.34 (m, 3H), 7.34–7.29 (m, 4H), 7.28 (d, *J* = 7.7 Hz, 1H), 7.25–7.18 (m, 9H), 7.17–7.08 (m, 2H), 6.47 (d, *J* = 2.0 Hz, 0.52H), 6.26 (d, *J* = 1.2 Hz, 0.47H), 5.97 (dd, *J* = 3.2, 1.1 Hz, 0.47H), 5.79 (dd, *J* = 3.4, 2.0 Hz, 0.52H), 5.67–5.57 (m, 3H), 5.50 (s, 1H), 5.34 (dd, *J* = 9.6, 3.2 Hz, 0.47H), 5.32–5.28 (m, 1H), 4.77 (s, 2H), 4.65 (d, *J* = 7.0 Hz, 1H), 4.59 (d, *J* = 12.5 Hz, 2H), 4.48 (dd, *J* = 11.9, 2.2 Hz, 1H), 4.37–4.32 (m, 1H), 4.30–4.24 (m, 2H), 4.17–4.08 (m, 1H), 4.07–3.91 (m, 2H), 3.89–3.74 (m, 4H), 3.63 (dd, *J* = 5.5, 3.0 Hz, 1H), 3.58–3.53 (m, 1H), 3.41–3.31 (m, 3H), 3.21 (td, *J* = 9.7, 4.8 Hz, 1H), 2.55–2.50

(m, 1.47H), 2.46 (t, *J* = 7.1 Hz, 0.52H), 1.68–1.43 (m, 8H), 1.11–1.05 (m, 3H), 1.00–0.89 (m, 3H), 0.67 (s, 9H), -0.28 (s, 3H), -0.35 (br s, 3H); ^{13}C NMR (126 MHz, $CDCl_3$) δ 165.31, 165.25, 165.12, 164.90, 164.09, 162.13, 138.64, 138.14, 137.64, 137.31, 134.90, 133.78, 133.72, 133.68, 133.53, 132.41, 130.64, 130.08, 130.01, 129.83, 129.78, 129.47, 129.45, 129.38, 129.29, 129.10, 128.95, 128.82, 128.80, 128.77, 128.62, 128.46, 128.37, 128.21, 128.18, 128.06, 127.90, 127.80, 127.43, 127.41, 127.07, 126.25, 126.07, 126.06, 103.30, 103.05, 101.87, 97.34, 97.34, 97.00, 95.20, 91.83, 91.60, 79.53, 79.36, 78.71, 78.05, 76.47, 76.20, 75.85, 75.73, 75.65, 75.22, 72.78, 72.45, 72.24, 71.37, 70.28, 70.13, 68.79, 68.70, 68.61, 68.23, 67.95, 67.59, 30.84, 30.82, 25.79, 22.24, 22.22, 21.20, 19.71, 19.68, 18.24, 18.19, 17.52, 14.35, 13.85, 13.81, -4.77, -5.02; MALDI-TOF MS calcd for $C_{99}H_{104}O_{24}SiNa$ $[M + Na]^+$ 1727.6579, found 1727.6583.

4-mer acceptor 9^{A}

To a solution of 4-mer **9** (3.53 g, 2.17 mmol) in anhydrous THF (70 mL), was added TBAF (1.0 M in THF, 4.33 mL, 4.33 mmol). The mixture was stirred at rt overnight, and was then diluted with CH_2Cl_2, and washed with sat. aq. NH_4Cl and brine, respectively. The organic layer was dried over anhydrous Na_2SO_4 and concentrated *in vacuo*. The residue was purified by silica gel column chromatography (petroleum ether/EtOAc, 2:1) to give **9^{A}** (2.61 g, 80%) as a white foam. R_f = 0.50; $[\alpha]_D^{25}$ = -29.3 (*c* 1.0, $CHCl_3$); ^{1}H NMR (500 MHz, $CDCl_3$) δ 8.11–8.03 (m, 5H), 7.96–7.84 (m, 5H), 7.72–7.60 (m, 2H), 7.59–7.49 (m, 6H), 7.47–7.42 (m, 6H), 7.41–7.37 (m, 2H), 7.36–7.31 (m, 7H), 7.30–7.22 (m, 4H), 7.21–7.11 (m, 6H), 6.92–6.85 (m, 2H), 5.84–5.79 (m, 1H), 5.71 (td, *J* = 9.8, 3.4 Hz, 2H), 5.60 (s, 1H), 5.54 (d, *J* = 1.8 Hz, 1H), 5.52–5.48 (m, 1H), 5.34–5.31 (m, 1H), 4.82–4.75 (m, 2H), 4.69 (d, *J* = 6.0 Hz, 2H), 4.66–4.62 (m, 1H), 4.38–4.29 (m, 2H), 4.28–4.22 (m, 2H), 4.17–4.10 (m, 3H), 4.04–3.93 (m, 3H), 3.90–3.77 (m, 6H), 3.72–3.67 (m, 2H), 3.64 (d, *J* = 3.1 Hz, 1H), 3.57 (dd, *J* = 10.1, 3.1 Hz, 1H), 3.49 (d, *J* = 3.8 Hz, 1H), 3.44–3.36 (m, 1H), 3.34–3.28 (m, 1H), 3.22 (td, *J* = 9.6, 4.8 Hz, 1H), 1.48 (d, *J* = 6.2 Hz, 3H), 1.13 (d, *J* = 6.2 Hz, 3H); ^{13}C NMR (126 MHz, $CDCl_3$) δ 165.53, 165.20, 165.15, 165.13, 155.39, 150.39, 138.04, 137.99, 137.40, 137.23, 133.70, 133.68, 133.65, 133.59, 129.91, 129.83, 129.77, 129.58, 129.51, 129.47, 129.41, 129.36, 129.19, 129.07, 128.83, 128.80, 128.77, 128.73, 128.72, 128.69, 128.62, 128.57, 128.39, 128.33, 128.28, 128.01, 127.85, 126.33, 126.30,

126.11, 118.05, 114.82, 103.28, 102.80, 102.03, 101.97, 96.95, 94.94, 79.46, 79.33, 78.21, 78.06, 76.39, 75.73, 75.32, 75.31, 72.56, 72.12, 71.22, 71.05, 70.42, 68.64, 68.59, 68.52, 68.20, 68.17, 67.31, 67.23, 67.22, 55.79, 18.13, 17.62; MALDI-TOF MS calcd for $C_{87}H_{84}O_{24}Na$ $[M + Na]^+$ 1535.5245, found 1535.5260.

4-mer 2

A solution of 4-mer $\mathbf{9^A}$ (170 mg, 0.11 mmol) in MeCN (10 mL) was stirred at 0 ^oC. Ammonium ceric nitrate (607 mg, 1.11 mmol) was dissolved in H_2O (1.2 mL) at 0 ^oC. The latter solution was added into the former via syringe. The mixture was stirred at 0 ^oC for 4 min, and was then diluted with CH_2Cl_2 and washed with sat. aq. $NaHCO_3$ and brine, respectively. The organic layer was dried over anhydrous Na_2SO_4 and concentrated *in vacuo*. The residue was used in the next step without further purification.

To a solution of the residue above, DMAP (1.36 mg, 11.2 μmol), Et_3N (0.155 mL, 1.12 mmol) in CH_2Cl_2 (1.5 mL), was added BzCl (65 μL, 0.56 mmol). The mixture was stirred at rt for 3 h, and was then diluted with CH_2Cl_2 and washed with sat. aq. $NaHCO_3$ and brine, respectively. The organic layer was dried over anhydrous Na_2SO_4 and concentrated *in vacuo*. The residue was purified by silica gel column chromatography (petroleum ether/EtOAc, 2:1) to give a yellowish foam (143 mg).

The foam above (143 mg) was dissolved in a mixed solvent of MeOH/EtOAc/HOAc (2 mL/2 mL/0.2 mL) containing 10% Pd/C (200 mg, wetted with 55% H_2O). The mixture was stirred under H_2 atmosphere (1 atm) at rt for 23 h, and was then filtrated through a pad of Celite, and the Celite pad was washed with acetone/MeOH (1:1, v/v) three times. The filtrates were concentrated *in vacuo*. The resulting residue was purified by silica gel column chromatography (MeOH/CH_2Cl_2, 1:15) to give a glassy solid (100 mg).

The residue above (100 mg) was added into a $NaOCH_3/CH_3OH$ solution (5 mL, pH = ~11). The mixture was stirred at rt for 24 h, and was then neutralized with Amberlyst 15 H^+ resin and filtered. The filtrate was concentrated *in vacuo*. The residue was purified by gel filtration (Sephadex LH–20, MeOH/H_2O, 1:1) to afford 4-mer **2** (46 mg, 64% for four steps; $\alpha/\beta = 1.7:1$ based on ^1H NMR analysis) as a glassy solid or white powder after lyophilization. ^1H NMR (400 MHz, D_2O) δ 5.09 (s, 0.63H), 4.96 (s, 1H), 4.88 (s, 2H), 4.84 (s, 0.35H), 4.27 (t, J = 3.3 Hz, 1H), 4.14–3.82 (m, 8.35H), 3.82–3.42 (m, 7H), 3.42–3.26

(m, 2.63H), 1.32 (d, J = 6.5 Hz, 6H); ^{13}C NMR (101 MHz, D_2O) δ 100.58, 100.47, 96.15, 93.81, 93.39, 79.80, 79.43, 76.87, 76.19, 76.07, 73.01, 72.84, 71.58, 71.04, 70.66, 70.56, 70.20, 70.01, 67.46, 66.97, 66.78, 66.62, 64.94, 60.97, 17.09, 16.85; HRMS (ESI) calcd for $C_{24}H_{42}O_{19}Na$ $[M + Na]^+$ 657.2218, found 657.2214.

Preparation of 8-mer 10, donor 10^D, acceptor 10^A, and 8-mer 3

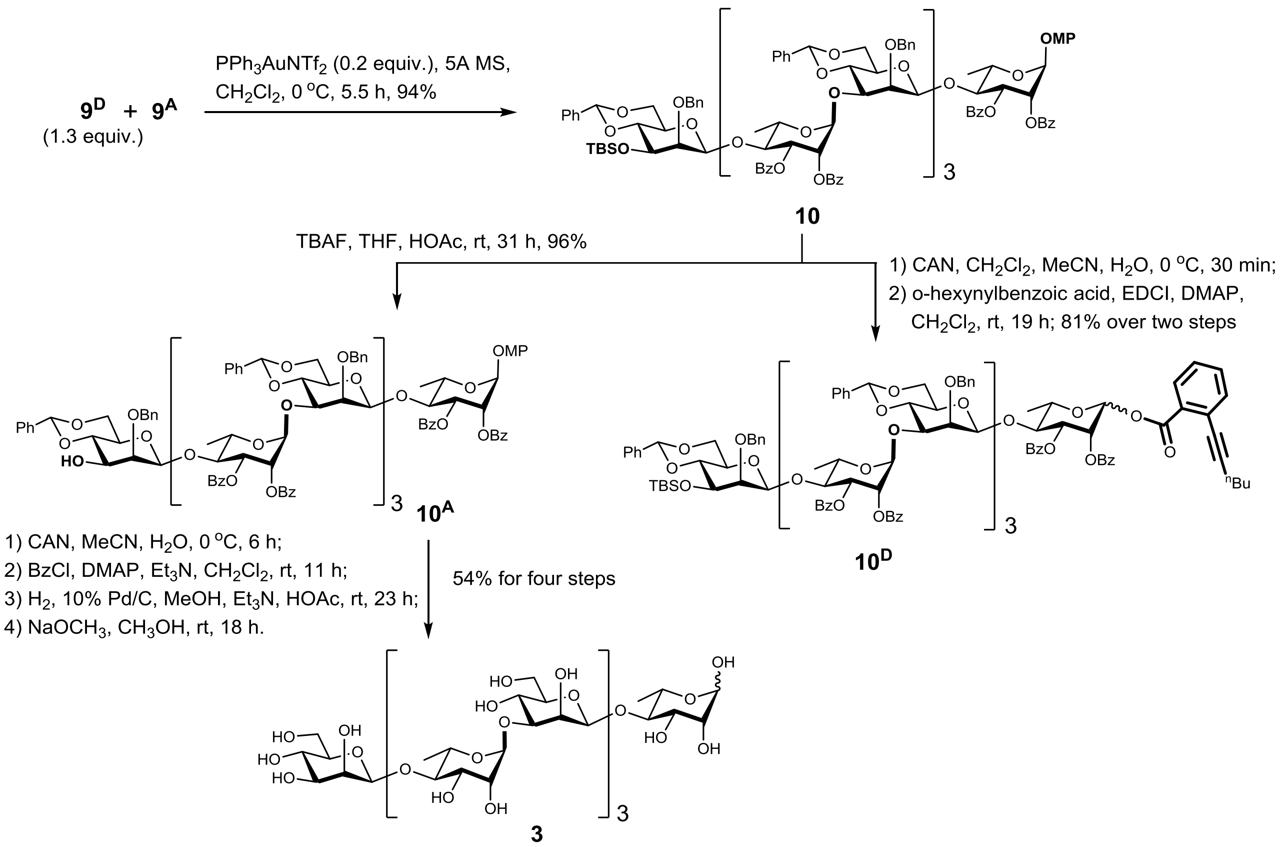

8-mer 10

A mixture of 4-mer donor $\mathbf{9^D}$ (3.28 g, 1.92 mmol) and acceptor $\mathbf{9^A}$ (2.24 g, 1.48 mmol) in a Schlenk flask equipped with a Teflon-coated magnetic stir bar was dried in high vacuum at 40 °C overnight, and then 5Å MS (5.5 g) and anhydrous CH_2Cl_2 (55 mL) were added. The mixture was stirred at rt for 15 min, and was then cooled to 0 °C, to which $Ph_3PAuNTf_2$ (218 mg, 0.29 mmol) was added. The resulting mixture was stirred at 0 °C for 5.5 h. After addition of Ph_3P (218 mg) and Et_3N (0.22 mL), the resulting mixture was filtrated through a pad of Celite. The filtrate was concentrated *in vacuo* to give a residue, which was purified by silica gel column chromatography (petroleum ether/EtOAc, 2:1) to give 8-mer **10** (4.19 g, 94%) as a white foam. R_f = 0.50; $[\alpha]_D^{25}$ = 9.2 (*c* 1.0, $CHCl_3$); ^1H

NMR (600 MHz, $CDCl_3$) δ 8.06–7.98 (m, 10H), 7.88–7.83 (m, 6H), 7.79–7.75 (m, 4H), 7.68–7.60 (m, 6H), 7.55–7.48 (m, 12H), 7.45–7.39 (m, 12H), 7.37–7.34 (m, 2H), 7.33–7.26 (m, 9H), 7.23–7.17 (m, 25H), 7.13–7.10 (m, 3H), 7.08–7.03 (m, 2H), 6.88–6.85 (m, 2H), 5.79 (dd, *J* = 3.4, 1.9 Hz, 1H), 5.69–5.66 (m, 1H), 5.62–5.57 (m, 3H), 5.53 (t, *J* = 3.0 Hz, 1H), 5.51 (d, *J* = 1.8 Hz, 2H), 5.49–5.47 (m, 2H), 5.33 (dd, *J* = 3.4, 1.7 Hz, 1H), 5.24 (dd, *J* = 3.4, 1.7 Hz, 1H), 5.19 (dd, *J* = 3.4, 1.7 Hz, 1H), 4.78–4.75 (m, 2H), 4.68–4.60 (m, 2H), 4.57–4.49 (m, 6H), 4.46–4.41 (m, 3H), 4.35–4.25 (m, 3H), 4.24–4.18 (m, 2H), 4.16–4.03 (m, 5H), 4.00–3.88 (m, 6H), 3.84–3.75 (m, 8H), 3.74–3.68 (m, 2H), 3.63–3.53 (m, 2H), 3.48–3.42 (m, 3H), 3.40–3.14 (m, 6H), 1.45 (dd, *J* = 6.1, 1.9 Hz, 3H), 1.11 (d, *J* = 6.3 Hz, 3H), 1.07 (d, *J* = 6.1 Hz, 3H), 1.02 (d, *J* = 6.2 Hz, 3H), 0.65 (s, 9H), -0.31 (s, 3H), -0.38 (s, 3H); ^{13}C NMR (151 MHz, $CDCl_3$) δ 165.55, 165.16, 165.13, 165.04, 165.01, 164.95, 164.91, 164.85, 155.34, 150.39, 138.59, 138.03, 138.00, 137.96, 137.70, 137.68, 137.59, 137.38, 137.34, 137.31, 137.28, 137.21, 134.59, 133.71, 133.65, 133.62, 133.47, 133.42, 132.27, 132.20, 132.13, 129.93, 129.87, 129.86, 129.84, 129.82, 129.79, 129.74, 129.61, 129.55, 129.51, 129.47, 129.44, 129.37, 129.35, 129.29, 129.21, 129.12, 128.80, 128.78, 128.76, 128.74, 128.70, 128.67, 128.41, 128.39, 128.35, 128.32, 128.29, 128.28, 128.21, 128.15, 128.02, 127.86, 127.84, 127.74, 127.71, 127.36, 126.30, 126.30, 126.21, 126.10, 126.09, 126.06, 126.01, 118.02, 114.80, 103.56, 103.53, 103.29, 103.27, 102.81, 102.02, 101.99, 101.81, 96.91, 94.97, 94.91, 94.79, 79.47, 79.43, 79.39, 79.32, 79.20, 79.16, 78.73, 78.64, 76.41, 76.37, 75.75, 75.71, 75.53, 75.29, 75.23, 75.14, 74.99, 74.97, 74.38, 74.19, 72.70, 72.57, 72.43, 72.40, 72.28, 71.26, 71.15, 71.02, 68.65, 68.19, 68.16, 67.86, 67.46, 67.39, 67.35, 55.79, 25.76, 18.22, 18.14, 17.57, 17.47, -4.82, -5.06; MALDI-TOF MS calcd for $C_{173}H_{174}O_{46}SiNa$ $[M + Na]^+$ 3038.0938, found 3038.0818.

8-mer donor 10^{D}

A solution of 8-mer **10** (2.55 g, 0.85 mmol) in CH_2Cl_2/MeCN (18 mL/21 mL) was stirred at 0 ^{o}C. Ammonium ceric nitrate (1.85 g, 3.38 mmol) was dissolved in H_2O (7 mL) at 0 ^{o}C. The latter solution was added into the former via syringe, and the mixture was stirred at 0 ^{o}C for 30 min. The mixture was diluted with CH_2Cl_2 and washed with sat. aq. $NaHCO_3$ and brine, respectively. The organic layer was dried over anhydrous Na_2SO_4 and concentrated *in vacuo*. The residue was purified by silica gel column chromatography

(petroleum ether/EtOAc/CH_2Cl_2, 3:1:1) to give the corresponding hemiacetal (2.26 g, 92%) as a yellowish foam.

To a mixture of the hemiacetal (2.26 g, 0.78 mmol), EDCI (0.37 g, 1.94 mmol), DMAP (0.24 g, 1.94 mmol), and *ortho*-hexynylbenzoic acid (0.24 g, 1.17 mmol), was added CH_2Cl_2 (16 mL). The mixture was stirred at rt for 19 h, and was then diluted with CH_2Cl_2 and washed with sat. aq. $NaHCO_3$. The organic layer was dried over anhydrous Na_2SO_4 and concentrated *in vacuo*. The residue was purified by silica gel column chromatography (petroleum ether/EtOAc/CH_2Cl_2, 3:1:1) to give **10^{D}** (2.1 g, 81% for two steps; α/β = 1.5:1) as a white foam. R_f = 0.80; ^{1}H NMR (600 MHz, $CDCl_3$) δ 8.19–7.98 (m, 9H), 7.96–7.77 (m, 8H), 7.76–7.59 (m, 6H), 7.59–7.41 (m, 21H), 7.41–7.06 (m, 44H), 6.51 (d, *J* = 2.0 Hz, 0.6H), 6.30 (s, 0.4H), 6.01 (d, *J* = 3.3 Hz, 0.4H), 5.88–5.79 (m, 0.6H), 5.72–5.46 (m, 8H), 5.43–5.22 (m, 4H), 4.91–4.74 (m, 2H), 4.73–4.44 (m, 10H), 4.43–4.23 (m, 6H), 4.23–3.71 (m, 21H), 3.70–3.57 (m, 2.4H), 3.55–3.16 (m, 10.6H), 2.59–2.44 (m, 2H), 1.70–1.46 (m, 8H), 1.32–1.23 (m, 3.6H), 1.21–1.11 (m, 6.4H), 1.07 (d, *J* = 6.1 Hz, 3.4H), 1.03–0.83 (m, 6.6H), 0.69 (s, 9H), -0.27 (s, 3H), -0.34 (s, 3H); ^{13}C NMR (151 MHz, $CDCl_3$) δ 165.67, 165.17, 165.08, 165.06, 164.98, 164.95, 164.93, 164.90, 164.84, 164.79, 164.73, 163.95, 163.17, 138.50, 137.97, 137.90, 137.61, 137.59, 137.49, 137.21, 137.18, 134.76, 134.40, 133.64, 133.59, 133.54, 133.52, 133.47, 133.37, 133.33, 133.30, 132.27, 132.14, 130.51, 130.49, 129.94, 129.87, 129.73, 129.70, 129.68, 129.61, 129.52, 129.41, 129.38, 129.36, 129.32, 129.30, 129.24, 129.17, 129.14, 129.09, 128.98, 128.92, 128.80, 128.69, 128.66, 128.63, 128.59, 128.55, 128.51, 128.32, 128.29, 128.22, 128.20, 128.08, 128.03, 127.90, 127.77, 127.70, 127.67, 127.61, 127.58, 127.30, 127.23, 126.94, 126.13, 126.10, 125.97, 125.95, 125.90, 125.70, 125.35, 103.41, 103.16, 102.94, 101.91, 101.87, 101.70, 97.20, 97.16, 94.90, 94.87, 94.69, 91.68, 90.93, 79.35, 79.21, 79.05, 78.87, 78.65, 78.61, 78.56, 78.53, 76.31, 76.27, 75.82, 75.74, 75.71, 75.65, 75.43, 75.38, 75.28, 75.21, 75.03, 74.89, 74.88, 74.31, 74.14, 74.02, 72.60, 72.41, 72.31, 72.25, 72.16, 71.19, 71.05, 70.14, 69.97, 68.54, 68.49, 68.11, 68.09, 68.06, 68.03, 67.75, 67.39, 67.28, 67.25, 30.67, 25.63, 25.63, 22.07, 19.56, 18.09, 17.43, 17.35, 13.66, -4.95, -5.19; MALDI-TOF MS calcd for $C_{179}H_{180}O_{46}SiNa$ $[M + Na]^+$ 3116.1407, found 3116.1424.

8-mer acceptor 10^{A}

To a solution of 8-mer **10** (1.65 g, 0.55 mmol) in anhydrous THF (11 mL), were added HOAc (62 μL, 1.09 mmol) and TBAF (1.0 M in THF, 2.47 mL, 2.47 mmol). The mixture was stirred at rt for 31 h, and was then diluted with CH_2Cl_2 and washed with sat. aq. NH_4Cl and brine, respectively. The organic layer was dried over anhydrous Na_2SO_4 and concentrated *in vacuo*. The residue was purified by silica gel column chromatography (petroleum ether/EtOAc/CH_2Cl_2, 3:1:1) to give $\mathbf{10^A}$ (1.53 g, 96%) as a white foam. R_f = 0.45; $[\alpha]_D^{25}$ = 10.4 (*c* 1.0, $CHCl_3$); ^{1}H NMR (600 MHz, $CDCl_3$) δ 8.08–8.00 (m, 10H), 7.88–7.85 (m, 4H), 7.81–7.77 (m, 4H), 7.69–7.62 (m, 5H), 7.56–7.50 (m, 11H), 7.45–7.42 (m, 8H), 7.40–7.36 (m, 4H), 7.35–7.27 (m, 10H), 7.25–7.17 (m, 18H), 7.17–7.05 (m, 8H), 6.90–6.87 (m, 2H), 5.80 (dd, *J* = 3.4, 1.9 Hz, 1H), 5.69 (dd, *J* = 9.6, 3.4 Hz, 1H), 5.64–5.60 (m, 3H), 5.56 (s, 1H), 5.55–5.48 (m, 4H), 5.34 (dd, *J* = 3.4, 1.6 Hz, 1H), 5.26 (dd, *J* = 3.4, 1.6 Hz, 1H), 5.21 (dd, *J* = 3.4, 1.7 Hz, 1H), 4.78 (s, 2H), 4.68 (s, 1H), 4.62 (d, *J* = 11.9 Hz, 1H), 4.58–4.54 (m, 4H), 4.51 (s, 1H), 4.46 (d, *J* = 11.9 Hz, 1H), 4.41 (d, *J* = 12.0 Hz, 1H), 4.35 (dd, *J* = 10.4, 4.7 Hz, 1H), 4.32–4.26 (m, 3H), 4.22–4.20 (m, 2H), 4.17–3.88 (m, 13H), 3.82–3.76 (m, 7H), 3.73 (t, *J* = 9.7 Hz, 1H), 3.68–3.63 (m, 2H), 3.58 (dd, *J* = 10.0, 3.0 Hz, 1H), 3.48 (d, *J* = 3.2 Hz, 1H), 3.46–3.44 (m, 3H), 3.42–3.36 (m, 2H), 3.33–3.24 (m, 3H), 3.18 (td, *J* = 9.7, 5.0 Hz, 1H), 1.47 (d, *J* = 6.1 Hz, 3H), 1.13 (d, *J* = 6.2 Hz, 3H), 1.09 (d, *J* = 6.2 Hz, 3H), 1.06 (d, *J* = 6.2 Hz, 3H); ^{13}C NMR (151 MHz, $CDCl_3$) δ 165.56, 165.17, 165.14, 165.01, 164.96, 164.92, 155.35, 150.39, 138.07, 138.03, 138.01, 137.83, 137.67, 137.65, 137.39, 137.32, 137.21, 133.80, 133.72, 133.68, 133.65, 133.63, 133.47, 133.41, 129.93, 129.88, 129.83, 129.81, 129.75, 129.59, 129.56, 129.53, 129.48, 129.38, 129.36, 129.30, 129.21, 129.13, 129.10, 129.07, 128.88, 128.80, 128.78, 128.75, 128.71, 128.68, 128.64, 128.58, 128.50, 128.41, 128.39, 128.35, 128.33, 128.29, 128.24, 128.02, 127.84, 127.75, 126.32, 126.30, 126.14, 126.09, 126.07, 118.03, 114.80, 103.57, 103.27, 102.78, 102.03, 102.00, 101.94, 96.91, 94.96, 94.78, 79.40, 79.32, 79.25, 79.21, 78.16, 78.03, 76.42, 76.39, 76.34, 75.75, 75.72, 75.29, 75.26, 75.23, 74.96, 74.94, 74.21, 74.16, 72.57, 72.45, 72.41, 72.16, 71.26, 71.14, 71.08, 71.03, 70.36, 68.65, 68.58, 68.22, 68.20, 68.14, 67.47, 67.35, 67.18, 55.80, 18.15, 17.58, 17.56; MALDI-TOF MS calcd for $C_{167}H_{160}O_{46}Na$ $[M + Na]^+$ 2924.0073, found 2924.0062.

8-mer 3

A solution of **10^A^** (46.0 mg, 15.8 μmol) in MeCN (8 mL) was stirred at 0 °C. Ammonium ceric nitrate (43.5 mg, 79.0 μmol) was dissolved in H_2O (1.0 mL) at 0 °C. The latter solution was added into the former via syringe. The mixture was stirred at 0 °C for 6 h, and was then diluted with CH_2Cl_2 and washed with sat. aq. $NaHCO_3$ and brine, respectively. The organic layer was dried over anhydrous Na_2SO_4 and concentrated *in vacuo*. The residue was used in the next step without further purification.

To a solution of the residue above, DMAP (20.0 mg, 0.16 mmol), and Et_3N (21.9 μL, 0.16 mmol) in CH_2Cl_2 (1.5 mL), was added BzCl (9.10 μL, 78.9 μmol). The mixture was stirred at rt for 11 h, and was then diluted with CH_2Cl_2 and washed with sat. aq. $NaHCO_3$ and brine, respectively. The organic layer was dried over anhydrous Na_2SO_4 and concentrated *in vacuo*. The residue was purified by silica gel column chromatography (petroleum ether/EtOAc, 2:1) to give a yellowish foam (28 mg).

The foam above (22 mg) was dissolved in a mixture solvent of MeOH/EA/HOAc (2 mL/2 mL/0.2 mL) containing 10% Pd/C (206 mg, wetted with 55% H_2O). The resulting mixture was stirred under H_2 atmosphere (1 atm) at rt for 23 h. The mixture was filtrated through a pad of Celite, and the Celite pad was washed with acetone/MeOH (1:1, v/v) three times. The filtrates were concentrated. The resulting residue was purified by silica gel column chromatography (MeOH/CH_2Cl_2, 1:15) to give a glassy solid (18.4 mg).

The residue above (18.4 mg) was added into a $NaOCH_3$/CH_3OH solution (3 mL, pH = ~11). The mixture was stirred at rt for 18 h, and was then neutralized by Amberlyst 15 H^+ resin and filtered. The filtrate was concentrated *in vacuo*. The residue was purified by gel filtration (Sephadex LH–20, MeOH/H_2O, 1:1) to afford 8-mer **3** (14.7 mg, 54% for four steps; α/β = 1.5:1 based on ^{1}H NMR analysis) as a glassy solid or white powder after lyophilization. ^{1}H NMR (600 MHz, D_2O) δ 5.10 (s, 0.6H), 4.97 (s, 3H), 4.89 (s, 4H), 4.86 (s, 0.4H), 4.30–4.27 (m, 3H), 4.07 (d, *J* = 3.1 Hz, 1H), 4.06–3.88 (m, 16.4H), 3.76 (m, 4.6H), 3.73–3.62 (m, 11.4H), 3.61–3.55 (m, 1.4H), 3.52–3.45 (m, 0.6H), 3.43–3.33 (m, 4.6H), 1.33 (d, *J* = 6.3 Hz, 12H); ^{13}C NMR (151 MHz, D_2O) δ 100.59, 100.51, 100.49, 100.45, 96.17, 93.82, 93.40, 79.82, 79.63, 79.45, 79.37, 76.90, 76.88, 76.20, 76.11, 76.09, 73.02, 72.85, 71.59, 71.05, 70.67, 70.57, 70.55, 70.21, 70.02, 67.47, 67.42, 66.98, 66.79, 66.66, 66.63, 66.60, 64.96, 61.00, 60.96, 17.08, 17.06, 16.85; HRMS (ESI) calcd for $C_{48}H_{82}O_{37}Na$ $[M + Na]^+$ 1273.4433, found 1273.4429.

Preparation of 16-mer 11, donor 11^D, acceptor 11^A, and 16-mer 4

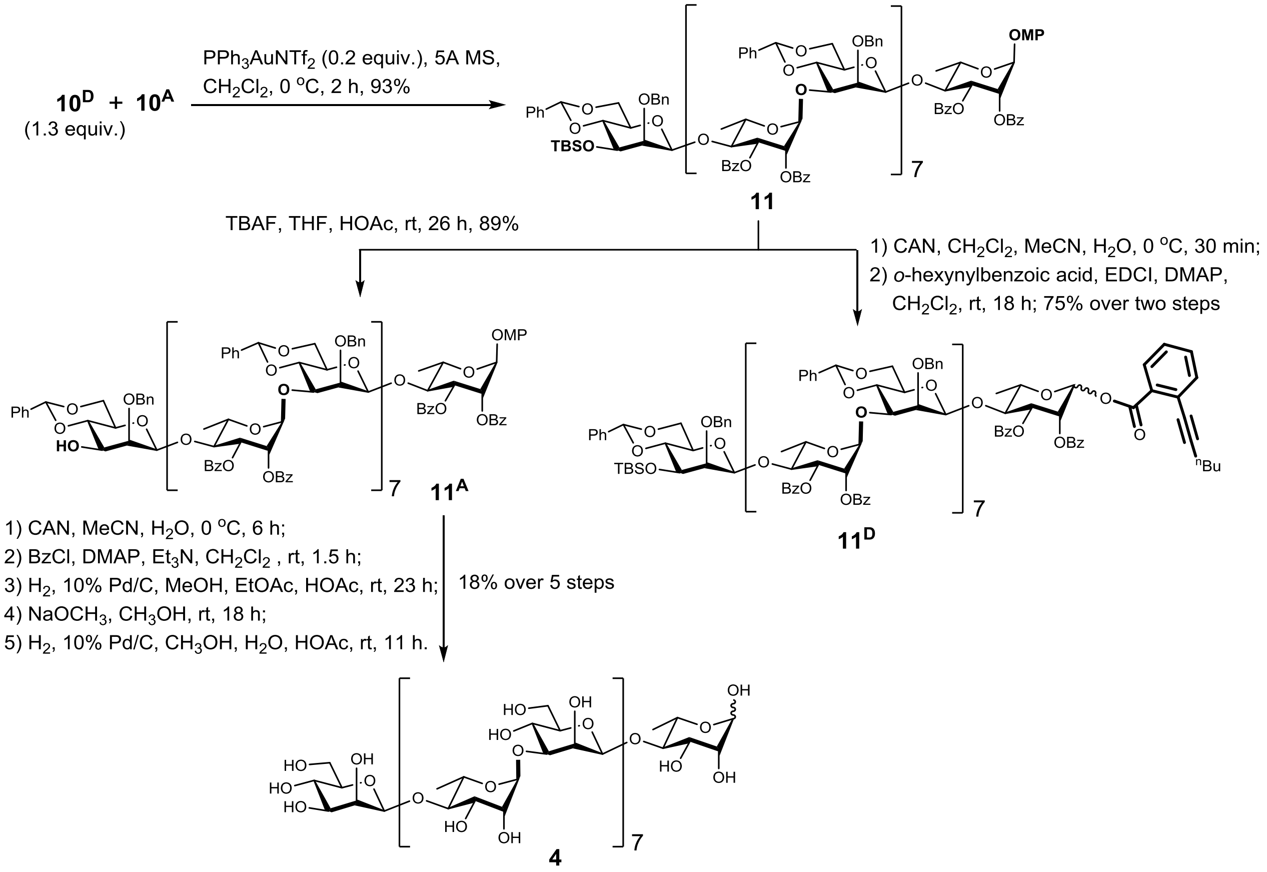

16-mer 11

A mixture of 8-mer donor **10^D** (2.10 g, 0.68 mmol) and acceptor **10^A** (1.53 g, 0.53 mmol) in a Schlenk flask equipped with a Teflon-coated magnetic stir bar was dried in high vacuum at 40 °C overnight. And then 5Å MS (2.60 g) and anhydrous CH_2Cl_2 (26 mL) were added. The mixture was stirred at rt for 15 min, and was then cooled to 0 °C, to which $Ph_3PAuNTf_2$ (78.0 mg, 0.11 mmol) was added. The resulting mixture was stirred at 0 °C for 2 h. After addition of Ph_3P (78 mg) and Et_3N (0.78 mL), the mixture was filtrated through a pad of Celite. The filtrate was concentrated *in vacuo* to give a residue, which was purified by silica gel column chromatography (petroleum ether/EtOAc/CH_2Cl_2, 3:1:1) to give 16-mer **11** (2.85 g, 93%) as a white foam. $R_f = 0.43$; $[\alpha]_D^{25} = 26.2$ (*c* 1.0, $CHCl_3$); ^1H NMR (500 MHz, $CDCl_3$) δ 8.06–7.95 (m, 16H), 7.87–7.71 (m, 18H), 7.67–7.57 (m, 9H), 7.54–7.46 (m, 16H), 7.45–7.36 (m, 18H), 7.36–7.25 (m, 11H), 7.21–7.15 (m, 64H), 7.12–7.01 (m, 11H), 6.89–6.83 (m, 2H), 5.77 (s, 1H), 5.68 (dd, *J* = 9.5, 4.0 Hz, 1H), 5.61–5.42

(m, 16H), 5.33 (d, *J* = 4.0 Hz, 1H), 5.24 (s, 10H), 5.20–5.17 (m, 1H), 4.76 (d, *J* = 4.6 Hz, 2H), 4.67–4.64 (m, 1H), 4.58–4.38 (m, 21H), 4.34–4.18 (m, 9H), 4.14–3.65 (m, 35H), 3.62 (s, 1H), 3.58–3.53 (m, 1H), 3.48–3.22 (m, 19H), 3.19–3.10 (m, 1H), 1.45 (d, *J* = 5.6 Hz, 3H), 1.14–0.98 (m, 21H), 0.64 (s, 9H), -0.32 (s, 3H), -0.39 (s, 3H); ^{13}C NMR (126 MHz, $CDCl_3$) δ 165.53, 165.15, 165.00, 164.90, 164.83, 164.82, 150.38, 138.61, 138.03, 137.69, 137.59, 137.31, 133.68, 133.61, 133.39, 132.23, 132.15, 132.07, 129.90, 129.80, 129.77, 129.53, 129.42, 129.34, 129.26, 129.15, 128.87, 128.75, 128.66, 128.37, 128.30, 128.17, 128.12, 128.00, 127.81, 127.68, 127.34, 126.21, 126.05, 126.00, 118.04, 114.80, 103.53, 103.25, 101.96, 101.80, 96.93, 94.97, 94.94, 94.80, 94.75, 79.48, 79.38, 79.36, 79.21, 78.73, 78.63, 76.38, 75.76, 75.72, 75.56, 75.55, 75.30, 75.15, 74.96, 74.45, 74.25, 72.72, 72.56, 72.55, 72.44, 72.43, 72.28, 71.28, 71.15, 71.03, 68.63, 68.58, 68.25, 68.18, 68.17, 67.86, 67.45, 67.42, 67.38, 67.35, 55.77, 53.55, 25.75, 18.20, 18.12, 17.54, 17.46, -4.82, -5.07; MALDI-TOF MS calcd for $C_{333}H_{326}O_{90}SiNa$ $[M + Na]^+$ 5818.0691, found 5818.0969.

16-mer donor 11^{D}

A solution of 16-mer **11** (1.78 g, 0.31 mmol) in CH_2Cl_2/MeCN (6 mL/12 mL) was stirred at 0 °C. Ammonium ceric nitrate (0.67 g, 1.23 mmol) was dissolved in H_2O (2 mL) at 0 °C. The latter solution was added into the former via syringe, and the mixture was stirred at 0 °C for 30 min. The mixture was diluted with CH_2Cl_2 and washed with sat. aq. $NaHCO_3$ and brine, respectively. The organic layer was dried over anhydrous Na_2SO_4 and concentrated *in vacuo*. The residue was purified by silica gel column chromatography (petroleum ether/EtOAc/CH_2Cl_2, 3:1:1) to give the corresponding hemiacetal (1.51 g, 87%) as a yellow foam.

To a mixture of the hemiacetal (1.51 g, 0.27 mmol), EDCI (0.13 g, 0.67 mmol), DMAP (81 mg, 0.67 mmol), and *ortho*-hexynylbenzoic acid (81 mg, 0.40 mmol), was added CH_2Cl_2 (10 mL). The mixture was stirred at rt for 18 h, and was then diluted with CH_2Cl_2 and washed with sat. aq. $NaHCO_3$. The organic layer was dried over anhydrous Na_2SO_4 and concentrated *in vacuo*. The residue was purified by silica gel column chromatography (petroleum ether/EtOAc/CH_2Cl_2, 7:2:3) to give **11^{D}** (1.18 g, 75% for two steps, α/β = 1:1) as a white foam. R_f = 0.80; ^{1}H NMR (600 MHz, $CDCl_3$) δ 8.12–7.96 (m, 18.6H), 7.90–7.73 (m, 18.5H), 7.72–7.58 (m, 10.5H), 7.57–7.48 (m, 20.5H), 7.47–7.38 (m,

22H), 7.38–7.33 (m, 5.5H), 7.33–7.26 (m, 13H), 7.26–7.14 (m, 73.5H), 7.14–7.03 (m, 11H), 6.47 (d, *J* = 1.9 Hz, 0.5H), 6.26 (s, 0.5H), 5.97 (d, *J* = 3.3 Hz, 0.5H), 5.82–5.78 (m, 0.5H), 5.65–5.51 (m, 10.5H), 5.51–5.45 (m, 6.5H), 5.38–5.31 (m, 1.5H), 5.29–5.18 (m, 6.5H), 4.80 (dd, *J* = 11.7, 5.0 Hz, 1H), 4.75 (dd, *J* = 11.7, 9.0 Hz, 1H), 4.66 (d, *J* = 8.2 Hz, 1H), 4.60–4.48 (m, 15.5H), 4.48–4.40 (m, 7.5H), 4.36–4.20 (m, 10.5H), 4.18–3.87 (m, 33H), 3.86–3.77 (m, 4.5H), 3.72 (m, 6.5H), 3.63 (dd, *J* = 6.6, 3.0 Hz, 1H), 3.60–3.55 (m, 1H), 3.51–3.21 (m, 24H), 3.17 (td, *J* = 9.8, 5.0 Hz, 1H), 2.52 (td, *J* = 7.1, 3.9 Hz, 1H), 2.46 (t, *J* = 7.1 Hz, 1H), 1.65–1.55 (m, 4.5H), 1.53–1.45 (m, 5.5H), 1.15–1.02 (m, 23.5H), 0.96 (t, *J* = 7.3 Hz, 2H), 0.92 (t, *J* = 7.3 Hz, 1.5H), 0.66 (s, 9H), -0.30 (s, 3H), -0.37 (s, 3H); ^{13}C NMR (151 MHz, $CDCl_3$) δ 165.66, 165.16, 165.07, 165.05, 164.97, 164.95, 164.92, 164.89, 164.84, 164.80, 164.72, 163.95, 163.17, 138.50, 137.96, 137.90, 137.61, 137.58, 137.49, 137.37, 137.20, 137.18, 134.75, 134.38, 133.64, 133.59, 133.51, 133.36, 133.32, 133.29, 132.27, 132.13, 130.51, 129.93, 129.87, 129.81, 129.73, 129.70, 129.67, 129.61, 129.52, 129.42, 129.35, 129.32, 129.29, 129.23, 129.16, 129.06, 128.98, 128.91, 128.79, 128.65, 128.63, 128.59, 128.55, 128.50, 128.39, 128.31, 128.27, 128.21, 128.19, 128.15, 128.07, 128.02, 127.89, 127.77, 127.70, 127.66, 127.61, 127.58, 127.29, 127.23, 126.93, 126.10, 125.97, 125.94, 125.89, 125.72, 125.69, 125.34, 125.02, 103.43, 103.39, 103.16, 102.95, 101.90, 101.86, 101.69, 97.19, 97.15, 94.90, 94.86, 94.68, 91.68, 90.93, 79.34, 79.21, 79.07, 79.04, 78.87, 78.66, 78.61, 78.57, 78.53, 77.24, 77.03, 76.82, 76.31, 76.26, 75.82, 75.73, 75.71, 75.65, 75.43, 75.37, 75.28, 75.17, 75.03, 74.88, 74.83, 74.31, 74.09, 74.01, 72.59, 72.40, 72.32, 72.25, 72.16, 71.18, 71.03, 70.13, 69.96, 68.53, 68.49, 68.11, 68.06, 67.75, 67.38, 67.27, 67.23, 30.68, 25.63, 22.06, 19.56, 19.52, 18.10, 18.08, 18.05, 17.43, 17.41, 17.34, 13.70, 13.66, -4.95, -5.19; MALDI-TOF MS calcd for $C_{339}H_{332}O_{90}SiK$ $[M + K]^+$ 5913.5, found 5912.5.

16-mer acceptor 11^A

To a solution of 16-mer **11** (1.07 g, 0.18 mmol) in anhydrous THF (6.20 mL), were added HOAc (21 μL, 0.36 mmol) and TBAF (1.0 M in THF, 0.92 mL, 0.92 mmol). The mixture was stirred at rt for 26 h, and was then diluted with CH_2Cl_2 and washed with sat. aq. NH_4Cl and brine, respectively. The organic layer was dried over anhydrous Na_2SO_4 and concentrated *in vacuo*. The residue was purified by silica gel column chromatography

(petroleum ether/EtOAc/CH_2Cl_2, 2.5:1:1) to give **11^{A}** (0.93 g, 89%) as a white foam. R_f = 0.40; $[\alpha]_D^{25}$ = 19.6 (*c* 1.0, $CHCl_3$); ^{1}H NMR (500 MHz, $CDCl_3$) δ 8.12–7.99 (m, 20H), 7.91–7.85 (m, 4H), 7.84–7.76 (m, 12H), 7.72–7.60 (m, 8H), 7.60–6.97 (m, 122H), 6.89 (d, *J* = 8.7 Hz, 2H), 5.82 (s, 1H), 5.75–5.69 (m, 1H), 5.68–5.45 (m, 16H), 5.40–5.19 (m, 7H), 4.80 (s, 2H), 4.73–4.41 (m, 19H), 4.39–4.20 (m, 10H), 4.20–3.86 (m, 26H), 3.85–3.56 (m, 14H), 3.53–3.15 (m, 27H), 1.49 (d, *J* = 5.5 Hz, 3H), 1.18–1.04 (m, 21H); ^{13}C NMR (126 MHz, $CDCl_3$) δ 165.15, 164.99, 164.98, 164.89, 155.36, 150.37, 138.02, 137.68, 137.38, 137.31, 137.23, 133.69, 133.64, 133.62, 133.60, 133.58, 133.56, 133.49, 133.45, 133.39, 129.90, 129.77, 129.53, 129.45, 129.33, 129.26, 129.16, 129.05, 128.75, 128.65, 128.54, 128.36, 128.30, 128.25, 127.98, 127.81, 127.69, 126.28, 126.05, 118.02, 114.80, 103.57, 103.54, 103.53, 103.51, 103.50, 102.76, 102.75, 102.02, 102.00, 101.97, 101.96, 101.94, 101.93, 101.91, 94.81, 94.79, 94.78, 79.25, 79.21, 79.18, 76.41, 76.38, 76.35, 75.70, 75.32, 75.29, 75.27, 74.95, 74.24, 74.21, 74.19, 72.45, 72.43, 72.42, 71.17, 71.15, 71.13, 71.08, 71.06, 71.03, 67.37, 67.35, 67.34, 67.32, 67.20, 67.17, 67.16, 55.76, 18.12, 17.59, 17.58, 17.55, 17.53; MALDI-TOF MS calcd for $C_{327}H_{312}O_{90}Na$ $[M + Na]^+$ 5702.9830, found 5703.9971.

16-mer 4

A solution of 16-mer **11^{A}** (43.0 mg, 7.59 μmol) in MeCN/CH_2Cl_2 (2/1 mL) was stirred at 0 °C. Ammonium ceric nitrate (100 mg, 0.182 mmol) was dissolved in H_2O (1.0 mL) at 0 °C. The latter solution was added into the former via syringe. The mixture was stirred at 0 °C for 2 mins, and was then diluted with CH_2Cl_2 and washed with sat. aq. $NaHCO_3$ and brine, respectively. The organic layer was dried over anhydrous Na_2SO_4 and concentrated *in vacuo*. The residue was used in the next step without further purification.

To a solution of the residue above, DMAP (20.0 mg, 0.16 mmol), Et_3N (14.6 μL, 0.11 mmol) in CH_2Cl_2 (2 mL), was added BzCl (6.06 μL, 52.6 μmol). The mixture was stirred at rt for 1.5 h, and was then diluted with CH_2Cl_2 and washed with sat. aq. $NaHCO_3$ and brine, respectively. The organic layer was dried over anhydrous Na_2SO_4 and concentrated *in vacuo*. The residue was purified by preparative TLC (petroleum ether/EtOAc/CH_2Cl_2, 2.5:1:1) to give a yellowish foam (23.3 mg).

The foam above (23.3 mg) was dissolved in a mixture of MeOH/EtOAc/HOAc (2

mL/4 mL/0.3 mL) containing 10% Pd/C (163 mg, wetted with 55% H_2O). The resulting mixture was stirred under H_2 atmosphere (1 atm) at rt for 23 h, and was then filtrated through a pad of Celite, and the Celite pad was washed with CH_2Cl_2/MeOH (1:1, v/v) three times. The filtrates were concentrated *in vacuo* to afford a glassy solid (7.9 mg).

The residue above (7.9 mg) was added into a $NaOCH_3$/CH_3OH solution (5 mL, pH = ~11). The mixture was stirred at rt for 18 h, and was then neutralized with Amberlyst 15 H^+ resin and filtered. The filtrate was concentrated *in vacuo*. The residue was purified by gel filtration (Sephadex G-25, H_2O) to afford crude 16-mer **4** (6.9 mg; a few benzyl groups were remained based on ^{1}H NMR analysis).

The crude **4** above (3.6 mg) was dissolved in a mixture of CH_3OH/H_2O/HOAc (1 mL/1 mL/0.2 mL) containing 10% Pd/C (4.8 mg, wetted with 55% H_2O). The resulting mixture was stirred under H_2 atmosphere (1 atm) at rt for 11 h, and was then filtered through a Millipore filter to give pure 16-mer **4** (3.5 mg, 18% over five steps; α/β = 1.7:1 based on ^{1}H NMR analysis) as a glass solid or white powder after lyophilization. ^{1}H NMR (600 MHz, D_2O) δ 5.12–5.11 (m, 0.58H), 4.99 (s, 7H), 4.90 (s, 8H), 4.87 (s, 0.35H), 4.33–4.27 (m, 7H), 4.09 (d, *J* = 3.2 Hz, 1H), 4.07–3.91 (m, 30H), 3.83–3.76 (m, 8H), 3.75–3.63 (m, 22H), 3.63–3.55 (m, 1.35H), 3.54–3.47 (m, 0.58H), 3.45–3.34 (m, 10H), 1.34 (d, *J* = 6.2 Hz, 24H); ^{13}C NMR (151 MHz, D_2O) δ 100.57, 100.48, 100.45, 100.42, 96.13, 93.79, 93.37, 79.78, 79.58, 79.40, 79.32, 76.84, 76.18, 76.08, 72.99, 72.83, 71.56, 71.02, 70.65, 70.54, 70.52, 70.23, 70.19, 70.00, 67.45, 67.39, 66.95, 66.75, 66.63, 66.59, 66.57, 64.93, 60.97, 60.92, 17.05, 17.02, 17.02, 16.82; HRMS (ESI) calcd for $C_{96}H_{162}O_{73}Na$ $[M + Na]^+$ 2506.8896, found 2506.8907.

Preparation of 32-mer 12, donor 12^D, acceptor 12^A, and 32-mer 5

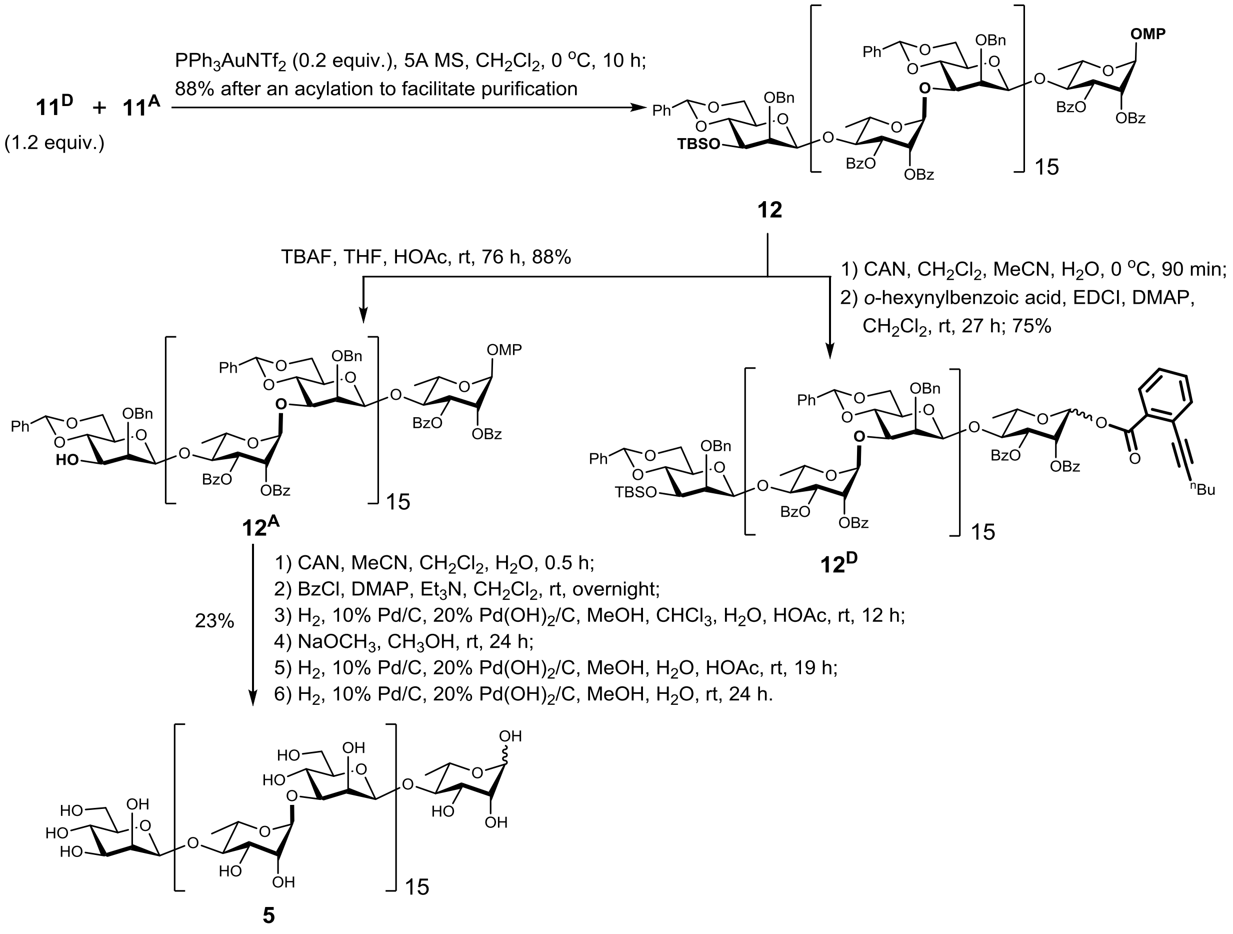

32-mer 12

A mixture of 16-mer donor $\mathbf{11^D}$ (1.08 g, 0.18 mmol) and acceptor $\mathbf{11^A}$ (0.85 g, 0.15 mmol) in a Schlenk flask equipped with a Teflon-coated magnetic stir bar was dried in high vacuum at 40 ^oC overnight. And then 5Å MS (4.0 g) and anhydrous CH_2Cl_2 (8.0 mL) were added. The mixture was stirred at rt for 15 min, and was then cooled to 0 ^oC, to which $Ph_3PAuNTf_2$ (22.2 mg, 30.1 μmol) was added. The resulting mixture was stirred at 0 ^oC for 10 h. After addition of Ph_3P (22 mg) and Et_3N (22 μL), the mixture was filtrated through a pad of Celite. The filtrate was concentrated *in vacuo* to give a residue, which was purified by silica gel column chromatography (petroleum ether/EtOAc/CH_2Cl_2, 3:1:1 then 1:1:1) to provide crude 32-mer **12** (1.72 g, ~92%) as a white solid.

To facilitate the purification of **12**, an acylation reaction was performed to convert the mixed compounds bearing hydroxyl groups, such as the remaining acceptor and hydrolyzed donor, into less polar products.

Thus, to a mixture of the crude **12** above, EDCI (0.74 g, 3.88 mmol), DMAP (1.59 g, 13.0 mmol), and *ortho*-hexynylbenzoic acid (1.31 g, 6.48 mmol), was added anhydrous CH_2Cl_2 (15 mL). The mixture was stirred at rt for 1.5 h, and was then diluted with CH_2Cl_2 and washed with sat. aq. $NaHCO_3$. The organic layer was dried over anhydrous Na_2SO_4 and concentrated *in vacuo*. The residue was purified by silica gel column chromatography (EtOAc/petroleum ether/CH_2Cl_2, 1:2.5:1) to give pure compound **12** (1.65 g, 88%) as a white solid. R_f = 0.38; $[\alpha]_D^{25}$ = 33.3 (*c* 1.0, $CHCl_3$); ^{1}H NMR (600 MHz, $CDCl_3$) δ 8.10–7.97 (m, 38H), 7.92–7.72 (m, 35H), 7.71–7.61 (m, 19H), 7.59–7.48 (m, 39H), 7.48–7.27 (m, 61H), 7.25–7.15 (m, 151H), 7.14–7.02 (m, 21H), 6.90–6.87 (m, 2H), 5.80 (dd, *J* = 3.4, 1.9 Hz, 1H), 5.69 (dd, *J* = 9.6, 3.4 Hz, 1H), 5.67–5.44 (m, 34H), 5.34 (dd, *J* = 3.4, 1.6 Hz, 1H), 5.29–5.23 (m, 14H), 5.20 (dd, *J* = 3.5, 1.6 Hz, 1H), 5.15–5.11 (m, 1H), 4.93–4.85 (m, 1H), 4.78 (s, 2H), 4.70–4.64 (m, 2H), 4.63–4.39 (m, 51H), 4.38–4.21 (m, 19H), 4.20–3.66 (m, 88H), 3.64 (d, *J* = 3.1 Hz, 1H), 3.60–3.52 (m, 2H), 3.51–3.13 (m, 53H), 1.47 (d, *J* = 6.1 Hz, 3H), 1.14–1.03 (m, 45H), -0.29 (s, 3H), -0.36 (s, 3H); ^{13}C NMR (151 MHz, $CDCl_3$) δ 165.58, 165.19, 165.16, 165.07, 165.04, 164.98, 164.94, 164.87, 155.40, 150.43, 138.65, 138.06, 137.75, 137.72, 137.63, 137.34, 133.72, 133.65, 133.44, 129.95, 129.85, 129.82, 129.66, 129.61, 129.56, 129.51, 129.47, 129.38, 129.30, 129.21, 128.80, 128.77, 128.70, 128.42, 128.36, 128.34, 128.22, 128.19, 128.17, 128.04, 127.85, 127.76, 127.73, 127.37, 126.30, 126.24, 126.11, 126.09, 126.04, 118.07, 114.84, 103.57, 103.30, 103.28, 102.05, 102.01, 101.84, 96.95, 95.00, 94.82, 79.49, 79.38, 79.21, 79.17, 78.75, 78.68, 77.37, 77.16, 76.95, 76.45, 76.41, 75.79, 75.74, 75.57, 75.31, 75.17, 75.02, 74.97, 74.45, 74.23, 72.74, 72.59, 72.46, 72.30, 71.31, 71.18, 71.07, 68.66, 68.23, 68.20, 67.89, 67.49, 67.42, 67.37, 55.81, 25.77, 18.23, 18.15, 17.56, 17.56, 17.49, 0.14, -4.80, -5.05; MALDI-TOF MS calcd for $C_{653}H_{630}O_{178}SiNa$ $[M + Na]^+$ 11377.1198, found 11377.3294.

32-mer donor 12^D

A solution of 32-mer **12** (320 mg, 28.2 μmol) in CH_2Cl_2/MeCN (5 mL/6.5 mL) was stirred at 0 °C. Ammonium ceric nitrate (262 mg, 0.48 mmol) was dissolved in H_2O (1 mL) at 0 °C. The latter solution was added into the former via syringe, and the mixture was stirred at 0 °C for 90 min. The mixture was diluted with CH_2Cl_2 and washed with sat. aq. $NaHCO_3$ and brine, respectively. The organic layer was dried over anhydrous Na_2SO_4 and

concentrated *in vacuo*. The residue was purified by silica gel column chromatography (petroleum ether/EtOAc/CH_2Cl_2, 2:1:1) to give the corresponding hemiacetal (237 mg, 83%) as a yellowish solid.

To a mixture of the hemiacetal (237 mg, 21.1 μmol), EDCI (10.1 mg, 52.8 μmol), DMAP (6.4 mg, 52.8 μmol), and *ortho*-hexynylbenzoic acid (6.41 mg, 31.67 μmol), was added CH_2Cl_2 (10 mL). The mixture was stirred at rt for 27 h, and was then diluted with CH_2Cl_2 and washed with sat. aq. $NaHCO_3$. The organic layer was dried over anhydrous Na_2SO_4 and concentrated *in vacuo*. The residue was purified by silica gel column chromatography (petroleum ether/EtOAc/CH_2Cl_2, 2.5:1:1) to give compound **12$^{\mathbf{D}}$** (216 mg, 75 % for two steps; α/β = 1.5:1) as a white foam. R_f = 0.55; ^{1}H NMR (600 MHz, $CDCl_3$) δ 8.12–7.94 (m, 32H), 7.90–7.68 (m, 32H), 7.68–7.57 (m, 17H), 7.56–7.45 (m, 35H), 7.45–7.37 (m, 34H), 7.36–7.25 (m, 26H), 7.25–7.15 (m, 129H), 7.15–6.99 (m, 25H), 6.47 (d, *J* = 2.0 Hz, 0.60H), 6.25 (s, 0.40H), 5.97 (d, *J* = 3.5 Hz, 0.40H), 5.80 (dd, *J* = 3.5, 2.0 Hz, 0.60H), 5.67–5.39 (m, 30H), 5.37–5.29 (m, 2H), 5.28–5.23 (m, 12H), 5.20 (dd, *J* = 3.4, 1.6 Hz, 1H), 4.92–4.84 (m, 1H), 4.80 (dd, *J* = 11.6, 4.9 Hz, 1H), 4.75 (dd, *J* = 11.7, 8.8 Hz, 1H), 4.65 (d, *J* = 8.8 Hz, 1H), 4.62–4.38 (m, 41H), 4.37–4.20 (m, 14H), 4.18–3.96 (m, 41H), 3.96–3.66 (m, 34H), 3.63 (dd, *J* = 6.6, 3.0 Hz, 1H), 3.60–3.51 (m, 2H), 3.50–3.14 (m, 43H), 2.63–2.39 (m, 2H), 1.64–1.53 (m, 3H), 1.53–1.44 (m, 4H), 1.19–1.00 (m, 45H), 0.99–0.90 (m, 3H), 0.66 (s, 9H), -0.30 (s, 3H), -0.37 (s, 3H); ^{13}C NMR (151 MHz, $CDCl_3$) δ 165.80, 165.67, 165.47, 165.30, 165.21, 165.11, 165.09, 165.06, 165.03, 164.97, 164.93, 164.86, 164.09, 163.30, 141.64, 141.01, 140.55, 139.80, 138.63, 138.29, 138.10, 138.04, 137.92, 137.91, 137.88, 137.74, 137.71, 137.62, 137.54, 137.50, 137.33, 137.13, 135.98, 135.21, 134.89, 134.52, 133.65, 133.46, 133.42, 132.41, 132.27, 130.75, 130.64, 130.07, 130.00, 129.84, 129.81, 129.74, 129.65, 129.55, 129.48, 129.45, 129.42, 129.37, 129.29, 129.21, 129.18, 129.15, 129.11, 129.07, 128.93, 128.84, 128.79, 128.76, 128.72, 128.69, 128.64, 128.56, 128.45, 128.41, 128.35, 128.33, 128.25, 128.21, 128.18, 128.16, 128.13, 128.11, 128.03, 127.91, 127.83, 127.80, 127.75, 127.72, 127.60, 127.43, 127.37, 127.15, 127.07, 126.62, 126.32, 126.29, 126.23, 126.17, 126.07, 126.03, 126.00, 125.83, 125.47, 103.56, 103.29, 103.08, 102.04, 102.00, 101.83, 97.33, 97.29, 94.99, 94.81, 91.81, 91.07, 79.48, 79.35, 79.20, 79.00, 78.79, 78.74, 78.69, 78.67, 76.44, 76.40, 75.95, 75.85, 75.78, 75.69, 75.56, 75.51, 75.30, 75.16, 75.09, 75.01, 74.96, 74.44, 74.22, 74.15, 73.27, 72.73,

72.54, 72.45, 72.38, 72.30, 71.32, 71.17, 70.91, 70.27, 70.10, 68.65, 68.19, 67.88, 67.52, 67.41, 67.36, 30.80, 25.76, 22.20, 19.57, 17.55, 13.79, -4.81, -5.05; MALDI-TOF MS calcd for $C_{659}H_{636}O_{178}SiNa$ $[M + Na]^+$ 11455.2338, found 11457.0144.

32-mer acceptor $\mathbf{12^A}$

To a solution of 32-mer **12** (0.65 g, 0.057 mmol) in anhydrous THF (4 mL), were added HOAc (6.51 µL, 0.11 mmol) and TBAF (1.0 M in THF, 0.29 mL, 0.29 mmol). The mixture was stirred at rt for 76 h, and was then diluted with CH_2Cl_2 and washed with sat. aq. NH_4Cl and brine, respectively. The organic layer was dried over anhydrous Na_2SO_4 and concentrated *in vacuo*. The residue was purified by silica gel column chromatography (petroleum ether/EtOAc/CH_2Cl_2, 2:1:1) to give $\mathbf{12^A}$ (0.57 g, 88%) as a white solid. R_f = 0.40; $[\alpha]_D^{20}$ = 31.1 (*c* 1.0, $CHCl_3$); ^1H NMR (600 MHz, $CDCl_3$) δ 8.16–7.95 (m, 32H), 7.92–7.71 (m, 33H), 7.70–7.60 (m, 17H), 7.59–7.47 (m, 35H), 7.46–7.35 (m, 38H), 7.34–6.91 (m, 189H), 6.91–6.86 (m, 2H), 5.82–5.77 (m, 1H), 5.69 (dd, *J* = 9.6, 3.5 Hz, 1H), 5.66–5.44 (m, 32H), 5.34 (d, *J* = 3.8 Hz, 1H), 5.30–5.22 (m, 12H), 5.20 (d, *J* = 3.6 Hz, 1H), 4.88 (d, *J* = 9.4 Hz, 1H), 4.80–4.73 (br s, 2H), 4.71–4.37 (m, 46H), 4.36–4.18 (m, 19H), 4.17–3.97 (m, 46H), 3.97–3.83 (m, 17H), 3.83–3.61 (m, 22H), 3.57 (dd, *J* = 10.0, 3.1 Hz, 1H), 3.51–3.34 (m, 29H), 3.34–3.13 (m, 16H), 1.46 (d, *J* = 6.2 Hz, 3H), 1.15–1.01 (m, 45H); ^{13}C NMR (151 MHz, $CDCl_3$) δ 165.53, 165.14, 165.12, 165.06, 164.99, 164.94, 164.90, 155.35, 150.38, 138.02, 137.67, 137.51, 137.38, 137.30, 137.20, 133.67, 133.61, 133.59, 133.39, 129.90, 129.81, 129.77, 129.56, 129.52, 129.48, 129.44, 129.37, 129.33, 129.26, 129.16, 129.04, 128.75, 128.73, 128.71, 128.65, 128.55, 128.37, 128.31, 128.29, 128.25, 128.18, 128.15, 128.12, 128.08, 127.99, 127.80, 127.71, 127.68, 126.27, 126.19, 126.06, 126.04, 125.98, 118.02, 114.79, 103.52, 103.24, 102.76, 102.00, 101.96, 96.91, 94.94, 94.77, 79.34, 79.30, 79.17, 78.14, 78.01, 77.33, 77.12, 76.91, 76.40, 76.36, 75.74, 75.70, 75.26, 75.05, 74.93, 74.18, 72.54, 72.42, 72.13, 71.26, 71.13, 71.06, 71.02, 70.35, 68.61, 68.55, 68.18, 68.15, 67.45, 67.32, 67.16, 55.77, 18.13, 17.57, 17.54; MALDI-TOF MS calcd for $C_{647}H_{616}O_{178}Na$ $[M + Na]^+$ 11262.8568, found 11261.9229.

32-mer 5

A solution of 32-mer $\mathbf{12^A}$ (58.5 mg, 5.20 µmol) in MeCN/CH_2Cl_2 (3 mL/3.5 mL) was

stirred at 0 °C. Ammonium ceric nitrate (111 mg, 0.20 mmol) was dissolved in H_2O (0.5 mL) at 0 °C. The latter solution was added into the former via syringe. The mixture was stirred at 0 °C for 0.5 h, and was then diluted with CH_2Cl_2 and washed with sat. aq. $NaHCO_3$ and brine, respectively. The organic layer was dried over anhydrous Na_2SO_4 and concentrated *in vacuo*. The residue was used in the next step without further purification.

To a solution of the residue above, DMAP (20 mg, 0.16 mmol), and Et_3N (14.6 μL, 0.105 mmol) in CH_2Cl_2 (2 mL), was added BzCl (6.06 μL, 52.6 μmol). The mixture was stirred at rt overnight, and was then diluted with CH_2Cl_2 and washed with sat. aq. $NaHCO_3$ and brine, respectively. The organic layer was dried over anhydrous Na_2SO_4 and concentrated *in vacuo*. The residue was purified by silica gel column chromatography (petroleum ether/EtOAc/CH_2Cl_2, 2:1:1) to give a white solid (31 mg).

The solid above (30 mg) was dissolved in a mixture of MeOH/$CHCl_3$/H_2O/HOAc (1 mL/2 mL/0.1 mL/0.1 mL) containing 10% Pd/C (30 mg, wetted with 55% H_2O) and 20% $Pd(OH)_2$/C (30 mg, wetted with 50% H_2O)[5]. The resulting mixture was stirred under H_2 atmosphere (1 atm) at rt for 12 h, and was then filtrated through a pad of Celite, and the Celite pad was washed with CH_2Cl_2/MeOH (1:1, v/v) three times. The filtrates were concentrated to afford a white solid (30 mg).

The residue above (30 mg) was added into a $NaOCH_3$/CH_3OH solution (5 mL, pH = ~11). The mixture was stirred at rt for 24 h, and was then neutralized with Amberlyst 15 H^+ resin and filtered. The filtrate was concentrated to give a residue; ^1H NMR analysis of the residue indicated remaining of a few benzyl groups.

The solid above (30 mg) was dissolved in a mixture of MeOH/H_2O/HOAc (3.5 mL/0.2 mL/0.2 mL) containing 10% Pd/C (36 mg, wetted with 55% H_2O) and 20% $Pd(OH)_2$/C (40 mg, wetted with 50% H_2O). The resulting mixture was stirred under H_2 atmosphere (1 atm) at rt for 19 h, and was then filtrated through a pad of Celite, and the Celite pad was washed with CH_2Cl_2/MeOH (1:1, v/v) three times. The filtrates were concentrated to afford a glassy solid (6 mg); ^1H NMR analysis of this solid indicated remaining of a few benzyl groups.

The solid above (6 mg) was dissolved in a mixture of MeOH/H_2O (1 mL/1 mL) containing 10% Pd/C (4.0 mg, wetted with 55% H_2O) and 20% $Pd(OH)_2$/C (4.0 mg, wetted with 50% H_2O). The resulting mixture was stirred under H_2 atmosphere (1 atm) at rt for 24 h, and was then filtrated through a pad of Celite, and the Celite pad was washed with

H_2O/MeOH (1:1, v/v) three times. The filtrates were concentrated. The residue was purified by gel filtration (Sephadex G-25, H_2O) to afford 32-mer **5** (6 mg, 23% over six steps; α/β = 1.6:1 based on ^{1}H NMR analysis) as a glassy solid or white powder after lyophilization. ^{1}H NMR (600 MHz, D_2O) δ 5.08–5.05 (m, 0.61H), 4.94 (s, 15H), 4.86 (s, 16H), 4.82 (s, 5H), 4.27–4.22 (m, 15H), 4.06–3.85 (m, 81H), 3.83–3.48 (m, 74H), 3.36 (s, 19H), 1.29 (d, J = 6.1 Hz, 48H); ^{13}C NMR (151 MHz, D_2O) δ 100.59, 100.50, 96.16, 79.62, 79.44, 76.88, 76.20, 76.10, 73.02, 70.55, 70.21, 70.02, 67.47, 67.42, 66.78, 66.60, 64.96, 60.95, 17.08, 16.85; MALDI-TOF MS calcd for $C_{192}H_{322}O_{145}Na$ $[M + Na]^+$ 4973.5328, found 4974.0401.

Preparation of 64-mer 13, donor 13^D, acceptor 13^A, and 64-mer 6

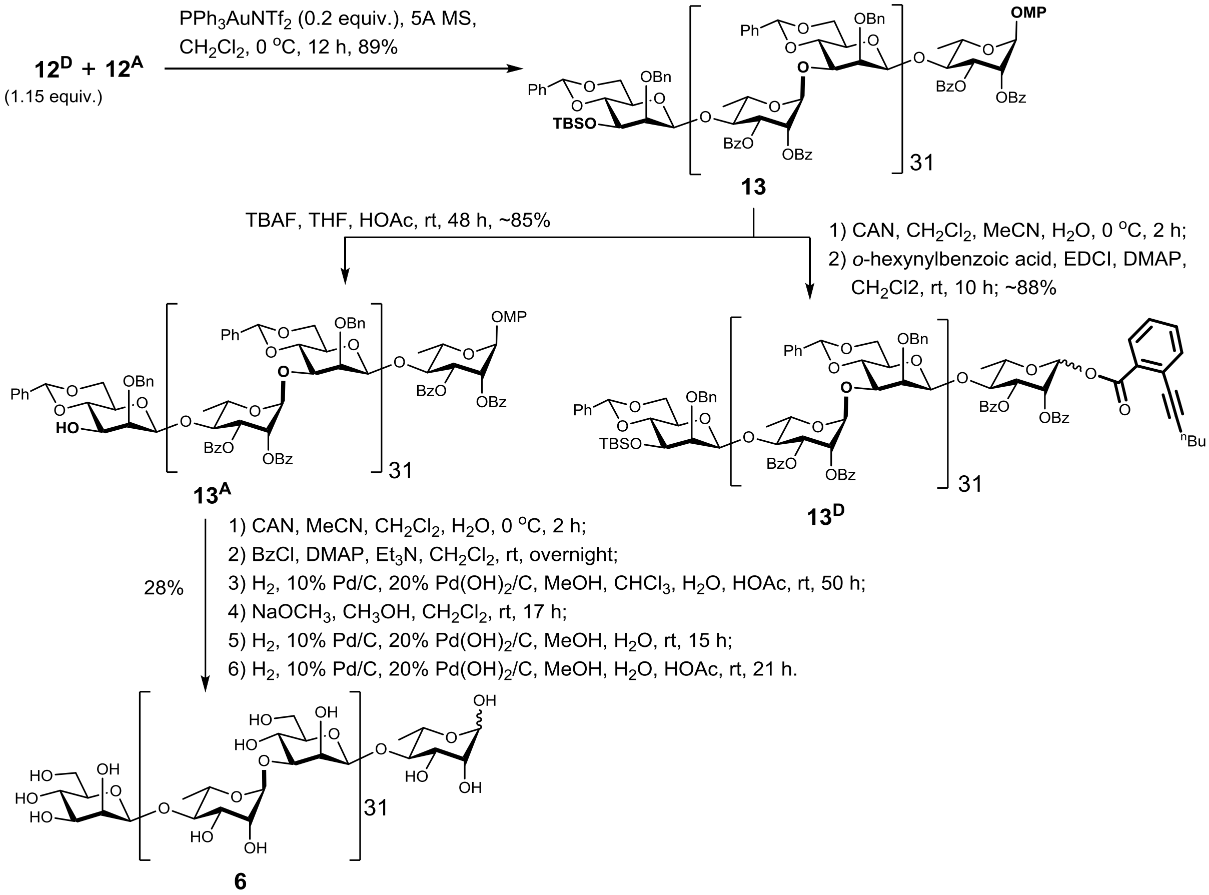

64-mer 13

A mixture of 32-mer donor **12^D** (343 mg, 30.0 μmol) and acceptor **12^A** (293 mg, 26.1 μmol) in a Schlenk flask equipped with a Teflon-coated magnetic stir bar was dried in high vacuum at 40 °C overnight. And then 5Å MS (2.1 g) and anhydrous CH_2Cl_2 (3.5 mL) were

added. The mixture was stirred at rt for 15 min, and was then cooled to 0 °C, to which $Ph_3PAuNTf_2$ (3.85 mg, 5.22 μmol) was added. The resulting mixture was stirred at 0 °C for 12 h. After addition of Ph_3P (4 mg) and Et_3N (4 μL), the mixture was filtrated through a pad of Celite. After removal of solvent, the resulting residue was dissolved in $CHCl_3$ and filtrated through Millipore filter, and then GPC ($CHCl_3$) was performed to give 64-mer **13** (556 mg, 89%) as a white solid. R_f = 0.38; $[\alpha]_D^{25}$ = 34.1 (*c* 1.0, $CHCl_3$); ^{1}H NMR (600 MHz, $CDCl_3$) δ 8.17–7.95 (m, 65H), 7.94–7.71 (m, 63H), 7.70–7.61 (m, 34H), 7.59–7.46 (m, 68H), 7.46–7.37 (m, 64H), 7.36–7.27 (m, 33H), 7.27–7.10 (m, 276H), 7.08–7.01 (m, 33H), 6.90–6.85 (m, 2H), 5.81–5.77 (m, 1H), 5.69 (dd, *J* = 9.6, 3.4 Hz, 1H), 5.66–5.37 (m, 64H), 5.37–5.17 (m, 31H), 4.78 (br s, 2H), 4.73–4.36 (m, 90H), 4.36–4.19 (m, 32H), 4.18–3.66 (m, 159H), 3.63 (d, *J* = 3.0 Hz, 1H), 3.61–3.35 (m, 64H), 3.34–3.12 (m, 32H), 1.46 (d, *J* = 6.1 Hz, 3H), 1.27–0.91 (m, 93H), 0.66 (s, 9H), -0.30 (s, 3H), -0.37 (s, 3H); ^{13}C NMR (151 MHz, $CDCl_3$) δ 165.46, 165.08, 165.05, 164.95, 164.92, 164.87, 164.83, 164.76, 155.29, 150.32, 138.53, 137.95, 137.64, 137.61, 137.52, 137.39, 137.31, 137.23, 133.61, 133.54, 133.36, 133.32, 129.84, 129.71, 129.55, 129.45, 129.40, 129.35, 129.30, 129.27, 129.19, 129.11, 128.97, 128.82, 128.68, 128.58, 128.43, 128.30, 128.22, 128.10, 128.06, 127.93, 127.74, 127.62, 127.26, 126.13, 125.97, 125.93, 117.96, 114.72, 103.46, 103.19, 103.17, 101.94, 101.90, 101.76, 101.72, 96.84, 94.89, 94.88, 94.71, 79.38, 79.27, 79.10, 78.63, 78.56, 76.30, 75.67, 75.63, 75.46, 75.20, 75.06, 74.86, 74.11, 72.63, 72.47, 72.35, 72.19, 71.19, 71.06, 70.95, 68.55, 68.09, 67.78, 67.38, 67.26, 55.70, 25.66, 25.63, 18.11, 18.04, 17.58, 17.44, 17.37, -4.91, -5.16; MALDI-TOF MS calcd for $C_{1293}H_{1240}O_{354}SiNa$ $[M + Na]^+$ 22494.8638, found 22483.7825.

64-mer donor 13^D

A solution of 64-mer **13** (320 mg, 14.2 μmol) in CH_2Cl_2/MeCN (10 mL/4 mL) was stirred at 0 °C. Ammonium ceric nitrate (281 mg, 0.51 mmol) was dissolved in H_2O (1 mL) at 0 °C. The latter solution was added into the former via syringe, and the mixture was stirred at 0 °C for 2 h. The mixture was diluted with CH_2Cl_2 and washed with sat. aq. $NaHCO_3$ and brine, respectively. The organic layer was dried over anhydrous Na_2SO_4 and concentrated *in vacuo*. The residue was purified by silica gel column chromatography

(petroleum ether/EtOAc/CH_2Cl_2, 4:1.5:3) to give the corresponding crude hemiacetal (301 mg) as a yellowish solid.

To a mixture of the crude hemiacetal (301 mg, ~13.5 μmol), EDCI (32.1 mg, 0.17 mmol), DMAP (20.6 mg, 0.170 mmol), and *ortho*-hexynylbenzoic acid (20.4 mg, 0.10 mmol), was added CH_2Cl_2 (10 mL). The mixture was stirred at rt for 10 h, and was then diluted with CH_2Cl_2 and washed with sat. aq. $NaHCO_3$. The organic layer was dried over anhydrous Na_2SO_4 and concentrated *in vacuo*. The residue was purified by silica gel column chromatography (petroleum ether/EtOAc/CH_2Cl_2, 12:1.5:3 then 4:1.5:3) to give compound **13^D** (283 mg, ~88% for two steps, α/β = 2.3:1) as a white solid. R_f = 0.70; ^1H NMR (600 MHz, $CDCl_3$) δ 8.11–7.94 (m, 66H), 7.87–7.70 (m, 67H), 7.68–7.58 (m, 34H), 7.57–7.47 (m, 69H), 7.46–7.38 (m, 66H), 7.37–7.10 (m, 349H), 7.09–7.01 (m, 35H), 6.48–6.45 (m, 0.68H), 6.27–6.24 (m, 0.25H), 5.98–5.95 (m, 0.25H), 5.81–5.77 (m, 0.68H), 5.68–5.39 (m, 64H), 5.37–5.17 (m, 31H), 5.15–5.09 (m, 1H), 4.90–4.84 (m, 1H), 4.82–4.71 (m, 3H), 4.70–4.46 (m, 63H), 4.46–4.36 (m, 33H), 4.36–4.20 (m, 35H), 4.19–4.02 (m, 62H), 4.02–3.96 (m, 31H), 3.96–3.80 (m, 38H), 3.79–3.61 (m, 32H), 3.61–3.34 (m, 64H), 3.34–3.14 (m, 34H), 2.56–2.42 (m, 2H), 1.65–1.44 (m, 7H), 1.21–0.88 (m, 96H), 0.66 (s, 9H), -0.30 (s, 3H), -0.37 (s, 3H); ^{13}C NMR (151 MHz, $CDCl_3$) δ 165.81, 165.31, 165.21, 165.12, 165.03, 164.94, 164.87, 164.09, 138.64, 138.11, 138.04, 137.75, 137.71, 137.63, 137.55, 137.34, 134.90, 133.65, 133.43, 132.41, 130.65, 130.08, 130.01, 129.84, 129.81, 129.66, 129.56, 129.48, 129.46, 129.44, 129.38, 129.30, 129.19, 129.08, 128.79, 128.77, 128.69, 128.65, 128.55, 128.41, 128.33, 128.22, 128.19, 128.17, 128.13, 128.04, 127.91, 127.81, 127.76, 127.72, 127.44, 127.37, 127.15, 127.08, 126.30, 126.24, 126.09, 126.08, 126.04, 125.93, 125.87, 125.66, 125.49, 103.56, 103.30, 103.08, 102.00, 101.84, 97.34, 95.00, 94.82, 91.82, 91.08, 79.49, 79.20, 78.74, 78.67, 77.37, 77.16, 76.95, 76.41, 75.96, 75.79, 75.57, 75.31, 75.17, 75.02, 74.97, 74.45, 74.22, 73.27, 72.74, 72.46, 72.30, 71.17, 70.27, 70.10, 68.66, 68.20, 67.89, 67.37, 30.81, 30.46, 25.77, 22.23, 22.21, 19.70, 18.24, 18.22, 17.55, 17.48, 13.84, 13.80, -4.81, -5.05; MALDI-TOF MS calcd for $C_{1293}H_{1240}O_{354}SiNa$ $[M + Na]^+$ 22570.9618, found 22550.4537.

64-mer acceptor 13^A

To a solution of 64-mer **13** (190 mg, 8.5 μmol) in anhydrous THF (5 mL), were added HOAc (2 μL, 34.9 μmol) and TBAF (1.0 M in THF, 86.0 μL, 86.0 μmol). The mixture was stirred at rt for 48 h, and was then diluted with CH_2Cl_2 and washed with sat. aq. NH_4Cl and brine, respectively. The organic layer was dried over anhydrous Na_2SO_4 and concentrated *in vacuo*. The residue was purified by silica gel column chromatography (petroleum ether/EtOAc/CH_2Cl_2, 4:1.5:3) to give **13^A** (179 mg, ~85%; >90% purity based on ^{1}H NMR analysis) as a white solid. R_f = 0.55; $[\alpha]_D^{20}$ = 36.7 (*c* 1.0, $CHCl_3$); ^{1}H NMR (600 MHz, $CDCl_3$) δ 8.09–7.92 (m, 67H), 7.90–7.70 (m, 68H), 7.68–7.62 (m, 37H), 7.56–7.49 (m, 68H), 7.44–7.35 (m, 76H), 7.32–7.27 (m, 39H), 7.24–7.11 (m, 289H), 7.10–7.02 (m, 34H), 6.90–6.85 (m, 2H), 5.81–5.77 (m, 1H), 5.69 (dd, *J* = 9.6, 3.4 Hz, 1H), 5.65–5.55 (m, 32H), 5.54–5.41 (m, 31H), 5.35–5.32 (m, 1H), 5.29–5.18 (m, 32H), 4.67 (s, 1H), 4.63 (s, 1H), 4.61 (s, 1H), 4.58–4.47 (m, 62H), 4.47–4.38 (m, 30H), 4.29–4.24 (m, 34H), 4.23–4.20 (m, 1H), 4.09–4.02 (m, 68H), 4.01–3.98 (m, 32H), 3.95–3.83 (m, 58H), 3.79–3.61 (m, 37H), 3.59–3.55 (m, 3H), 3.48–3.37 (m, 71H), 3.30–3.22 (m, 37H), 1.46 (d, *J* = 6.1 Hz, 3H), 1.21–0.92 (m, 96H); ^{13}C NMR (151 MHz, $CDCl_3$) δ 165.58, 165.19, 165.17, 165.04, 164.98, 164.94, 155.40, 150.43, 138.06, 137.72, 137.51, 137.42, 137.34, 137.25, 133.66, 133.44, 129.95, 129.85, 129.82, 129.61, 129.56, 129.49, 129.46, 129.42, 129.38, 129.31, 129.21, 129.08, 128.80, 128.77, 128.70, 128.60, 128.42, 128.34, 128.30, 128.22, 128.19, 128.17, 128.12, 128.03, 127.85, 127.73, 127.15, 126.32, 126.30, 126.24, 126.11, 126.09, 126.02, 125.87, 118.07, 114.84, 103.57, 103.28, 102.80, 102.05, 102.01, 96.96, 94.99, 94.82, 79.38, 79.35, 79.21, 78.19, 78.06, 76.66, 76.45, 76.41, 75.79, 75.74, 75.31, 75.18, 74.97, 74.22, 72.59, 72.46, 72.18, 71.17, 71.11, 71.07, 70.40, 68.66, 68.23, 68.20, 67.37, 67.21, 55.82, 18.15, 17.59, 17.56; MALDI-TOF MS calcd for $C_{1287}H_{1224}O_{354}Na$ $[M + Na]^+$ 22378.5848, found 22375.2123.

64-mer 6

A solution of 64-mer **13^A** (27.2 mg, 1.21 μmol) in MeCN/CH_2Cl_2 (4.5 mL/4.0 mL) was stirred at 0 °C. Ammonium ceric nitrate (143 mg, 0.26 mmol) was dissolved in H_2O (0.5 mL) at 0 °C. The latter solution was added into the former via syringe. The mixture was stirred at 0 °C for 2 h, and was then diluted with CH_2Cl_2 and washed with sat. aq.

$NaHCO_3$ and brine, respectively. The organic layer was dried over anhydrous Na_2SO_4 and concentrated *in vacuo*. The residue was used in the next step without further purification.

To a solution of the residue above (27 mg), DMAP (20 mg, 0.16 mmol), and Et_3N (14.6 μL, 0.11 mmol) in CH_2Cl_2 (2 mL), was added BzCl (6.06 μL, 52.6 μmol). The mixture was stirred at rt overnight, and was then diluted with CH_2Cl_2 and washed with sat. aq. $NaHCO_3$ and brine, respectively. The organic layer was dried over anhydrous Na_2SO_4 and concentrated *in vacuo*. The residue was purified by silica gel column chromatography (petroleum ether/EtOAc/CH_2Cl_2, 1:1:1) to give a white solid (31 mg).

The solid above (31 mg) was dissolved in a mixture of MeOH/$CHCl_3$/H_2O/HOAc (1 mL/2 mL/0.1 mL/0.1 mL) containing 10% Pd/C (60 mg, wetted with 55% H_2O) and 20% $Pd(OH)_2$/C (60 mg, wetted with 50% H_2O). The resulting mixture was stirred under H_2 atmosphere (1 atm) at rt for 50 h, and was then filtrated through a pad of Celite, and the Celite pad was washed with CH_2Cl_2, MeOH, and H_2O, respectively. The filtrates were concentrated to afford a white solid (26 mg).

The residue above (26 mg) was added into a solution of $NaOCH_3$/CH_3OH (5 mL, pH = ~11) and CH_2Cl_2 (4 mL). The mixture was stirred at rt for 17 h, and was then neutralized with Amberlyst 15 H^+ resin and filtered. The filtrate was concentrated to give a residue (4.3 mg). ^1H NMR analysis of the residue indicated remaining of a few benzyl groups.

Thus, the solid above (4.3 mg) was dissolved in a mixture of MeOH/H_2O (1.5 mL/1.5 mL) containing 10% Pd/C (4 mg, wetted with 55% H_2O) and 20% $Pd(OH)_2$/C (4 mg, wetted with 50% H_2O). The resulting mixture was stirred under H_2 atmosphere (1 atm) at rt for 15 h, and was then filtrated through a pad of Celite, and the Celite pad was washed with H_2O and MeOH, respectively, several times. The filtrates were concentrated to afford a glassy solid (4.3 mg). ^1H NMR analysis indicated remaining of a few benzyl groups.

The solid above (4.3 mg) was dissolved in a mixture of MeOH/H_2O/HOAc (2 mL/2 mL/0.1 mL HOAc) containing 10% Pd/C (4 mg, wetted with 55% H_2O) and 20% $Pd(OH)_2$/C (4 mg, wetted with 50% H_2O). The resulting mixture was stirred under H_2 atmosphere (1 atm) at rt for 21 h, and was then filtrated through a pad of Celite, and the Celite pad was washed with H_2O and MeOH (1:1, v/v) three times. The filtrates were concentrated to give 64-mer **6** (3.4 mg, 28% over six steps; α/β = 1.3:1 based on ^1H NMR analysis) as a glassy solid or white powder after lyophilization. ^1H NMR (600 MHz, D_2O)

δ 5.05–5.04 (m, 0.56H), 4.92 (s, 31H), 4.84 (s, 32H), 4.23 (s, 29H), 4.04–3.84 (m, 132.44H), 3.79–3.69 (m, 34H), 3.69–3.48 (m, 99H), 3.39–3.28 (m, 32H), 1.28 (d, J = 6.0 Hz, 96H); ^{13}C NMR (151 MHz, D_2O) δ 101.78, 101.69, 97.35, 95.01, 94.59, 81.01, 80.81, 80.63, 78.07, 77.40, 77.30, 74.21, 74.04, 72.24, 71.75, 71.40, 71.22, 68.62, 68.17, 67.98, 67.80, 66.97, 66.15, 62.19, 62.14, 18.27, 18.24, 18.04; MALDI-TOF MS calcd for $C_{384}H_{642}O_{289}Na$ $[M + Na]^+$ 9906.0608, found 9902.6061.

Preparation of 128-mer 14 and 7

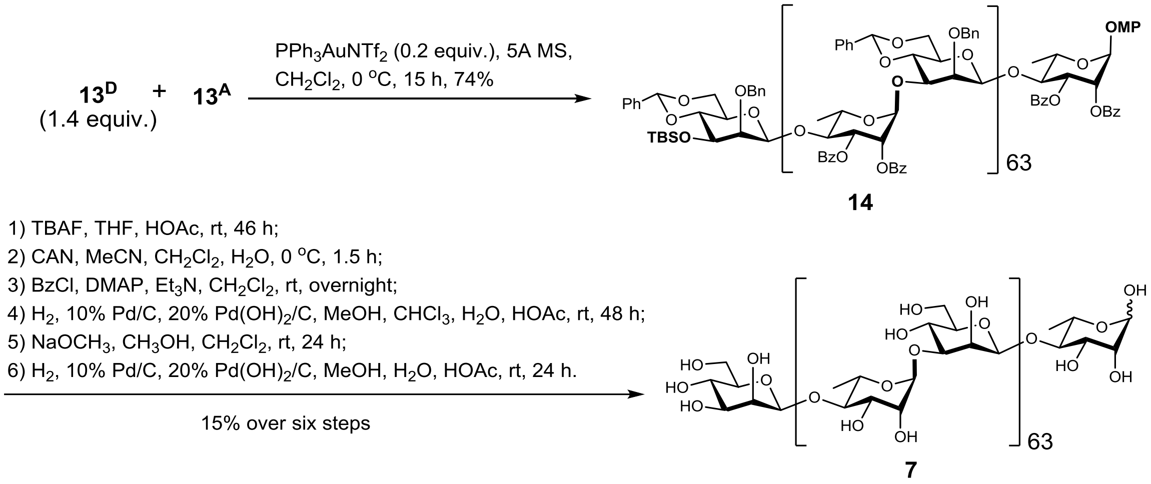

128-mer 14

A mixture of 64-mer donor **13^D^** (113 mg, 5.00 μmol) and acceptor **13^A^** (80 mg, 3.58 μmol) in a Schlenk flask equipped with a Teflon-coated magnetic stir bar was dried in high vacuum at 40 °C overnight. And then 5Å MS (100 mg) and anhydrous CH_2Cl_2 (1 mL) were added. The mixture was stirred at rt for 15 min, and was then cooled to 0 °C, to which $Ph_3PAuNTf_2$ (0.53 mg, 0.72 μmol) was added. The resulting mixture was stirred at 0 °C for 15 h. After addition of Ph_3P (0.5 mg) and Et_3N (4 μL), the mixture was filtrated through a pad of Celite. After removal of solvent, the resulting residue was dissolved in $CHCl_3$ and filtrated through Millipore filter, and then GPC ($CHCl_3$) was performed to give 128-mer **14** (114 mg, 74%) as a white solid. $[\alpha]_D^{25}$ = 29.9 (*c* 1.0, $CHCl_3$); ^{1}H NMR (500 MHz, $CDCl_3$) δ 8.18–7.91 (m, 135H), 7.90–7.71 (m, 134H), 7.70–7.61 (m, 68H), 7.60–7.47 (m, 132H), 7.46–7.36 (m, 135H), 7.35–7.25 (m, 62H), 7.24–7.11 (m, 573H), 7.08–7.01 (m, 68H), 6.91–6.84 (m, 2H), 5.81–5.77 (m, 1H), 5.76–5.37 (m, 128H), 5.35–5.03 (m, 61H),

4.92–4.49 (m, 126H), 4.47–4.19 (m, 109H), 4.18–4.01 (m, 132H), 3.99–3.84 (m, 125H), 3.82–3.60 (m, 65H), 3.60–3.36 (m, 129H), 3.34–3.14 (m, 70H), 1.46 (d, J = 6.1 Hz, 3H), 1.44–0.71 (m, 189H), 0.65 (s, 9H), -0.31 (s, 3H), -0.38 (s, 3H); ^{13}C NMR (126 MHz, $CDCl_3$) δ 165.44, 165.04, 165.01, 164.89, 164.80, 164.72, 155.22, 150.27, 138.47, 137.88, 137.75, 137.54, 137.47, 137.38, 137.18, 133.53, 133.30, 129.81, 129.68, 129.49, 129.39, 129.32, 129.16, 128.94, 128.67, 128.64, 128.56, 128.43, 128.28, 128.09, 128.04, 127.90, 127.72, 127.59, 126.17, 126.15, 126.12, 126.09, 125.93, 125.68, 117.90, 114.67, 103.43, 103.16, 101.87, 101.68, 96.80, 94.84, 94.66, 94.47, 79.04, 78.59, 78.51, 76.24, 75.63, 75.59, 75.40, 75.13, 74.80, 74.01, 72.57, 72.44, 72.31, 72.15, 71.13, 71.01, 70.90, 68.51, 68.04, 67.73, 67.22, 55.67, 25.63, 18.09, 18.01, 17.55, 17.41, 17.34, -4.96, -5.20; MALDI-TOF MS calcd for $C_{2573}H_{2455}O_{706}Si$ $[M + Na]^+$ 44724.3038, found 44725.1974.

128-mer 7

To a solution of 128-mer **14** (45 mg, 1.0 μmol) in anhydrous THF (5 mL), were added HOAc (2 μL, 34.9 μmol) and TBAF (1.0 M in THF, 86.2 μL, 86.2 μmol). The mixture was stirred at rt for 46 h, and was then diluted with CH_2Cl_2 and washed with sat. aq. NH_4Cl and brine, respectively. The organic layer was dried over anhydrous Na_2SO_4 and concentrated *in vacuo* to give a residue.

A solution of the residue above (45 mg, 1.0 μmol) in $MeCN/CH_2Cl_2$ (8 mL/5 mL) was stirred at 0 °C. Ammonium ceric nitrate (242 mg, 0.44 mmol) was dissolved in H_2O (0.5 mL) at 0 °C. The latter solution was added into the former via syringe. The mixture was stirred at 0 °C for 1.5 h, and was then diluted with CH_2Cl_2 and washed with sat. aq. $NaHCO_3$ and brine, respectively. The organic layer was dried over anhydrous Na_2SO_4 and concentrated *in vacuo*. The residue was used in the next step without further purification.

To a mixture of the residue above (45 mg), DMAP (20 mg, 0.16 mmol), and Et_3N (30 μL, 0.21 mmol) in CH_2Cl_2 (2 mL), was added BzCl (12 μL, 104 μmol). The mixture was stirred at rt overnight, and was then diluted with CH_2Cl_2 and washed with sat. aq. $NaHCO_3$ and brine, respectively. The organic layer was dried over anhydrous Na_2SO_4 and concentrated *in vacuo*. The residue was purified by silica gel chromatography ($EtOAc/CH_2Cl_2$, 1:2) to give a white solid (40 mg).

The solid above (40 mg) was dissolved in a mixture of $MeOH/CHCl_3/H_2O/HOAc$ (1.5

mL/3 mL/0.1 mL/0.15 mL) containing 10% Pd/C (80 mg, wetted with 55% H_2O) and 20% $Pd(OH)_2/C$ (80 mg, wetted with 50% H_2O). The resulting mixture was stirred under H_2 atmosphere (1 atm) at rt for 48 h, and was then filtrated through a pad of Celite, and the Celite pad was washed with CH_2Cl_2/MeOH (2:1, v/v) three times. The filtrates were concentrated to give a white solid (30 mg).

The residue above (30 mg) was added into a solution of $NaOCH_3/CH_3OH$ (5 mL, pH = ~11) and CH_2Cl_2 (4 mL). The mixture was stirred at rt for 24 h, and was then neutralized with Amberlyst 15 H^+ resin and filtered. The filtrate was concentrated to give a residue (12 mg). ^1H NMR analysis indicated remaining of a few benzyl groups.

Thus, the solid above (6 mg) was dissolved in a mixture of MeOH/H_2O/HOAc (1.5 mL/1.5 mL/0.1 mL) containing 10% Pd/C (12 mg, wetted with 55% H_2O) and 20% $Pd(OH)_2/C$ (6 mg, wetted with 50% H_2O). The resulting mixture was stirred under H_2 atmosphere (1 atm) at rt for 24 h, and was then filtrated through a pad of Celite, and the Celite pad was washed with H_2O and MeOH three times. The filtrate was concentrated. The residue was purified by gel filtration (Sephadex LH-60, H_2O) to afford 128-mer **7** (3 mg, 15% over six steps) as a glassy solid or white powder after lyophilization. ^1H NMR (600 MHz, D_2O) δ 5.10 (br s, 1H), 4.97 (s, 64H), 4.89 (s, 63H), 4.28 (s, 64H), 4.09–3.90 (m, 321H), 3.84–3.54 (m, 345H), 3.42–3.38 (m, 65H), 1.33 (d, J = 6.2 Hz, 192H), -3.75 (s, 3H); ^{13}C NMR (151 MHz, D_2O) δ 101.78, 101.69, 101.60, 97.35, 81.01, 80.81, 80.73, 80.63, 78.07, 77.39, 77.30, 74.21, 73.86, 72.27, 71.86, 71.75, 71.40, 71.29, 68.61, 68.17, 67.98, 67.79, 66.15, 62.14, 18.27, 18.24, 18.04; MALDI FT-ICR MS calcd for $C_{768}H_{1282}O_{577}Na$ $[M + Na]^+$ 19771, found 19771.

NMR studies on the glycans

General methods. For structural assignments, 1D and 2D ^1H-NMR spectra were recorded in D_2O at 308 K on Bruker 600 MHz equipped with a cryo probe. ROESY and NOESY experiments were recorded using data sets ($t_1 \times t_2$) of 4096 × 800 points with mixing times between 100 ms and 700 ms. Double quantum-filtered phase-sensitive COSY experiments were performed using data sets of 4096 × 800 points. TOCSY experiments were performed with spinlock times of 100 ms, using data sets ($t_1 \times t_2$) of 4096 × 800 points. In all homonuclear experiments the data matrix was zero-filled in both dimensions to give a

matrix of 4K × 2K points and was resolution enhanced in both dimensions by a cosine-bell function before Fourier transformation. HSQC, HSQC-ROESY and HMBC experiments were measured in the ^{1}H-detected mode via single quantum coherence with proton decoupling in the ^{13}C domain, using data sets of 2048 × 600 points. Experiments were carried out in the phase-sensitive mode. A 60 ms delay was used for the evolution of long-range connectivities in the HMBC experiment. In all heteronuclear experiments the data matrix was extended to 4096 × 4096 points using forward linear prediction extrapolation.

DOSY-NMR. DOSY-NMR measurements were performed to determine the self-diffusion coefficients of the 2-, 4-, 8-, 16-, 32-mer (Supplementary Figures 13 and 3c). PFG (Pulsed-field gradient)-NMR is a useful technique to calculate the self-diffusion coefficient (*D*) of the samples in solution and represent as a versatile and fast NMR tool to construct calibration curves[6-8] and determine the average molecular weight of oligo- and polysaccharides. In detail, self-diffusion coefficients, dependent on weight, size and shape of the molecules, were determined by following the decay of the signal intensity of various sugar signals and double-logarithmic plot of D against Mw (Supplementary Figures 13 and 3c) provided a calibration curve described by the least-squares fitted linear equation Log D = -7,873 - (0,486*Log Mw). The calibration curve was then used to evaluate the molecular weights of glycans **6** and **7** (Supplementary Figure 13); DOSY-derived Mw for the glycans up to 64-mer (Mw 9885 *vs.* calcd. 9906) were well consistent with the theoretical value.

PFG-NMR analysis. All the experiments were performed on 100-200 μg of glycans solved in 500 μL of D_2O. 1D ^{1}H NMR experiments for diffusion measurements were performed using a stimulated echo sequence with bipolar gradient pulses and one spoil gradient (stebpgp1s1d) with a diffusion time, Δ from 50-150 ms and a gradient duration varying from 1 to 4 ms. These parameters were optimized, kept as short as possible to minimize T_2 and T_1 losses, the sequence was run as 2D NMR experiment with a linear gradient incremented, in 32 steps, from 2% to 95% (between 1.8 and 32.9 G/cm). The values of diffusion coefficients were calculated from the decay of the signal intensity of different protons. The normalized integral intensities of each proton were fitted to an exponential decay, according to: $I = I_0 \exp -Dk$ with $k=(G*\gamma*\delta)^2*(\Delta-\delta/3-\tau/2)$, where: I is the integral intensity, normalized to the integral obtained at the lowest gradient amplitude; I_0 is the

signal intensity in the absence of an applied magnetic field gradient; γ is the magnetogyric ratio of the proton; G is the strength of the magnetic field gradient pulses; δ is their duration; Δ is the distance between the leading edges of the gradient pulses; and τ is the gradient recovery time. Diffusion rates were extracted from the slope of the straight lines obtained by plotting $\ln(I/I_0)$ against the complex abscissa k.

Conformational studies. Molecular mechanics calculations were performed using the MM3* force field, a dielectric constant of 80 was used. For the disaccharide structure, both Φ and Ψ were varied incrementally using a grid step of 18°, each (Φ, Ψ) point of the map was optimized using 2000 P.R. conjugate gradients. Molecular dynamic simulations were run by using the MM3* force field, bulk water solvation was simulated by using MacroModel generalized Born GB/SA continuum solvent model. All simulations were performed at 298 K, structures were initially subjected to an equilibration time of 300 ps, then a 10000 ps molecular dynamic simulation was performed with a dynamic time-step of 1.5 fs, a bath constant of 0.2 ps and the SHAKE protocol to the hydrogen bonds. Trajectory coordinates were sampled every 2 ps, and a total of 5000 structures were collected for every simulation. Ensemble average-interproton distances were calculated using the NOEPROM program by applying the isolated spin pair approximation as described[9,10]. Solvent-accessible surfaces were calculated with the Surface utility of Maestro. Conformers were visualized with Maestro, Discovery Studio Visualizer and SweetUnitMol.

NMR and conformational analysis of 2- to 128-Mer. The NMR spectra of the synthesized oligomers (2-, 4-, 8- 16- 32-mer) are reported in Supplementary Figures 3-7. A combination of homo- and heteronuclear 2D NMR experiments (DQF-COSY, TOCSY, ROESY, NOESY, HSQC, HSQC-ROESY, and HMBC) allowed to assign the spin systems of rhamnose ***R*** and mannose ***M*** (Supplementary Table 6) of the repeating units and to evaluate the conformational behavior of the synthesized oligomers. The resonances of the internal repeating units were almost coincident, differently from the reducing and terminal non reducing units of the shorter 8-, 4- and 2-mer (Supplementary Table 6), clearly distinguishable. The α-anomeric configurations of the rhamnose units and the β-anomeric configuration of the mannose units were assigned on the basis of $^1J_{C,H}$ coupling constants

values (Supplementary Table 6), whereas the relative and anomeric configurations of the sugar units were confirmed by analysis of *intra*-molecular NOE contacts. Furthermore, the downfield shift of carbon resonances identified the glycosylated positions at *O*-3 of **M** and *O*-4 of **R**; the sequence of the repeating unit was defined by the long-range scalar correlations from the HMBC spectra and the *inter*-residual NOE contacts (Supplementary Figures 3-7 and Table 6) of **M1** with **R4** and **R3** and of **R1** with **M3** and **M2**.

To evaluate the conformational behavior of the oligomers, a molecular mechanics and dynamic simulation analysis was carried out. First, the two **R-3M** (L-Rha-α→(1-3)-D-Man) and **M-4R** (D-Man-β→(1-4)-L-Rha) disaccharides were constructed to evaluate the energetically accessible conformational regions. The corresponding adiabatic energy maps for the glycosidic torsions φ (H1-C1-O-CX') and ψ (C1-O-CX'-HX') are reported in Supplementary Figure 8. The energy maps showed a moderate flexibility around Φ torsion and a higher flexibility around Ψ angle. For each disaccharide, the global minimum values were in accordance with the *exo*-anomeric effect. The lowest energy regions of **R-3M** disaccharide were located around two energy minima at Φ/Ψ glycosidic linkages 55/45 and 35/-50 separated by a low energy barrier and corresponding to exo-Φ/syn-Ψ conformations. As for the **M-4R** disaccharide, the MM calculations suggested the existence of greater flexibility around ψ angle than for φ torsion, two stable conformations around the glycosidic linkages were identified, which were respectively the exo-Φ/syn-Ψ (Φ/Ψ ca. 56/36), exo-Φ/anti-Ψ (Φ/Ψ ca. 50/-177) conformations. Ensemble average *inter*-proton distances for each disaccharide entity were extracted from molecular mechanic calculations and translated into predicted NOEs by a full-matrix relaxation approach and key *inter*-proton distances were evaluated (Supplementary Table 7). The remarkable differences between the *inter*-proton distances of **M1** with **R3**, **R4** and **R5** potentially allowed to discriminate between the two exo-syn and exo-anti conformations adopted by the **M-4R** unit in the analyzed oligomers.

Once the optimal values for Φ and Ψ dihedral angles were estimated (Supplementary Figure 8), 2-mer, 4-mer, 8-mer, 16-mer and 32-mer were built from the global minima, the conformational behavior was studied by using Molecular Dynamic simulation (Supplementary Figures 9-12). MD simulations were thus performed in a GB/SA water solvation model as implemented in Maestro[11]. Trajectories and Φ/Ψ scatter

plots of the glycosidic linkages of some of the oligoes are shown in Supplementary Figures 9-12. The computational models obtained from the MD were then compared to the experimental results; ensemble average *inter*-proton distances were extracted from dynamic simulations and translated into NOE contacts according to a full-matrix relaxation approach. The corresponding average distances obtained for the simulation from $<r^{-6}>$ values were compared to those collected experimentally obtained; an excellent accordance was found (Supplementary Table 7) between the calculated and the experimentally observed distances. A first analysis of MD results showed that trajectories matched those predicted by the MM calculation, with the glycosidic linkages adopting Φ values in accordance with the *exo*-syn anomeric conformation, still showing some flexibility around the glycosidic linkages. Despite the higher flexibility around the **M-4R** glycosidic linkage (Supplementary Figure 8 and Table 7), the *exo*-anti conformation was excluded by the absence of the key NOE contacts and the corresponding *inter*-proton distances predicted by the MM calculations. In fact, the existence of NOE contacts of **M1** exclusively with **R3** and **R4** (see above) allowed to identify the exo-syn conformation as the only adopted by the **M-4R** glycosidic linkage for all the oligomers.

The oligomers tend to form extended, flexible structures, as also confirmed by the few *inter*-residue NOE contacts, diagnostic of the absence of a compact disposition of the sugar backbone. This organization was ascribable to the nature of glycosidic linkage geometries of **M-4R** and **R-3M** units, that introduced a partial twist of the orientation of the sugar chains, that resulted in flat ribbons. On longer chains this probably might lead to the tendency to pack into supramolecular assemblies and could explain the low solubility of higher polysaccharide chains.

MALDI FT-ICR mass spectrometry of 128-mer 7

The mass spectrometry (MS) measurements of 128-mer **7** were performed using a 15T solariX XR Fourier transform ion cyclotron resonance (FT-ICR) mass spectrometer (Bruker Daltonics) equipped with a ParaCell. Matrix-assisted laser desorption/ionization (MALDI) was performed using a mixture of 2,5-dihydroxybenzoic acid (DHB) and 2-hydroxy-5-methoxybenzoic acid (called super-DHB) as a MALDI matrix. MALDI FT-ICR

MS spectra were acquired in the m/z-range 3493.5-30000 with 524288 data points. Every single spectrum was obtained from the acquisition of 200 laser shots at 500 Hz.

The MALDI spotting was performed as follows: 1 μL of 128-mer **7** solution (200 μg/mL in water) was spotted onto an 800 μm AnchorChip MALDI target plate (Bruker Daltonics) then, 2 μL of a super-DHB solution (5 mg/mL in 50% acetonitrile) and 1 μL of glycerol solution (1% v/v in water) were added. The spot was then let to dry at room temperature.

Six different "average" spectra were obtained from the sum of 735, 408, 370, 281, 177, and 142 single spectra, respectively, which were acquired from different MALDI spots. The half-sine window mode was used for the apodization process. The spectra were summed resulting in the spectrum reported in Supplementary Figure **14**. MALDI-in-source decay (ISD) fragment ions of 128-mer **7** dominate the spectrum while the intact 128-mer **7** was detected at a signal-to-noise ratio of 9.5 (inset in Supplementary Figure 14a). The observed average m/z (*i.e.,* m/z 19739) was in agreement with the theoretical m/z value of the intact 128-mer **7** detected as $[M+Na]^+$. Often, the MALDI process leads to the fragmentation of the analyzed molecule in a process called ISD. On the one hand, this process decreases the sensitivity for the intact molecule, on the other hand, it provides valuable structural information. The MALDI-MS analysis of 128-mer **7** resulted in an extensive ISD fragmentation.

Immunological assays on the synthetic glycans and LPS *O*-chain fractions from *Bacteroides vulgatus*

ELISA experiments. 50 μL (PBS 10 mM, pH = 7.4) from a 25 μg/mL *B. vulgatus* LPS solution, were used to coat Nunc MaxiSorp plate (2 h at room temperature). After discarding and washing with PBS (1 × 150 μL), the wells were blocked with 100 μL of 1% BSA (Sigma-Aldrich, lyophilized powder, ≥96%, agarose gel electrophoresis) in PBS at room temperature for 30 min. The blocking solution was discarded and 50 μL of DC-SIGN-Fc at 25 μg mL^{-1} (alone or pre-incubated for 1 h with serial dilutions of the synthetic glycans), in assay buffer [0.5% BSA in calcium and magnesium containing buffer TSM (20 mM tris(hydroxymethyl)aminomethane (Tris)-HCl, pH 8.0; 150 mM NaCl; 1 mM $CaCl_2$; 2 mM $MgCl_2$)] were added to the wells. After 1 h at room temperature, the wells

were washed with PBS (2 × 150 μL) and then 100 μL of anti-human horseradish peroxidase (0.8 μg/mL, life technologies, Novex® Goat anti-Human IgG-HRP) were added. After 30 min at room temperature, wells were washed with PBS (2 × 150 μL). Finally, 100 μL of substrate solution (3,3',5,5'-tetramethylbenzidine, TMB, in citric/acetate buffer, pH = 4, and H_2O_2) were added, and after 3 min incubation at room temperature the reaction was stopped with 50 μL of H_2SO_4 (0.8 M). The optical density (OD) was measured at 450 nm in an ELISA reader. Data were normalized over the signal at 450 nm from the BSA-containing wells.

Supplementary References

(1) J. D. C. Codée, L. H. Hossain, P. H. Seeberger, Efficient installation of β-mannosides using a dehydrative coupling strategy. *Org. Lett.* **7**, 3251–3254 (2005).

(2) Y. Zhu, B. Yu, Highly stereoselective β-mannopyranosylation via the 1-α-glycosyloxy-isochromenylium-4-gold(I) intermediates. *Chem. -Eur. J.* **21**, 8771–8780 (2015).

(3) Y. Zhu, Z. Shen, W. Li, B. Yu, Stereoselective synthesis of β-rhamnopyranosides via gold(I)-catalyzed glycosylation with 2-alkynyl-4-nitro-benzoate donors. *Org. Biomol. Chem.* **14**, 1536–1539 (2016).

(4) D. B. Werz, P. H. Seeberger, Total synthesis of antigen Bacillus anthracis tetrasaccharide - Creation of an anthrax vaccine candidate. *Angew. Chem., Int. Ed.* **44**, 6315–8318 (2005).

(5) Y. Li, G. Manickam, A. Ghoshal, P. Subramaniam, More efficient palladium catalyst for hydrogenolysis of benzyl groups. *Synth. Commun.* **36**, 925−928 (2006).

(6) P. Groves, Diffusion ordered spectroscopy (DOSY) as applied to polymers. *Polym. Chem.* **8**, 6700–6708 (2017).

(7) P. Groves, M. Palczewska, M. D. Molero, G. Batta, F. J. Cañada, J. Jiménez-Barbero, Protein molecular weight standards can compensate systematic errors in diffusion-ordered spectroscopy. *Anal. Biochem.* **331**, 395–397 (2004).

(8) S. Viel, D. Capitani, L. Mannina, A. Segre, Diffusion-ordered NMR spectroscopy: A versatile tool for the molecular weight determination of uncharged polysaccharides. *Biomacromolecules* **4**, 1843–1847 (2003).

(9) W. Li, A. Silipo, L. B. Gersby, M. A, Newman, A. Molinaro, B. Yu, Synthesis of bradyrhizose oligosaccharides relevant to the Bradyrhizobium O-antigen. *Angew. Chem. Int. Ed.* **56**, 2092–2096 (2017).

(10) A. Silipo, F. Di Lorenzo, A. De Felice, A. Vanacore, C. De Castro, D. Gully, R. Lanzetta, M. Parrilli, E. Giraud, A. Molinaro, Structural and conformational study of the O-polysaccharide produced by the metabolically versatile photosynthetic bacterium *Rhodopseudomonas palustris* strain BisA53. *Carbohydr. Polym.* **114**, 384–391 (2014)

(11) https://www.schrodinger.com/maestro
