# Supplementary figures and images for: Chemical synthesis of glycans up to a 128-mer relevant to the O-antigen of Bacteroides vulgatus
Source: Nat Commun. 2020 Aug 18;11:4142. doi: 10.1038/s41467-020-17992-x (PMC7434892; doi:10.1038/s41467-020-17992-x)

## Slide 1
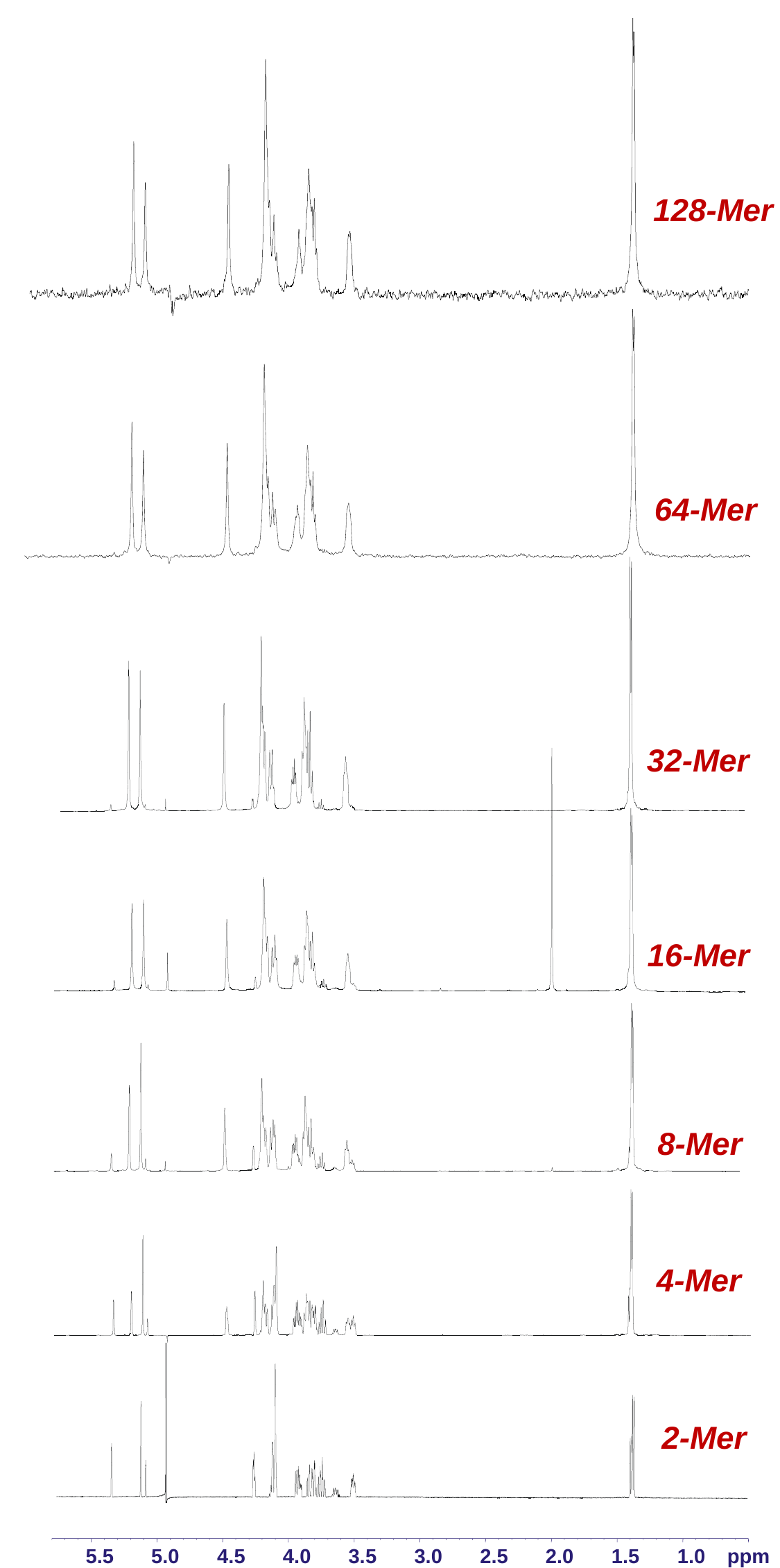

128-Mer
64-Mer
32-Mer
16-Mer
8-Mer
4-Mer
2-Mer
5.5
5.0
4.5
4.0
3.5
3.0
2.5
2.0
1.5
1.0
ppm

Supplement: Supplementary file 3 — Source Data [file 41467_2020_17992_MOESM3_ESM.zip › Source_Data/Figure_3a.pptx]

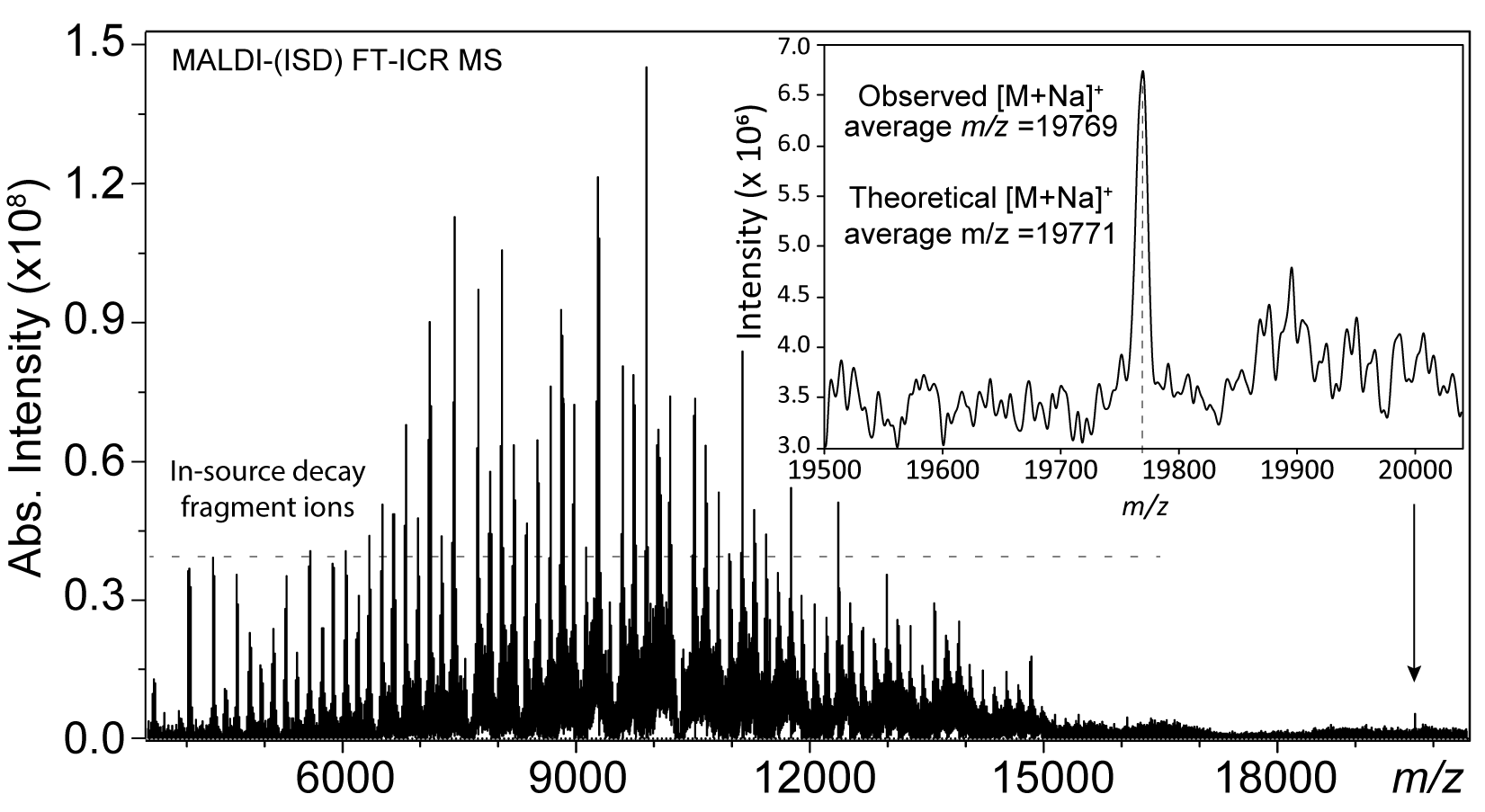

Supplement: Supplementary file 3 — Source Data [file 41467_2020_17992_MOESM3_ESM.zip › Source_Data/Figure_3b.tif]

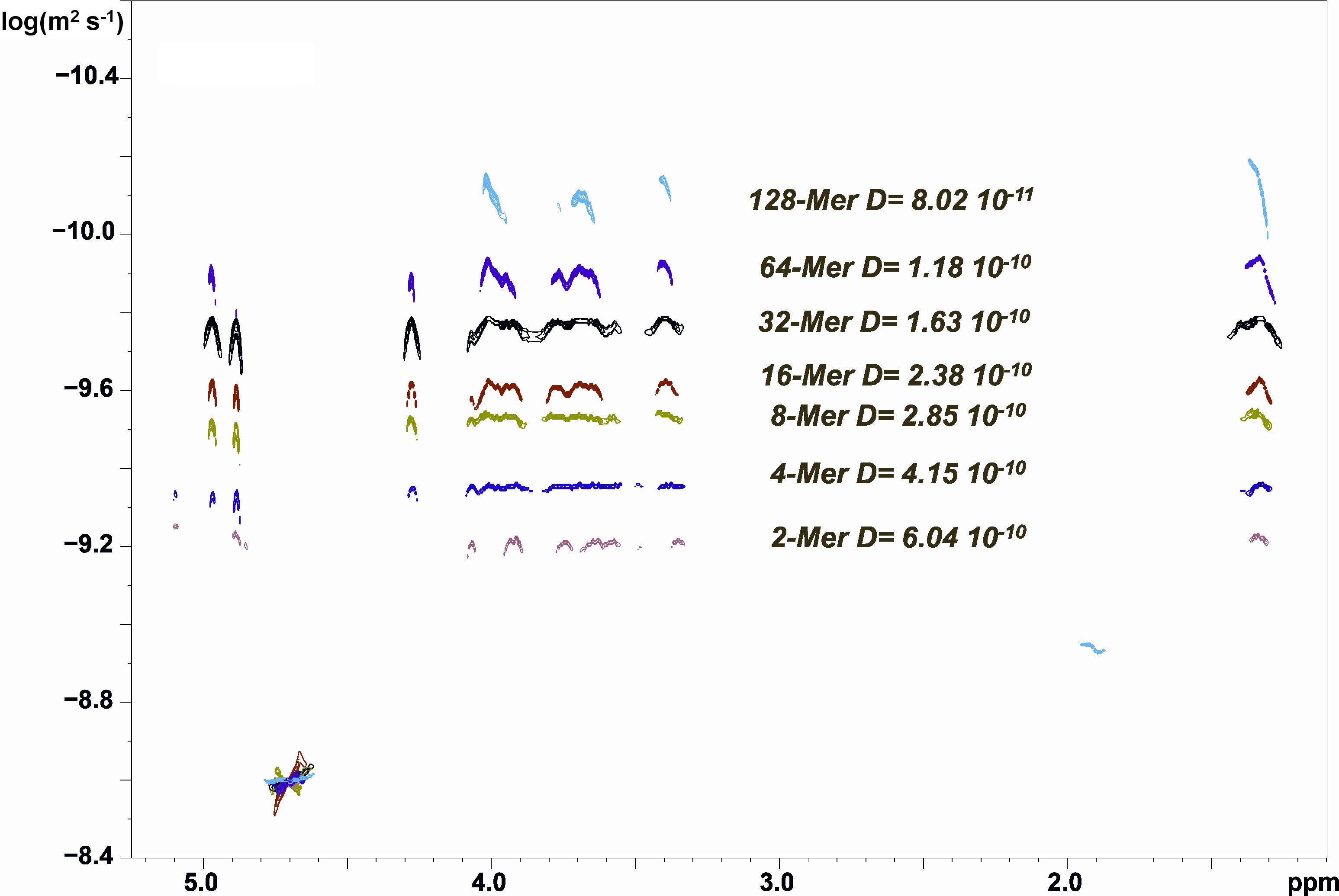

Supplement: Supplementary file 3 — Source Data [file 41467_2020_17992_MOESM3_ESM.zip › Source_Data/Figure_3c.tiff]

## Slide 1
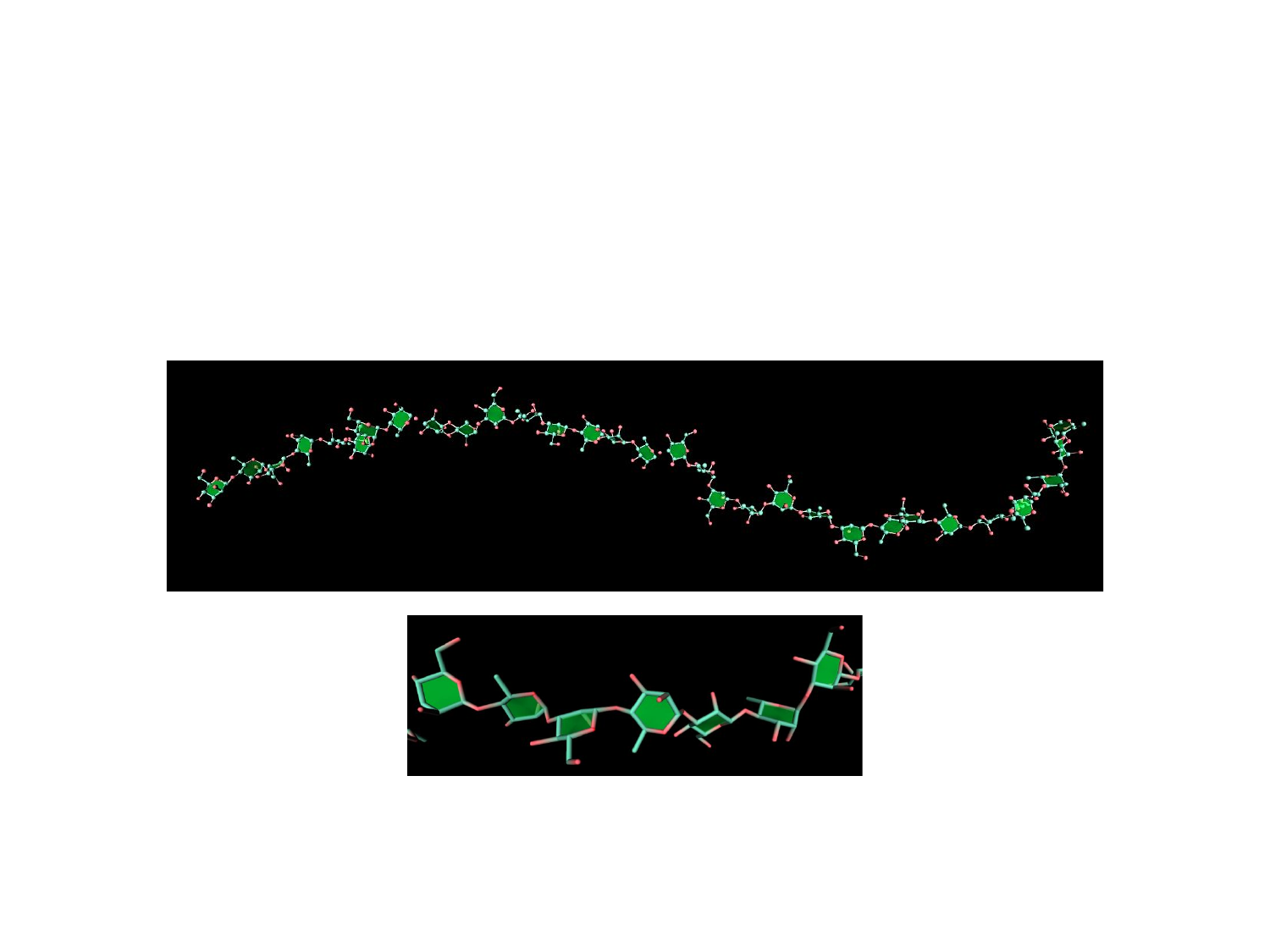

Supplement: Supplementary file 3 — Source Data [file 41467_2020_17992_MOESM3_ESM.zip › Source_Data/Figure_4a.pptx]

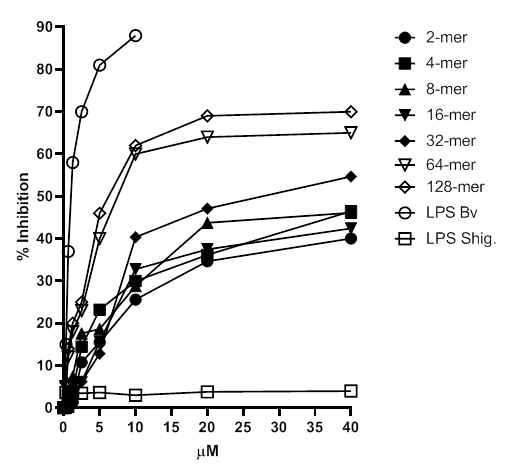

Supplement: Supplementary file 3 — Source Data [file 41467_2020_17992_MOESM3_ESM.zip › Source_Data/Figure_4b.png]
